# Mild Generation of Highly Nucleophilic *N*‐Heterocyclic Carbene Boryl Anion From Neutral sp^2^–sp^3^ Diboron Reagents and Its Applications in Nucleophilic Borylation

**DOI:** 10.1002/anie.7517492

**Published:** 2026-02-03

**Authors:** Weixuan Sun, Peiqi Zhang, Hairong Lyu

**Affiliations:** ^1^ Department of Chemistry The Chinese University of Hong Kong Shatin Hong Kong SAR China; ^2^ Department of Chemistry The Hong Kong University of Science and Technology Kowloon Hong Kong SAR China; ^3^ Shanghai‐Hong Kong Joint Laboratory in Chemical Synthesis The Chinese University of Hong Kong Shatin Hong Kong SAR China

**Keywords:** boron, boron synthon, boryl anion, neutral sp^2^–sp^3^ diboron, nucleophilic borylation

## Abstract

Due to its high activity, the synthesis or generation of the *N*‐heterocyclic carbene (NHC) boryl anion remains challenging, with only limited methods available. Traditional stabilization through bulky ligands or aromatic systems restricts its reactivity as a boron synthon. In this work, we present a straightforward strategy for the in situ generation of an NHC boryl anion at room temperature through the reaction of neutral sp^2^–sp^3^ diboron reagent (NHC)BH_2_Bpin with KO*^t^* Bu. This less sterically hindered and strongly nucleophilic boryl anion demonstrates broad reactivity with diverse electrophiles, including those that are unreactive with other types of boryl anions or traditional diboron reagents. These nucleophilic borylation reactions enable the synthesis of a diverse array of four‐coordinate organoboron compounds. Detailed mechanistic studies and DFT calculations confirm the intermediacy of the NHC boryl anion and provide clear insights into the reaction pathways for the newly discovered reactivities.

## Graphical abstract

A less hindered and highly nucleophilic *N*‐heterocyclic carbene (NHC) boryl anion is generated at room temperature by reacting sp^2^–sp^3^ diboron reagent (NHC)BH_2_Bpin with KO*^t^* Bu. This highly reactive anion demonstrates broad reactivity with various electrophiles, including those traditionally considered inert, enabling their efficient borylation. DFT calculations confirm the anion's high nucleophilicity and offer insights into reaction pathways.

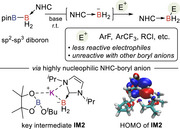

## 1 Introduction

Since E. J. Corey introduced the concept of synthons as retrosynthetic building blocks in 1967 [1, 2], expectations for these reagents have grown well beyond their basic role. Ideally, a modern synthon is easy to prepare from affordable starting materials, avoids highly toxic or hazardous reagents, can be handled without special precautions, and shows good compatibility to enable flexible synthesis. Identifying synthons that satisfy these criteria remains an active area of interest. The same considerations apply in boron chemistry: access to reactive yet user‐friendly boron synthons would further broaden the scope and utility of organoboron compounds.

**SCHEME 1:**
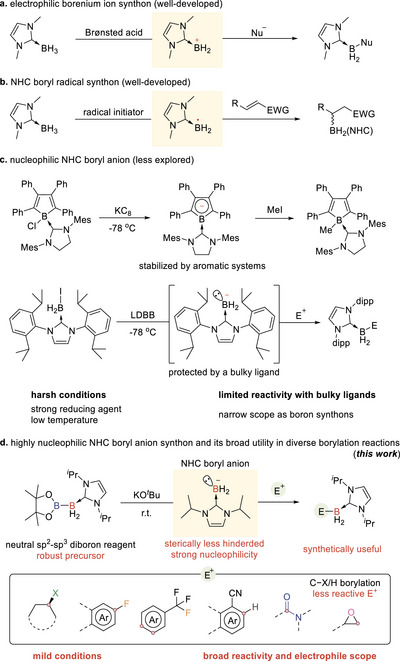
Representative *N*‐heterocyclic carbene stabilized sp^3^ boryl synthons and their applications in borylation reactions and four‐coordinate organoboron compound synthesis.

*N*‐heterocyclic carbene stabilized borane (NHC)BH_3_ is relatively electron‐rich and smoothly undergoes dehydridation with acids to form borenium species (NHC)BH_2_^+^ (Scheme 1a), which serves as an electrophilic boryl intermediate in borylation reactions [3, 4, 5, 6, 7, 8, 9, 10, 11, 12, 13, 14, 15]. Moreover, (NHC)BH_3_ can be subjected to single electron oxidation or hydrogen atom transfer to give a three‐coordinated boryl radical, which often displays nucleophilic properties and is widely used in borylation of electron‐deficient unsaturated substrates via a radical addition process (Scheme 1b) [16, 17, 18, 19, 20, 21, 22, 23, 24, 25, 26, 27]. These developments provide fundamental insights and useful sp^3^ boryl synthons for the preparation of four‐coordinate organoboron compounds.

In sharp contrast, the exploration of NHC boryl anion species remains significantly underdeveloped (Scheme 1c). Due to the weak electronegativity of boron (∼2.0), it has traditionally presented significant challenges in the formation of its nucleophilic boryl anions [28, 29, 30, 31, 32, 33, 34, 35, 36]. The initial success in stabilizing and isolation of these highly active species was marked by Yamashita and Nozaki's pioneering synthesis of boryl lithium compounds in 2006 [37]. Subsequent efforts have explored various stabilization strategies, including the use of bulky ligands [37, 38, 39, 40, 41, 42, 43, 44], incorporation into aromatic systems [37, 41, 43, 44, 45, 46, 47, 48, 49], and the addition of electron‐withdrawing groups [38, 50, 51, 52, 53]. These methods, while effective in stabilizing boryl anions, may inadvertently compromise their nucleophilic capability. In 2010, Braunschweig and coworkers first isolated and characterized a SIMes (SIMes = 1,3‐bis(2,4,6‐trimethylphenyl)‐imidazolin‐2‐ylidene) stabilized borole monoanion through the reduction of corresponding boryl chloride with KC_8_, which exhibited reactivity with electrophile iodomethane [46]. In the same year, Lacôte and coworkers reported an approach for the generation of a boryl anion protected with the bulky IDip (IDip = 1,3‐bis(2,6‐diisopropylphenyl)‐imidazole‐2‐ylidene) via the reduction of boryl iodide (IDip)BH_2_I with lithium di‐*tert*‐butylbiphenylide (LDBB) at −78°C [54]. This (IDip)BH_2_‾ could not be isolated, and its nucleophilic reactivities were demonstrated via the in situ reactions with organic halides and electron‐deficient aromatics.

In this context, the use of a highly nucleophilic NHC boryl anion as a boron synthon is anticipated to enable new borylation reactions and expand product diversity in the synthesis of organoboron compounds. To achieve this, a new strategy and robust boron precursors are greatly needed, which should 1) avoid the use of harsh conditions, such as strong reducing agents and cryogenic temperatures, and 2) exhibit remarkable nucleophilic properties, allowing reactions with a wide range of electrophiles, including those that were previously challenging to access.

## 2 Results and Discussion

### 2.1 Generation of Boryl Anions

Recently, we reported a neutral sp^2^–sp^3^ diborane (I*^i^* Pr)BH_2_Bpin (I*^i^* Pr = 1,3‐diisopropylimidazol‐2‐ylidene), which exhibits good stability toward air and moisture and can be used as a borylation reagent in organic synthesis [55, 56, 57, 58, 59, 60, 61, 62, 63, 64, 65, 66, 67, 68, 69]. In our prior studies, this reagent effectively engaged in copper‐catalyzed borylation of unsaturated substrates through the intermediacy of a key Cu−B(sp^3^) complex [55, 57]. Here, we envision that if the sp^2^–sp^3^ diborane can serve as a robust precursor for the generation of a strongly nucleophilic NHC boryl anion synthon under transition metal‐free conditions to unlock new borylation reactions (Scheme 1d).

**SCHEME 2:**
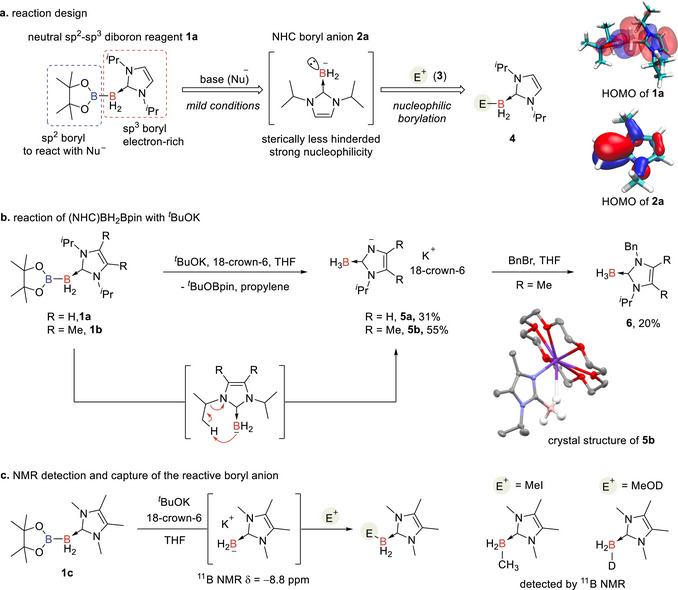
Generation of NHC boryl anion from the neutral sp^2^–sp^3^ diboron reagent (NHC)BH_2_Bpin.

Considering the unique structure and electronic properties of this sp^2^–sp^3^ diboron reagent, specifically, the sp^3^ boryl is stabilized by a less‐bulky, strong electron‐donating I*^i^* Pr ligand and is electron‐rich, while the sp^2^ boryl group is electron‐deficient and electrophilic, we propose that the sp^2^ boryl can be coordinated by a base, triggering the heterolytic cleavage of the B−B bond and leading to the in situ generation of an I*^i^* Pr‐stabilized boryl anion (Scheme 2a). Compared to the reduction of NHC boryl halides with strong reductants of LDBB or KC_8_, this strategy is anticipated to provide a milder approach for generating NHC boryl nucleophiles. Beyond the strong nucleophilicity, the low steric hindrance of the boryl anion may further expand its reactivity toward relatively inert or less electron‐deficient substrates, potentially expanding their applications as boron synthons in organic synthesis (Scheme 1d). In our initial trial, *^t^* BuOK was used as the base to facilitate B─B bond heterolytic cleavage, thereby generating the boryl anion through the elimination of *^t^* BuOBpin. In the reaction of diborane **1a** or **1b** with *^t^* BuOK in the presence of 18‐crown‐6, colorless crystals **5a** and **5b** were obtained. The structure of compound **5b** was further confirmed via X‐ray analysis (Scheme 2b) [70]. It is deduced that the NHC boryl anion was initially formed. However, due to the strong basicity, it rapidly underwent an intramolecular pericyclic reaction, affording the stable potassium amide **5b**, and releasing propylene as a by‐product. Compound **5b** was subsequently reacted with the electrophile benzyl bromide to yield compound **6** [28]. These results indicate that the neutral sp^2^–sp^3^ diborane (NHC)BH_2_Bpin might serve as a suitable precursor for generating the NHC boryl anion under basic conditions.

To detect the possible NHC boryl anion by ^11^B NMR, diboron reagent **1c**, which lacks β‐hydrogens for the pericyclic reaction, was reacted with *^t^* BuOK. A predominant triplet signal at *δ* = −8.8 ppm appeared in the ^11^B NMR spectrum. Comparison with reported chemical shifts for carbene‐stabilized dihydroboryl anions (^11^B NMR *δ* = −4.7 ppm, *δ* = −18.1 ppm) [39, 54] suggests that this signal likely corresponds to a similar species. Upon subsequent treatment with electrophiles such as MeI and MeOD, this triplet was completely converted into signals corresponding to methyl borane and deuterated borane, respectively (Scheme 2c). These results provide evidence for the generation of a reactive boron species that acts as both a strong base and a nucleophile, which is presumably identified as the NHC boryl anion (see Supporting Information, Section 6 and Figure S2 for details).

### 2.2 Nucleophilic Borylation

In this connection, nucleophilic borylation of various electrophiles (E^+^) was subsequently evaluated using (I*^i^* Pr)BH_2_Bpin **1a** as a robust precursor of the NHC boryl anion synthon (Table 1). In the presence of *^t^* BuOK, **1a** underwent a nucleophilic substitution reaction with alkyl chlorides, affording alkyl boranes in satisfactory yields (**4a‐4e**). The S_N_Ar reaction of **1a** with phenyl fluoride and substituted aryl fluorides also went well, producing aryl NHC boranes (**4f‐4i**) in moderate to good yields. The comparable reactivity with aryl fluorides containing electron‐donating groups indicates the strong nucleophilicity of the in situ generated NHC boryl anion.

**TABLE 1:** Nucleophilic borylation using (I*^i^* Pr)BH_2_Bpin (**1a**) as the NHC boryl anion precursor.

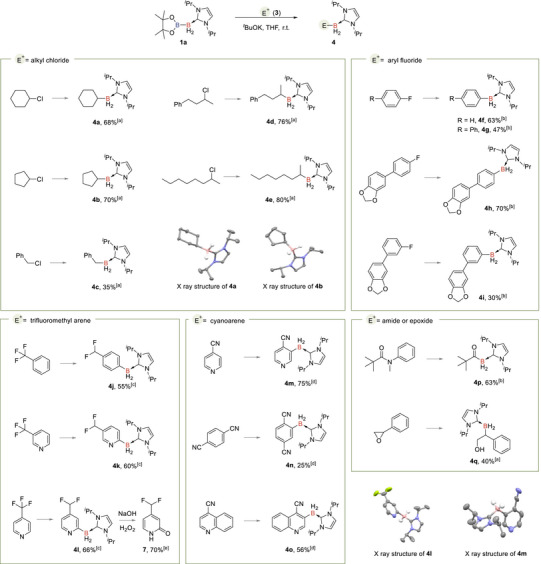

^a^Reaction conditions: reactions were conducted with **3** (0.10 mmol, 1.0 equiv.), **1a** (0.20 mmol, 2.0 equiv.), and *^t^* BuOK (0.25 mmol, 2.5 equiv.) in THF (1 mL) at r.t. under argon for 12 h.

^b^The reactions were conducted with **3** (0.10 mmol, 1.0 equiv.), **1a** (0.20 mmol, 2.0 equiv.), and *^t^* BuOK (0.30 mmol, 3.0 equiv.) in THF/tol (1/1, 0.34 mL) at r.t. under argon for 4 h.

^c^The reactions were conducted with **3** (0.10 mmol, 1.0 equiv.), **1a** (0.15 mmol, 1.5 equiv.), and *^t^* BuOK (0.25 mmol, 2.5 equiv.) in THF (2.5 mL) at r.t. under argon for 12 h.

^d^The reactions were conducted with **3** (0.20 mmol, 2.0 equiv.), **1a** (0.10 mmol, 1.0 equiv.), and *^t^* BuOK (0.25 mmol, 2.5 equiv.) in THF (2.5 mL) at r.t. under argon for 4 h.

^e^The reaction was conducted with **4l** (0.05 mmol, 1.0 equiv.), NaOH (0.20 mmol, 4.0 equiv.), and H_2_O_2_ (30% *w/w* in H_2_O, 40 µL) in MeOH/MeCN (1/1, 0.20 mL) at r.t. for 12 h. All yields are isolated yields.

Additionally, reactions of **1a** with trifluoromethyl arenes were explored. Trifluorotoluene, 4‐(trifluoromethyl)pyridine and 3‐(trifluoromethyl)pyridine reacted well, yielding defluoroborylation products **4j‐4l** in 55% to 66% yields and good regioselectivity. Subsequent oxidation of **4l** using H_2_O_2_ provided 4‐(difluoromethyl)‐2(1*H*)‐pyridinone **7** in 70% yield. It is noteworthy that diboron reagent **1a** was also applied to achieve the direct C3 borylation of cyanoarenes [54] under the metal‐free conditions (**4m‐4o**). Moreover, the in situ generated boryl anion demonstrated nucleophilic reactivity with *N*‐methyl‐*N*‐phenylpivalamide, efficiently producing acyl borane **4p**. Additionally, the nucleophilic attack of the boryl anion on an epoxide resulted in the ring‐opening product **4q** with a yield of 40%.

**SCHEME 3:**
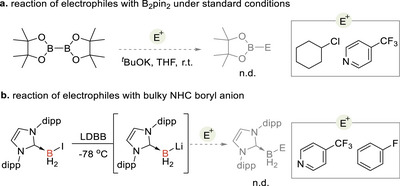
Control experiments using other nucleophilic boryl precursor and boron reagent.

The broad reactivity of the (I*^i^* Pr)BH_2_Bpin (**1a**) is likely attributed to the strong nucleophilicity and low steric hindrance of the (I*^i^* Pr)BH_2_‾ intermediate. Control experiments demonstrated that, under the same reaction conditions, B_2_pin_2_ did not react with alkyl chloride or trifluoromethyl pyridine (Scheme 3a) [71, 72]. Additionally, the in situ generated, more sterically bulky IDipBH_2_Li showed no reactivity with aryl fluoride and trifluoromethyl pyridine (Scheme 3b, see Supporting Information Figure S1 for details).

To further expand the scope of electrophiles to include more inert ones, particularly aryl fluorides and trifluoromethyl arenes, which are unreactive toward other types of boryl anions, we employed **1c** as the boryl anion precursor (Table 2). The in situ generated boryl anion derived from **1c** is proposed to exhibit higher reactivity due to the less sterically hindered IMe_4_ (IMe_4_ = 1,3‐dihydro‐1,3,4,5‐tetramethyl‐2*H*‐imidazol‐2‐ylidene) ligand. S_N_Ar reactions of **1c** with *p*‐fluorotoluene and *p*‐fluoroanisole afforded the corresponding aryl NHC boranes in moderate yields (**4r, 4s**). Notably, the more sterically hindered 2‐fluorobiphenyl and 3‐fluorobiphenyl afforded the desired products **4t** and **4u** in 62% and 66% yields, respectively. Additionally, a thiophenyl‐substituted aryl fluoride was also compatible, yielding **4v** in 40% yield. The use of **1c** further expanded the scope of trifluoromethyl arenes. Substrates substituted with both electron‐withdrawing (−F) and electron‐donating (−OMe) groups selectively yielded para‐defluoroborylation products (**4w, 4x**). Additionally, trifluoromethyl benzenes containing heterocyclic groups, such as benzofuran and thiophene, reacted with **1c** to generate difluoromethyl aryl borane derivatives with moderate yields (**4y, 4z**). Moreover, the trifluoromethyl arene derived from a natural product (L)‐citronellol furnished the corresponding product **4aa**.

**TABLE 2:** Nucleophilic borylation using (IMe_4_)BH_2_Bpin (**1c**) as the NHC boryl anion precursor.

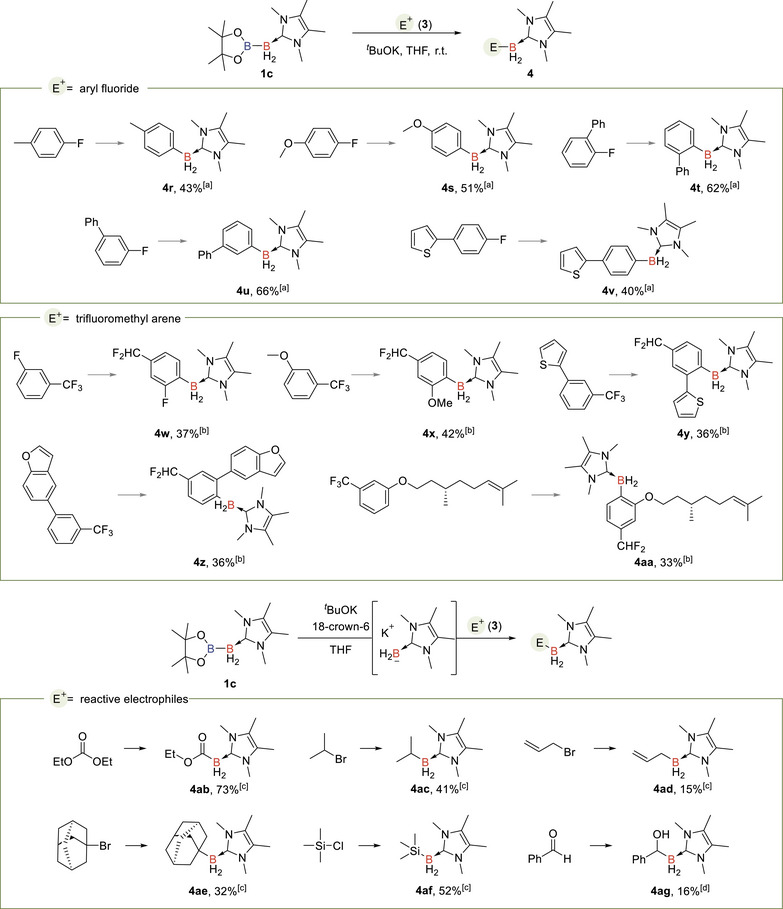

^a^The reactions were conducted with **3** (0.10 mmol, 1.0 equiv.), **1c** (0.20 mmol, 2.0 equiv.), and *^t^* BuOK (0.30 mmol, 3.0 equiv.) in THF/tol (1/1, 0.34 mL) at r.t. under argon for 4 h.

^b^The reactions were conducted with **3** (0.10 mmol, 1.0 equiv.), **1c** (0.15 mmol, 1.5 equiv.), and *^t^* BuOK (0.25 mmol, 2.5 equiv.) in THF (2.5 mL) at r.t. under argon for 12 h.

^c^The reactions were conducted using a premixed solution containing **1c** (0.10 mmol, 1.0 equiv.), 18‐crown‐6 (0.10 mmol, 1.0 equiv.), and *^t^* BuOK (0.15 mmol, 1.5 equiv.) in THF (1.5 mL), followed by the addition of **3** (1.0 mmol, 10 equiv.) at ‐30°C, and stirred at r.t. under argon for 30 min.

^d^The reaction was conducted according to procedure c, using 0.10 mmol of **3**.

In the one‐pot reactions, some reactive electrophiles may directly react with *^t^* BuOK, resulting in low yields or trace amounts of the desired borylation products. To address this, diboron **1c** was first treated with a base in the presence of 18‐crown‐6 to generate the boryl anion, which was subsequently reacted with electrophiles. Using this protocol, various alkyl bromides reacted to afford alkyl boranes (**4ac‐4ae**). Diethyl carbonate selectively yielded the mono‐substituted acyl borane **4ab** with satisfactory efficiency, indicating efficient conversion of **1c** to the boryl anion in the first step. Additionally, treatment with TMSCl successfully generated the corresponding silylborane **4af**, while the reaction of **1c** with an aldehyde yielded the α‐hydroxyl borane product **4ag**, albeit with a lower yield of 16%.

### 2.3 Mechanistic Study and DFT Calculation

**SCHEME 4:**
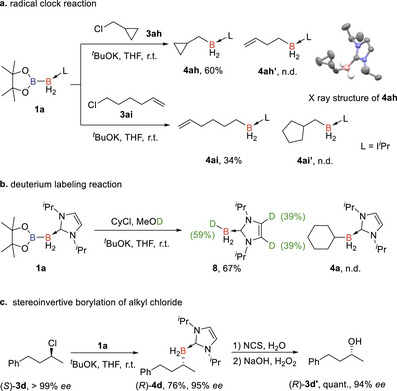
Mechanistic study of the S_N_2 borylation.

To gain deeper insight into the reaction mechanism and further verify the involvement of boryl anion in the reaction pathway, we first performed the mechanistic study of the S_N_2 borylation reaction. A radical clock experiment was conducted using (chloromethyl)cyclopropane (**3ah**) as the electrophile [73, 74, 75, 76]. The alkyl borane **4ah** was obtained as the sole product, while the ring‐opening product **4ah’** was not detected. Similarly, the reaction with 6‐chloro‐1‐hexene did not afford the cyclized product **4ai’**. These results suggest that a radical pathway might not be involved in the reaction process (Scheme 4a).

We then examined the generation of the basic NHC boryl anion via a quenching experiment. The reaction mixture of **1a**, KO*^t^* Bu, and cyclohexyl chloride was immediately quenched with MeOD before the S_N_2 reaction started. Instead of the detection of **4a**, deuterated NHC borane **8** was obtained in 67% yield, with a deuteration rate of 59% on boron. A 39% deuteration rate was also observed on the NHC, which indicates a possible deprotonation of the C−H on NHC by the boryl anion. Since the hydrogen atom transfer (HAT) from MeOD by a boron radical species is not feasible, this result suggests that the reaction is likely an acid–base reaction involving a basic boryl anion (Scheme 4b).

More importantly, when the enantiopure alkyl chloride (*S*)‐**3d** (> 99% ee) was reacted with diboron **1a** under standard conditions, the stereoinvertive borylation product (*R*)‐**4d** was obtained in 76% yield with 95% ee. The configuration of (*R*)‐**4d** was confirmed by in situ oxidation, resulting the alcohol (*R*)‐**3d’** (Scheme 4c) with 94% ee. This result provides solid evidence for the S_N_2‐type mechanism in the nucleophilic borylation of alkyl chlorides and further supports the generation of an NHC‐boryl anion in the reaction pathway. It also represents the first example of transition‐metal‐free, stereospecific nucleophilic borylation of unactivated alkyl halides [77, 78, 79, 80], potentially offering a straightforward method for the synthesis of chiral alkyl boranes.

**FIGURE 1:**
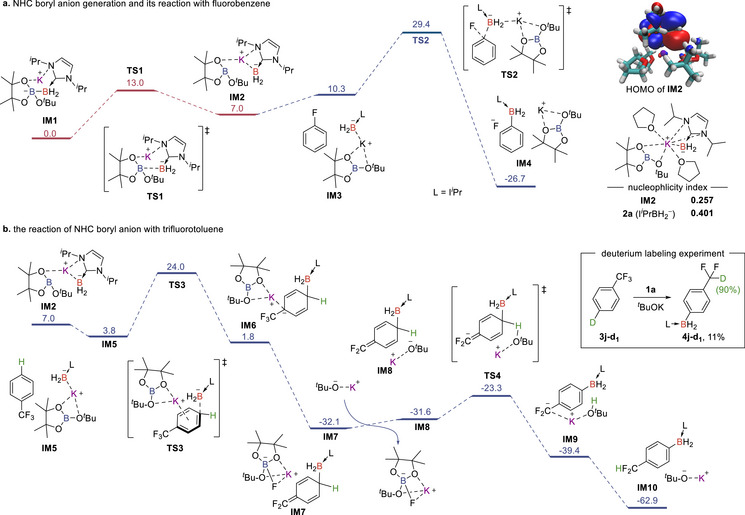
Mechanistic investigation and DFT‐calculated pathways for NHC boryl anion generation and its reactions with phenyl fluoride and trifluorotoluene. DFT computation on M06‐L / 6–31G(d,p) level. Explicit THF molecules around the potassium ion are omitted for clarity. Relative free energies are given in kcal/mol.

DFT computation was carried out to further understand the reaction mechanism (Figure 1), especially for the unique reactivities exhibited by the boryl anion **2a** toward aryl fluorides and trifluoromethyl arenes, which have not been observed and explored with other types of boryl anions [37, 38, 39, 40, 41, 42, 43, 44, 45, 46, 47, 48, 49, 50, 51, 52, 53, 54].

The DFT calculations were initiated with the borate intermediate **IM1**, formed from the reaction of KO*^t^* Bu and diborane **1a**. The calculated energy barrier for B─B bond heterolytic cleavage was relatively low (13.0 kcal/mol), leading to the formation of the boryl potassium complex **IM2**. The highest occupied molecular orbital (HOMO) of **IM2** was primarily localized on the p orbital of the boron atom bonded to the NHC, indicating a significant negative charge on the boron atom. Furthermore, natural fragment bonding orbital (NFBO) [81] analysis revealed a high electron distribution of 0.92 on boron, demonstrating its superior anionic character (see Figure S3 for details). The calculated nucleophilicity index [82] of **IM2** (0.257) and the anionic fragment **2a** (0.401) are also higher than those of other reported boryl anion complexes [37, 39, 51, 54] (see Figure S4 for details). These computational findings align well with the strong nucleophilicity and special reactivities of the boron anion observed experimentally.

When boron anion **IM2** reacted with phenyl fluoride, it readily attacked phenyl fluoride via an S_N_Ar‐type mechanism to produce the arylborane product. A single transition state was identified, with no intermediate observed, as verified by an intrinsic reaction coordinate (IRC) scan (Figure 1a).

For the reactions with different trifluoromethyl arenes, distinct reaction mechanisms were calculated (Figure 1b and Supporting Information Figure S5). When trifluorotoluene was used as the substrate, the boryl anion initially attacked the electron‐deficient para position, resulting in a dearomative 1,4‐addition to form intermediate **IM6**. **IM6** sequentially underwent defluoronation facilitated by *^t^* BuOBpin to give the neutral intermediate **IM7**. A tandem sequence of deprotonation by *^t^* BuOK, aromatization, and protonation occurred to yield the final product **4j**. The nucleophilic attack of boryl anion on trifluorotoluene exhibited the highest energy barrier (24.0 kcal/mol), making it the rate‐determining step. A deuterium labeling experiment further supported the proposed mechanism. The reaction of **1a** with 4‐D‐C_6_H_4_CF_3_ (**3j‐d_1_**) afforded **4j‐d_1_**, with the deuterium transferred to the difluoromethyl group in 90% deuteration rate, providing evidence of the reaction mechanism (Figure 1b).

For the α‐borylation of 4‐(trifluoromethyl)pyridine, although the exact mechanism remains unclear, a proposed reaction pathway has been provided, supported by a deuterium‐labeling experiment (Figure S5). The boryl anion **IM2** preferentially attacked the most electron‐deficient ortho‐position, resulting in the formation of **IM14**. The deuterium labeling experiment with substrate **3l‐d_2_** [83] resulted in a 48% deuteration rate at the C5‐position of **4l‐d_2_**, indicating the occurrence of a possible [1,4]‐hydrogen shift of **IM14**. Our calculations indicate that the coordination of 4‐(trifluoromethyl)pyridine to the −Bpin unit in **IM2** (**IM11**) might facilitate the nucleophilic addition of boryl anion, reducing the energy barrier to 5.4 kcal/mol (**TS6**) (see Figure S5 for details).

## 3 Conclusion

In conclusion, a reactive NHC boryl anion was generated under mild conditions and applied as a highly nucleophilic boron synthon, exhibiting broad reactivity with various organic substrates. The neutral sp^2^–sp^3^ diboron reagent (NHC)BH_2_Bpin proved to be a versatile boron reagent and precursor in nucleophilic borylation reactions, enabling the discovery of unique reactivities that are difficult to achieve with traditional diboron reagents.

## Conflicts of Interest

The authors declare no conflicts of interest.

## Supporting information




**Supporting File 1**: The authors have cited additional references within the Supporting Information [84–98].

## Data Availability

The data that supports the findings of this study are available in the supplementary material of this article.
