# Supplementary material for: Mild Generation of Highly Nucleophilic N‐Heterocyclic Carbene Boryl Anion From Neutral sp2–sp3 Diboron Reagents and Its Applications in Nucleophilic Borylation
Source: Angew Chem Int Ed Engl. 2026 Feb 3;65(11):e7517492. doi: 10.1002/anie.7517492 (PMC12970507; doi:10.1002/anie.7517492)
Supplement: Supplementary file 1 — Supporting File 1: The authors have cited additional references within the Supporting Information [84–98]. [file ANIE-65-e7517492-s001.pdf]

Mild Generation of Highly Nucleophilic *N*-Heterocyclic Carbene Boryl Anion from Neutral sp^2-sp^3 Diboron Reagents and Its Applications in Nucleophilic Borylation

Weixuan Sun,[1†] Peiqi Zhang,[2†] Hairong Lyu[1,3*]

[1]Department of Chemistry, The Chinese University of Hong Kong, Shatin, N.T., Hong Kong SAR, China
[2]Department of Chemistry, The Hong Kong University of Science and Technology, Clear Water Bay, Kowloon, Hong Kong SAR, China
[3]Shanghai-Hong Kong Joint Laboratory in Chemical Synthesis, The Chinese University of Hong Kong, Shatin, N.T., Hong Kong SAR, China

*Email: hrlyu@cuhk.edu.hk

Table of Contents

1. **General Information..2**
2. **Reagents and Substrates..2**
3. **Optimization of Reaction Conditions..8**
4. **Nucleophilic Borylation Using 1a and 1c as NHC Boryl Anion Precursors....9**
5. **Control Experiments..25**
6. **Mechanistic Study..26**
7. **Computational Details..34**
8. **X-ray Data..35**
9. **NMR Spectra..42**
10. **HPLC Analysis of Enantioenriched Substrates..123**
11. **References..126**

1. General Information

Unless noted otherwise, all solvents were dried and distilled from sodium benzophenone ketyl prior to use. Reaction temperatures were reported as the temperatures of the bather surrounding the flasks or vials. Sensitive reagents and solvents were transferred under nitrogen into an argon-filled glovebox with standard techniques. Analytical thin-layer chromatography (TLC) was carried out using 0.2 mm commercial silica gel plates (TLC silica gel 60 F254 glass plates). Vials (13 x 80 mm pressure vessel, heavy wall (4 mL) and Schlenk tube, with vacuum valve and a Teflon screw cap (10 mL)) were purchased from Synthware glass and flame-dried or put in an oven overnight. High resolution mass spectra (HRMS) were obtained on an Thermo Q Exactive Focus Orbitrap Mass Spectrometer or a Waters Xevo G2-XS Tof mass spectrometer and are reported as m/z (relative intensity). Accurate masses are reported for the molecular ion $[M+H]^+$, $[M+Na]^+$. Infrared spectra were recorded on a Bruker Alpha spectrometer using a setup with a ZnSe crystal in the attenuated total reflection (ATR) mode and reported in terms of frequency of absorption (cm^{-1}). Nuclear magnetic resonance spectra (^1H NMR, ^2H NMR, ^{13}C NMR, ^{11}B NMR, ^{19}F NMR) were recorded with a Bruker Avance III 400 NMR Spectrometer (400 MHz, ^1H at 400 MHz, ^{13}C at 101 MHz, ^{11}B at 128 MHz, ^2H at 61 MHz) and a Bruker Avance III HD 500 NMR Spectrometer (500 MHz, ^1H at 500 MHz, ^{13}C at 126 MHz, ^{11}B at 160 MHz, ^{19}F at 471 MHz). Chemical shifts are reported in parts per million (ppm, δ), downfield from tetramethylsilane (TMS, δ = 0.00 ppm) and are referenced to residual solvent ($CDCl_3$, δ = 7.26 ppm (^1H) and 77.16 ppm (^{13}C); C_6D_6, δ = 7.15 ppm (^1H) and 128.06 ppm (^{13}C); CD_3CN, δ = 1.96 ppm (^1H)). All the ^{11}B chemical shifts were referenced to external $BF_3{\cdot}OEt_2$ (0.00 ppm). All the ^{19}F chemical shifts were not referenced. Coupling constants were reported in Hertz (Hz). Data for ^1H NMR spectra were reported as follows: chemical shift (ppm, referenced to protium; s = singlet, d = doublet, t = triplet, q = quartet, h = sextet, hept = septet, dd = doublet of doublets, dt = doublet of triplets, m = multiplet, br = broad, coupling constant (Hz), and integration). GC-MS spectra were recorded on an Agilent 7890B GC System equipped with Agilent5977B GC/MSD detector. Enantiomeric excess was determined by chiral HPLC (Waters 1525 System with 2998 PDA and Thermo HPLC UltiMate 3000), equipped with Chiracel OX-H column (0.46 cm × 25 cm), Chiracel OJ-3 column (0.46 cm × 25 cm) and Chiralpak AD-H column (0.46 cm × 25 cm). Optical rotation was determined using a Rudolph Autopol II Polarimeter.

2. Reagents and Substrates

Diboron reagents **1a**, **1b** and **3l-d₂** were prepared according to literature methods.[56,57,83] Compounds **1c, 3d,** (*S*)-**3d, 3e, 3g, 3h, 3i, 3j-*d*₁, 3p, 3v, 3y, 3z** and **3aa** were prepared according to the following procedures. The others were commercially available and were used as received.

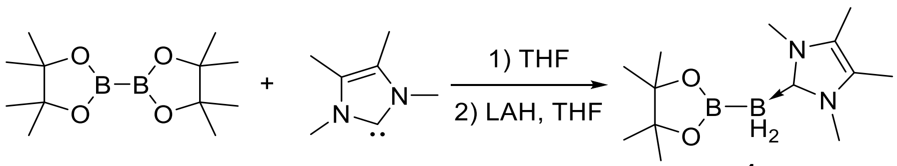

1c

Synthesis of compound 1c. To a 100 mL round-bottom flask charged with bis(pinacolato)diboron (1.27 g, 5 mmol, 1.0 equiv.) and THF (50 mL), 1,2,3,4-tetramethylimidazol-2-ylidene (620.9 mg, 5 mmol, 1.0 equiv.) was added slowly. The mixture was stirred at room temperature for 6 h and then cooled down to -36°C. $LiAlH_4$ (151.6 mg, 0.8 mmol, 0.8 equiv.) was added in small portions into the cooled reaction mixture. The reaction mixture was then allowed to warm up to room temperature and stir overnight under argon. The resulting mixture was filtered thought a pad of Celite and washed with ethyl acetate. The filtrate was concentrated to dryness under vacuum. The residue was further purified by flash column chromatography on triethylamine-basified aluminum oxide to give the product as white solid.

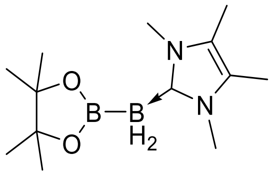

1c: Yield: 13%, white solid, m.p. = 85 – 92 °C, R_f = 0.4 (hexane/ethyl acetate = 3/1).
^{1}H NMR (400 MHz, $CDCl_3$) δ 3.48 (s, 6H), 2.00 (s, 6H), 1.11 (s, 12H).
^{13}C NMR (101 MHz, $CDCl_3$) δ 122.6, 80.8, 32.4, 25.1, 8.8.
^{11}B NMR (128 MHz, $CDCl_3$) δ 41.3 (s, 1B), -39.4 (t, J = 64.9 Hz, 1B).
HRMS (ESI): Calcd for $C_{13}H_{26}B_2N_2O_2Na^+$ $[M+Na]^+$: 287.2073, found: 287.2071.
IR (neat, cm^{-1}): v 2976, 2926, 2856, 2319, 1660, 1470, 1458, 1442, 1394, 1369, 1263, 1233, 1141, 1090, 1007.

Synthesis of substrates 3d, 3e and (*S*)-3d

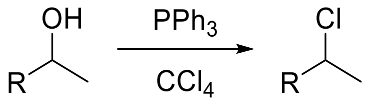

To a 10 ml Schleck tube equipped with a Teflon stir bar, alkyl alcohol (2 mmol, 1.0 equiv.) and PPh_3 (786.9 mg, 3.0 mmol, 1.5 equiv.) was dissolved in CCl_4 (2.0 ml). The reaction mixture was heated to reflux then filtered after 3 hours. The filtrate was purified by column chromatography on silica gel (hexane) to give the product alkyl chloride as a colorless oil.

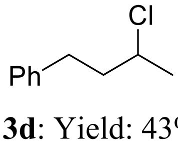

3d: Yield: 43%. Colorless oil.

^{1}H NMR (400 MHz, $CDCl_3$) δ 7.33 – 7.27 (m, 2H), 7.25 – 7.16 (m, 3H), 4.00 (h, *J* = 6.5 Hz, 1H), 2.86 (m, 1H), 2.75 (m, 1H), 2.02 (m, 2H), 1.54 (d, *J* = 6.5 Hz, 3H).
The NMR data match with those reported in literature.[84]

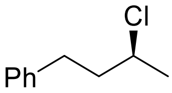

(*S*)-**3d**: Yield: 60%. > 99% ee. Prepared from (*R*)-**3d'**. The enantiomeric excess was determined using chiral HPLC (Chiralcel OJ-3, n-hexane, flow rate: 1.0 mL/min). Other analytic data was identical to the racemic product **3d**.

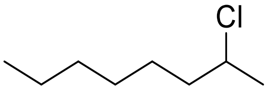

3e: Yield: 37%. Colorless oil.
^{1}H NMR (400 MHz, $CDCl_3$) δ 4.10 – 3.95 (m, 1H), 1.75 – 1.65 (m, 2H), 1.50 (d, *J* = 6.5 Hz, 3H), 1.48 – 1.34 (m, 2H), 1.33 – 1.24 (m, 6H), 0.91 – 0.86 (m, 3H).
The NMR data match with those reported in literature.[85]

Synthesis of substrates 3g-3i, 3ac, 3af and 3ag

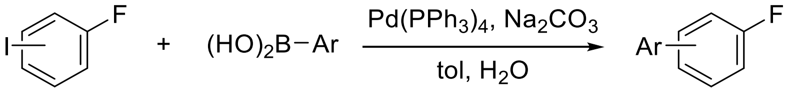

In an oven-dried 100 mL Schlenk flask equipped with a Teflon screw cap and a Teflon stir bar, 3-fluoroiodobenzene or 4-fluoroiodobenzene (444.0 mg, 2.0 mmol, 1.0 equiv.), corresponding boronic acid (2.4 mmol, 1.2 equiv.), $Pd(PPh_3)_4$ (115.6 mg, 0.1 mmol, 0.05 equiv.) and Na_2CO_3 (699.6 mg, 6.6 mmol, 3.3 equiv.) was mixed with toluene/water (1/1, 6.6 mL) under argon. The reaction mixture was heated to reflux overnight. Upon completion, the mixture was cooled to room temperature, diluted by dichloromethane (10 mL), and extracted with dichloromethane (20 mL x 3). The combined organic layers were dried over anhydrous Na_2SO_4, filtered and concentrated under vacuum. The residue was purified by column chromatography on silica gel (hexane) to afford the product.

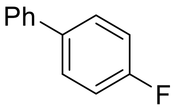

3g: Yield: 77%. White solid.
^{1}H NMR (500 MHz, $CDCl_3$) δ 7.53 – 7.56 (m, 4H), 7.44 (t, *J* = 7.7 Hz, 2H), 7.35 (t, *J* = 7.2, 1.6 Hz, 1H), 7.13 (t, *J* = 8.7 Hz, 2H).
The NMR data match with those reported in literature.[86]

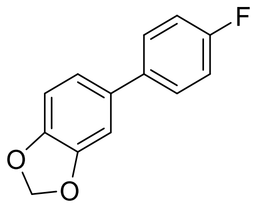

3h: Yield: 65%. White solid.
^{1}H NMR (500 MHz, $CDCl_3$) δ 7.46 (dd, *J* = 8.6, 5.4 Hz, 2H), 7.09 (t, *J* = 8.7 Hz, 2H), 7.00 (d, *J* = 10.7 Hz, 2H), 6.87 (d, *J* = 7.9 Hz, 1H), 6.00 (s, 2H).
The NMR data match with those reported in literature.[87]

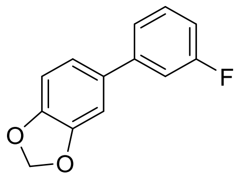

3i: Yield: 44%. White solid.
^{1}H NMR (500 MHz, $CDCl_3$) δ 7.36 (td, *J* = 8.0, 6.0 Hz, 1H), 7.29 (dt, *J* = 7.8, 1.3 Hz, 1H), 7.21 (dt, *J* = 10.3, 2.1 Hz, 1H), 7.08 – 7.03 (m, 2H), 7.03 – 6.94 (m, 1H), 6.88 (d, *J* = 8.5 Hz, 1H), 6.01 (s, 2H).
The NMR data match with those reported in literature.[88]

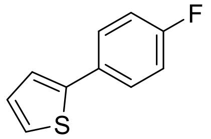

3v: Yield: 61%. White solid.
^{1}H NMR (400 MHz, $CDCl_3$) δ 7.57 (dd, *J* = 8.7, 5.3 Hz, 2H), 7.29 – 7.26 (m, 1H), 7.25 – 7.23 (m, 1H), 7.11 – 7.04 (m, 3H).
The NMR data match with those reported in literature.[89]

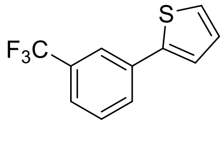

3y: Yield: 80%. Colorless oil.
^{1}H NMR (400 MHz, $CDCl_3$) δ 7.83 (s, 1H), 7.77 (d, *J* = 8.8 Hz, 1H), 7.69 – 7.56 (m, 1H), 7.55 – 7.51 (m, 2H), 7.48 – 7.42 (m, 1H), 7.42 – 7.40 (m, 1H).
The NMR data match with those reported in literature.[90]

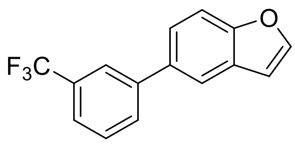

3z: Yield: 53%. Colorless oil, $R_f = 0.8$ (hexane).
^{1}H NMR (500 MHz, $CDCl_3$) δ 7.87 (s, 1H), 7.82 – 7.77 (m, 2H), 7.69 (d, *J* = 2.3 Hz, 1H), 7.63 – 7.59 (m, 2H), 7.57 (d, *J* = 7.6 Hz, 1H), 7.54 – 7.50 (m, 1H), 6.84 (d, *J* = 2.5 Hz, 1H).
^{13}C NMR (126 MHz, $CDCl_3$) δ 155.0, 146.0, 142.6, 135.1, 131.3 (q, $J_{C\text{-}F}$ = 31.5 Hz), 130.8, 129.3, 128.3, 124.4 (q, $J_{C\text{-}F}$ = 273.4 Hz), 124.3 (q, $J_{C\text{-}F}$ = 3.8 Hz), 124.0, 123.7 (q, $J_{C\text{-}F}$ = 3.8 Hz), 120.1, 111.9, 106.9.
^{19}F NMR (471 MHz, $CDCl_3$) δ -62.5 (s, 3F).
HRMS (ESI): Calcd for $C_{15}H_9F_3O^+$ $[M]^+$: 262.0600, found: 262.0608.
IR (neat, cm^{-1}): ν 1614, 1449, 1331, 1229, 1120.

Synthesis of substrate 3p

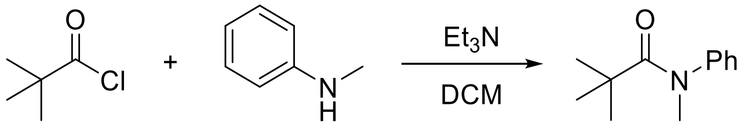

At 0 °C, to a stirred solution of *N*-methyl aniline (2.0 mmol, 1.0 equiv.) and triethylamine (2.2 mmol, 1.1 equiv.) in 2.0 mL of anhydrous dichloromethane was added dropwise pivaloyl chloride (2.1 mmol, 1.05 equiv.). The mixture was stirred at room temperature for 2 hours. Then, HCl solution (1 M, 2.0 mL) was added. The organic layer was extracted with dichloromethane (10 mL x 3), washed with saturated $NaHCO_3$ (aq. 3.0 mL) and brine (3.0 mL), dried over anhydrous Na_2SO_4, filtered and evaporated under reduced pressure. The residue was purified by column chromatography on silica gel (hexane/ethyl acetate = 3/1).

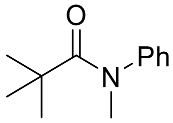

3p: Yield: 55%. White solid.
^{1}H NMR (500 MHz, $CDCl_3$) δ 7.38 (d, *J* = 6.8 Hz, 2H), 7.33 (d, *J* = 6.1 Hz, 1H), 7.21 (d, *J* = 7.9 Hz, 2H), 3.21 (s, 3H), 1.03 (s, 9H).
The NMR data match with those reported in literature.[91]

Synthesis of substrate 3aa

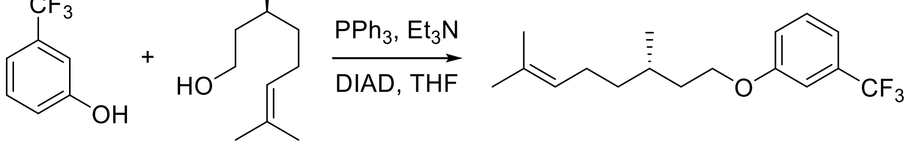

In a glove box, 3-(trifluoromethyl)phenol (245 μL, 2.0 mmol, 1.0 equiv.), (L)-citronellol (440 μL, 2.4 mmol, 1.2 equiv.), PPh_3 (525 mg, 2.0 mmol, 1.0 equiv.) and Et_3N (280 μL, 2.0 mmol, 1.0 equiv.) was dissolved in 2 mL dry THF. The mixture was cooled to 0 °C and DIAD (395 μL, 2.0 mmol, 1.0 equiv.) was added dropwise. After

addition, the reaction mixture was stirred overnight at room temperature. Then, saturated NH_4Cl solution (2.0 mL) was added. The organic layer was extracted with diethyl ether (5 mL x 3), dried over anhydrous Na_2SO_4, filtered and evaporated under reduced pressure. The residue was purified by column chromatography on silica gel (hexane).

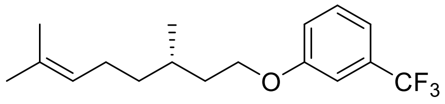

3aa: Yield 65%. Colorless oil. R_f = 0.6 (hexane).

^{1}H NMR (500 MHz, $CDCl_3$) δ 7.37 (d, J = 7.4 Hz, 1H), 7.19 (d, J = 7.9 Hz, 1H), 7.12 (s, 1H), 7.06 (d, J = 8.5 Hz, 1H), 5.11 (t, J = 8.5 Hz, 1H), 4.02 (q, J = 7.6, 7.2 Hz, 2H), 2.01 (dt, J = 15.8, 7.8 Hz, 2H), 1.89 – 1.82 (m, 1H), 1.69 (s, 3H), 1.61 (s, 3H), 1.57 – 1.55 (m, 1H), 1.45 – 1.35(m, 1H), 1.29 – 1.15 (m, 2H), 1.00 – 0.93 (m, 3H).

^{13}C NMR (126 MHz, $CDCl_3$) δ 159.4, 131.9 (q, $J_{C\text{-}F}$ = 31.5 Hz), 131.6, 130.0, 124.7, 124.2 (q, $J_{C\text{-}F}$ = 273.4 Hz), 118.1, 117.3 (q, $J_{C\text{-}F}$ = 5.0 Hz), 111.4 (q, $J_{C\text{-}F}$ = 5.0 Hz), 66.7, 37.2, 36.1, 29.6, 25.9, 25.6, 19.7, 17.8.

^{19}F NMR (471 MHz, $CDCl_3$) δ -62.7 (s, 3F).

HRMS (ESI): Calcd for $C_{17}H_{24}F_3O^+$ $[M+H]^+$: 301.1774, found: 301.1777.

IR (neat, cm^{-1}): ν 2921, 1596, 1451, 1326, 1167, 1124, 1062.

Synthesis of substrate 3j-d₁

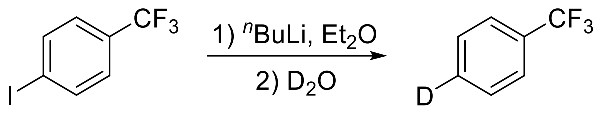

To a 100 ml Schleck tube equipped with a Teflon stir bar, 4-iodobenzotrifluoride (735 μL, 5.0 mmol, 1.0 equiv.) was dissolved in dry Et_2O (20 mL). ^{n}BuLi solution (1.6 M in Et_2O, 3.4 mL, 5.5 mmol, 1.1 equiv.) was added dropwise to the solution at -78 °C. The suspension was stirred at room temperature for 1 hour and the volatiles were removed under vacuum. The solid residue was dissolved in dry Et_2O (20 mL) followed by addition of D_2O (135 μL, 7.5 mmol, 1.5 equiv.) at -78 °C. The resulting solution was distilled at 100 °C to obtain **3j-d$_1$** as a colorless oil (with 35% Et_2O impurity).

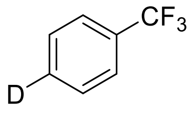

3j-d$_1$: Yield 46%. Colorless oil. R_f = 0.8 (hexane). D%: > 99%.

^{1}H NMR (500 MHz, $CDCl_3$) δ 7.62 (d, J = 6.2 Hz, 2H), 7.48 (d, J = 7.8 Hz, 2H).

^{2}H NMR (61 MHz, $CHCl_3$) δ 7.67 (s, 1D).

^{13}C NMR (101 MHz, $CDCl_3$) δ 131.6 (t, $J_{C\text{-}D}$ = 24.2 Hz), 130.8 (q, $J_{C\text{-}F}$ = 32.3 Hz), 128.7, 125.3 (q, $J_{C\text{-}F}$ = 4.0 Hz), 124.3 (q, $J_{C\text{-}F}$ = 272.7 Hz).

^{19}F NMR (471 MHz, $CDCl_3$) δ -62.8 (s, 3F).

HRMS (ESI): Calcd for $C_7H_4F_3DNa^+$ $[M+Na]^+$: 170.0299, found: 170.0281.

IR (neat, cm^{-1}): ν 3737, 2927, 1322, 1123, 1066, 1021.

3. Optimization of Reaction Conditions

Table S1. Condition optimization of reaction of **1a** with alkyl chlorides

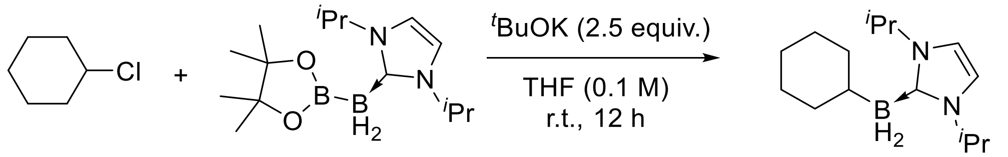

3a, 1.0 equiv. **1a** (2.0 equiv.) **"Standard conditions"** **4a**

entry	variation from "standard conditions"	**4a** [%][b]
1	None	90
2	^{t}BuONa instead of ^{t}BuOK	0
3	^{t}BuOLi instead of ^{t}BuOK	0
4	$KOCH_3$ instead of ^{t}BuOK	trace
5	DBU instead of ^{t}BuOK	0
6	0.20 M instead of 0.10 M	76
7	0.05 M instead of 0.10 M	88
8	1,4-Dioxane instead of THF	0
9	Hexane instead of THF	50
10	1.0 equiv. **1a** instead of 2.0 equiv.	45
11	2.0 equiv. ^{t}BuOK instead of 2.5 equiv.	46
12	60 °C instead of r.t.	56
13	80 °C instead of r.t.	0

[a] Standard reaction conditions: reactions were conducted with with **3a** (0.10 mmol, 1.0 equiv.), **1a** (0.20 mmol, 2.0 equiv.) and ^{t}BuOK (0.25 mmol, 2.5 equiv.) in THF (1 mL) at r.t. under argon for 12 h. [b] Yields were determined by ^{1}H NMR with 1,3,5-trimethoxybenzene as internal standard. DBU = 1,8-diazabicyclo[5.4.0]undec-7-ene, THF = tetrahydrofuran, r.t. = room temperature.

Table S2. Condition optimization of reaction of **1a** with alkyl chlorides

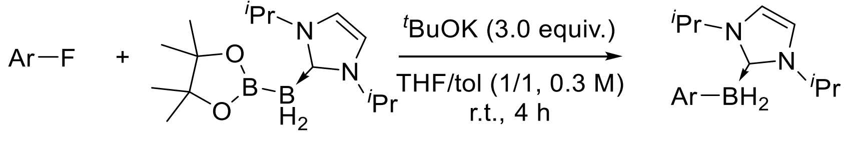

3h, 1.0 equiv. **1a** (2.0 equiv.) **"Standard conditions"** **4h**

entry	variation from "standard conditions"	**4h** [%][b]
1	None	86
2	^{t}BuONa instead of ^{t}BuOK	0
3	^{t}BuOLi instead of ^{t}BuOK	0
4	$KOCH_3$ instead of ^{t}BuOK	trace
5	0.10 M instead of 0.30 M	30
6	1,4-Dioxane instead of THF/tol	52
7	THF/cyclohexane instead of THF/tol	54
8	2-MeTHF/tol instead of THF/tol	52
9	1.0 equiv. **1a** instead of 2.0 equiv.	34
10	3.2 equiv. ^{t}BuOK instead of 3.0 equiv.	65
11	2.0 equiv. ^{t}BuOK instead of 3.0 equiv.	63
12	0 ºC instead of r.t.	0
13	6 h instead of 4 h	83

[a] Standard reaction conditions: reactions were conducted with with **3h** (0.10 mmol, 1.0 equiv.), **1a** (0.20 mmol, 2.0 equiv.) and ^{t}BuOK (0.30 mmol, 3.0 equiv.) in THF/tol (1/1 0.34 mL) at r.t. under argon for 4 h. [b] Yields were determined by ^{1}H NMR with 1,3,5-trimethoxybenzene as internal standard. tol = toluene, THF = tetrahydrofuran, r.t. = room temperature.

4. Nucleophilic Borylation Using 1a and 1c as NHC Boryl Anion Precursors

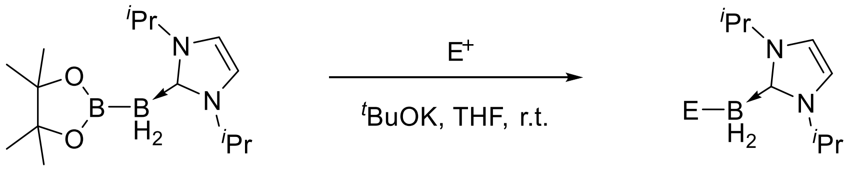

4.1 Reaction of 1a with alkyl chlorides

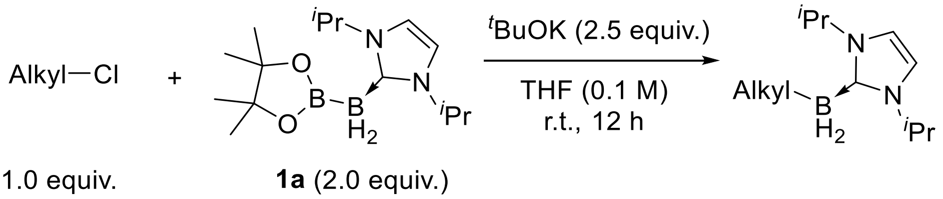

Synthesis of 4a-4e (representative procedure A). In an oven-dried 4 mL vial equipped with a Teflon stir bar, alkyl chloride (0.10 mmol, 1.0 equiv.), diboron reagent **1a** (58.4 mg, 0.20 mmol, 2.0 equiv.) and ^{t}BuOK (28.1 mg, 0.25 mmol, 2.5 equiv.) were added. The mixture was dissolved in THF (1.0 mL) and stirred at room temperature under argon for 12 hours. After completion, the reaction mixture was quenched by exposure to air and diluted with dichloromethane (1 mL). The solvent was then removed under vacuum, and the residue was purified by column chromatography on basic aluminum oxide (hexane/ethyl acetate = 20/1) to give the product.

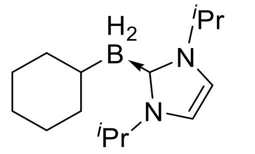

4a: Yield 68%, white solid, m.p. 115 - 117 °C. R_f = 0.6 (hexane/ethyl acetate = 3/1)
^{1}H NMR (500 MHz, $CDCl_3$) δ 6.91 (s, 2H), 5.23 (hept, J = 6.8 Hz, 2H), 1.66 – 1.60 (m, 3H), 1.50 – 1.44 (m, 2H), 1.37 (d, J = 6.8 Hz, 12H), 1.25 (s, 2H), 1.19 – 1.14 (m, 2H), 1.07 – 0.96 (m, 2H).
^{13}C NMR (126 MHz, $CDCl_3$) δ 115.1, 48.9, 36.2, 29.2, 27.7, 23.2.
^{11}B NMR (160 MHz, $CDCl_3$) δ -23.9 (t, J = 83.2 Hz, 1B).
HRMS (ESI): Calcd for $C_{15}H_{29}BN_2Na^+$ $[M+Na]^+$: 271.2316, found: 271.2313.
IR (neat, cm^{-1}): ν 3127, 2981, 2910, 2841, 2257, 1602, 1438, 1207, 1152.

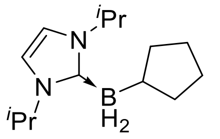

4b: Yield 50%, white solid, m.p. 98 – 100 °C. R_f = 0.6 (hexane/ethyl acetate = 3/1)
^{1}H NMR (500 MHz, $CDCl_3$) δ 6.92 (s, 2H), 5.26 (hept, J = 6.8 Hz, 2H), 1.63 – 1.53 (m, 6H), 1.45 – 1.40 (m, 3H), 1.38 (d, J = 6.7 Hz, 12H).
^{13}C NMR (126 MHz, $CDCl_3$) δ 115.2, 49.0, 35.6, 26.8, 23.4.
^{11}B NMR (160 MHz, $CDCl_3$) δ -24.9 (t, J = 83.4 Hz, 1B).
HRMS (ESI): Calcd for $C_{14}H_{27}BN_2Na^+$ $[M+Na]^+$: 257.2160, found: 257.2155.
IR (neat, cm^{-1}): ν 3126, 2980, 2930, 2850, 2254, 1438, 1214.

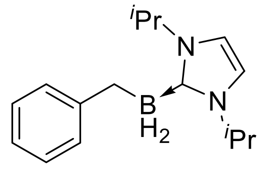

4c: Yield 35%, white solid, m.p. 71 – 73 °C. R_f = 0.6 (hexane/ethyl acetate = 3/1)
^{1}H NMR (400 MHz, $CDCl_3$) δ 7.05 (t, J = 7.6 Hz, 2H), 6.88 (m, 5H), 4.93 (hept, J = 6.8 Hz, 2H), 1.96 (s, 2H), 1.22 (d, J = 6.8 Hz, 12H).
^{13}C NMR (101 MHz, $CDCl_3$) δ 151.9, 127.8, 127.6, 122.3, 115.3, 49.2, 23.1.

^{11}B NMR (128 MHz, $CDCl_3$) δ -25.6 (t, *J* = 86.5 Hz, 1B).
HRMS (ESI): Calcd for $C_{16}H_{25}BN_2Na^+$ $[M+Na]^+$: 279.2003, found: 279.2003.
IR (neat, cm^{-1}): ν 2982, 2820, 2359, 1438, 1209, 1043.

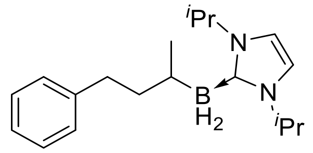

4d: Yield 76%, white solid, m.p. 82 – 84 °C, R_f = 0.6 (hexane/ethyl acetate = 3/1)
^{1}H NMR (400 MHz, $CDCl_3$) δ 7.25 – 7.15 (m, 4H), 7.14 – 7.07 (m, 1H), 6.91 (s, 2H), 5.20 (hept, *J* = 6.9 Hz, 2H), 2.76 (m, 1H), 2.62 (m, 1H), 1.64 – 1.47 (m, 3H), 1.34 (m, 12H), 0.82 (d, *J* = 6.9 Hz, 3H).
^{13}C NMR (101 MHz, $CDCl_3$) δ 145.3, 128.6, 128.0, 124.9, 115.2, 49.0, 42.2, 35.7, 23.4, 23.1, 22.5.
^{11}B NMR (128 MHz, $CDCl_3$) δ -23.9 (t, *J* = 83.5 Hz, 1B).
HRMS (ESI): Calcd for $C_{19}H_{31}BN_2Na^+$ $[M+Na]^+$: 321.2473, found: 321.2468.
IR (neat, cm^{-1}): ν 2926, 2847, 2273, 1437, 1209, 1040.

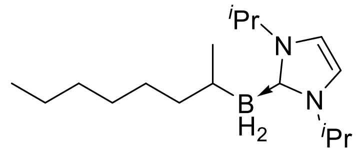

4e: Yield 80%, colorless oil, R_f = 0.6 (hexane/ethyl acetate = 3/1)
^{1}H NMR (400 MHz, $CDCl_3$) δ 6.92 (s, 2H), 5.24 (hept, *J* = 6.9 Hz, 2H), 1.37 (m, 12H), 1.30 – 1.11 (m, 10H), 0.85 (t, *J* = 6.8 Hz, 3H), 0.76 (d, *J* = 6.8 Hz, 3H), 0.64 – 0.48 (m, 1H).
^{13}C NMR (101 MHz, $CDCl_3$) δ 115.2, 49.0, 40.5, 32.4, 30.3, 29.5, 23.4, 23.2, 23.0, 22.5, 14.3.
^{11}B NMR (128 MHz, $CDCl_3$) δ -23.7 (t, *J* = 83.6 Hz, 1B).
HRMS (ESI): Calcd for $C_{17}H_{35}BN_2Na^+$ $[M+Na]^+$: 301.2786, found: 301.2782.
IR (neat, cm^{-1}): ν 2921, 2850, 2269, 1437, 1208, 1041.

4.2 Reaction of 1a with aryl fluoride

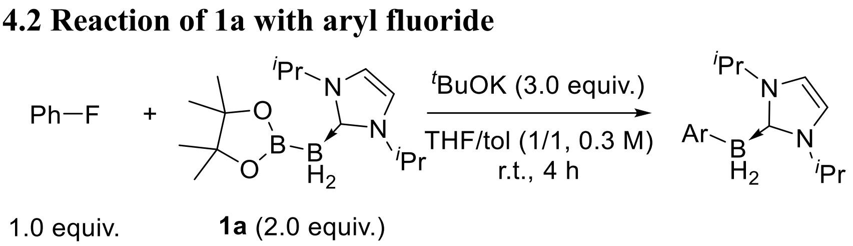

Synthesis of 4f-4i. In an oven-dried 4 mL vial equipped with a Teflon stir bar, aryl fluoride (0.10 mmol, 1.0 equiv.), diboron reagent **1a** (58.4 mg, 0.20 mmol, 2.0 equiv.) and ^{t}BuOK (34.0 mg, 0.30 mmol, 3.0 equiv.) were added. The mixture was dissolved in THF (170 μL) and toluene (170 μL) and stirred at room temperature under argon for 4 hours. After completion, the reaction mixture was quenched by exposure to air and

diluted with dichloromethane (1 mL). The solvent was then removed under vacuum, and the residue was purified by column chromatography on basic aluminum oxide (hexane/dichloromethane = 3/1) to give the product.

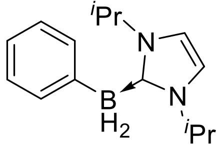

4f: Yield 63%, white solid, m.p. 136 – 138 °C, R_f = 0.6 (hexane/ethyl acetate = 3/1)
^{1}H NMR (500 MHz, $CDCl_3$) δ 7.12 (d, *J* = 6.1 Hz, 4H), 7.00 (d, *J* = 9.2 Hz, 3H), 5.18 (hept, *J* = 6.8 Hz, 2H), 1.34 (d, *J* = 6.7 Hz, 12H).
^{13}C NMR (126 MHz, $CDCl_3$) δ 134.1, 127.2, 123.5, 115.6, 49.5, 23.3.
^{11}B NMR (160 MHz, $CDCl_3$) δ -25.3 (t, *J* = 84.7 Hz, 1B).
HRMS (ESI): Calcd for $C_{15}H_{23}BN_2Na^+$ $[M+Na]^+$: 265.1847, found: 265.1844.
IR (neat, cm^{-1}): ν 2924, 2291, 1434, 1200.

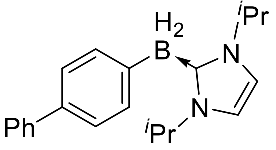

4g: Yield 50%, white solid, m.p. 143 – 145 °C, R_f = 0.6 (hexane/ethyl acetate = 3/1)
^{1}H NMR (400 MHz, $CDCl_3$) δ 7.59 (d, *J* = 7.6 Hz, 2H), 7.39 (t, *J* = 7.7 Hz, 4H), 7.30 – 7.16 (m, 3H), 7.04 (s, 2H), 5.20 (hept, *J* = 6.8 Hz, 2H), 1.37 (d, *J* = 6.8 Hz, 12H).
^{13}C NMR (126 MHz, $CDCl_3$) δ 142.4, 136.3, 134.4, 128.6, 126.9, 126.3, 125.9, 115.7, 49.5, 23.3.
^{11}B NMR (128 MHz, $CDCl_3$) δ -25.4 (t, *J* = 84.8 Hz, 1B).
HRMS (ESI): Calcd for $C_{21}H_{27}BN_2Na^+$ $[M+Na]^+$: 341.2160, found: 341.2153.
IR (neat, cm^{-1}): ν 2980, 2926, 2299, 1438, 1013.

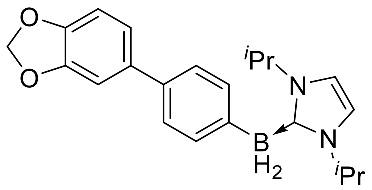

4h: Yield 70%, white solid, m.p. 84 - 86 °C, R_f = 0.4 (hexane/ethyl acetate = 3/1)
^{1}H NMR (500 MHz, $CDCl_3$) δ 7.30 (d, *J* = 7.6 Hz, 2H), 7.17 (d, *J* = 7.8 Hz, 2H), 7.05 (d, *J* = 17.1 Hz, 4H), 6.83 (d, *J* = 8.0 Hz, 1H), 5.96 (s, 2H), 5.19 (hept, *J* = 6.8 Hz, 2H), 1.36 (d, *J* = 6.8 Hz, 12H).
^{13}C NMR (126 MHz, $CDCl_3$) δ 148.0, 146.3, 137.0, 136.0, 134.4, 125.7, 120.2, 115.7, 108.5, 107.6, 101.0, 49.5, 23.3.
^{11}B NMR (160 MHz, $CDCl_3$) δ -25.5 (t, *J* = 85.3 Hz, 1B).
HRMS (ESI): Calcd for $C_{22}H_{27}BN_2O_2Na^+$ $[M+Na]^+$: 385.2058, found: 385.2051.
IR (neat, cm^{-1}): ν 2965, 2924, 2855, 2301, 1438, 1216, 1040.

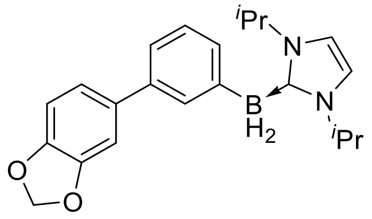

4i: Yield 30%, white solid, m.p. 102 – 104 °C, R$_f$ = 0.4 (hexane/ethyl acetate = 3/1)
^{1}H NMR (400 MHz, $CDCl_3$) δ 7.34 (s, 1H), 7.19 – 7.14 (m, 2H), 7.09 – 7.01 (m, 5H), 6.84 (d, J = 8.0 Hz, 1H), 5.96 (s, 2H), 5.21 (hept, J = 6.8 Hz, 2H), 1.35 (d, J = 6.7 Hz, 12H).
^{13}C NMR (101 MHz, $CDCl_3$) δ 147.9, 146.4, 139.4, 137.7, 132.9, 132.8, 127.5, 122.5, 120.6, 115.7, 108.4, 107.9, 101.0, 49.5, 23.3.
^{11}B NMR (128 MHz, $CDCl_3$) δ -25.2 (t, J = 85.2 Hz, 1B).
HRMS (ESI): Calcd for $C_{22}H_{27}BN_2O_2Na^+$ $[M+Na]^+$: 385.2058, found: 385.2055.
IR (neat, cm^{-1}): ν 2981, 2924, 2856, 2301, 1439, 1217, 1039.

4.3 Reaction of 1a with trifluoromethyl arenes

Synthesis of 4j-4l (representative procedure B). In an oven-dried 4 mL vial equipped with a Teflon stir bar, aromatic trifluoromethyl compound (0.10 mmol, 1.0 equiv.), diboron reagent **1a** (44.0 mg, 0.15 mmol, 1.5 equiv.) and ^{t}BuOK (28.0 mg, 0.25 mmol, 2.5 equiv.) were added. The mixture was dissolved in THF (2.5 mL) and stirred at room temperature under argon for 12 hours. After completion, the reaction mixture was quenched by exposure to air and diluted with dichloromethane (1 mL). The solvent was then removed under vacuum, and the residue was purified by column chromatography on basic aluminum oxide (hexane/ethyl acetate = 10/1) to give the product.

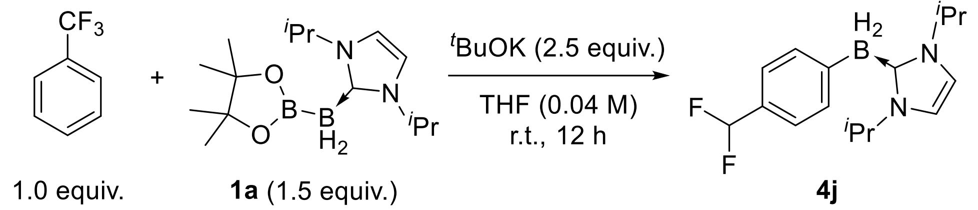

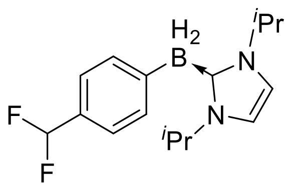

4j: Yield 55%, white solid, m.p. 59 – 61 °C. R$_f$ = 0.4 (hexane/ethyl acetate = 3/1)
^{1}H NMR (500 MHz, $CDCl_3$) δ 7.24 (d, J = 7.6 Hz, 2H), 7.19 (d, J = 7.7 Hz, 2H), 7.03 (s, 2H), 6.56 (t, J = 57.1 Hz, 1H), 5.11 (hept, J = 6.5 Hz, 2H), 1.34 (d, J = 6.8 Hz, 12H).
^{13}C NMR (101 MHz, $CDCl_3$) δ 134.0, 129.6 (t, $J_{C\text{-}F}$ = 22.2 Hz), 124.2 (t, $J_{C\text{-}F}$ = 5.7 Hz), 116.1 (t, $J_{C\text{-}F}$ = 237.4 Hz), 115.8, 49.6, 23.2.

^{11}**B NMR** (160 MHz, $CDCl_3$) δ -25.1 (t, J = 85.6 Hz, 1B).
^{19}**F NMR** (471 MHz, $CDCl_3$) δ -108.6 (d, J = 56.9 Hz, 2F).
HRMS (ESI): Calcd for $C_{16}H_{23}BF_2N_2Na^+$ $[M+Na]^+$: 315.1815, found: 315.1810.
IR (neat, cm^{-1}): ν 3357, 2982, 2920, 2309, 1439, 1372, 1014.

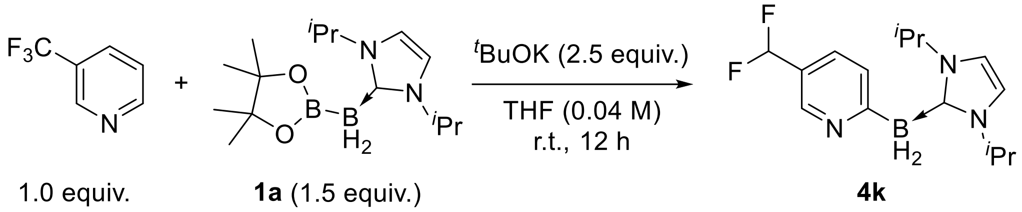

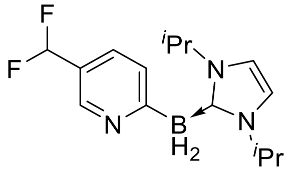

4k: Yield 60%, white solid, m.p. 100 - 102 °C, R_f = 0.5 (ethyl acetate).
^{1}**H NMR** (400 MHz, $CDCl_3$) δ 8.56 (d, J = 2.0 Hz, 1H), 7.47 (m, 1H), 7.34 (d, J = 8.0 Hz, 1H), 7.02 (s, 2H), 6.58 (t, J = 56.4 Hz, 1H), 5.13 (hept, J = 6.8 Hz, 2H), 1.35 (d, J = 6.8 Hz, 12H).
^{13}**C NMR** (126 MHz, $CDCl_3$) δ 146.2, 130.0 (t, J_{C-F} = 5.0 Hz), 128.5, 124.5 (t, J_{C-F} = 22.7 Hz), 115.8, 115.0 (t, J_{C-F} = 238.1 Hz), 49.7, 23.2.
^{11}**B NMR** (128 MHz, $CDCl_3$) δ -25.2 (t, J = 85.1 Hz, 1B).
^{19}**F NMR** (471 MHz, $CDCl_3$) δ -110.1 (d, J = 56.2 Hz, 2F).
HRMS (ESI): Calcd for $C_{15}H_{23}BF_2N_3^+$ $[M+H]^+$: 294.1950, found: 294.1947.
IR (neat, cm^{-1}): ν 3126, 2983, 2929, 2304, 1442, 1399, 1211, 1076, 1034.

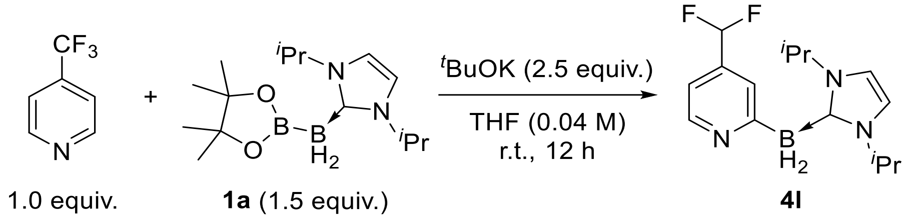

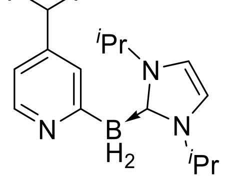

4l: Yield 66%, white solid, m.p. 101 – 103 °C. R_f = 0.5 (ethyl acetate).

^{1}H NMR (500 MHz, $CDCl_3$) δ 8.57 (d, J = 5.1 Hz, 1H), 7.34 (s, 1H), 7.02 (s, 2H), 6.95 (d, J = 5.1 Hz, 1H), 6.51 (t, J = 55.0 Hz, 1H), 5.23 – 5.09 (m, 2H), 1.36 (dd, J = 6.7, 2.2 Hz, 12H).
^{13}C NMR (126 MHz, $CDCl_3$) δ 149.4, 138.9 (t, $J_{C\text{-}F}$ = 22.5 Hz), 124.6, 115.8, 114.5 (t, $J_{C\text{-}F}$ = 239.4 Hz), 114.2 (t, $J_{C\text{-}F}$ = 5.7 Hz), 49.7, 23.2.
^{11}B NMR (128 MHz, $CDCl_3$) δ -25.1 (t, J = 85.3 Hz, 1B).
^{19}F NMR (471 MHz, $CDCl_3$) δ -114.5 (d, J = 56.3 Hz, 2F).
HRMS (ESI): Calcd for $C_{15}H_{23}BF_2N_3^+$ $[M+H]^+$: 294.1948, found: 294.1945.
IR (neat, cm^{-1}): ν 3177, 2983, 2927, 2326, 1442, 1398, 1210, 1074, 1036.

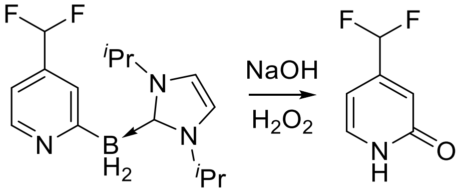

4l **7**, 70%

Synthesis of 7. In a 4 mL vial equipped with a Teflon stir bar, compound **4l** (14.7 mg, 0.05 mmol, 1.0 equiv.) was dissolved in methanol (100 μL) and acetonitrile (100 μL). To this solution, NaOH (8.0 mg, 0.20 mmol, 4.0 equiv.) and water (32 μL) were added, followed by the addition of H_2O_2 (30% *w*/*w* in H_2O, 40 μL). The reaction mixture was stirred overnight and quenched by sat. NH_4Cl solution (1 mL), extracted by dichloromethane (1 mL), dried by anhydrous Na_2SO_4 and purified by column chromatography on silica gel (hexane/ethyl acetate = 5/1) to obtain the product **7**.

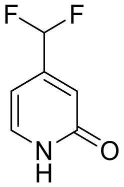

7: Yield 70%, white solid, m.p. 160-162 °C. R_f = 0.5 (ethyl acetate).
^{1}H NMR (400 MHz, $CDCl_3$) δ 7.46 (d, J = 6.7 Hz, 1H), 6.70 (s, 1H), 6.41 (d, J = 5.2 Hz, 1H), 6.46 (t, J = 56.0 Hz, 1H).
^{13}C NMR (101 MHz, $CDCl_3$) δ 173.3, 136.0, 119.1, 118.1 (t, $J_{C\text{-}F}$ = 8.1 Hz), 112.6 (t, $J_{C\text{-}F}$ = 242.4 Hz), 102.7 (t, $J_{C\text{-}F}$ = 5.1 Hz).
^{19}F NMR (377 MHz, $CDCl_3$) δ -118.0 (d, J = 55.1 Hz, 2F).
HRMS (ESI): Calcd for $C_6H_5F_2NONa^+$ $[M+Na]^+$: 168.0231, found: 168.0233.
IR (neat, cm^{-1}): ν 3271, 2921, 2852, 2362, 1668, 1620, 1071.

4.4 Reaction of 1a with cyanoarenes

Synthesis of 4m-4o. In an oven-dried 4 mL vial equipped with a Teflon stir bar, aromatic carbonitrile compound (0.20 mmol, 2.0 equiv.), diboron reagent **1a** (29.0 mg, 0.10 mmol, 1.0 equiv.) and ^{t}BuOK (28.0 mg, 0.25 mmol, 2.5 equiv.) were added. The mixture was dissolved in THF (2.5 mL) and stirred at room temperature under argon

for 4 hours. After completion, the reaction mixture was quenched by exposure to air and diluted with dichloromethane (1 mL). The solvent was then removed under vacuum, and the residue was purified by column chromatography on basic aluminum oxide (hexane/ethyl acetate = 10/1) to give the product.

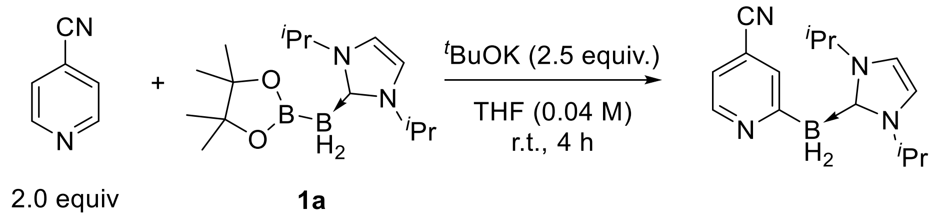

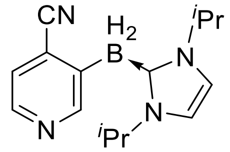

4m: Yield 75%, white solid, m.p. 128 – 130 °C, R_f = 0.5 (ethyl acetate)
^{1}H NMR (400 MHz, $CDCl_3$) δ 8.46 (s, 1H), 8.36 (d, J = 5.0 Hz, 1H), 7.30 (dd, J = 5.1, 1.0 Hz, 1H), 7.07 (s, 2H), 5.01 (hept, J = 6.8 Hz, 2H), 1.35 (d, J = 6.7 Hz, 12H).
^{13}C NMR (126 MHz, $CDCl_3$) δ 156.4, 145.5, 125.5, 124.7, 116.3, 106.6, 50.0, 23.2.
^{11}B NMR (128 MHz, $CDCl_3$) δ -27.2 (t, J = 87.1 Hz, 1B).
HRMS (ESI): Calcd for $C_{15}H_{21}BN_4Na^+$ $[M+Na]^+$: 291.1752, found: 291.1747.
IR (neat, cm^{-1}): ν 2983, 2925, 2331, 1441, 1394, 1212, 1040.

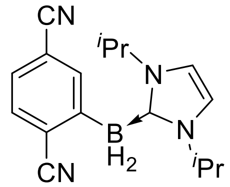

4n: Yield 25%, white solid, m.p. 128 – 130 °C, R_f = 0.7 (dichloromethane)
^{1}H NMR (400 MHz, $CDCl_3$) δ 7.55 (d, J = 7.9 Hz, 1H), 7.42 (s, 1H), 7.35 (d, J = 7.9 Hz, 1H), 7.09 (s, 2H), 4.94 (hept, J = 6.8 Hz, 2H), 1.35 (d, J = 6.8 Hz, 12H).
^{13}C NMR (126 MHz, $CDCl_3$) δ 138.2, 132.6, 127.4, 121.3, 119.7, 119.1, 116.4, 114.4, 50.0, 23.1.
^{11}B NMR (128 MHz, $CDCl_3$) δ -26.1 (t, J = 87.6 Hz, 1B).
HRMS (ESI): Calcd for $C_{17}H_{21}BN_4Na^+$ $[M+Na]^+$: 315.1752, found: 315.1752.
IR (neat, cm^{-1}): ν 2983, 2333, 2225, 1441, 1209, 1041.

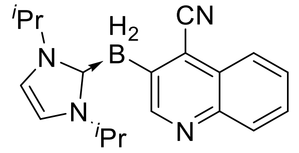

4o: Yield 56%, white solid, m.p. 143 - 145 °C, R_f = 0.6 (hexane/ethyl acetate = 3/1)

^{1}H NMR (500 MHz, $CDCl_3$) δ 8.90 (s, 1H), 8.05 (d, *J* = 8.3 Hz, 2H), 7.68 – 7.61 (m, 1H), 7.58 (t, *J* = 7.6 Hz, 1H), 7.10 (s, 2H), 5.03 (hept, *J* = 6.8 Hz, 2H), 1.37 (d, *J* = 6.8 Hz, 12H).
^{13}C NMR (126 MHz, $CDCl_3$) δ 158.1, 145.5, 129.8, 128.4, 127.7, 127.1, 124.1, 120.4, 117.6, 116.4, 50.0, 23.1.
^{11}B NMR (160 MHz, $CDCl_3$) δ -26.7 (t, *J* = 87.1 Hz, 1B).
HRMS (ESI): Calcd for $C_{19}H_{23}BN_4Na^+$ $[M+Na]^+$: 341.1908, found: 341.1907.
IR (neat, cm^{-1}): ν 2982, 2326, 2221, 1442, 1210, 1040.

4.5 Reaction of 1a with amide

Synthesis of 4p. In an oven-dried 4 mL vial equipped with a Teflon stir bar, *N*-methyl-*N*-phenylpivalamide (19.1 mg, 0.10 mmol, 1.0 equiv.), diboron reagent **1a** (58.4 mg, 0.20 mmol, 2.0 equiv.) and ^{t}BuOK (34.0 mg, 0.30 mmol, 3.0 equiv.) were added. The mixture was dissolved in THF (170 μL) and toluene (170 μL) and stirred at room temperature under argon for 4 hours. After completion, the reaction mixture was quenched by exposure to air and diluted with dichloromethane (1 mL). The solvent was then removed under vacuum, and the residue was purified by column chromatography on basic aluminum oxide (hexane/ethyl acetate = 3/1) to give the product.

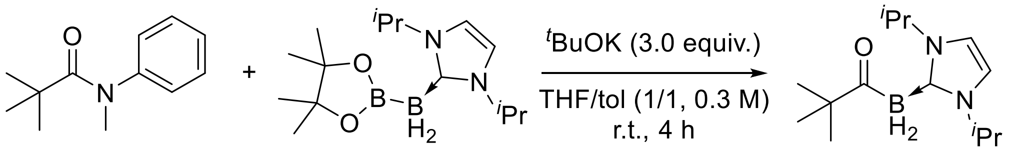

1.0 equiv. **1a** (2.0 equiv.) **4p**

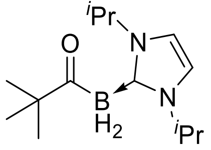

4p: Yield 63%, white solid, m.p. 137 – 139 °C, R_f = 0.4 (hexane/ethyl acetate = 3/1)
^{1}H NMR (400 MHz, $CDCl_3$) δ 6.96 (s, 2H), 4.88 (hept, *J* = 6.8 Hz, 2H), 1.37 (d, *J* = 6.8 Hz, 12H), 1.09 (s, 9H).
^{13}C NMR (126 MHz, $CDCl_3$) δ 115.5, 49.5, 26.8, 23.2.
^{11}B NMR (128 MHz, $CDCl_3$) δ -28.5 (t, *J* = 85.5 Hz, 1B).
HRMS (ESI): Calcd for $C_{14}H_{27}BN_2ONa^+$ $[M+Na]^+$: 273.2109, found: 273.2108.
IR (neat, cm^{-1}): ν 2950, 2335, 1607, 1447, 1218.

4.6 Reaction of 1a with epoxide

Synthesis of 4q. In an oven-dried 4 mL vial equipped with a Teflon stir bar, styrene oxide (12.0 mg, 0.10 mmol, 1.0 equiv.), diboron reagent **1a** (58.4 mg, 0.20 mmol, 2.0 equiv.) and ^{t}BuOK (28.1 mg, 0.25 mmol, 2.5 equiv.) were added. The mixture was dissolved in THF (1.0 mL) and stirred at room temperature under argon for 12 hours.

After completion, the reaction mixture was quenched by methanol (100 μL) and diluted with dichloromethane (1 mL). The solvent was then removed under vacuum, and the residue was purified by column chromatography on basic aluminum oxide (hexane/ethyl acetate = 10/1) to give the product.

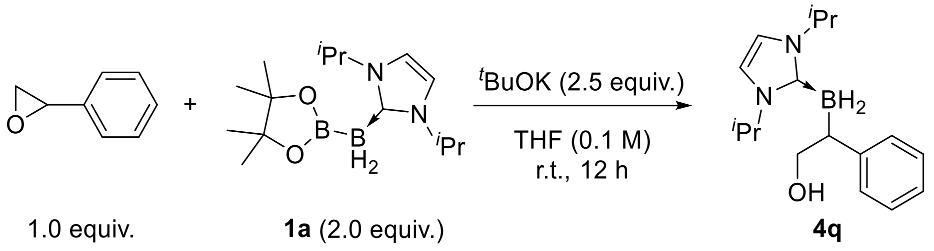

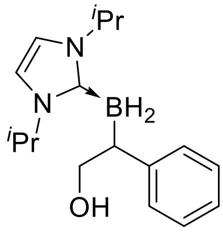

4q: Yield 40%, white solid, m.p. 105 – 107 °C, R_f= 0.6 (hexane/ethyl acetate = 3/1)
^{1}H NMR (400 MHz, $CDCl_3$) δ 7.11 (t, *J* = 7.6 Hz, 2H), 7.02 – 6.90 (m, 3H), 6.85 (s, 2H), 4.84 (hept, *J* = 6.8 Hz, 2H), 4.04 (td, *J* = 9.9, 4.9 Hz, 1H), 3.94 (dt, *J* = 10.6, 6.6 Hz, 1H), 2.21 (br, 1H), 1.78 (dd, *J* = 7.0, 5.0 Hz, 1H), 1.34 (d, *J* = 6.8 Hz, 6H), 1.03 (d, *J* = 6.7 Hz, 6H).
^{13}C NMR (101 MHz, $CDCl_3$) δ 150.4, 128.2, 127.4, 123.7, 115.5, 69.6, 49.3, 23.6, 22.7.
^{11}B NMR (128 MHz, $CDCl_3$) δ -25.4 (t, *J* = 85.7 Hz, 1B).
HRMS (ESI): Calcd for $C_{17}H_{27}BN_2ONa^+$ $[M+Na]^+$: 309.2109, found: 309.2109.
IR (neat, cm^{-1}): ν 2978, 2923, 2859, 2283, 1440, 1209, 1047.

4.7 Reaction of 1c with aryl fluoride

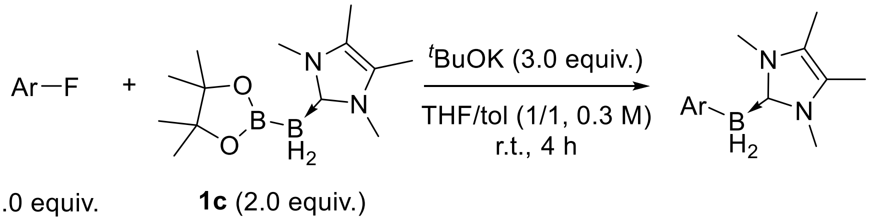

Synthesis of 4r-4v. In an oven-dried 4 mL vial equipped with a Teflon stir bar, aryl fluoride (0.10 mmol, 1.0 equiv.), diboron reagent **1c** (52.8 mg, 0.20 mmol, 2.0 equiv.) and ^{t}BuOK (34.0 mg, 0.30 mmol, 3.0 equiv.) were added. The mixture was dissolved in THF (170 μL) and toluene (170 μL) and stirred at room temperature under argon for 4 hours. After completion, the reaction mixture was quenched by exposure to air and diluted with dichloromethane (1 mL). The solvent was then removed under vacuum,

and the residue was purified by column chromatography on basic aluminum oxide (hexane/dichloromethane = 1/1) to give the product.

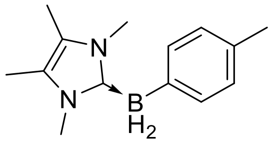

4r: Yield 43%, white solid, m.p. 94 – 96 °C, R$_f$= 0.6 (hexane/ethyl acetate = 1/1)
^{1}H NMR (400 MHz, $CDCl_3$) δ 7.10 (d, *J* = 7.2 Hz, 2H), 6.96 (d, *J* = 7.4 Hz, 2H), 3.66 (s, 6H), 2.26 (s, 3H), 2.13 (s, 6H).
^{13}C NMR (101 MHz, $CDCl_3$) δ 134.3, 132.7, 128.1, 123.5, 32.8, 21.3, 8.9.
^{11}B NMR (128 MHz, $CDCl_3$) δ -25.0 (t, *J* = 84.1 Hz, 1B).
HRMS (ESI): Calcd for $C_{14}H_{21}BN_2Na^+$ $[M+Na]^+$: 251.1690, found: 251.1691.
IR (neat, cm^{-1}): ν 2922, 2859, 2289, 1440, 1050.

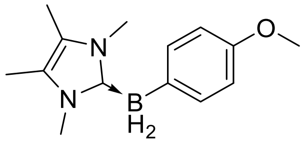

4s: Yield 51%, colorless oil, R$_f$= 0.6 (hexane/ethyl acetate = 1/1)
^{1}H NMR (400 MHz, $CDCl_3$) δ 7.12 (d, *J* = 7.7 Hz, 2H), 6.79 – 6.69 (m, 2H), 3.75 (s, 3H), 3.65 (s, 6H), 2.13 (s, 6H).
^{13}C NMR (101 MHz, $CDCl_3$) δ 156.7, 135.1, 123.5, 113.1, 55.2, 32.8, 9.0.
^{11}B NMR (128 MHz, $CDCl_3$) δ -25.2 (t, *J* = 84.5 Hz, 1B).
HRMS (ESI): Calcd for $C_{14}H_{21}BN_2ONa^+$ $[M+Na]^+$: 267.1639, found: 267.1641.
IR (neat, cm^{-1}): ν 2923, 2286, 1656, 1494, 1269, 1233, 1033.

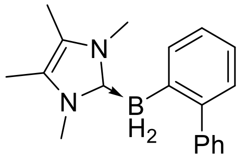

4t: Yield 62%, white solid, m.p. 87 – 89 °C, R$_f$= 0.6 (hexane/ethyl acetate = 1/1)
^{1}H NMR (400 MHz, $CDCl_3$) δ 7.47 (d, *J* = 7.2 Hz, 1H), 7.19 – 7.11 (m, 6H), 7.09 (dd, *J* = 7.3, 1.6 Hz, 1H), 7.03 (dd, *J* = 7.4, 1.6 Hz, 1H), 3.24 (s, 6H), 1.94 (s, 6H).
^{13}C NMR (101 MHz, $CDCl_3$) δ 146.8, 137.3, 136.6, 128.8, 128.5, 126.8, 126.1, 125.0, 123.8, 122.8, 32.29, 8.59.
^{11}B NMR (128 MHz, $CDCl_3$) δ -24.9 (t, *J* = 84.4 Hz, 1B).
HRMS (ESI): Calcd for $C_{19}H_{23}BN_2Na^+$ $[M+Na]^+$: 313.1846, found: 313.1847.
IR (neat, cm^{-1}): ν 3043, 2924, 2330, 1437, 1151, 1039, 1002.

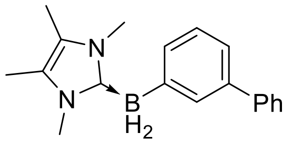

4u: Yield 66%, colorless oil, R_f= 0.6 (hexane/ethyl acetate = 1/1)

^{1}H NMR (500 MHz, $CDCl_3$) δ 7.60 (d, *J* = 8.0 Hz, 2H), 7.49 (s, 1H), 7.44 – 7.36 (m, 2H), 7.31 – 7.24 (m, 2H), 7.24 – 7.20 (m, 2H), 3.68 (s, 6H), 2.13 (s, 6H).

^{13}C NMR (126 MHz, $CDCl_3$) δ 143.2, 139.8, 133.5, 133.4, 128.5, 127.5, 127.4, 126.4, 123.6, 122.8, 32.8, 8.9.

^{11}B NMR (128 MHz, $CDCl_3$) δ -24.7 (t, *J* = 84.4 Hz, 1B).

HRMS (ESI): Calcd for $C_{19}H_{23}BN_2Na^+$ $[M+Na]^+$: 313.1846, found: 313.1847.

IR (neat, cm^{-1}): ν 3036, 2925, 2300, 1468, 1441, 1395, 1071.

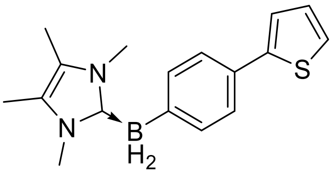

4v: Yield 40%, white solid, m.p. 103 – 105 °C, R_f= 0.6 (hexane/ethyl acetate = 1/1)

^{1}H NMR (400 MHz, $CDCl_3$) δ 7.39 (d, *J* = 8.1 Hz, 2H), 7.22 – 7.19 (m, 3H), 7.16 (d, *J* = 5.2 Hz, 1H), 7.04 – 7.00 (m, 1H), 3.66 (s, 6H), 2.14 (s, 6H).

^{13}C NMR (101 MHz, $CDCl_3$) δ 146.3, 134.7, 129.8, 127.8, 125.0, 123.6, 123.3, 121.6, 32.8, 9.0.

^{11}B NMR (128 MHz, $CDCl_3$) δ -25.0 (t, *J* = 84.1 Hz, 1B).

HRMS (ESI): Calcd for $C_{17}H_{21}BN_2SNa^+$ $[M+Na]^+$: 319.1411, found: 319.1413.

IR (neat, cm^{-1}): ν 3048, 2925, 2298, 1437, 1396, 1050.

4.8 Reaction of 1c with trifluoromethyl arenes

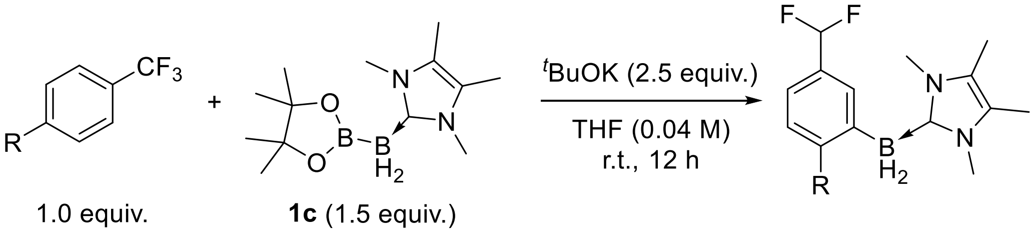

Synthesis of 4w-4z and 4aa. In an oven-dried 4 mL vial equipped with a Teflon stir bar, aromatic trifluoromethyl compound (0.10 mmol, 1.0 equiv.), diboron reagent **1c** (40.0 mg, 0.15 mmol, 1.5 equiv.) and ^{t}BuOK (28.0 mg, 0.25 mmol, 2.5 equiv.) were added. The mixture was dissolved in THF (2.5 mL) and stirred at room temperature under argon for 12 hours. After completion, the reaction mixture was quenched by exposure to air and diluted with dichloromethane (1 mL). The solvent was then removed under vacuum, and the residue was purified by column chromatography on basic aluminum oxide (hexane/ethyl acetate = 5/1) to give the product.

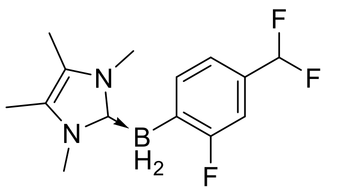

4w: Yield 37%, colorless oil, R_f= 0.7 (hexane/ethyl acetate = 1/1)
^{1}H NMR (500 MHz, $CDCl_3$ δ 7.43 (s, 1H), 7.07 (d, *J* = 7.5 Hz, 1H), 6.92 (d, *J* = 9.2 Hz, 1H), 6.55 (t, *J* = 56.8 Hz, 1H), 3.62 (s, 6H), 2.12 (s, 6H).
^{13}C NMR (126 MHz, $CDCl_3$) δ 166.3 (d, $J_{C\text{-}F}$ = 238.1 Hz), 138.1 (d, $J_{C\text{-}F}$ = 12.6 Hz), 132.3, 123.6, 120.4 (m), 115.0 (t, $J_{C\text{-}F}$ = 235.6 Hz), 110.8 (dt, $J_{C\text{-}F}$ = 29.0, 5.8 Hz), 32.7, 8.9.
^{11}B NMR (160 MHz, $CDCl_3$) δ -27.5 (t, *J* = 86.1 Hz, 1B).
^{19}F NMR (471 MHz, $CDCl_3$) δ -105.4 (s, 1F), -109.2 (d, *J* = 57.3 Hz, 2F).
HRMS (ESI): Calcd for $C_{14}H_{18}BF_3N_2Na^+$ $[M+Na]^+$: 305.1407, found: 305.1408.
IR (neat, cm^{-1}): ν 2923, 2360, 2332, 1408, 1374, 1066, 1016.

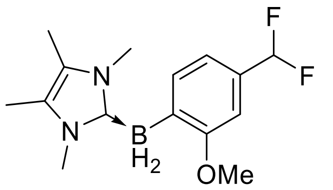

4x: Yield 42%, white solid, m.p. 119 – 121 °C, R_f= 0.7 (hexane/ethyl acetate = 1/1)
^{1}H NMR (400 MHz, $CDCl_3$) δ 7.24 (d, *J* = 8.4 Hz, 1H), 6.90 (d, *J* = 6.1 Hz, 1H), 6.81 (s, 1H), 6.57 (t, *J* = 57.0 Hz, 1H), 3.73 (s, 3H), 3.62 (s, 6H), 2.12 (s, 6H).
^{13}C NMR (126 MHz, $CDCl_3$) δ 162.9, 136.5, 131.7 (t, $J_{C\text{-}F}$ = 22.7 Hz), 123.3, 117.6 (t, $J_{C\text{-}F}$ = 6.3 Hz), 115.9 (t, $J_{C\text{-}F}$ = 236.9 Hz), 105.4 (t, $J_{C\text{-}F}$ = 5.0 Hz), 55.3, 32.7, 8.9.
^{11}B NMR (128 MHz, $CDCl_3$) δ -26.7 (t, *J* = 85.3 Hz, 1B).
^{19}F NMR (471 MHz, $CDCl_3$) δ -108.3 (d, *J* = 57.1 Hz, 2F).
HRMS (ESI): Calcd for $C_{15}H_{21}BF_2N_2ONa^+$ $[M+Na]^+$: 317.1607, found: 317.1608.
IR (neat, cm^{-1}): ν 2926, 2329, 1460, 1406, 1243, 1172, 1067, 1009.

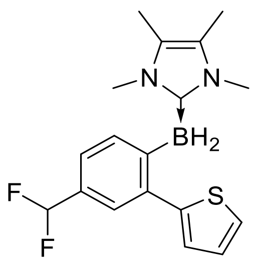

4y: Yield 36%, colorless oil, R_f= 0.7 (hexane/ethyl acetate = 1/1)
^{1}H NMR (400 MHz, $CDCl_3$) δ 7.36 (d, *J* = 7.9 Hz, 1H), 7.24 (m, 2H), 7.15 – 7.14 (m, 1H), 7.11 (m, 1H), 7.06 (m, 1H), 6.59 (t, *J* = 57.0 Hz, 1H), 3.34 (s, 6H), 2.03 (s, 6H).
^{13}C NMR (101 MHz, $CDCl_3$) δ 146.2, 136.2, 130.1 (t, $J_{C\text{-}F}$ = 22.2 Hz), 129.6, 128.8, 127.1, 125.8 (t, $J_{C\text{-}F}$ = 6.1 Hz), 123.3, 123.1, 121.2, 115.9 (t, $J_{C\text{-}F}$ = 238.4 Hz), 32.6, 8.8.
^{11}B NMR (128 MHz, $CDCl_3$) δ -25.0 (t, *J* = 85.2 Hz, 1B).

^{19}F NMR (471 MHz, $CDCl_3$) δ -108.7 (d, *J* = 57.4 Hz, 2F).
HRMS (ESI): Calcd for $C_{18}H_{21}BF_2N_2SNa^+$ $[M+Na]^+$: 369.1379, found: 369.1381.
IR (neat, cm^{-1}): ν 2926, 2332, 1435, 1365, 1071, 1011.

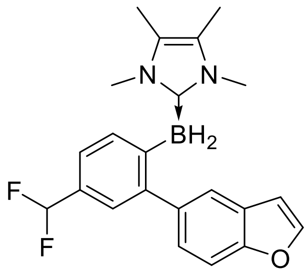

4z: Yield 36%, colorless oil, R_f= 0.7 (hexane/ethyl acetate = 1/1)
^{1}H NMR (500 MHz, $CDCl_3$) δ 7.61 – 7.57 (m, 2H), 7.33 – 7.29 (m, 3H), 7.18 (s, 1H), 7.09 (d, *J* = 8.3 Hz, 1H), 6.68 (s, 1H), 6.61 (t, *J* = 57.0 Hz, 1H), 3.16 (s, 6H), 1.82 (s, 6H).
^{13}C NMR (126 MHz, $CDCl_3$) δ 153.6, 147.0, 145.1, 140.8, 137.0, 130.0 (t, J_{C-F} = 22.7 Hz), 126.5, 125.8 (t, J_{C-F} = 6.3 Hz), 125.5, 123.0 (m), 120.8, 116.0 (t, J_{C-F} = 239.4 Hz), 109.6, 106.6, 32.4, 8.5.
^{11}B NMR (160 MHz, $CDCl_3$) δ -25.0 (t, *J* = 84.9 Hz, 1B).
^{19}F NMR (471 MHz, $CDCl_3$) δ -108.6 (d, *J* = 57.0 Hz, 2F).
HRMS (ESI): Calcd for $C_{22}H_{23}BF_2N_2ONa^+$ $[M+Na]^+$: 403.1764, found: 403.1763.
IR (neat, cm^{-1}): ν 2925, 2360, 1467, 1371, 1198, 1070, 1011.

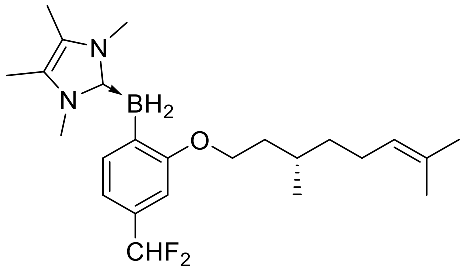

4aa: Yield 37%, colorless oil, R_f= 0.7 (hexane/ethyl acetate = 1/1)
^{1}H NMR (500 MHz, $CDCl_3$) δ 7.40 (d, *J* = 7.2 Hz, 1H), 6.90 (d, *J* = 7.8 Hz, 1H), 6.78 (s, 1H), 6.56 (t, *J* = 57.1 Hz, 1H), 5.11 – 5.05 (m, 1H), 3.88 – 3.79 (m, 2H), 3.57 (s, 6H), 2.11 (s, 6H), 2.02 – 1.97 (m, 1H), 1.94 – 1.88 (m, 1H), 1.68 (s, 3H), 1.59 – 1.53 (m, 3H), 1.53 – 1.47 (m, 1H), 1.44 – 1.26 (m, 3H), 1.18 – 1.12 (m, 1H), 0.89 (d, *J* = 6.6 Hz, 3H).
^{13}C NMR (126 MHz, $CDCl_3$) δ 162.2, 137.3, 131.5 (t, J_{C-F} = 21.4 Hz), 131.5, 124.8, 123.1, 117.5 (t, J_{C-F} = 6.3 Hz), 116.0 (t, J_{C-F} = 236.9 Hz), 106.3 (t, J_{C-F} = 5.0 Hz), 37.4, 36.6, 32.8, 29.7, 25.9, 25.6, 19.8, 17.8, 15.4, 8.9.
^{11}B NMR (160 MHz, $CDCl_3$) δ -26.5 (t, *J* = 85.0 Hz, 1B).
^{19}F NMR (377 MHz, $CDCl_3$) δ -108.2 (d, *J* = 56.6 Hz, 2F).
HRMS (ESI): Calcd for $C_{24}H_{37}BN_2F_2ONa^+$ $[M+Na]^+$: 441.2860, found: 441.2857.
IR (neat, cm^{-1}): ν 2922, 2361, 1378, 1240, 1180,1064, 1012.

4.9 Reaction of 1c with reactive electrophiles

Synthesis of 4ab-4ag. In an argon-charged glove box, an oven-dried flask equipped with a Teflon stir bar was charged with a THF (1.5 mL) solution of diboron reagent **1c** (26.4 mg, 0.1 mmol, 1.0 equiv.) and 18-crown-6 (26.4 mg, 0.1 mmol, 1.0 equiv.). The flask was cooled down to -30°C and ^{t}BuOK (16.8 mg, 0.15 mmol, 1.5 equiv.) was added to the mixture. The mixture was stirred for 40 seconds until the color turned dark red. Then, electrophiles **3** (1.0 mmol, 10.0 equiv.) were added, and the reaction mixture was stirred at room temperature under argon for 30 minutes. After completion, the reaction mixture was diluted with dichloromethane (1 mL). The solvent was then removed under vacuum, and the residue was purified by column chromatography on basic aluminum oxide (hexane/ethyl acetate = 4/1) to give the product.

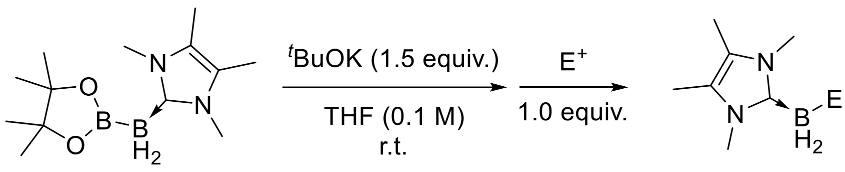

1c (1.0 equiv.) **4**

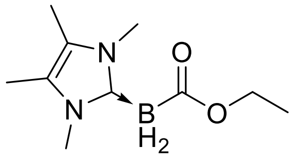

4ab: Yield 73%, white solid, m.p. 109 – 111 °C, R_f= 0.6 (hexane/ethyl acetate = 1/1)
^{1}H NMR (500 MHz, $CDCl_3$) δ 3.99 (q, J = 6.4, 5.8 Hz, 2H), 3.61 (s, 6H), 2.11 (s, 6H), 1.20 (t, J = 7.0 Hz, 3H).
^{13}C NMR (126 MHz, $CDCl_3$) δ 123.9, 56.6, 32.8, 15.0, 8.9.
^{11}B NMR (160 MHz, $CDCl_3$) δ -30.4 (t, J = 86.9 Hz, 1B).
HRMS (ESI): Calcd for $C_{10}H_{19}BN_2O_2Na^+$ $[M+Na]^+$: 233.1432, found: 233.1433.
IR (neat, cm^{-1}): ν 3386, 2927, 2381, 1326, 1646, 1397, 1162, 1042.

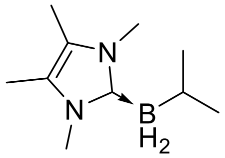

4ac: Yield 41%, colorless oil, R_f= 0.7 (hexane/ethyl acetate = 1/1)
^{1}H NMR (400 MHz, $CDCl_3$) δ 3.64 (s, 6H), 2.11 (s, 6H), 0.82 (br, 6H), 0.72 (br, 1H).
^{13}C NMR (101 MHz, $CDCl_3$) δ 123.0, 32.7, 25.7, 8.9.
^{11}B NMR (128 MHz, $CDCl_3$) δ -22.8 (t, J = 83.1 Hz, 1B).
HRMS (ESI): Calcd for $C_{10}H_{21}BN_2Na^+$ $[M+Na]^+$: 203.1690, found: 203.1693.
IR (neat, cm^{-1}): ν 2922, 2843, 2261, 1658, 1443, 1395, 1173, 1071.

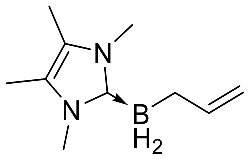

4ad: Yield 15%, colorless oil, R_f= 0.7 (hexane/ethyl acetate = 1/1)
^{1}H NMR (400 MHz, $CDCl_3$) δ 6.03 (dq, *J* = 17.1, 8.4 Hz, 1H), 4.52 (d, *J* = 17.1 Hz, 1H), 4.46 (dd, *J* = 10.0, 3.0 Hz, 1H), 3.64 (s, 6H), 2.10 (s, 6H), 1.36 (br, 2H).
^{13}C NMR (101 MHz, $CDCl_3$) δ 148.0, 123.1, 106.6, 32.6, 8.9.
^{11}B NMR (128 MHz, $CDCl_3$) δ -26.8 (t, *J* = 85.0 Hz, 1B).
HRMS (ESI): Calcd for $C_{10}H_{19}BN_2Na^+$ $[M+Na]^+$: 201.1534, found: 201.1536.
IR (neat, cm^{-1}): ν 3061, 2282, 1620, 1174, 1092.

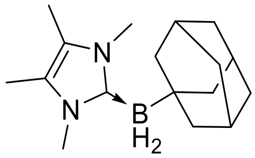

4ae: Yield 32%, white solid, m.p. 115 – 117 °C, R_f= 0.7 (hexane/ethyl acetate = 1/1)
^{1}H NMR (400 MHz, $CDCl_3$) δ 3.66 (s, 6H), 2.12 (s, 6H), 1.75 (br, 3H), 1.66 (br, 6H), 1.55 (m, 6H).
^{13}C NMR (101 MHz, $CDCl_3$) δ 123.3, 46.1, 38.4, 33.5, 29.6, 9.1.
^{11}B NMR (128 MHz, $CDCl_3$) δ -20.6 (t, *J* = 83.3 Hz, 1B).
HRMS (ESI): Calcd for $C_{17}H_{29}BN_2Na^+$ $[M+Na]^+$: 295.2316, found: 295.2318.
IR (neat, cm^{-1}): ν 2886, 2263, 1659, 1440, 1392.

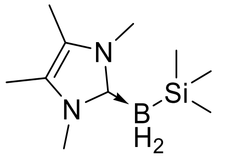

4af: Yield 52%, colorless oil, R_f= 0.6 (hexane/ethyl acetate = 1/1)
^{1}H NMR (400 MHz, $CDCl_3$) δ 3.50 (s, 6H), 2.08 (s, 6H), -0.14 (s, 9H).
^{13}C NMR (101 MHz, $CDCl_3$) δ 122.5, 32.6, 9.0, 2.6.
^{11}B NMR (128 MHz, $CDCl_3$) δ -36.8 (t, *J* = 85.0 Hz, 1B).
HRMS (ESI): Calcd for $C_{10}H_{23}BN_2SiNa^+$ $[M+Na]^+$: 233.1616, found: 233.1617.
IR (neat, cm^{-1}): ν 2938, 2327, 2274, 1660, 1442, 1231, 1111.

Synthesis of 4ag. In an argon-charged glove box, an oven-dried flask equipped with a Teflon stir bar was charged with a THF (1.5 mL) solution of diboron reagent **1c** (26.4 mg, 0.1 mmol, 1.0 equiv.) and 18-crown-6 (26.4 mg, 0.1 mmol, 1.0 equiv.). The flask was cooled down to -30°C and ^{t}BuOK (16.8 mg, 0.15 mmol, 1.5 equiv.) was added to the mixture. The mixture was stirred for 40 seconds until the color turned dark red. Then, benzaldehyde **3ag** (10.6 mg, 0.1 mmol, 1.0 equiv.) were added, and the reaction mixture was stirred at room temperature under argon for 30 minutes. After completion, the reaction mixture was quenched by methanol (100 μL) and diluted with dichloromethane (1 mL). The solvent was then removed under vacuum, and the residue

was purified by column chromatography on basic aluminum oxide (hexane/ethyl acetate = 3/1) to give the product.

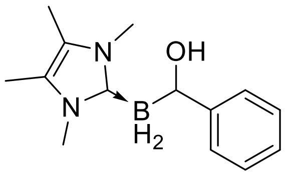

4ag: Yield 16%, colorless oil, R_f = 0.3 (hexane/ethyl acetate = 1/1)
^{1}H NMR (400 MHz, CD_3CN) δ 7.15 – 7.09 (m, 2H), 7.03 – 6.91 (m, 3H), 4.39 (br, 1H), 3.53 (s, 1H), 3.37 (s, 6H), 2.09 (s, 6H).
^{13}C NMR (126 MHz, $CDCl_3$) δ 153.1, 127.6, 124.3, 124.0, 123.5, 32.4, 8.9.
^{11}B NMR (128 MHz, CD_3CN) δ -21.9 (t, J = 87.2 Hz, 1B).
HRMS (ESI): Calcd for $C_{14}H_{21}BN_2ONa^+$ $[M+Na]^+$: 267.1639, found: 267.1644.
IR (neat, cm^{-1}): ν 3398, 2922, 2361, 2331, 1442, 1069, 1000.

5. Control Experiments

5.1 Reaction of B_2pin_2 with electrophiles

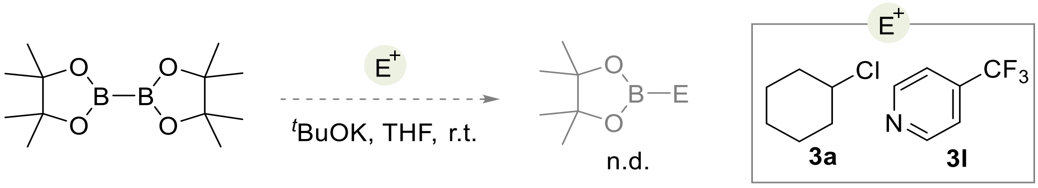

Representative procedure A and **representative procedure B** were followed for the reaction of **3a** and **3l** with B_2pin_2, respectively. Reagent **1a** was replaced by the same equivalent of B_2pin_2. Desired products were not detected by GC-MS.

5.2 Reaction of bulky boryl anion with electrophiles

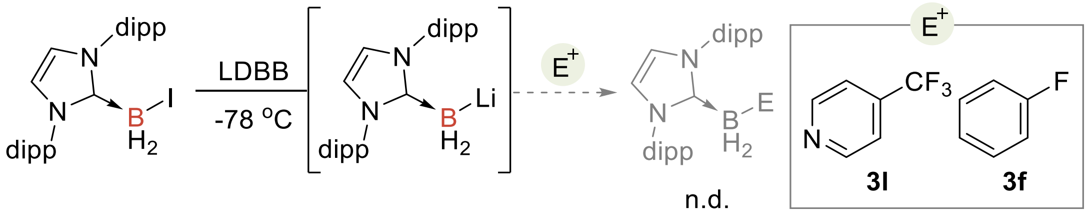

A boryl anion solution was in situ prepared by reductive metalation from 1,3-Bis(2,6-diisopropylphenyl)imidazol-2-ylidene iodoborane according to a literature procedure.[54]
Lithium metal (8.5 mg, 1.25 mmol, 1.0 equiv.) was weighed in an argon-charged glove box. The lithium was cut into small pieces and each piece was scratched to expose a fresh surface. The pieces were added to a 4,4'-di-tert-butylbiphenyl (399.3 mg, 1.5 mmol 1.2 equiv.) solution in THF (7.5 mL) at -30 °C. After the addition, the solution

became dark green. The resulting suspension was sonicated for 1 h at 0 °C. Then, the freshly distilled TMEDA (0.19 mL, 1.25 mmol, 1.0 equiv.) was added to the solution of LDBB dropwise at -30 °C. A solution of LDBB and TMEDA (0.17 M) in THF was obtained.

In the glove box, a 10 mL oven-dried flask equipped with a Teflon stir bar was charged by the LDBB/TMEDA solution (1.78 mL, 4.0 equiv). Then, a solution of 1,3-bis(2,6-diisopropylphenyl)imidazol-2-ylidene iodoborane (40.0 mg, 0.075 mmol, 1.0 equiv.) in dry THF (1 mL) was added dropwise to the LDBB/TMEDA solution at -30 °C. The color remained dark. After 5 min of stirring, the electrophile was added (0.9 mmol, 12.0 equiv.) at -30 °C. A ^{11}B NMR spectrum was recorded after stirring 2 h at room temperature.

The solution was treated with 10.0 equivalents of the literature reported reactive substrate C_6F_6 or unreported substrates **3f** and **3l**. The crude ^{11}B NMR spectra indicated that only C_6F_6 was reactive under this condition (Figure S1).

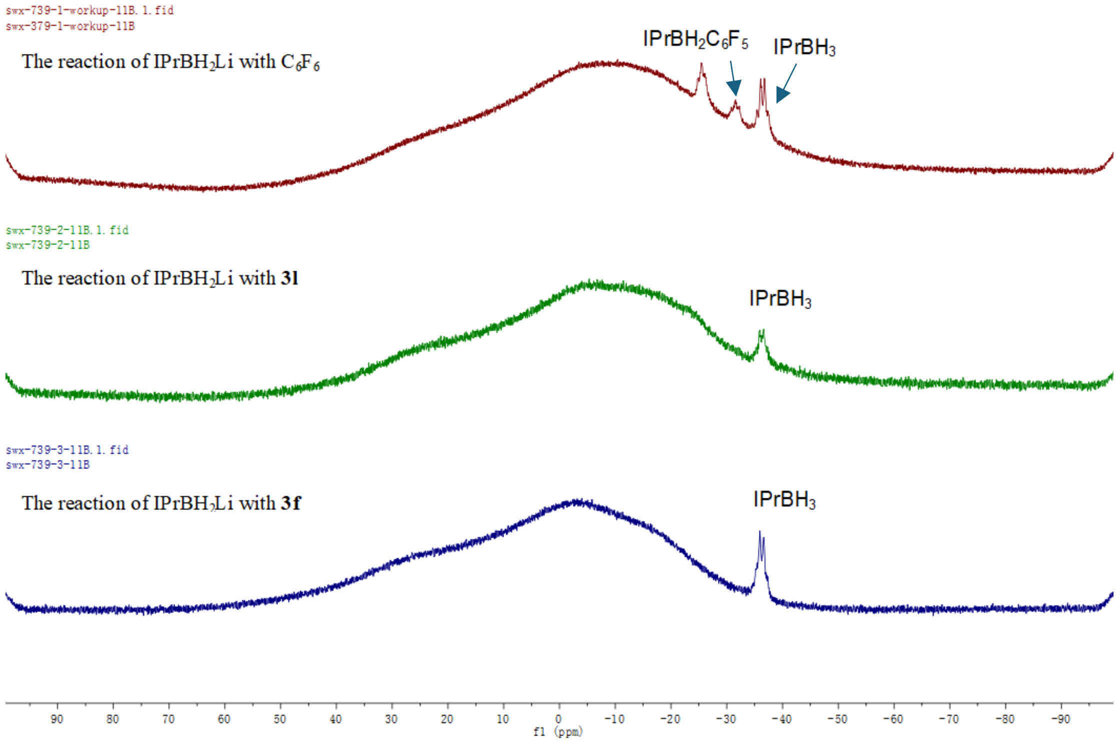

Figure S1. Crude ^{11}B NMR spectra of the reactions of bulky boryl anion $IPrBH_2Li$ ($(IDip)BH_2Li$) with different electrophiles

6. Mechanistic Study

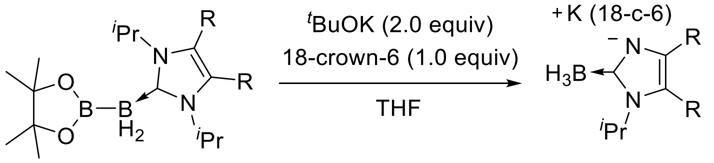

R = H, **1a**
R = Me, **1b**

R = H, **5a**
R = Me, **5b**

Synthesis of compounds 5a and 5b. In an argon charged glove box, an oven-dried 10 mL Schleck tube equipped with a PTFE stir bar was charged with a THF (1.0 mL) solution of 18-crown-6 (26.4 mg, 0.10 mmol, 1.0 equiv.), diboron reagent **1a** or **1b** (0.10 mmol, 1.0 equiv.) and ^{t}BuOK (22.4 mg, 0.20 mmol, 2.0 equiv.). The solution was stirred for 10 minutes then slowly evaporated at room temperature to give potassium salt **5a** or **5b** as colorless crystals.

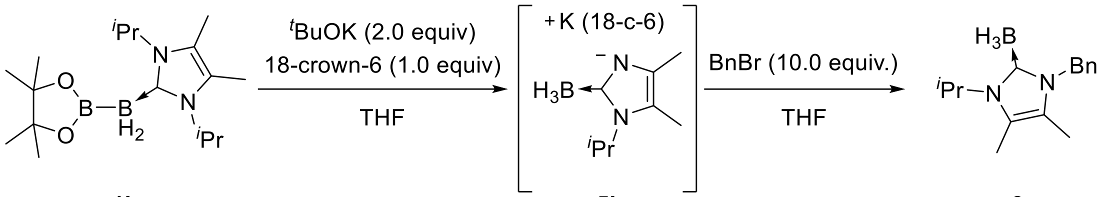

1b **5b** **6**

Synthesis of compound 6. In an argon charged glove box, an oven-dried 10 mL Schleck tube equipped with a PTFE stir bar was charged with a THF (1.0 mL) solution of 18-crown-6 (26.4 mg, 0.10 mmol, 1.0 equiv.), diboron reagent **1b** (32.0 mg, 0.10 mmol, 1.0 equiv.) and ^{t}BuOK (22.4 mg, 0.20 mmol, 2.0 equiv.). The solution was stirred for 10 minutes and was then treated with BnBr (119 μL, 1.0 mmol, 10.0 equiv.). After stirring for an additional 10 minutes, the volatiles were removed under vacuum, and the residue was purified by column chromatography on basic aluminum oxide (hexane/ethyl acetate = 3/1) to obtain product **6**.

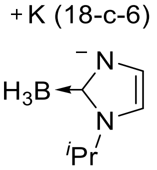

5a: Yield 31%, colorless crystal.

^{1}H NMR (400 MHz, C_6D_6) δ 7.26 (d, J = 1.4 Hz, 1H), 7.02 (d, J = 1.3 Hz, 1H), 5.55 (hept, J = 13.8, 6.8 Hz, 1H), 3.23 (s, 24H), 1.33 (d, J = 6.7 Hz, 6H).

^{13}C NMR (126 MHz, C_6D_6) δ 112.0, 70.2, 47.1, 24.0.

^{11}B NMR (128 MHz, C_6D_6) δ -32.6 (q, J = 82.4 Hz, 1B).

HRMS (ESI): Calcd for $C_6H_{12}BN_2^-$: 123.1094, found: 123.1099. Calcd for $C_{12}H_{24}O_6K^+$: 303.1205, found: 303.1200.

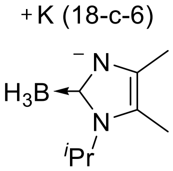

5b: Yield 55%, colorless crystal.

^{1}H NMR (400 MHz, C_6D_6) δ 5.70 (br, 1H), 3.21 (s, 24H), 2.37 (s, 3H), 2.30 (s, 3H), 1.50 (d, *J* = 7.1 Hz, 6H).

^{13}C NMR (126 MHz, C_6D_6) δ 128.2, 128.0, 70.1, 49.6, 25.8, 22.6, 21.2.

^{11}B NMR (128 MHz, C_6D_6) δ -31.7 (q, *J* = 81.4 Hz, 1B).

HRMS (ESI): Calcd for $C_8H_{16}N_2B^-$: 151.1407, found: 151.1411. Calcd for $C_{12}H_{24}O_6K^+$: 303.1205, found: 303.1200.

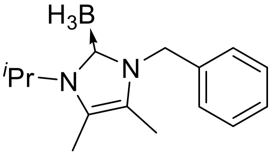

6: Yield 20%, white solid, m.p. 139 – 141 °C, R$_f$ = 0.4 (hexane/ethyl acetate = 1/1)

^{1}H NMR (500 MHz, $CDCl_3$) δ 7.33 – 7.28 (m, 2H), 7.25 (d, *J* = 6.1 Hz, 1H), 7.15 (d, *J* = 7.5 Hz, 2H), 5.38 (s, 3H), 2.22 (s, 3H), 1.93 (s, 3H), 1.51 (d, *J* = 7.1 Hz, 6H).

^{13}C NMR (126 MHz, $CDCl_3$) δ 136.8, 128.8, 127.6, 127.1, 124.6, 122.9, 50.2, 49.4, 21.4, 10.7, 8.8.

^{11}B NMR (160 MHz, $CDCl_3$) δ -36.2 (q, *J* = 86.0 Hz, 1B).

HRMS (ESI): Calcd for $C_{15}H_{23}BN_2Na^+$ $[M+Na]^+$: 265.1847, found: 265.1844.

IR (neat, cm^{-1}): ν 2975, 2927, 2334, 2280, 1408, 1131.

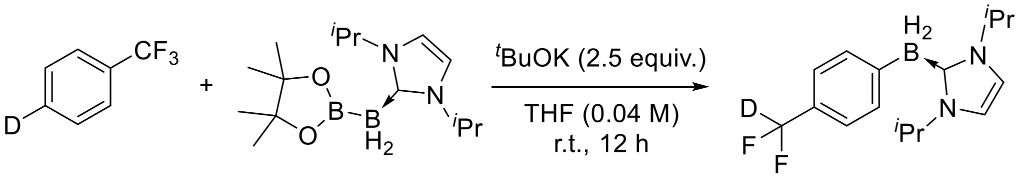

1.0 equiv. **1a** (1.5 equiv.) **4j-d$_1$**

Representative procedure B was followed for the synthesis of **4j-d$_1$**.

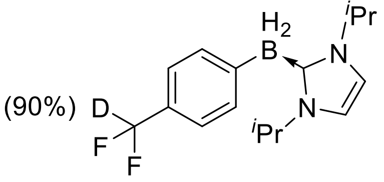

4j-d$_1$: Yield 11%, colorless oil, R$_f$ = 0.4 (hexane/ethyl acetate = 3/1).

^{1}H NMR (400 MHz, $CDCl_3$) δ 7.24 (d, *J* = 7.8 Hz, 2H), 7.19 (d, *J* = 7.7 Hz, 2H), 7.03 (s, 2H), 6.56 (t, *J* = 57.1 Hz, 0.1H), 5.12 (hept, *J* = 6.8 Hz, 2H), 1.34 (d, *J* = 6.7 Hz, 12H).

^{2}H NMR (61 MHz, $CHCl_3$) δ 6.57 (t, *J* = 8.6 Hz, 1D).

^{13}C NMR (126 MHz, $CDCl_3$) δ 134.0, 129.5 (t, $J_{C\text{-}F}$ = 21.4 Hz), 124.2 (t, $J_{C\text{-}F}$ = 5.0 Hz), 116.1 – 115.5 (m), 115.8, 49.6, 23.2.
^{11}B NMR (128 MHz, $CDCl_3$) δ -25.4 (t, J = 85.2 Hz, 1B).
^{19}F NMR (471 MHz, $CDCl_3$) δ -109.8 (t, J = 9.4 Hz, 2F).
HRMS (ESI): Calcd for $C_{16}H_{22}DBF_2N_2Na^+$ $[M+Na]^+$: 316.1878, found: 316.1881.
IR (neat, cm^{-1}): ν 3243, 2981, 2305, 1282, 1208, 1066.

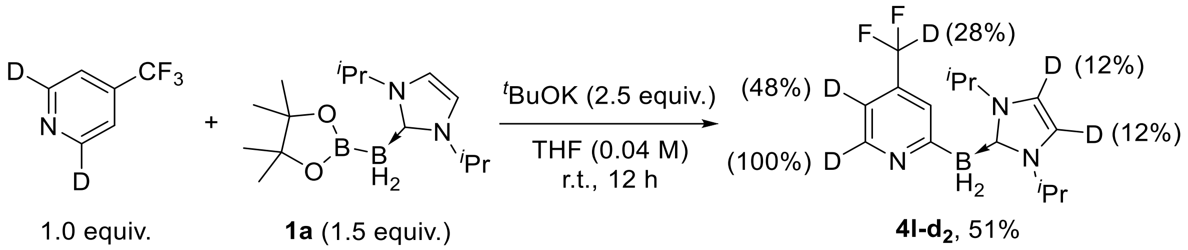

Representative procedure B was followed for the synthesis of **4l-d$_2$**.

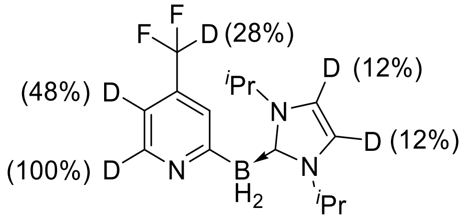

4l-d$_2$: Yield 51%, white solid, R_f = 0.5 (ethyl acetate).
^{1}H NMR (500 MHz, $CDCl_3$) δ 7.34 (s, 1H), 7.02 (s, 1.76H), 6.95 (s, 0.52H), 6.51 (t, J = 56.4 Hz, 0.72H), 5.15 (hept, J = 6.7 Hz, 2H), 1.35 (d, J = 6.8 Hz, 12H).
^{2}H NMR (61 MHz, $CHCl_3$) δ 8.60 (d, J = 2.2 Hz, 1D), 7.30 – 6.74 (m, 0.72H), 6.60 – 6.35 (m, 0.28D).

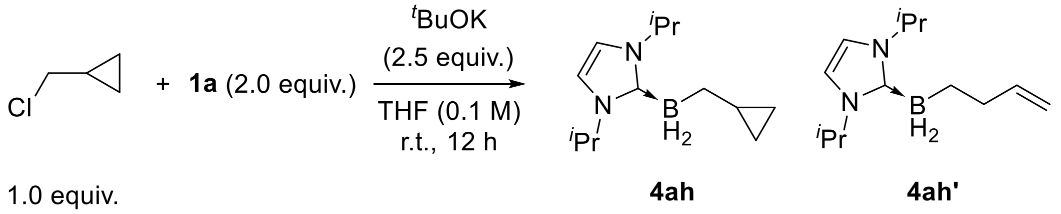

Representative procedure A was followed for the synthesis of **4ah**. Compound **4ah'** was not detected.

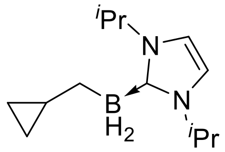

4ah: Yield 60%, white solid, m.p. 47 – 49°C, R_f = 0.6 (hexane/ethyl acetate = 3/1)

^{1}H NMR (500 MHz, $CDCl_3$) δ 6.92 (s, 2H), 5.25 (hept, J = 7.2 Hz, 2H), 1.38 (d, J = 6.8 Hz, 12H), 0.51 (m, 1H), 0.43 – 0.35 (m, 2H), 0.25 (d, J = 7.7 Hz, 2H), -0.18 (d, J = 5.0 Hz, 2H).
^{13}C NMR (126 MHz, $CDCl_3$) δ 115.2, 49.0, 23.3, 13.4, 6.5.
^{11}B NMR (160 MHz, $CDCl_3$) δ -27.8 (t, J = 83.7 Hz, 1B).
HRMS (ESI): Calcd for $C_{13}H_{25}BN_2Na^+$ $[M+Na]^+$: 243.2003, found: 243.2002.
IR (neat, cm^{-1}): ν 2983, 2859, 2275, 1437, 1211.

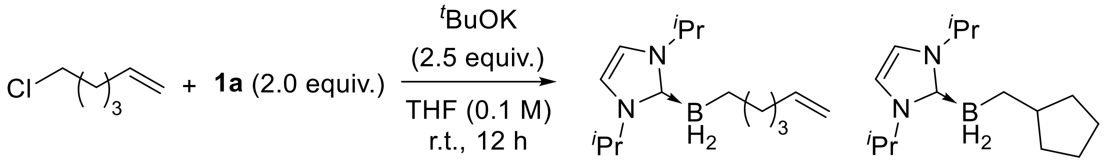

1.0 equiv. **4ai** **4ai'**

Representative procedure A was followed for the synthesis of **4ai**. Compound **4ai'** was not detected.

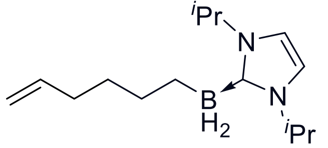

4ai: Yield 34%, colorless oil, R_f = 0.6 (hexane/ethyl acetate = 3/1)
^{1}H NMR (500 MHz, $CDCl_3$) δ 6.91 (s, 2H), 5.83 (ddt, J = 16.9, 10.1, 6.7 Hz, 1H), 5.21 (hept, J = 6.8 Hz, 2H), 4.94 (dd, J = 17.1, 2.0 Hz, 1H), 4.89 – 4.83 (m, 1H), 2.02 (td, J = 8.3, 7.8, 6.0 Hz, 2H), 1.41 – 1.35 (m, 14H), 1.25 – 1.21 (m, 2H), 0.38 (t, J = 7.8 Hz, 2H).
^{13}C NMR (126 MHz, $CDCl_3$) δ 140.5, 115.2, 113.4, 49.0, 34.4, 33.1, 32.4, 23.4.
^{11}B NMR (160 MHz, $CDCl_3$) δ -27.1 (t, J = 83.1 Hz, 1B).
HRMS (ESI): Calcd for $C_{15}H_{29}BN_2Na^+$ $[M+Na]^+$: 271.2316, found: 271.2315.
IR (neat, cm^{-1}): ν 2980, 2914, 2879, 2804, 2268, 1436, 1210.

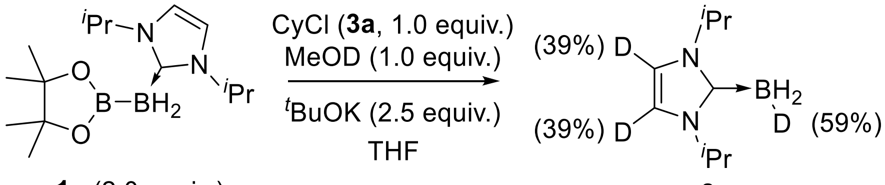

1a (2.0 equiv.) **8**

Synthesis of 8. In an oven-dried 4 mL vial equipped with a Teflon stir bar, cyclohexylchloride (11.9 mg, 0.10 mmol, 1.0 equiv.), diboron reagent **1a** (58.4 mg, 0.20 mmol, 2.0 equiv.), methanol-d_1 (3.4 mg, 0.10 mmol, 1.0 equiv.) and ^{t}BuOK (28.1 mg, 0.25 mmol, 2.5 equiv.) were added. The mixture was dissolved in THF (1.0 mL) and stirred at room temperature under argon for 12 hours. After completion, the reaction mixture was quenched by exposure to air and diluted with dichloromethane (1 mL). The solvent was then removed under vacuum, and the residue was purified by column

chromatography on basic aluminum oxide (hexane/ethyl acetate = 10/1) to give the product.

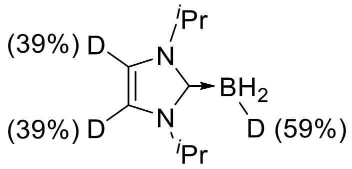

8: Yield 67%, white solid, m.p. 127 – 129 °C, R_f = 0.4 (hexane/ethyl acetate = 3/1).
^{1}H NMR (500 MHz, $CDCl_3$) δ 6.91 (s, 1.2H), 5.11 (hept, *J* = 6.9 Hz, 2H), 1.39 (d, *J* = 6.8 Hz, 12H).
^{1}H{^{11}B} NMR (400 MHz, $CDCl_3$) δ 6.92 (s, 1.2H), 5.12 (hept, *J* = 6.8 Hz, 2H), 1.39 (d, *J* = 6.8 Hz, 12H), 1.06 (s, 1.04H), 1.05 (t, *J* = 2.0 Hz, 0.99H).
^{2}H NMR (61 MHz, $CHCl_3$) δ 6.96 (s), 1.12 – 0.60 (m).
^{13}C NMR (126 MHz, $CDCl_3$) δ 115.1, 49.3, 22.8.
^{11}B NMR (160 MHz, $CDCl_3$) δ -36.4 – -38.3 (m, 1B).
^{11}B{^{1}H} NMR (160 MHz, $CDCl_3$) δ -37.3 – -37.5 (m, 1B).
HRMS (ESI): Calcd for $C_9H_{18}DBN_2Na^+$ $[M+Na]^+$: 190.1597, found: 190.1597.
Calcd for $C_9H_{17}D_2BN_2Na^+$ $[M+Na]^+$: 191.1660, found: 191.1659.
Calcd for $C_9H_{16}D_3BN_2Na^+$ $[M+Na]^+$: 192.1722, found: 192.1725.
IR (neat, cm^{-1}): ν 2928, 2880, 2283, 1640, 1439, 1188.

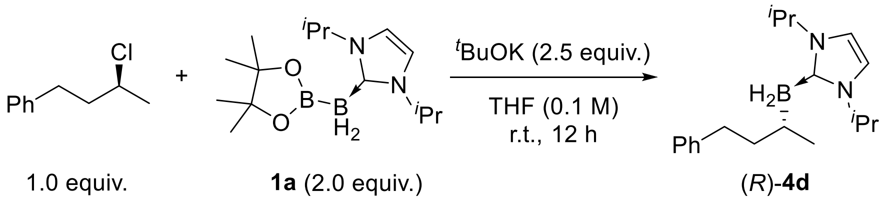

Representative procedure A was followed for the synthesis of (*R*)-**4d**.

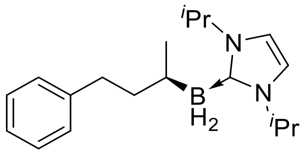

(*R*)-**4d:** Yield 70%. The enantiomeric excess was determined using chiral HPLC (Chiralpak-AD-H, n-hexane/isopropanol = 95/5, flow rate: 1.0 mL/min). $[\alpha]_D^{20}$ = +16.6 (c = 0.90 g/100 mL, $CHCl_3$, 95% *ee*). Other analytic data was identical to the racemic product **4d**.

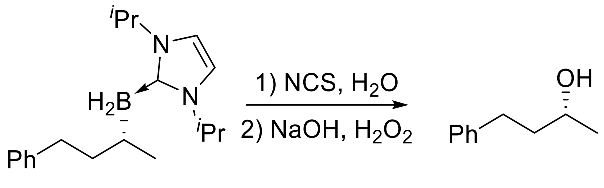

(*R*)-**4d** (*R*)-**3d'**

Oxidation of compound (*R*)-**4d.** To a 4 mL vial equipped with a Teflon stir bar, compound (*R*)-**4d** (29.8 mg, 0.10 mmol, 1.0 equiv.) and *N*-chlorosuccinimide (26.7 mg, 0.20 mmol, 2.0 equiv.) was dissolved in dichloromethane (1 mL). Water (1 mL) was added dropwise to the mixture after stirring for several minutes. The reaction mixture was allowed to stir for 30 minutes followed by addition of H_2O_2 (30% *w*/*w* in H_2O, 100 μL) and NaOH (40.0 mg, 1.0 mmol, 10.0 equiv.). The reaction was quenched by sat. NH_4Cl solution (1 mL), extracted by dichloromethane (1 mL), dried by anhydrous Na_2SO_4 and purified by column chromatography on silica gel (hexane/ethyl acetate = 10/1) to obtain the product (*R*)-**3d'**.

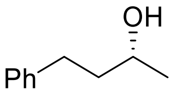

(*R*)-**3d'**: Yield 96%, colorless oil. The enantiomeric excess was determined using chiral HPLC (Chiralcel OJ-3, n-hexane/isopropanol = 98/2, flow rate: 1.0 mL/min). $[\alpha]_D^{20}$ = −15.9 (c = 0. 09 g/100 mL, $CHCl_3$, 94% *ee*). lit. $[\alpha]_D$ = −18.3 ($CHCl_3$).
^{1}H NMR (400 MHz, $CDCl_3$) δ 7.34 – 7.26 (m, 2H), 7.26 – 7.13 (m, 3H), 3.83 (h, *J* = 6.2 Hz, 1H), 2.81 – 2.63 (m, 2H), 1.77 (m, 2H), 1.33 (s, 1H), 1.23 (d, *J* = 6.1 Hz, 3H). The NMR data match with those reported in literature.[92]

^{11}B NMR detection of the possible NHC boryl anion

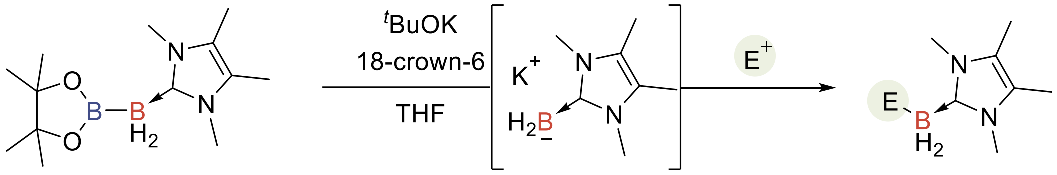

1c ^{11}B NMR δ = –8.8 ppm

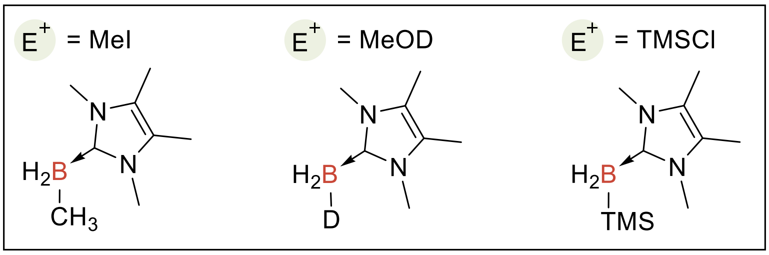

To detect the possible NHC boryl anion by ^{11}B NMR, diboron reagent **1c**, which lacks β-hydrogens for the pericyclic reaction, was reacted with ^{t}BuOK. A triplet at δ = −8.8 ppm appeared in the ^{11}B NMR spectrum. Upon subsequent treatment with electrophiles

such as MeI, MeOD, and TMSCl, signals corresponding to alkyl borane, deuterated borane, and borylsilane were detected by ^{11}B NMR (Figure S2).

^{11}B NMR analysis of the possible NHC boryl anion. In an argon-charged glove box, an oven-dried J-Young NMR tube was charged with a THF (500 μL) solution of diboron reagent **1c** (13.2 mg, 0.05 mmol, 1.0 equiv.) and 18-crown-6 (13.2 mg, 0.05 mmol, 1.0 equiv.). To avoid the quick generation and decomposition of the reactive boryl anion, ^{t}BuOK (8.4 mg, 0.075 mmol, 1.5 equiv.) was first carefully added to the inner wall of the J-Young tube, ensuring it did not come into contact with the diboron solution. After removal from the glove box, the tube was immediately immersed in liquid nitrogen. The solution was then allowed to warm to around -30°C, partially melting, at which point the ^{t}BuOK was quickly mixed with the solution. Within 40 seconds, the solution turned dark red, and it was immediately analyzed by ^{11}B NMR.

Experimental procedure for the capture of the NHC boryl anion by electrophiles. In an argon-charged glove box, an oven-dried flask equipped with a Teflon stir bar was charged with a THF (500 μL) solution of diboron reagent **1c** (13.2 mg, 0.05 mmol, 1.0 equiv.) and 18-crown-6 (13.2 mg, 0.05 mmol, 1.0 equiv.). The flask was cooled down to -30°C and ^{t}BuOK (8.4 mg, 0.075 mmol, 1.5 equiv.) was added to the mixture. The mixture was stirred for 40 seconds until the color turned dark red. Then, MeI (31 μL, 0.50 mmol, 10.0 equiv.), MeOD (20 μL, 0.50 mmol, 10.0 equiv.), or TMSCl (63 μL, 0.50 mmol, 10.0 equiv.) was added, and the crude reaction mixtures were analyzed by ^{11}B NMR to confirm the formation of methyl borane, deuterated borane, or borylsilane, respectively.

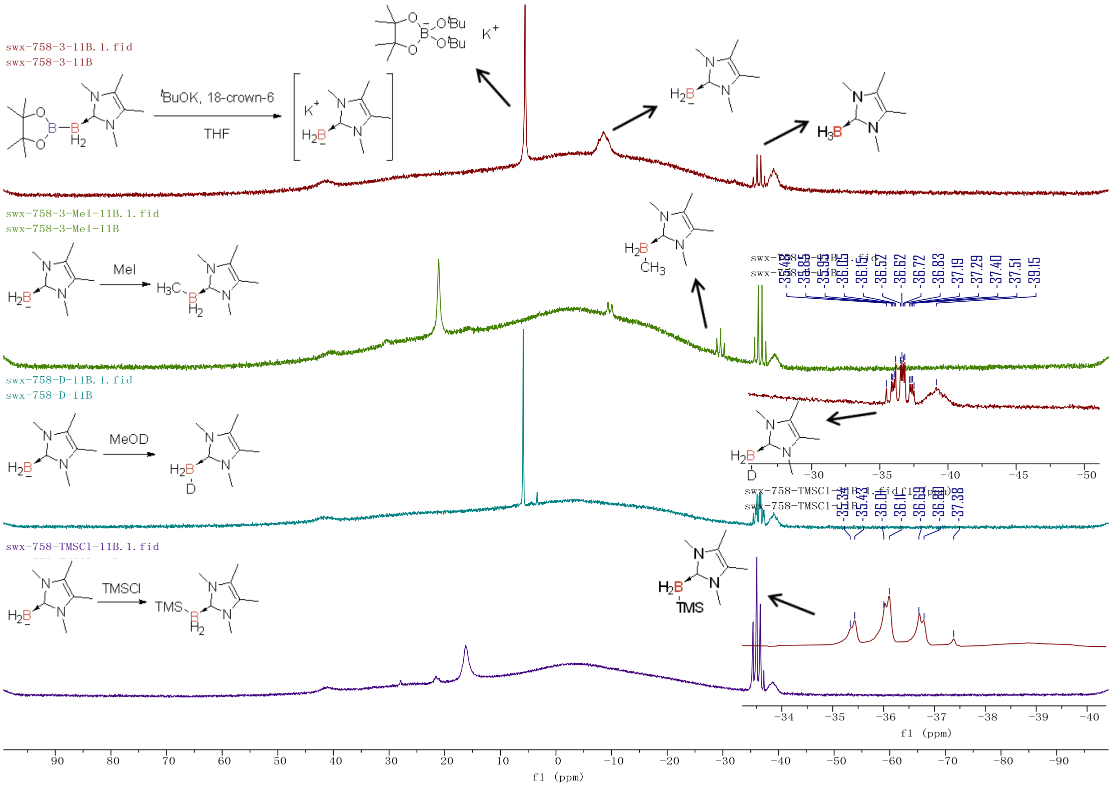

Figure S2. Detection of a possible boryl anion and its reactions with electrophiles.

7. Computational Details

All computation was carried out using the Gaussian 16 software.[93] Structure optimization and frequency analysis was carried out under M06-L / 6-31G(d,p) level. Gibbs free energy was estimated from the frequency analysis result. Explicit THF solvent around the potassium cation was considered, and computation was carried out in gas phase. Molecular orbital graph was drawn using VMD software.[94] Natural fragment bond orbital (NFBO) analysis was carried out using the code from literature.[95] The coordinates were provided in a separate .xyz file.

Due to computational limitations, a methyl variant of the proposed intermediate (^{i}Pr groups in **IM2** are replaced by methyl groups) was used for NFBO analysis. The NHC-B portion was treated as one fragment, while the rest of the molecule was assigned as a separate fragment.

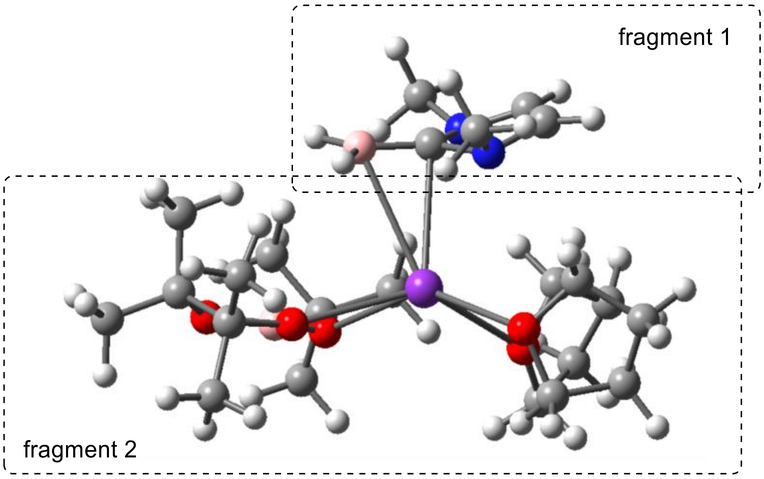

Figure S3. Natural fragment bond orbital (NFBO) analysis

To further verify the nucleophilicity of the boryl anion intermediate **IM2**, the nucleophilicity index was computed by a reported method[96], in comparison with several other boryl anion species.[37,39,54,51] The results revealed that the proposed boryl anion intermediate **IM2** exhibits the highest nucleophilicity among them. To gain additional insight into the effect of anion structure on its nucleophilic ability, the nucleophilicity index of anion fragments were calculated, which confirmed the same conclusion.

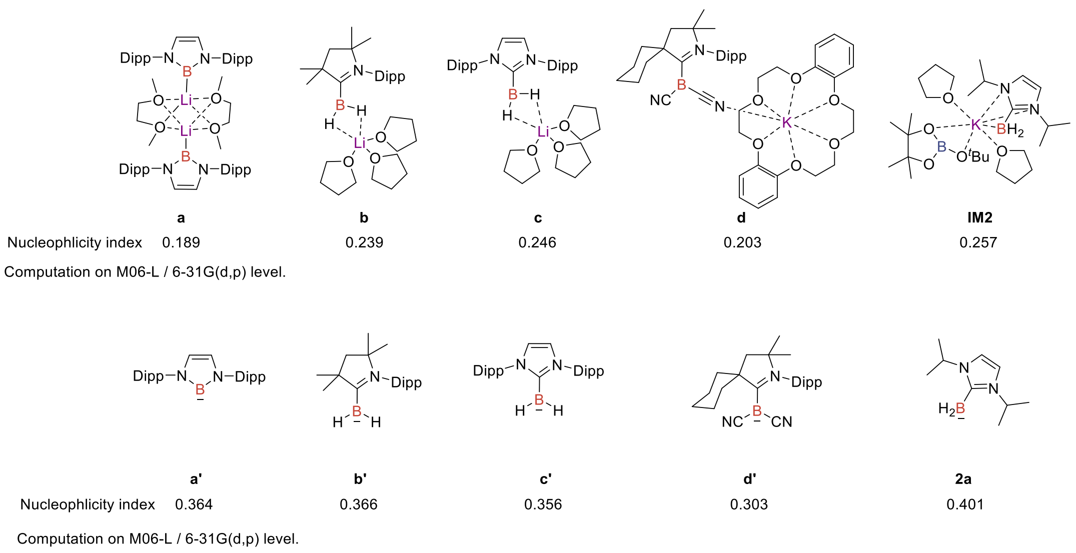

Figure S4. Calculated nucleophilicity index for different boryl anion species

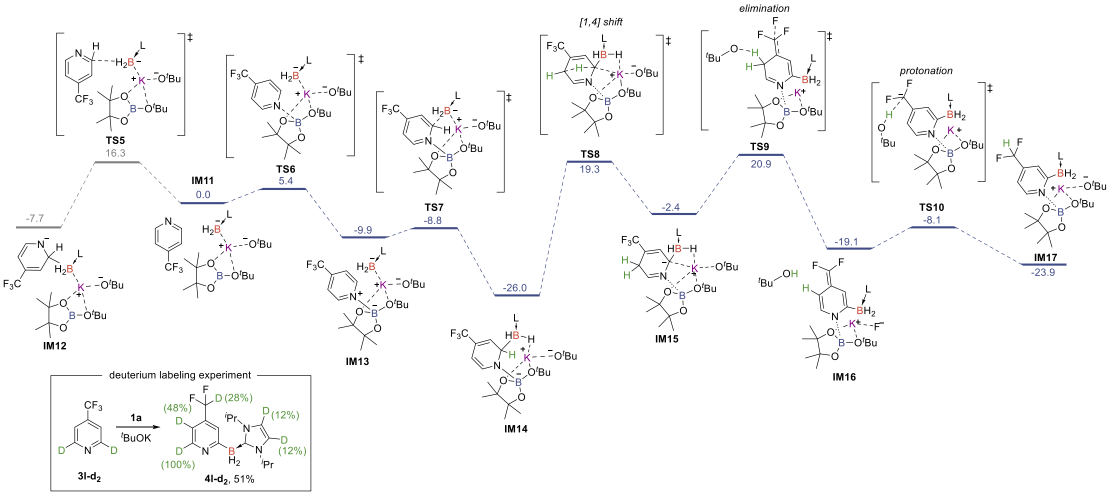

Figure S5. Proposed reaction mechanism for the defluoroborylation of 4-(trifluoromethyl)pyridine and deuterium labelling experiment. Relative free energies are given in kcal/mol.

8. X-ray Data

X-ray Structure Determination. Single-crystal X-ray data of **5b, 4a** and **4b** were collected at 173 K**, 4l** was collected at 297 K**, 4m** was collected at 229 K and **4ah** was collected at 296 K on a Bruker SMART 1000 CCD diffractometer using Mo-Kα radiation. An empirical absorption correction was applied using the SADABS program.[97] All structures were solved by direct methods and subsequent Fourier difference techniques and refined anisotropically for all non-hydrogen atoms by full-matrix least squares calculations on F2 using the SHELXTL program package.[98] All hydrogen atoms were geometrically fixed using the riding model. CCDC 2465888 (**5b**), 2465893 (**4a**), 2465890 (**4b**), 2465847 (**4l**) 2465885 (**4m**) and 2465879 (**4ah**) contain the supplementary crystallographic data for this paper. These data can be obtained free

of charge from the Cambridge Crystallographic Data Centre via www.ccdc.cam.ac.uk/data_request/cif.

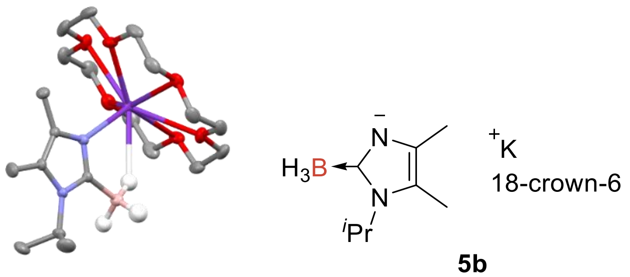

5b

Molecular structure of **5b**, drawn with 50% probability ellipsoids

Crystal data and structure refinement for **5b**.

Identification code	SWX6704(LOW)	
Empirical formula	C20 H38 B K N2 O6	
Formula weight	452.43	
Temperature	173(2) K	
Wavelength	0.71073 Å	
Crystal system	Triclinic	
Space group	P-1	
Unit cell dimensions	a = 9.6736(14) Å	α = 82.794(4)°.
	b = 10.8074(14) Å	β = 72.424(4)°.
	c = 13.5457(15) Å	γ = 69.925(5)°.
Volume	1267.6(3) $Å^3$	
Z	2	
Density (calculated)	1.185 Mg/m^3	
Absorption coefficient	0.244 mm^{-1}	
F(000)	488	
Crystal size	0.500 x 0.400 x 0.200 mm^3	
Theta range for data collection	3.160 to 28.300°.	
Index ranges	-12<=h<=12, -14<=k<=14, -18<=l<=18	
Reflections collected	46421	
Independent reflections	6291 [R(int) = 0.0455]	
Completeness to theta = 25.242°	99.8 %	
Absorption correction	multi-scan	
Max. and min. transmission	0.7457 and 0.6871	
Refinement method	Full-matrix least-squares on F^2	
Data / restraints / parameters	6291 / 0 / 278	

Goodness-of-fit on F^2	1.039
Final R indices [I>2sigma(I)]	R1 = 0.0434, wR2 = 0.1244
R indices (all data)	R1 = 0.0507, wR2 = 0.1317
Extinction coefficient	n/a
Largest diff. peak and hole	0.619 and -0.269 e.Å^{-3}

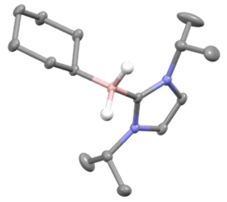

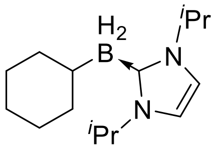

4a

Molecular structure of **4a**, drawn with 30% probability ellipsoids

Crystal data and structure refinement for **4a**.

Identification code	swycyb(low)	
Empirical formula	C15 H29 B N2	
Formula weight	248.21	
Temperature	173(2) K	
Wavelength	0.71073 Å	
Crystal system	Monoclinic	
Space group	$P2_1/c$	
Unit cell dimensions	a = 14.4140(13) Å	α = 90°.
	b = 9.2990(6) Å	β = 92.198(3)°.
	c = 11.9984(10) Å	γ = 90°.
Volume	1607.0(2) Å^3	
Z	4	
Density (calculated)	1.026 Mg/m^3	
Absorption coefficient	0.059 mm^{-1}	
F(000)	552	
Crystal size	0.400 x 0.300 x 0.200 mm^3	
Theta range for data collection	3.578 to 28.310°.	
Index ranges	-19<=h<=17, -12<=k<=12, -16<=l<=13	
Reflections collected	38181	
Independent reflections	3986 [R(int) = 0.0729]	
Completeness to theta = 25.242°	99.7 %	
Absorption correction	multi-scan	
Max. and min. transmission	0.7457 and 0.6647	

Refinement method	Full-matrix least-squares on F^2
Data / restraints / parameters	3986 / 0 / 172
Goodness-of-fit on F^2	1.099
Final R indices [I>2sigma(I)]	R1 = 0.0688, wR2 = 0.1824
R indices (all data)	R1 = 0.0839, wR2 = 0.1950
Extinction coefficient	n/a
Largest diff. peak and hole	0.517 and -0.304 e.Å^{-3}

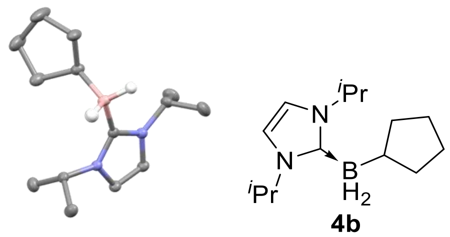

4b

Molecular structure of **4b**, drawn with 30% probability ellipsoids

Crystal data and structure refinement for **4b**.

Identification code	swxcptb(low)	
Empirical formula	C14 H27 B N2	
Formula weight	234.18	
Temperature	173(2) K	
Wavelength	0.71073 Å	
Crystal system	Orthorhombic	
Space group	$Pca2_1$	
Unit cell dimensions	a = 26.117(5) Å	α = 90°.
	b = 11.606(2) Å	β = 90°.
	c = 20.218(3) Å	γ = 90°.
Volume	6128.3(19) Å^3	
Z	16	
Density (calculated)	1.015 Mg/m^3	
Absorption coefficient	0.058 mm^{-1}	
F(000)	2080	
Crystal size	0.500 x 0.400 x 0.300 mm^3	
Theta range for data collection	1.920 to 28.230°.	
Index ranges	-34<=h<=34, -15<=k<=14, -26<=l<=26	
Reflections collected	104766	
Independent reflections	15098 [R(int) = 0.0976]	

Completeness to theta = 25.242°	99.8 %
Absorption correction	Semi-empirical from equivalents
Max. and min. transmission	0.7457 and 0.6755
Refinement method	Full-matrix least-squares on F^2
Data / restraints / parameters	15098 / 1 / 645
Goodness-of-fit on F^2	1.028
Final R indices [I>2sigma(I)]	R1 = 0.0530, wR2 = 0.1123
R indices (all data)	R1 = 0.0906, wR2 = 0.1296
Absolute structure parameter	-0.3(9)
Extinction coefficient	n/a
Largest diff. peak and hole	0.188 and -0.180 e.Å^{-3}

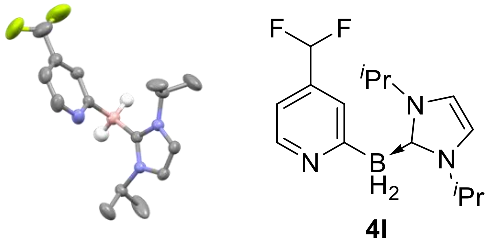

4l

Molecular structure of **4l**, drawn with 30% probability ellipsoids

Crystal data and structure refinement for **4l**.

Identification code	559cf3py	
Empirical formula	C15 H22 B F2 N3	
Formula weight	293.16	
Temperature	297(2) K	
Wavelength	0.71073 Å	
Crystal system	Monoclinic	
Space group	$P2_1/n$	
Unit cell dimensions	a = 7.5933(4) Å	α = 90°.
	b = 25.4187(13) Å	β = 90.638(2)°.
	c = 8.7090(5) Å	γ = 90°.
Volume	1680.84(16) Å^3	
Z	4	
Density (calculated)	1.158 Mg/m^3	
Absorption coefficient	0.084 mm^{-1}	
F(000)	624	
Crystal size	0.500 x 0.400 x 0.300 mm^3	

Theta range for data collection	3.355 to 28.367°.
Index ranges	-10<=h<=10, -33<=k<=33, -11<=l<=11
Reflections collected	38147
Independent reflections	4184 [R(int) = 0.0434]
Completeness to theta = 25.242°	99.8 %
Absorption correction	multi-scan
Max. and min. transmission	0.7457 and 0.7036
Refinement method	Full-matrix least-squares on F^2
Data / restraints / parameters	4184 / 24 / 217
Goodness-of-fit on F^2	1.033
Final R indices [I>2sigma(I)]	R1 = 0.0672, wR2 = 0.2120
R indices (all data)	R1 = 0.0770, wR2 = 0.2237
Extinction coefficient	n/a
Largest diff. peak and hole	0.302 and -0.262 e.$Å^{-3}$

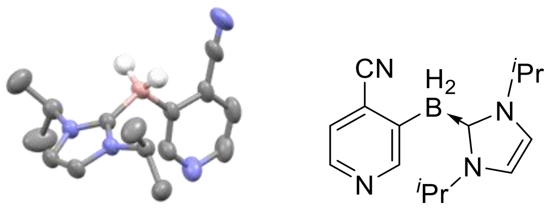

4m

Molecular structure of **4m**, drawn with 30% probability ellipsoids

Crystal data and structure refinement for **4m**.

Identification code	SWX606CNPY	
Empirical formula	C15 H21 B N4	
Formula weight	268.17	
Temperature	229(2) K	
Wavelength	0.71073 Å	
Crystal system	Monoclinic	
Space group	$P2_1/n$	
Unit cell dimensions	a = 11.1896(3) Å	α = 90°.
	b = 8.6273(3) Å	β = 91.3370(10)°.
	c = 16.8978(5) Å	γ = 90°.
Volume	1630.80(9) $Å^3$	
Z	4	
Density (calculated)	1.092 Mg/m^3	

Absorption coefficient	0.066 mm^{-1}
F(000)	576
Crystal size	0.500 x 0.400 x 0.300 mm^3
Theta range for data collection	2.207 to 28.298°.
Index ranges	-14<=h<=14, -11<=k<=11, -22<=l<=22
Reflections collected	43114
Independent reflections	4051 [R(int) = 0.0661]
Completeness to theta = 25.242°	99.7 %
Absorption correction	multi-scan
Max. and min. transmission	0.7457 and 0.7013
Refinement method	Full-matrix least-squares on F^2
Data / restraints / parameters	4051 / 0 / 190
Goodness-of-fit on F^2	1.042
Final R indices [I>2sigma(I)]	R1 = 0.0522, wR2 = 0.1464
R indices (all data)	R1 = 0.0884, wR2 = 0.1716
Extinction coefficient	0.010(3)
Largest diff. peak and hole	0.203 and -0.137 e.Å^{-3}

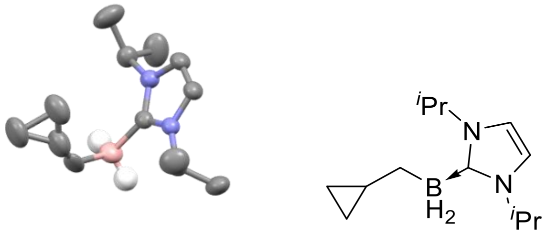

4ah

Molecular structure of **4ah**, drawn with 30% probability ellipsoids

Crystal data and structure refinement for **4ah**.

Identification code	swx591cpv	
Empirical formula	C13 H25 B N2	
Formula weight	220.16	
Temperature	296(2) K	
Wavelength	0.71073 Å	
Crystal system	Monoclinic	
Space group	$P2_1/c$	
Unit cell dimensions	a = 10.3387(5) Å	α = 90°.
	b = 25.0388(11) Å	β = 103.3610(10)°.

	c = 11.9005(4) Å γ = 90°.
Volume	2997.3(2) $Å^3$
Z	8
Density (calculated)	0.976 Mg/m^3
Absorption coefficient	0.056 mm^{-1}
F(000)	976
Crystal size	0.500 x 0.400 x 0.300 mm^3
Theta range for data collection	2.396 to 25.248°.
Index ranges	-12<=h<=12, -30<=k<=30, -14<=l<=13
Reflections collected	50777
Independent reflections	5431 [R(int) = 0.0533]
Completeness to theta = 25.242°	99.8 %
Absorption correction	multi-scan
Max. and min. transmission	0.7456 and 0.6706
Refinement method	Full-matrix least-squares on F^2
Data / restraints / parameters	5431 / 274 / 372
Goodness-of-fit on F^2	1.043
Final R indices [I>2sigma(I)]	R1 = 0.0692, wR2 = 0.1956
R indices (all data)	R1 = 0.0907, wR2 = 0.2147
Extinction coefficient	0.009(2)
Largest diff. peak and hole	0.333 and -0.232 e.$Å^{-3}$

Notes: An Alert B pertains to the observed low-density value (p = 0.976 < 1), which may be attributed either to the intrinsic characteristics of the sample or to the presence of diethyl ether molecules, employed during crystal growth, within the unit cell. These solvent molecules could have volatilized during data collection at room temperature due to their low boiling point, resulting in a reduced calculated density.

9. NMR Spectra

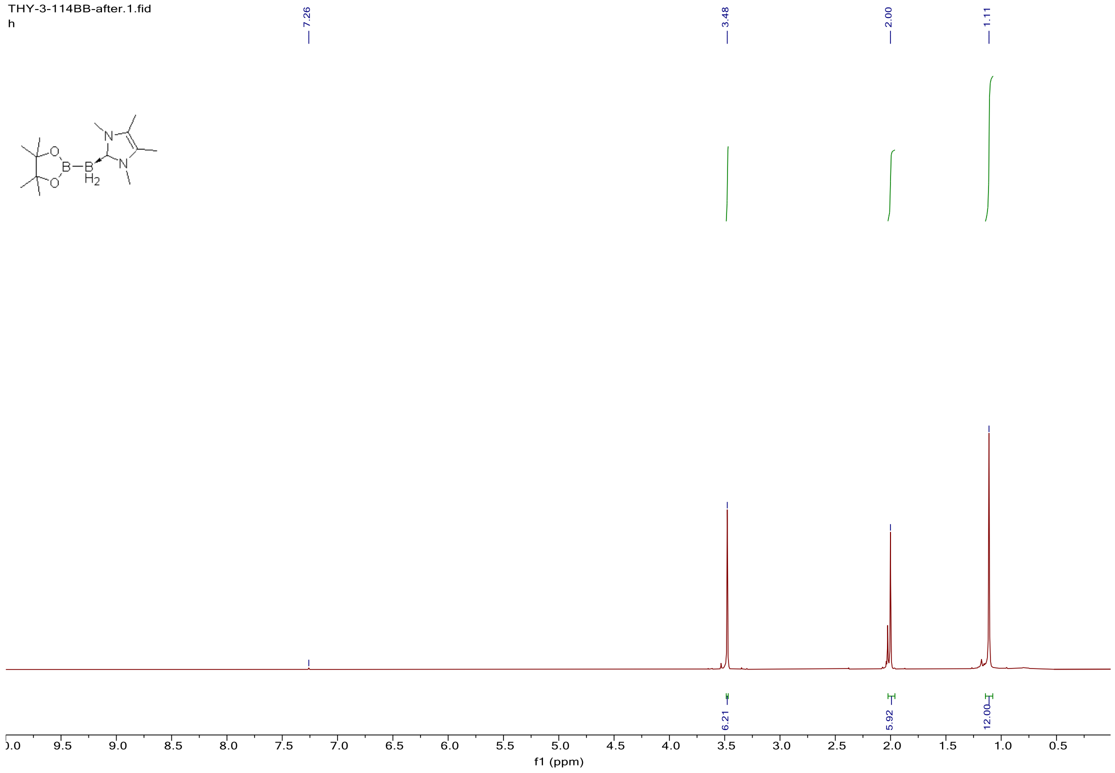

^{1}H NMR spectrum of **1c** in $CDCl_3$, 400 MHz.

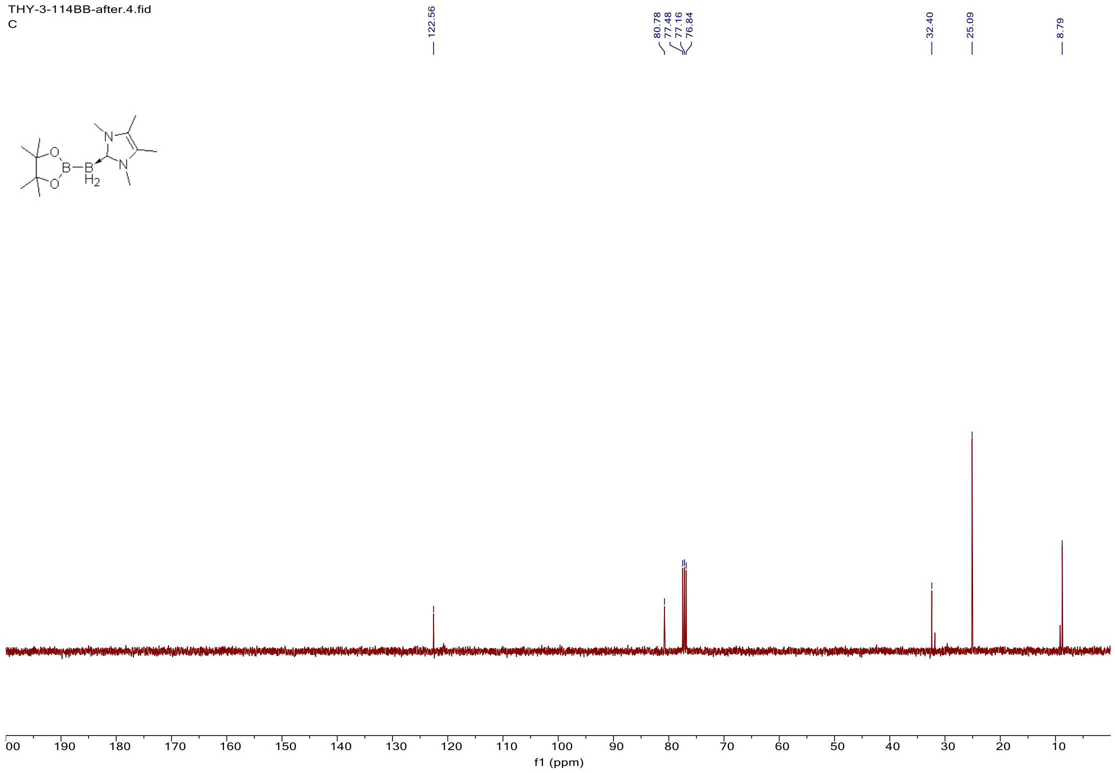

^{13}C NMR spectrum of **1c** in $CDCl_3$, 101 MHz.

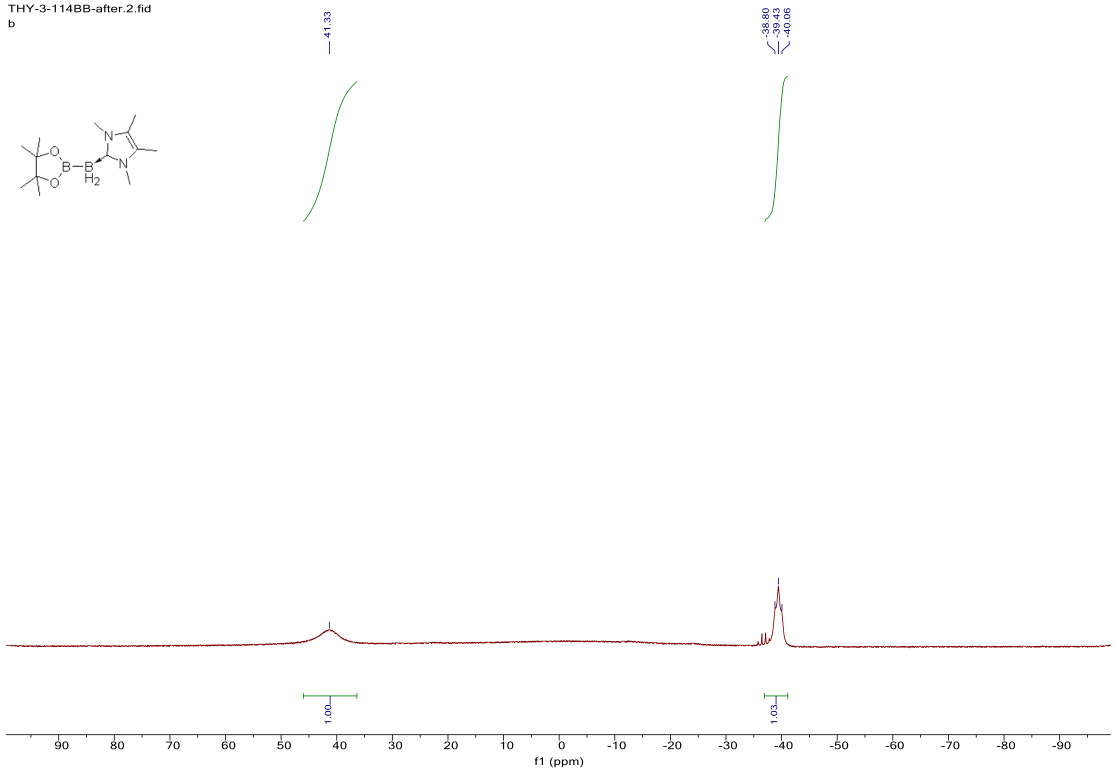

^{11}B NMR spectrum of **1c** in $CDCl_3$, 128 MHz.

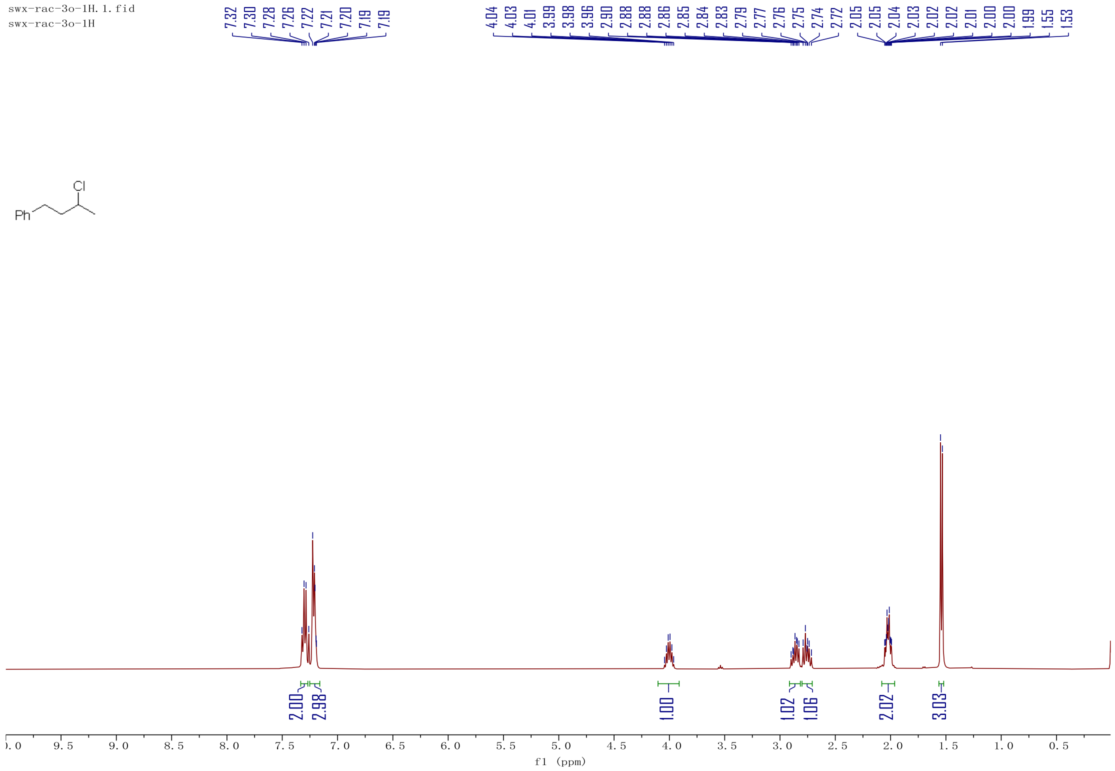

^{1}H NMR spectrum of **3d** in $CDCl_3$, 400 MHz.

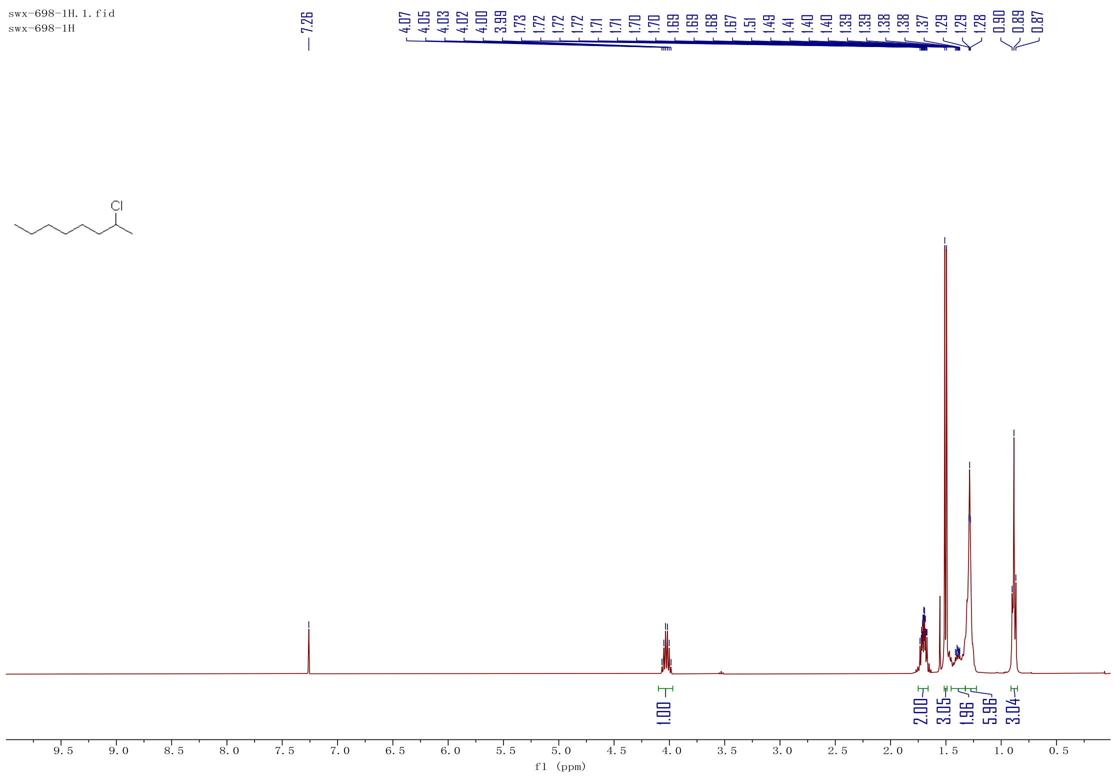

^{1}H NMR spectrum of **3e** in $CDCl_3$, 400 MHz.

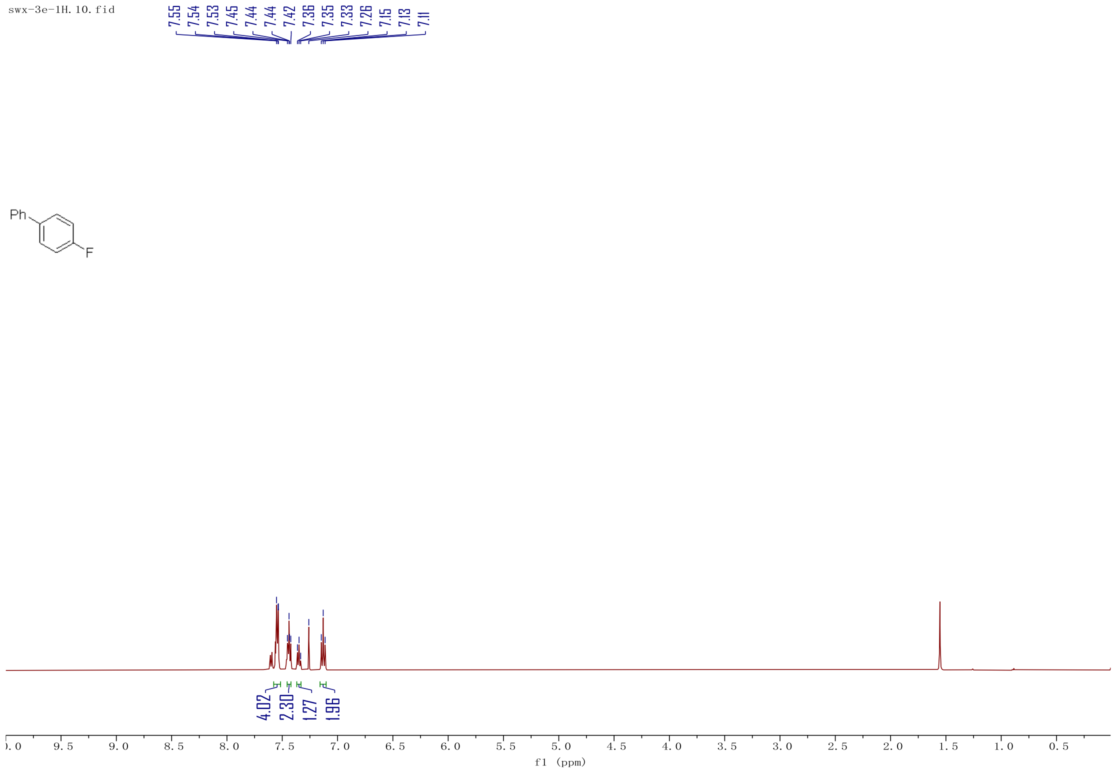

^{1}H NMR spectrum of **3g** in $CDCl_3$, 500 MHz.

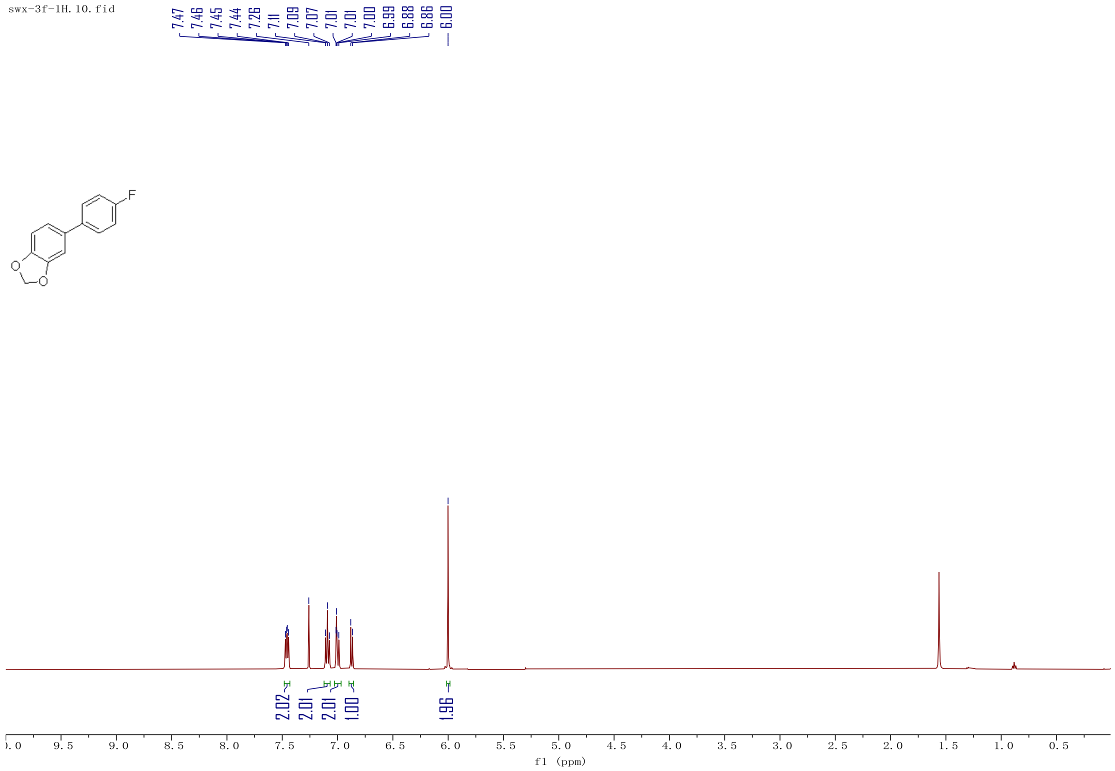

^{1}H NMR spectrum of **3h** in $CDCl_3$, 500 MHz.

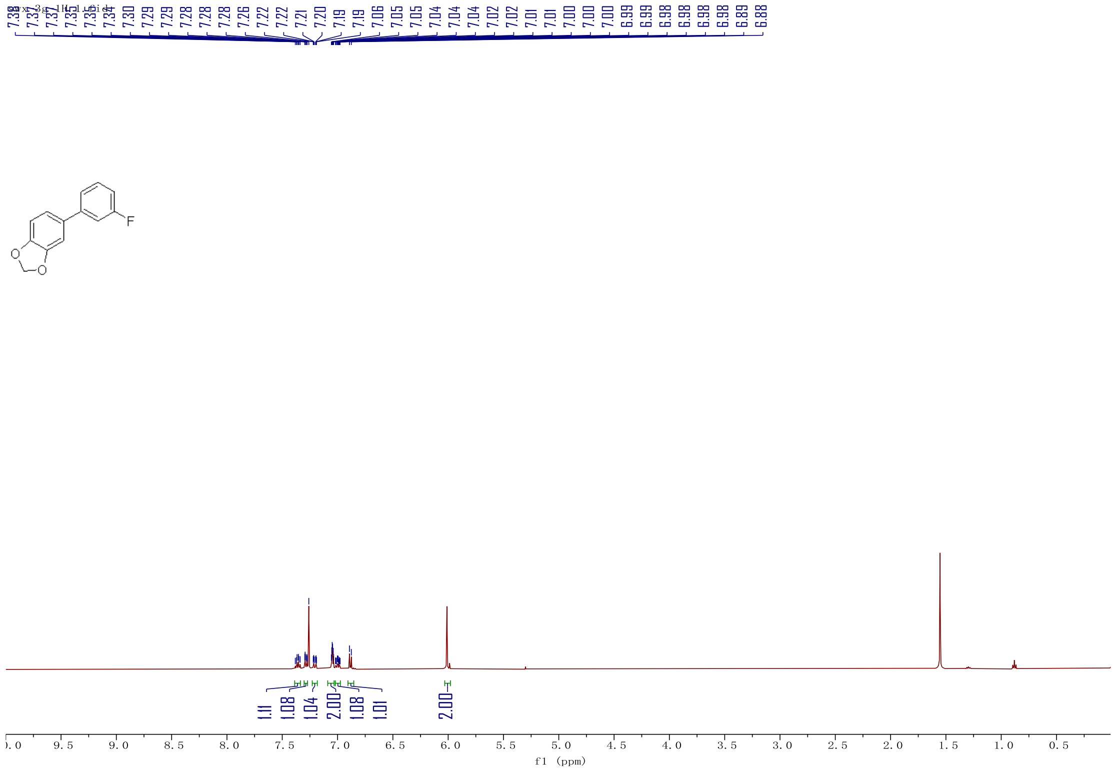

^{1}H NMR spectrum of **3i** in $CDCl_3$, 500 MHz.

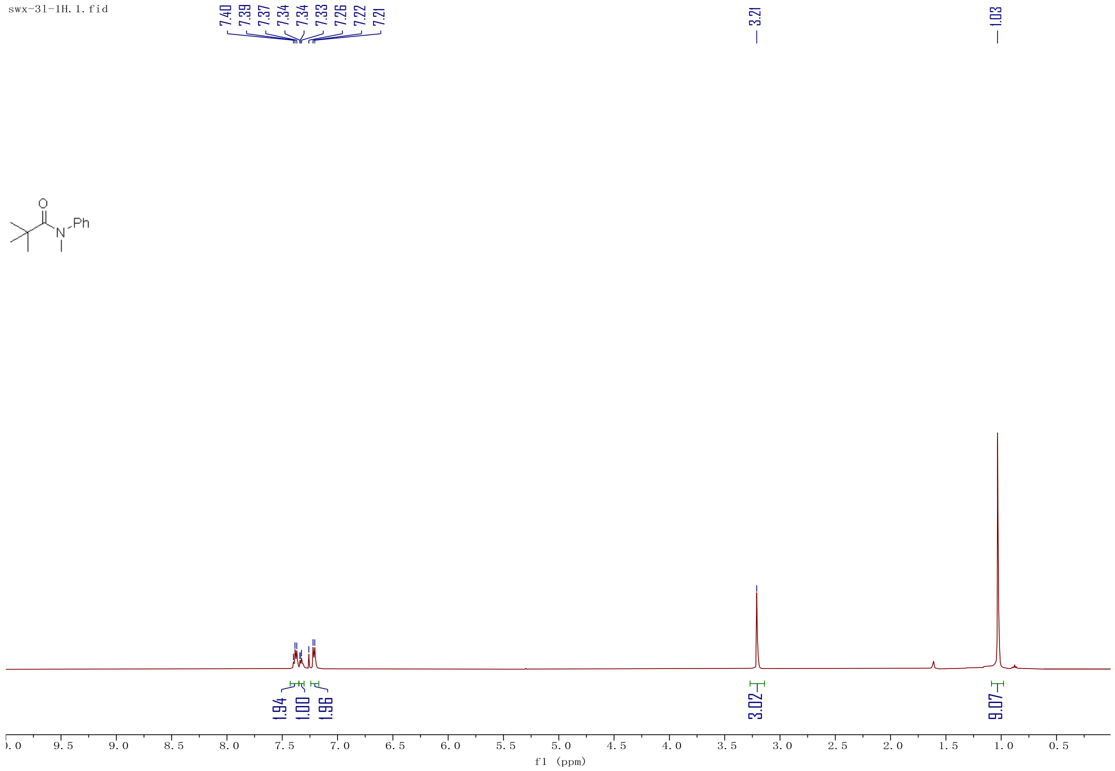

^{1}H NMR spectrum of **3p** in $CDCl_3$, 500 MHz.

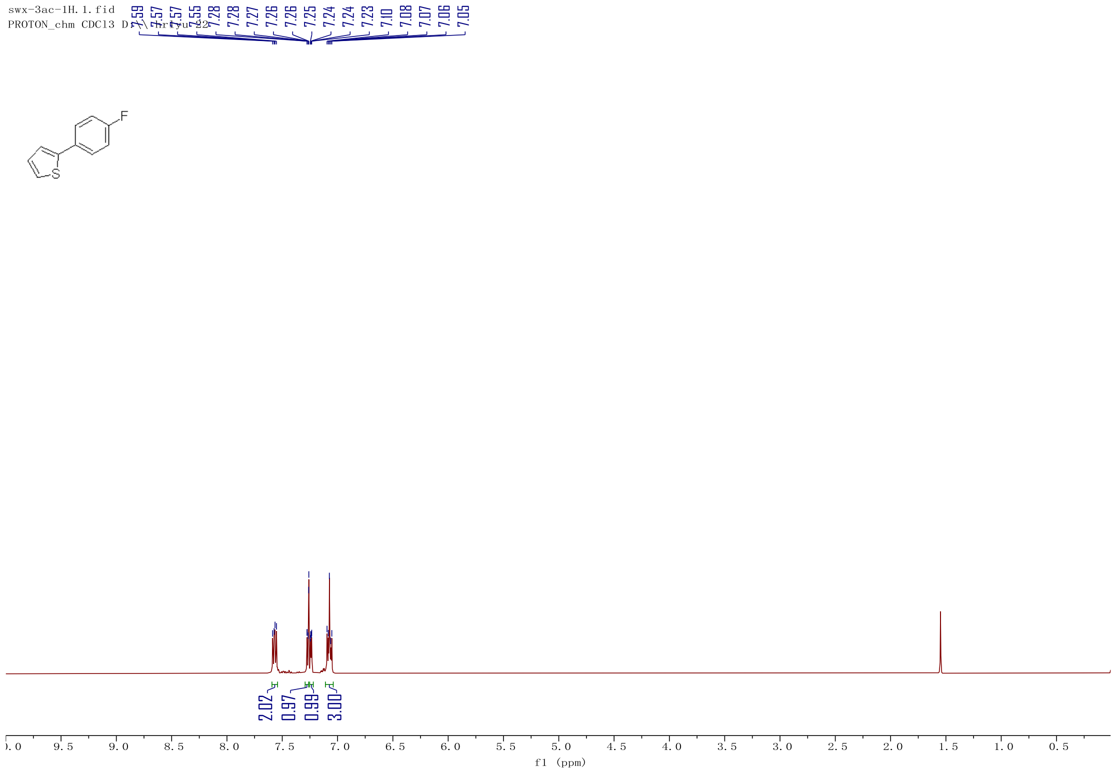

^{1}H NMR spectrum of **3v** in $CDCl_3$, 400 MHz.

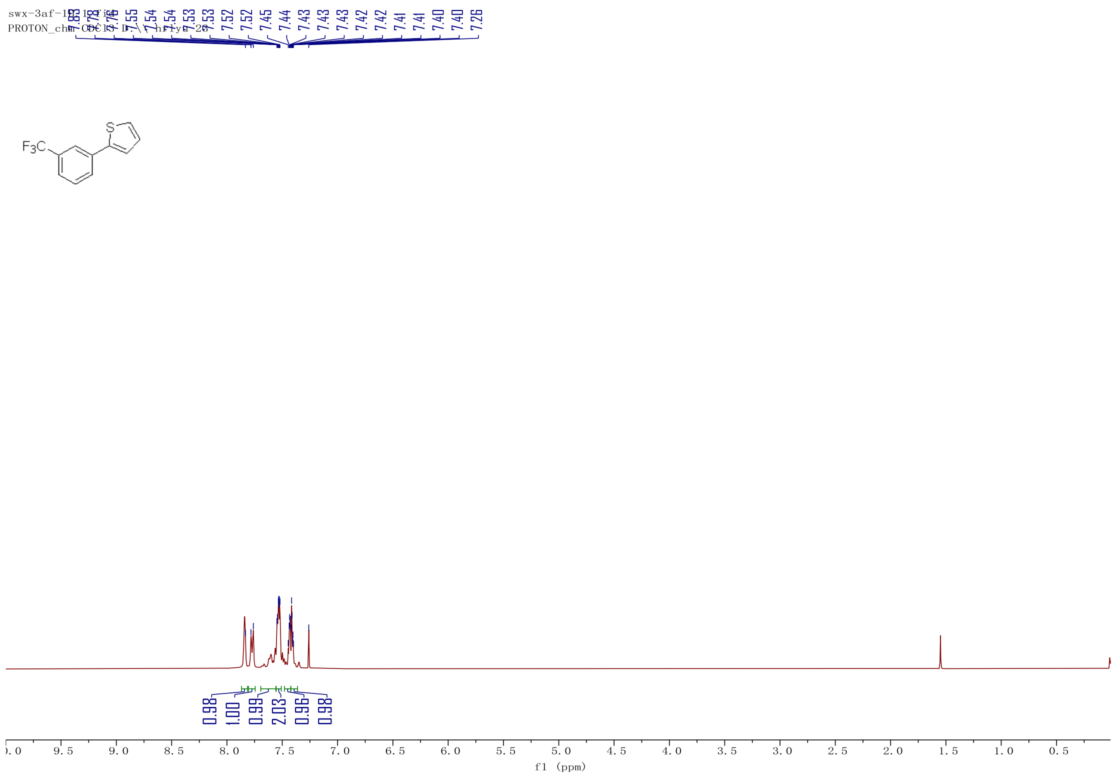

^{1}H NMR spectrum of **3y** in $CDCl_3$, 400 MHz.

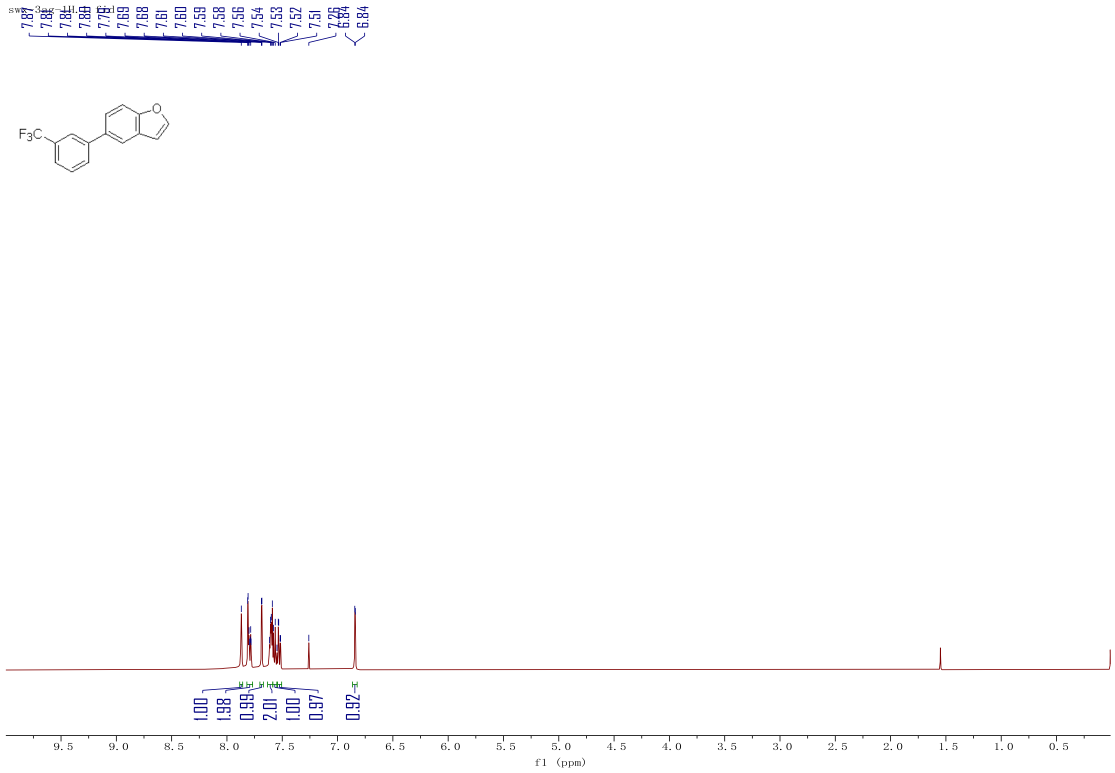

^{1}H NMR spectrum of **3z** in $CDCl_3$, 500 MHz.

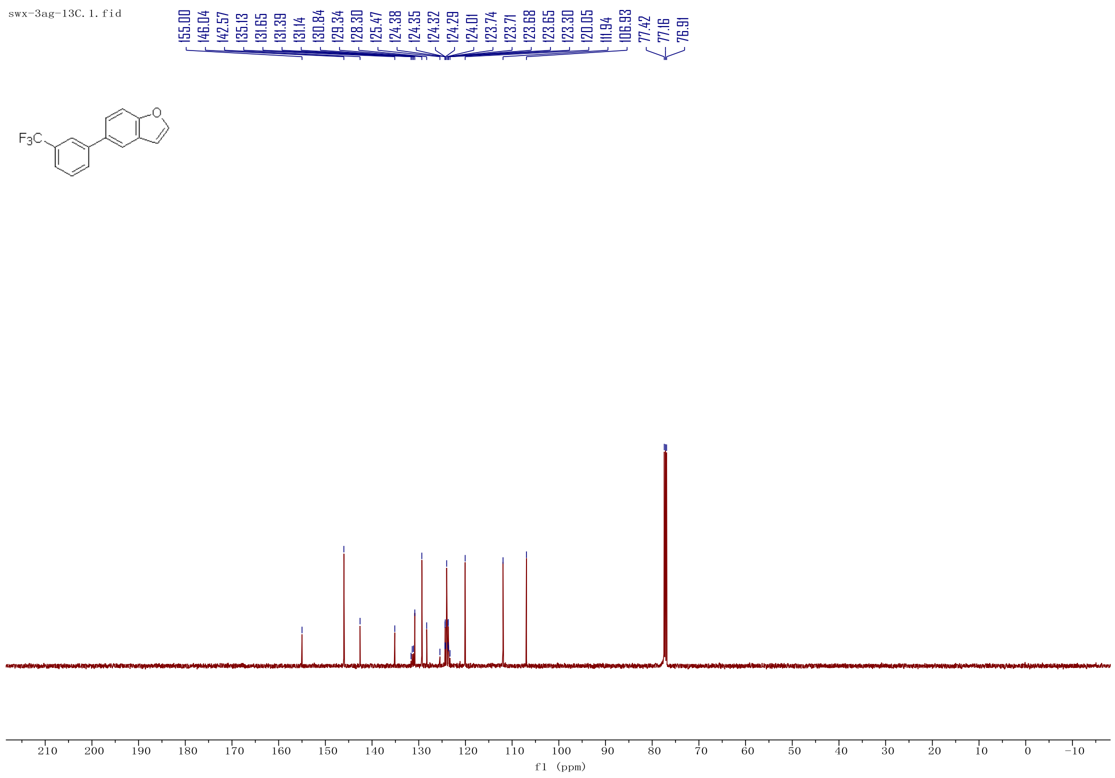

^{13}C NMR spectrum of **3z** in $CDCl_3$, 126 MHz.

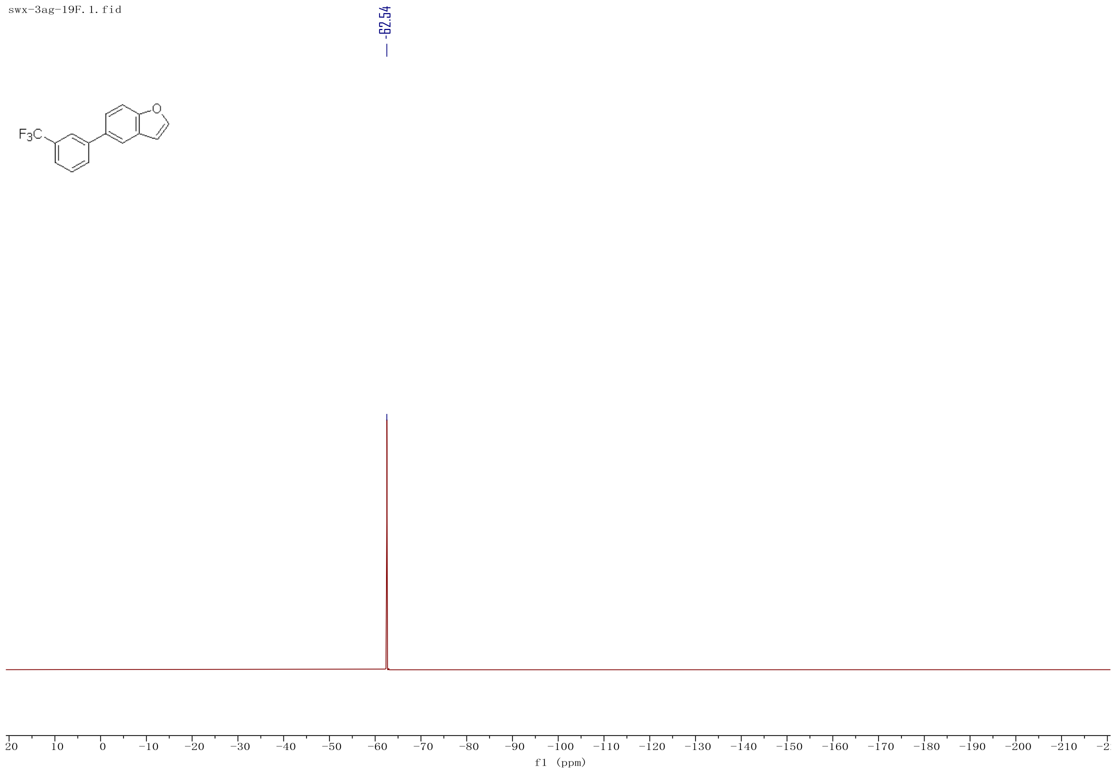

^{19}F NMR spectrum of **3z** in $CDCl_3$, 471 MHz.

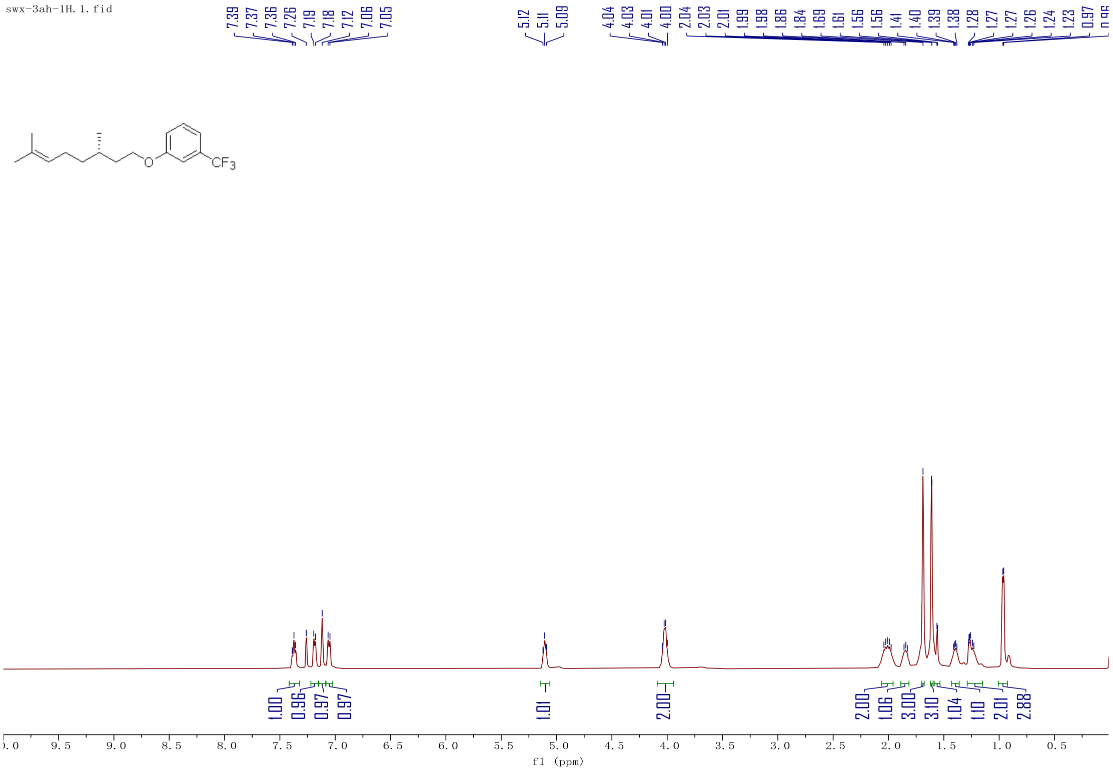

^{1}H NMR spectrum of **3aa** in $CDCl_3$, 500 MHz.

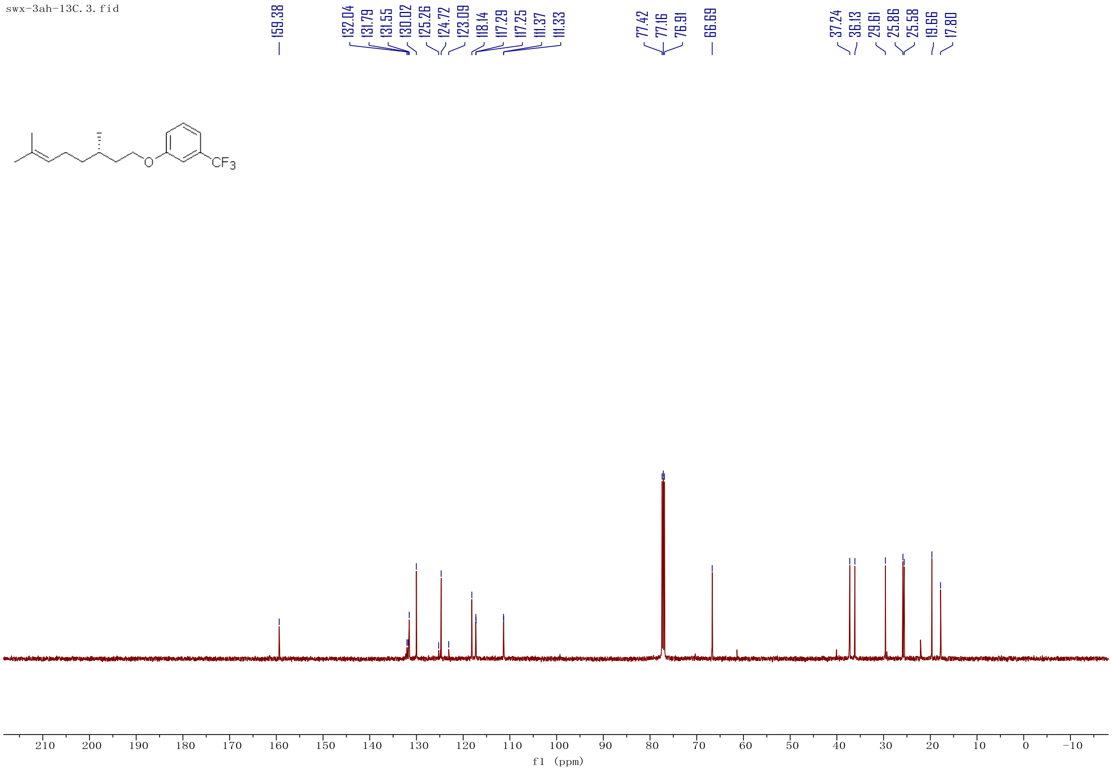

^{13}C NMR spectrum of **3aa** in $CDCl_3$, 126 MHz.

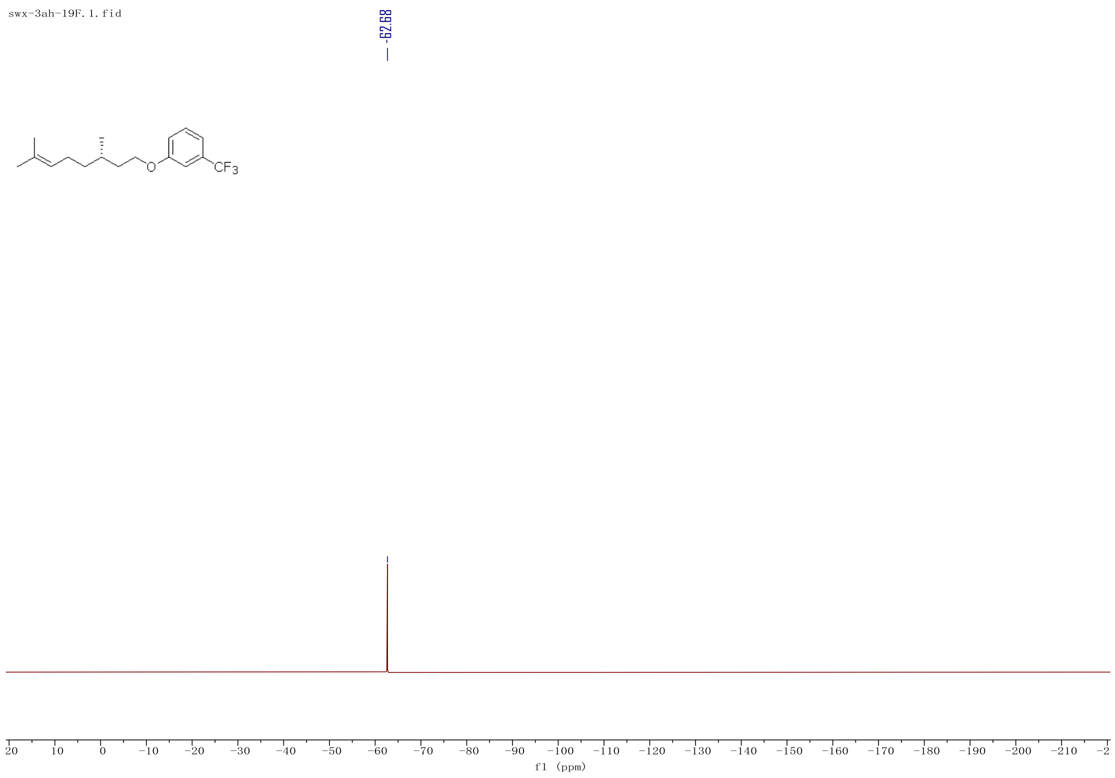

^{19}F NMR spectrum of **3aa** in $CDCl_3$, 471 MHz.

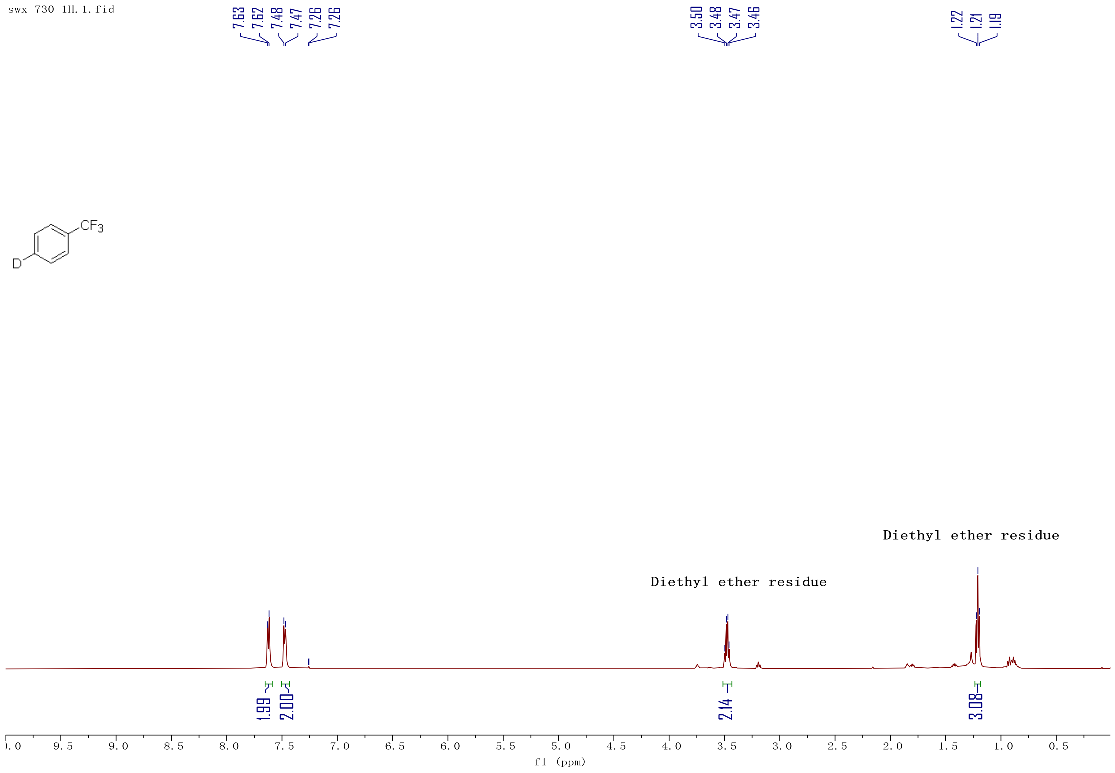

^{1}H NMR spectrum of **3j-d$_1$** in $CDCl_3$, 500 MHz.

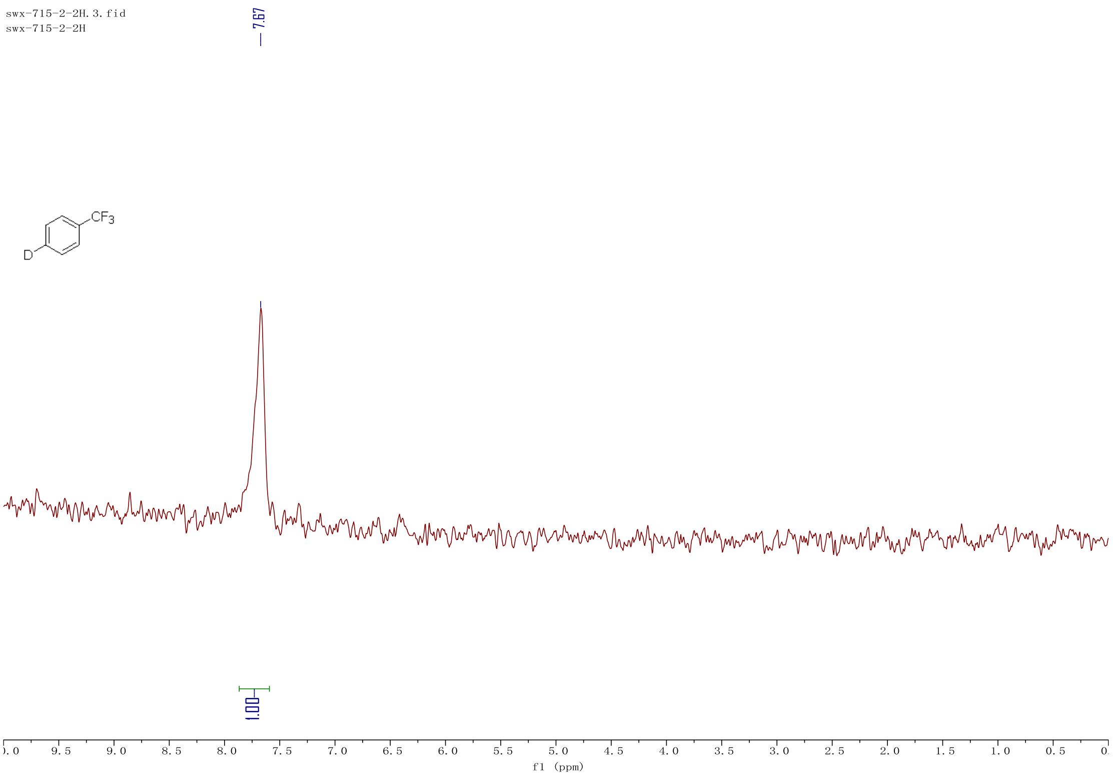

^{2}H NMR spectrum of **3j-d₁** in $CHCl_3$, 61 MHz.

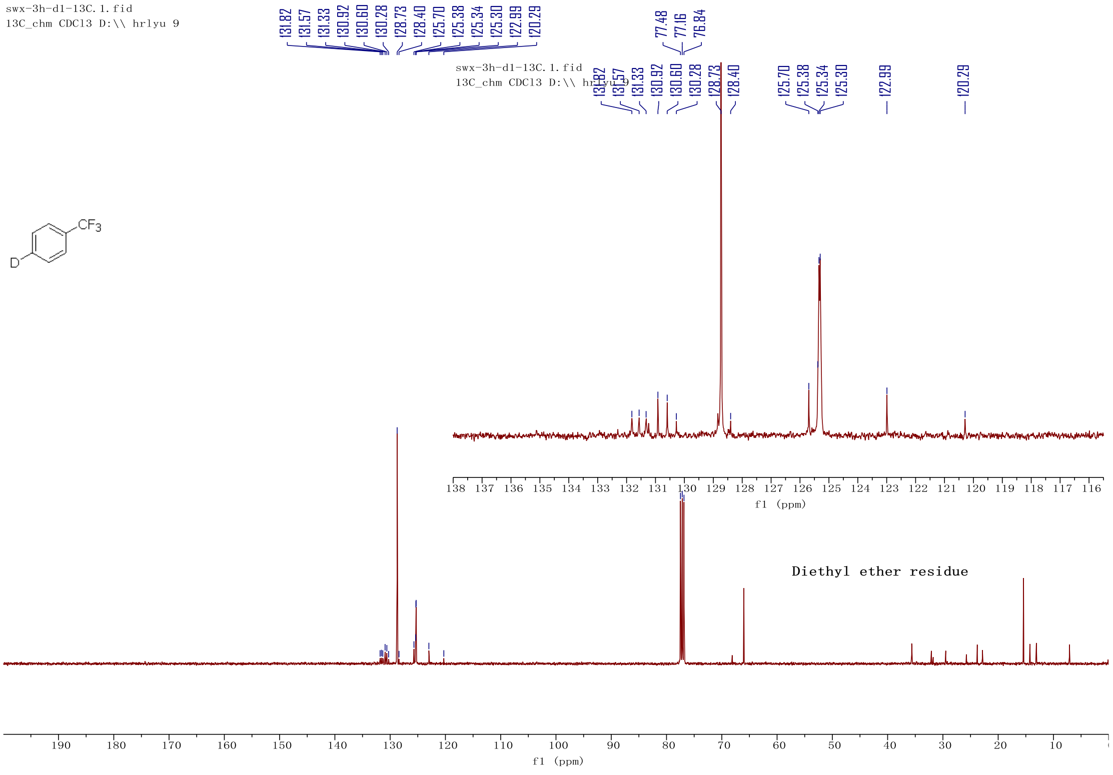

^{13}C NMR spectrum of **3j-d₁** in $CDCl_3$, 101 MHz.

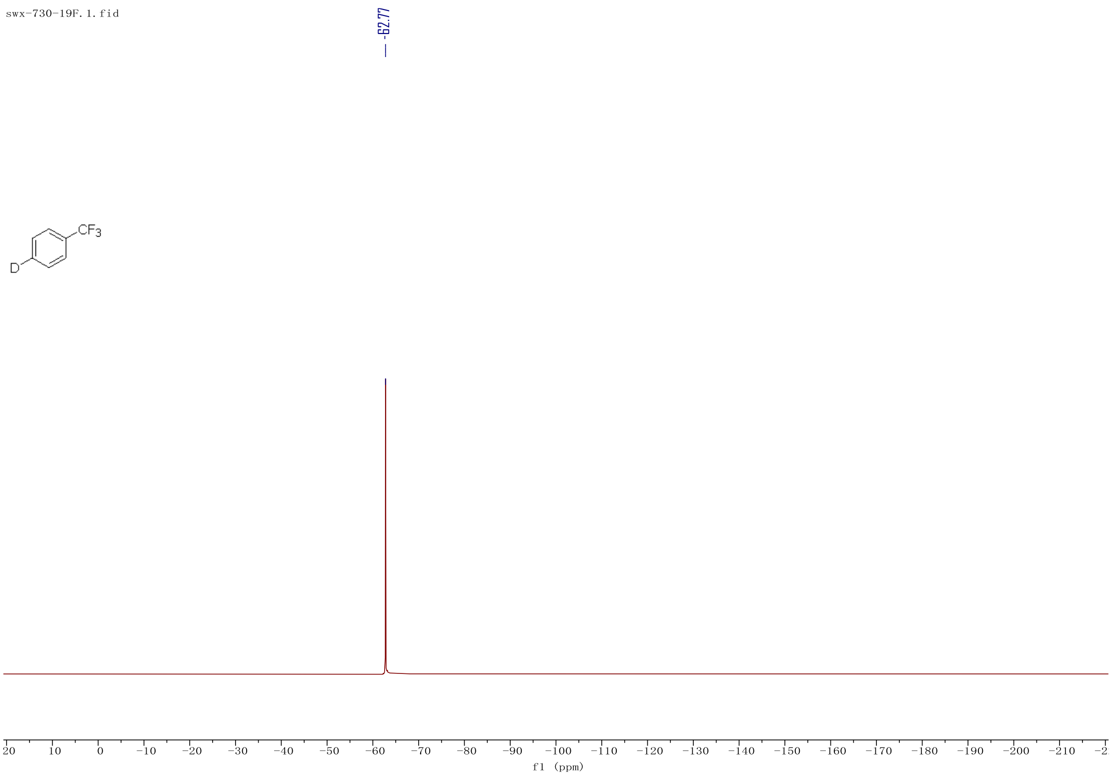

^{19}F NMR spectrum of **3j-d$_1$** in $CDCl_3$, 471 MHz.

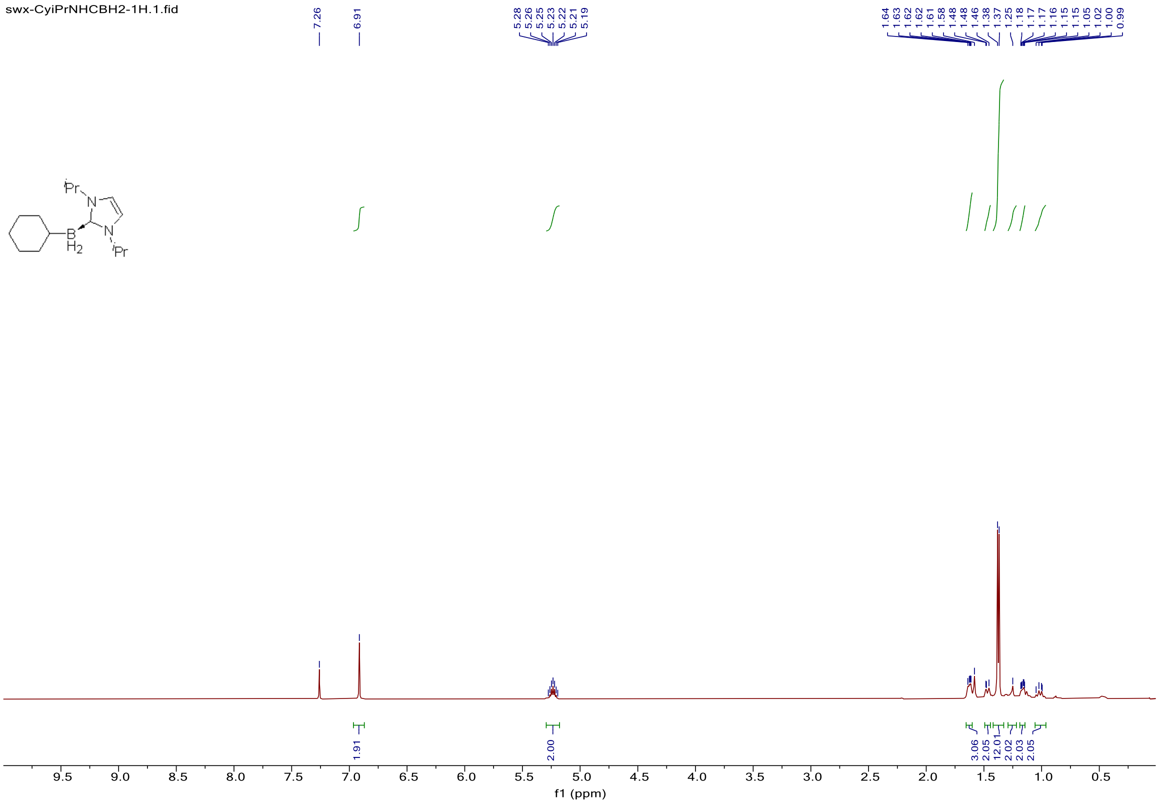

^{1}H NMR spectrum of **4a** in $CDCl_3$, 500 MHz.

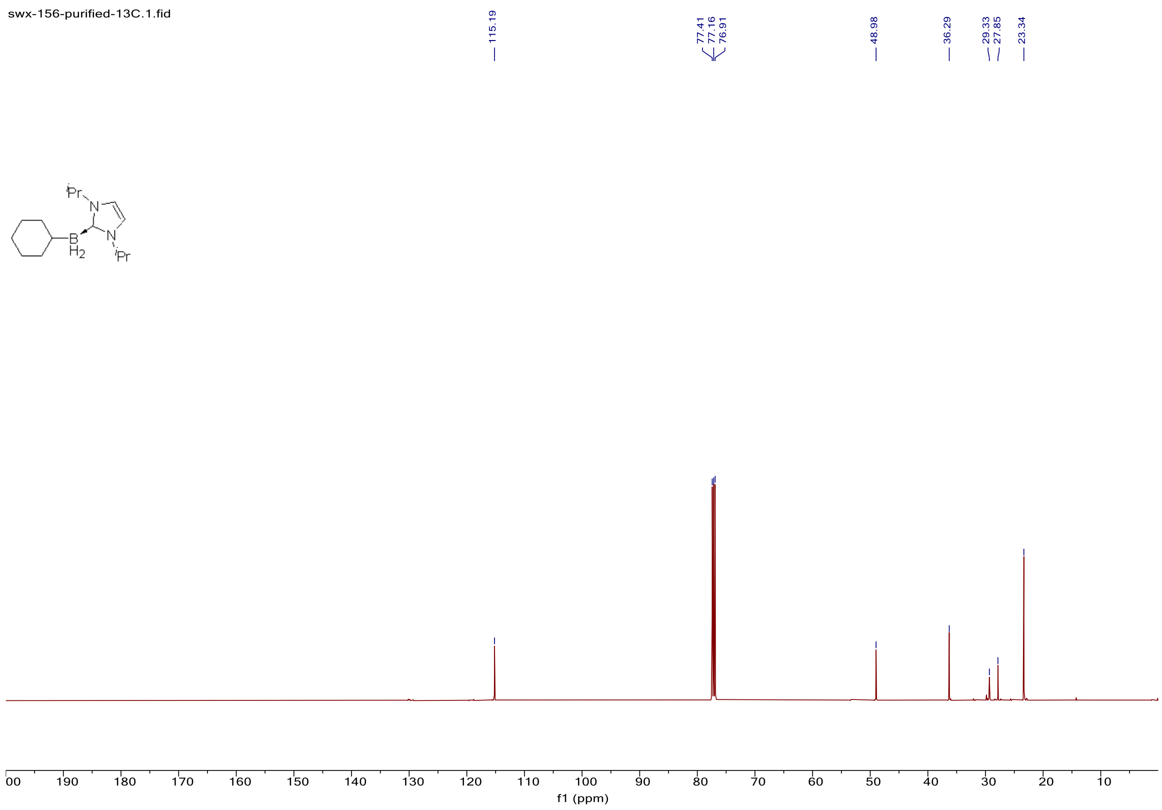

^{13}C NMR spectrum of **4a** in $CDCl_3$, 126 MHz.

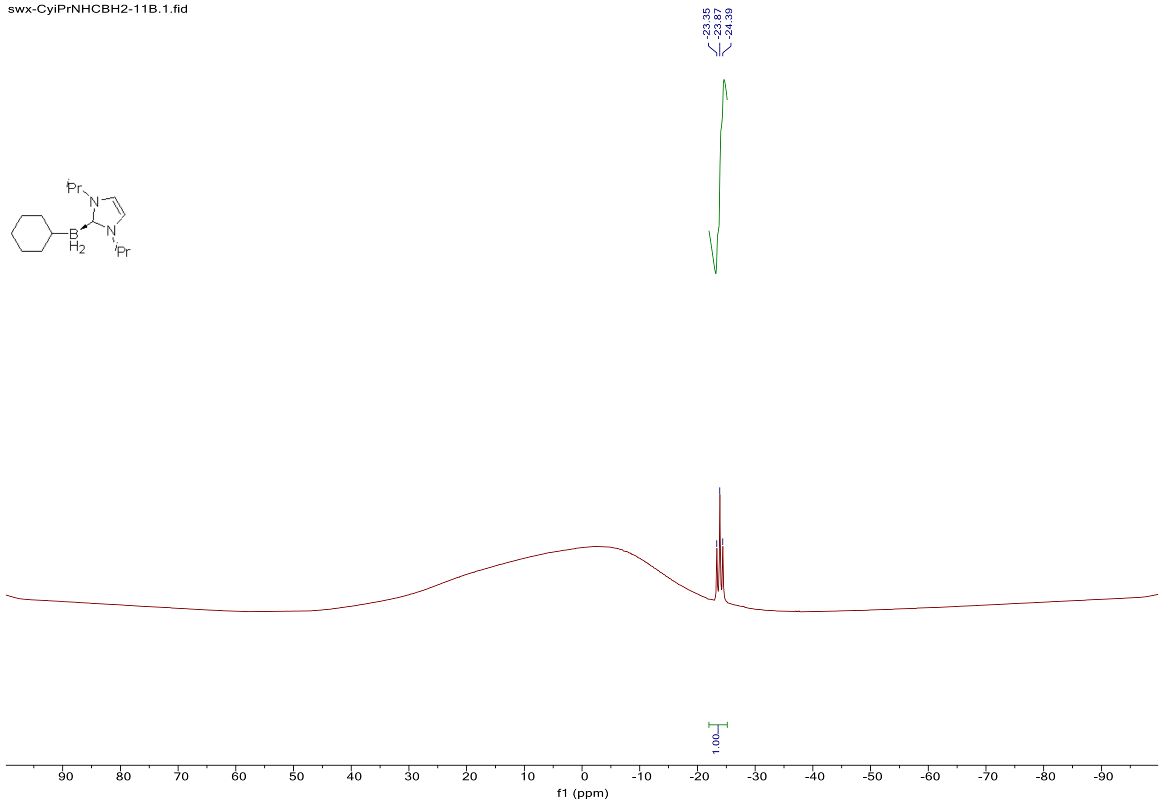

^{11}B NMR spectrum of **4a** in $CDCl_3$, 160 MHz.

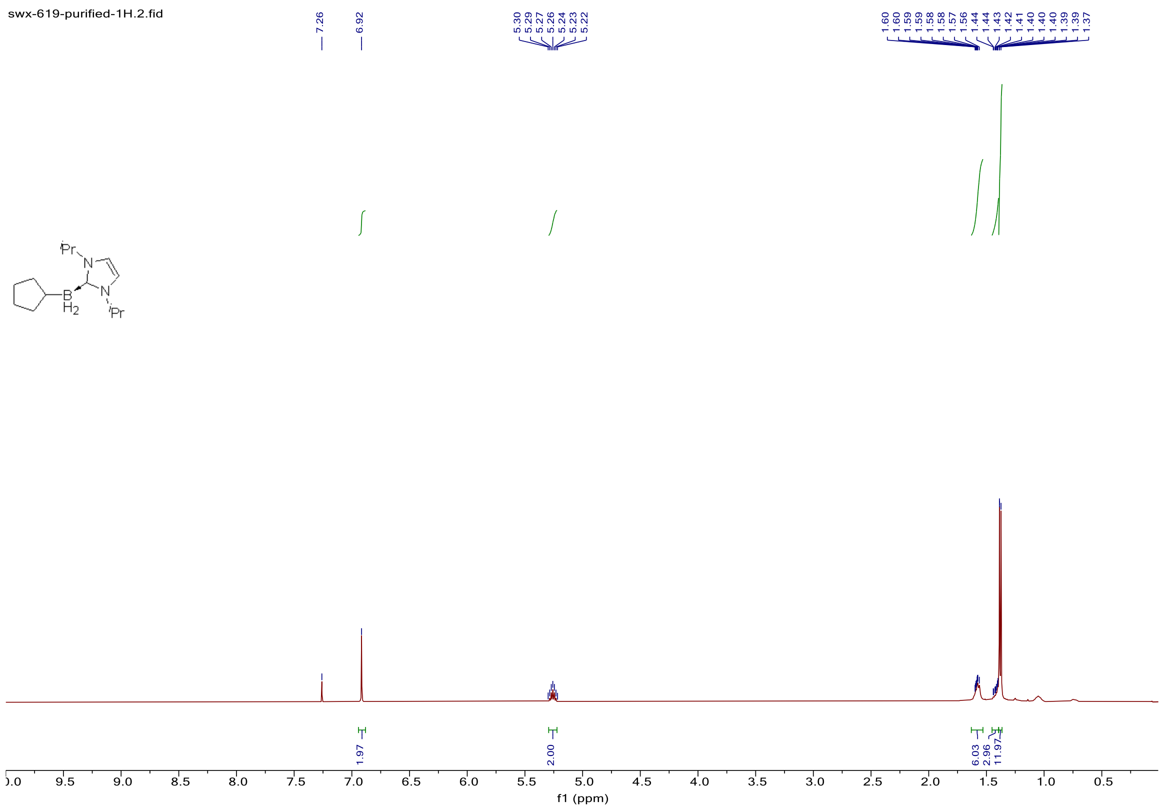

^{1}H NMR spectrum of **4b** in $CDCl_3$, 500 MHz.

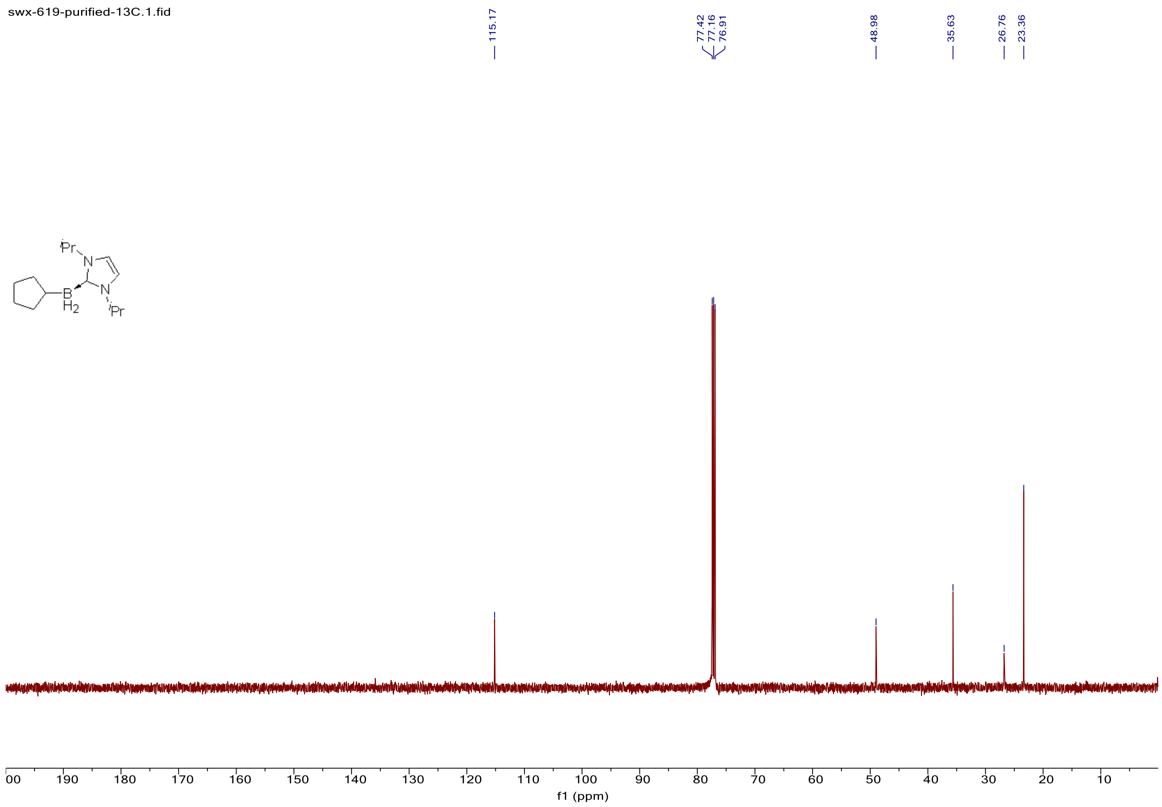

^{13}C NMR spectrum of **4b** in $CDCl_3$, 126 MHz.

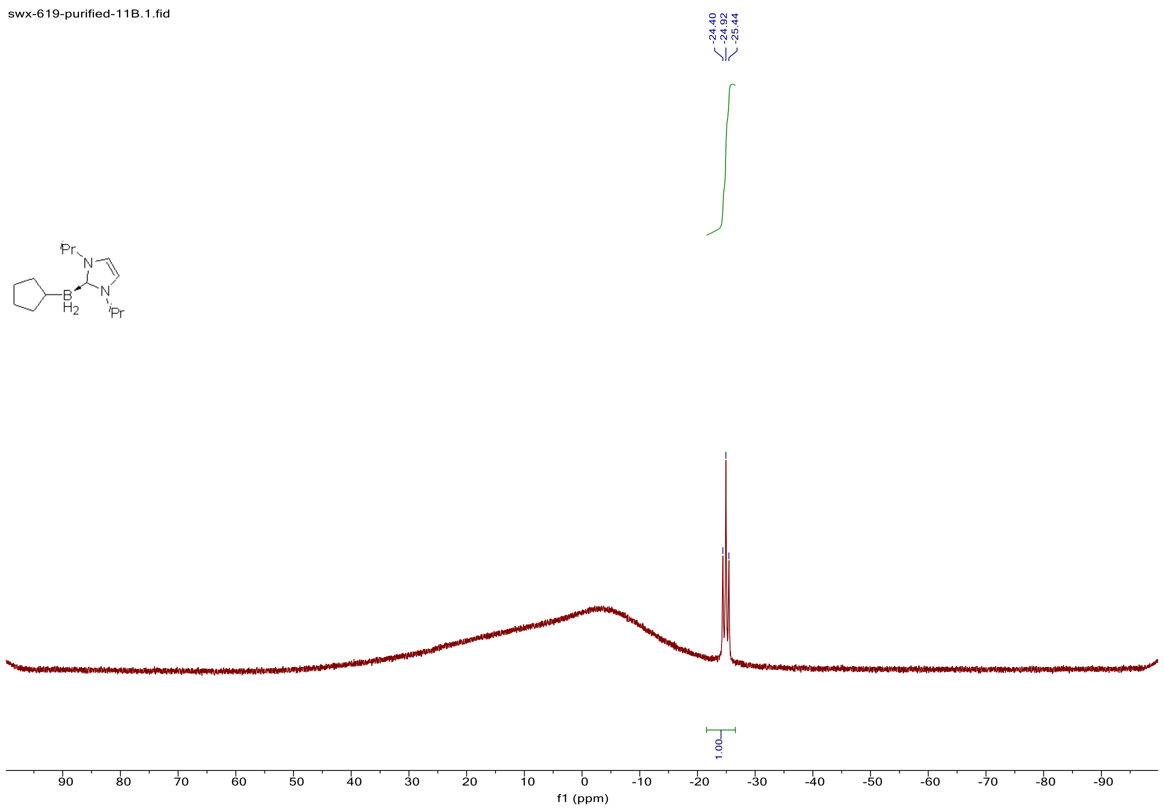

^{11}B NMR spectrum of **4b** in $CDCl_3$, 160 MHz.

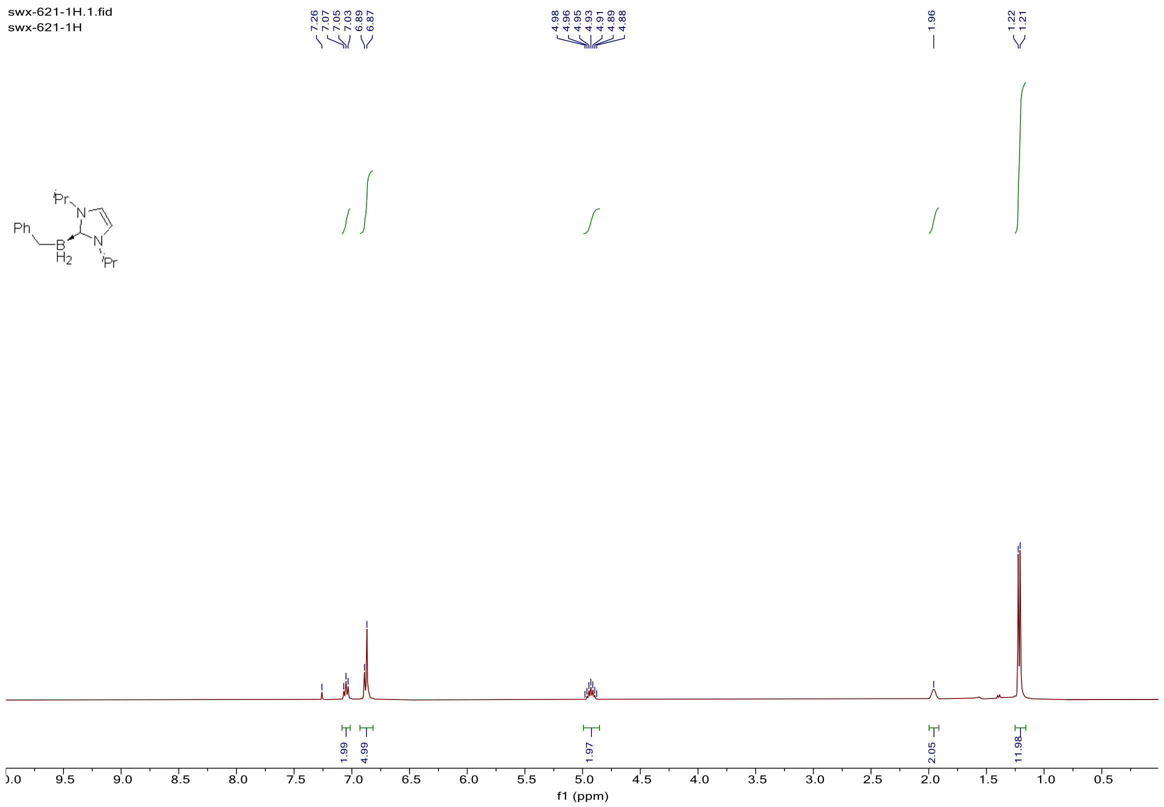

^{1}H NMR spectrum of **4c** in $CDCl_3$, 400 MHz.

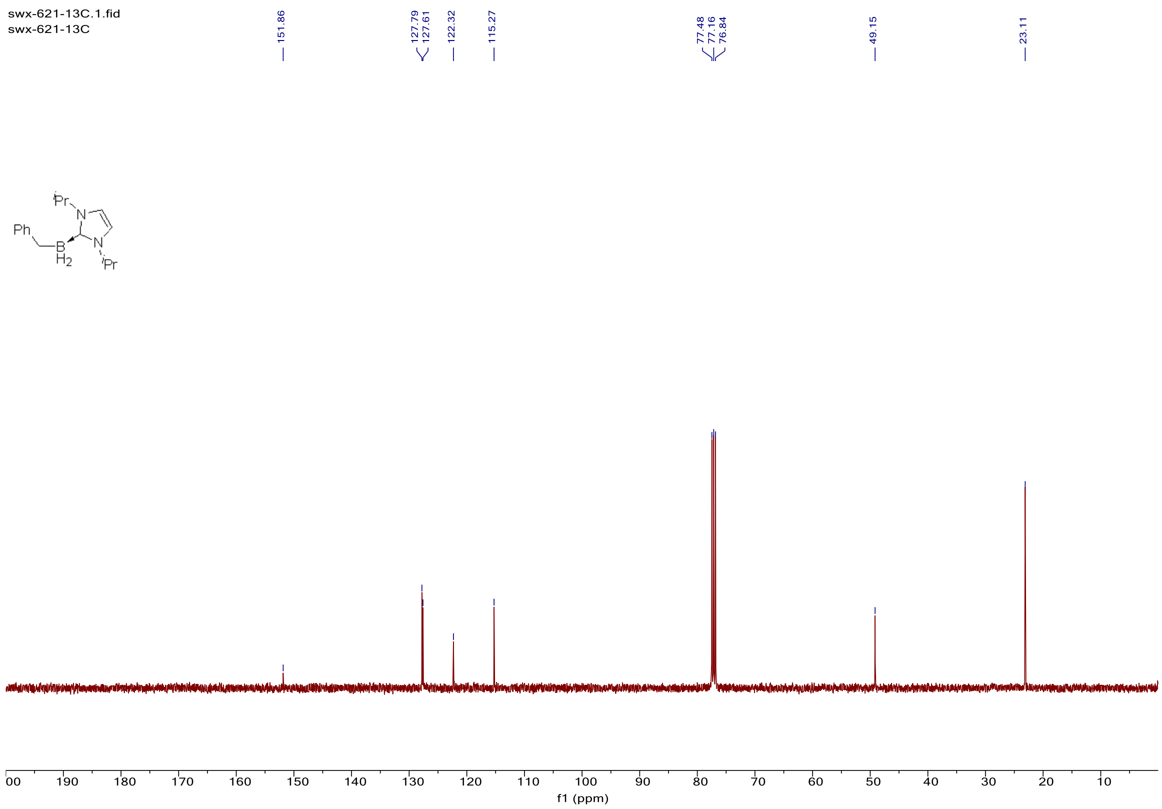

^{13}C NMR spectrum of **4c** in $CDCl_3$, 101 MHz.

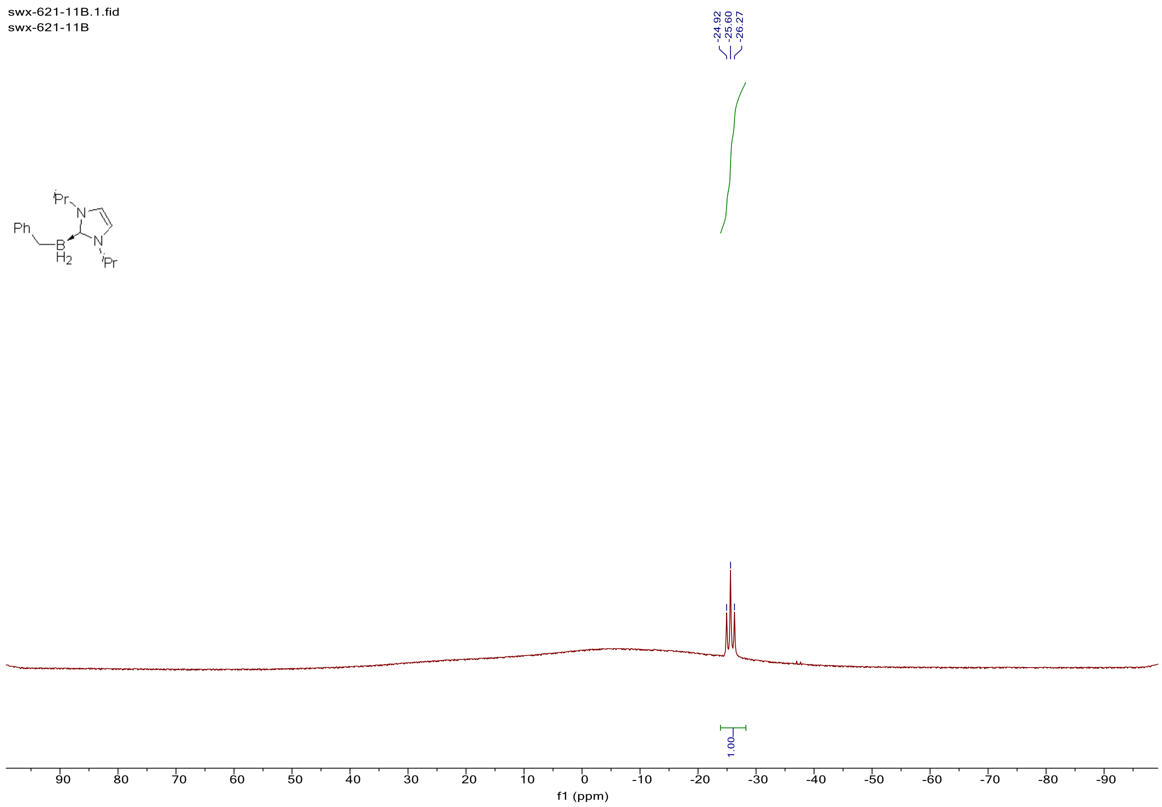

^{11}B NMR spectrum of **4c** in $CDCl_3$, 128 MHz.

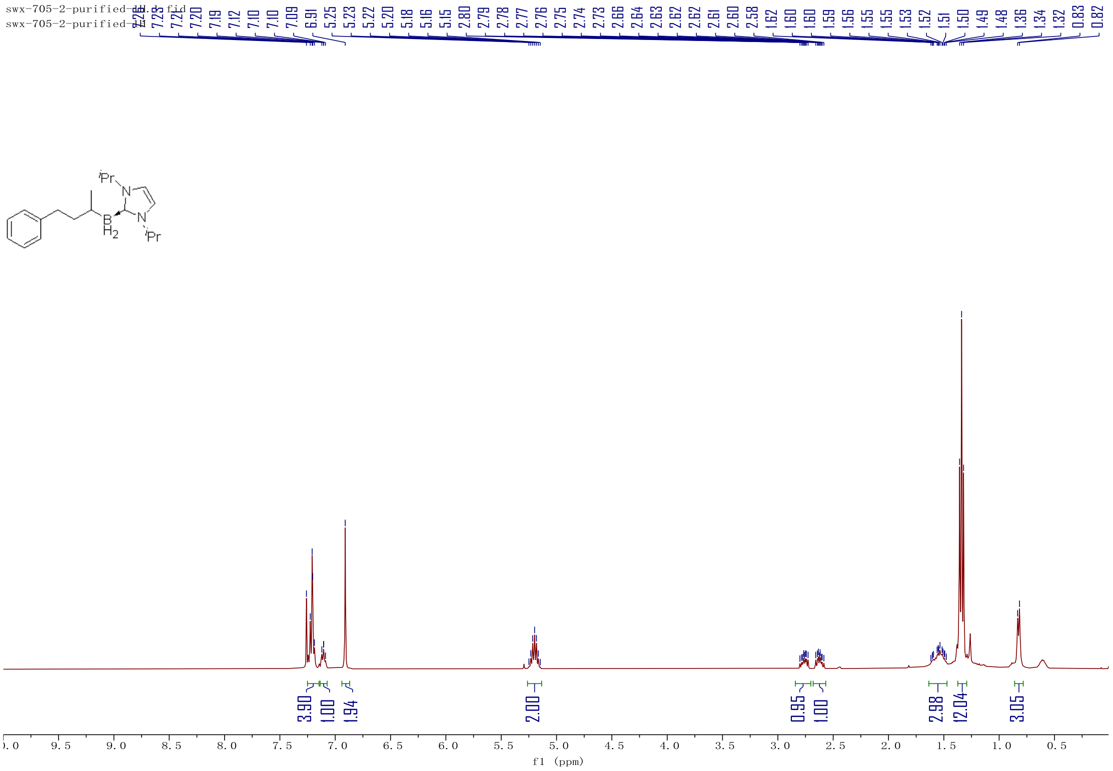

^{1}H NMR spectrum of **4d** in $CDCl_3$, 400 MHz.

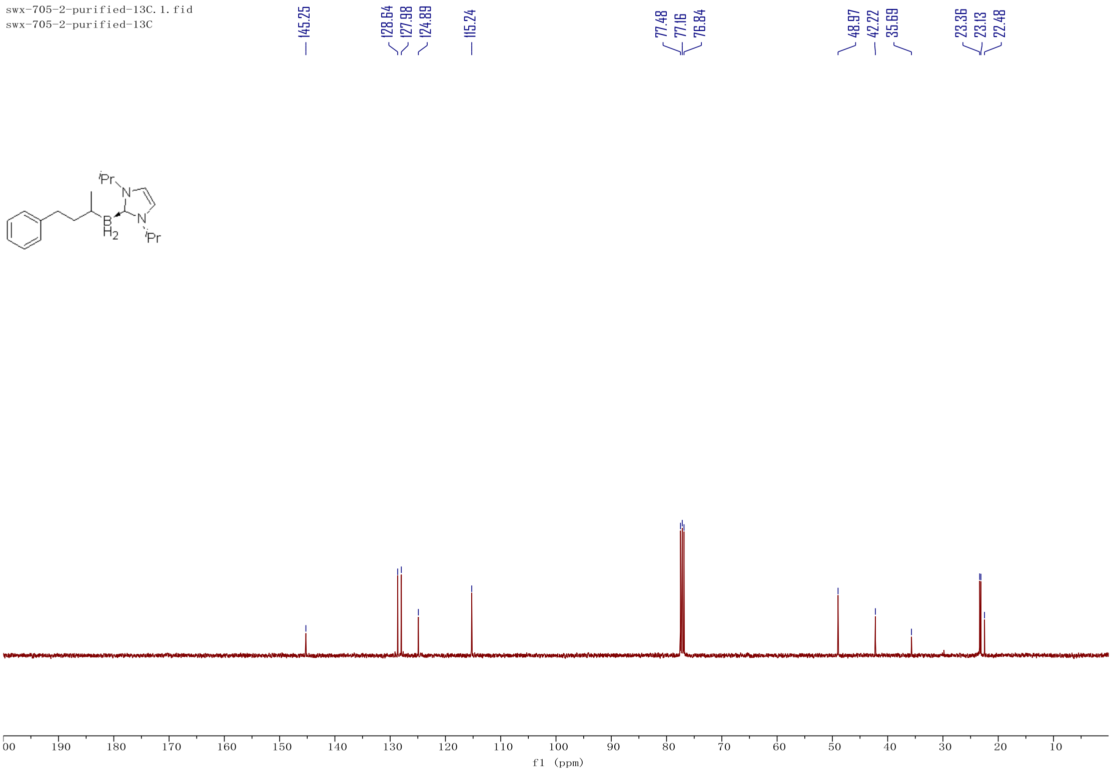

^{13}C NMR spectrum of **4d** in $CDCl_3$, 101 MHz.

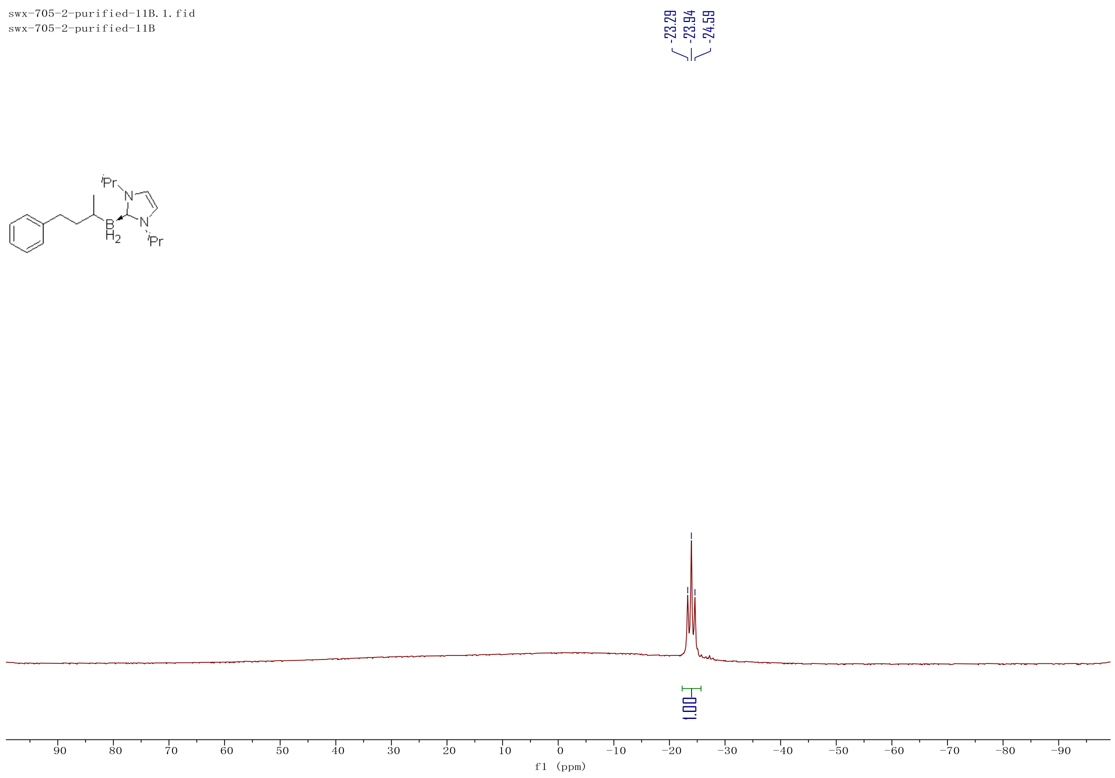

^{11}B NMR spectrum of **4d** in $CDCl_3$, 128 MHz.

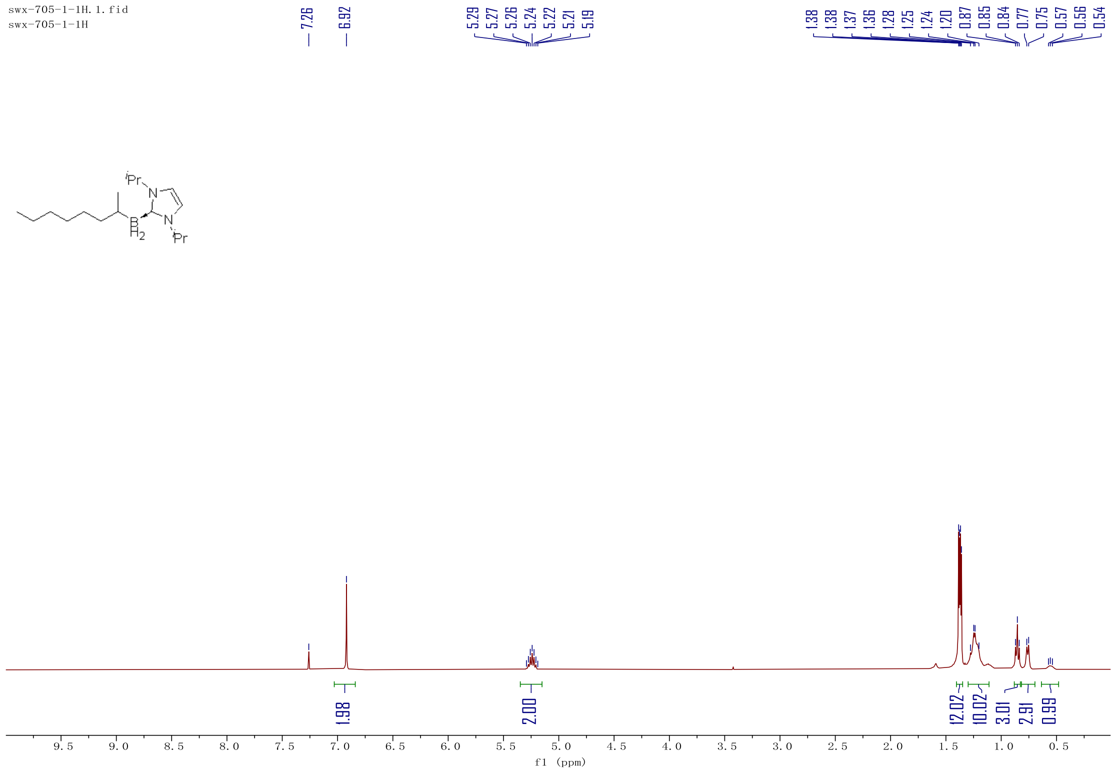

^{1}H NMR spectrum of **4e** in $CDCl_3$, 400 MHz.

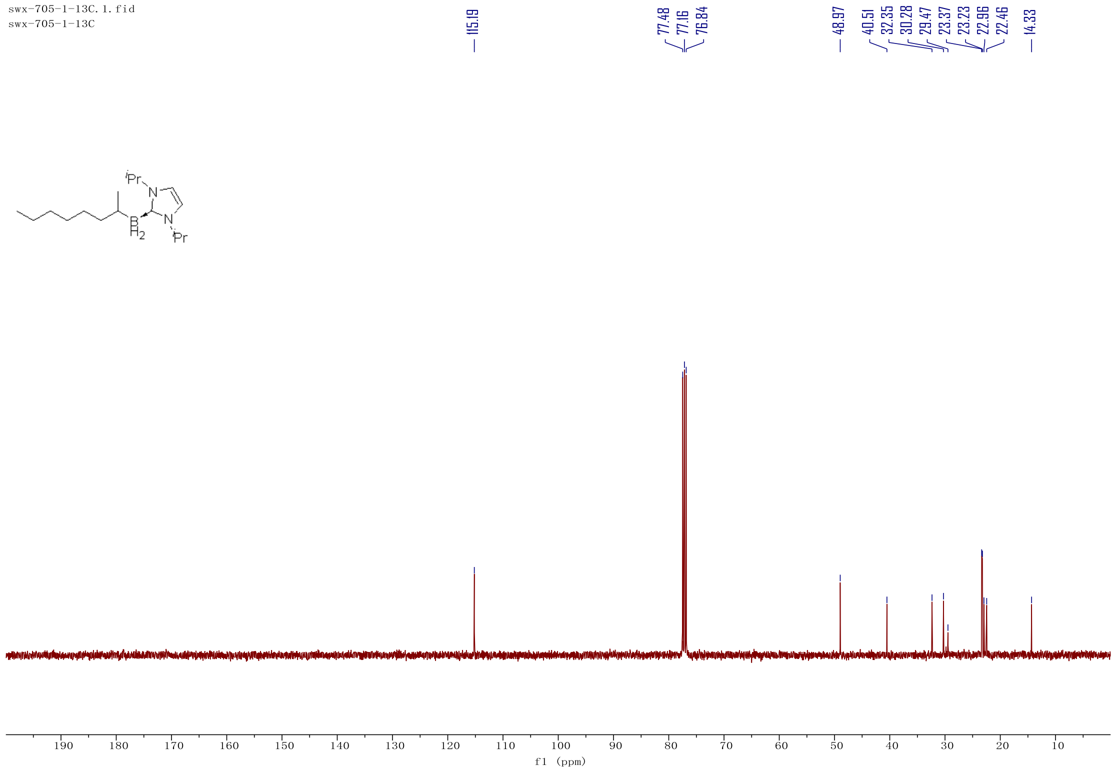

^{13}C NMR spectrum of **4e** in $CDCl_3$, 101 MHz.

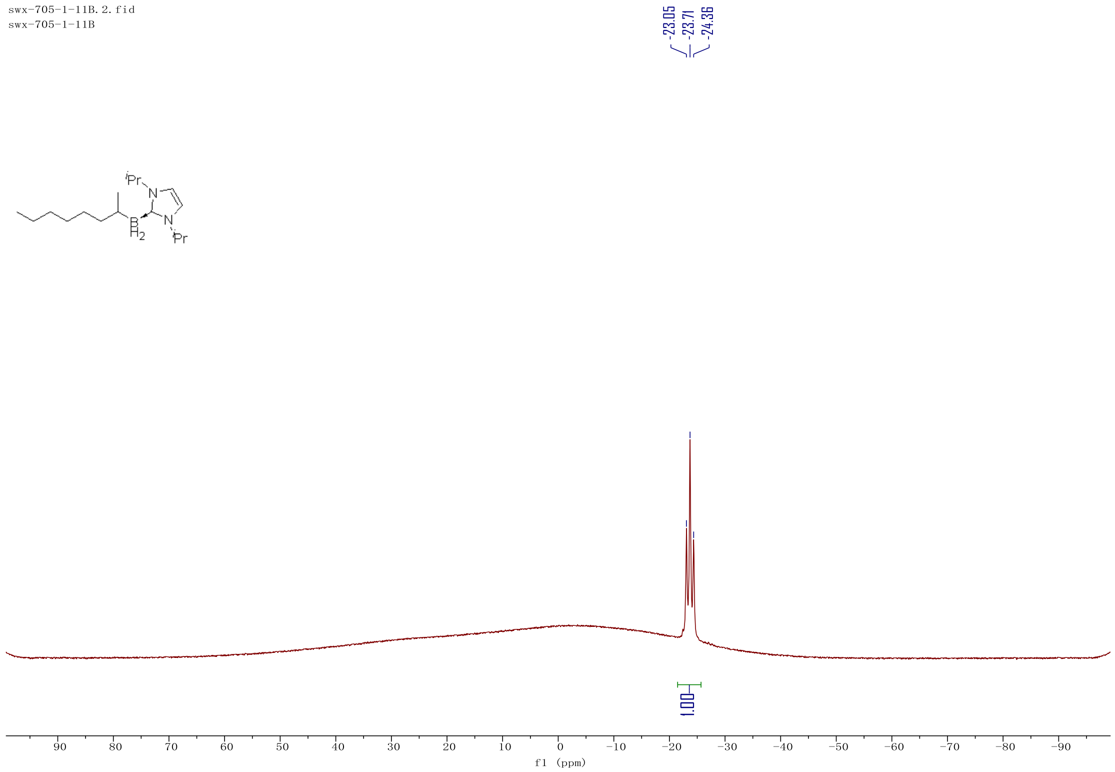

^{11}B NMR spectrum of **4e** in $CDCl_3$, 128 MHz.

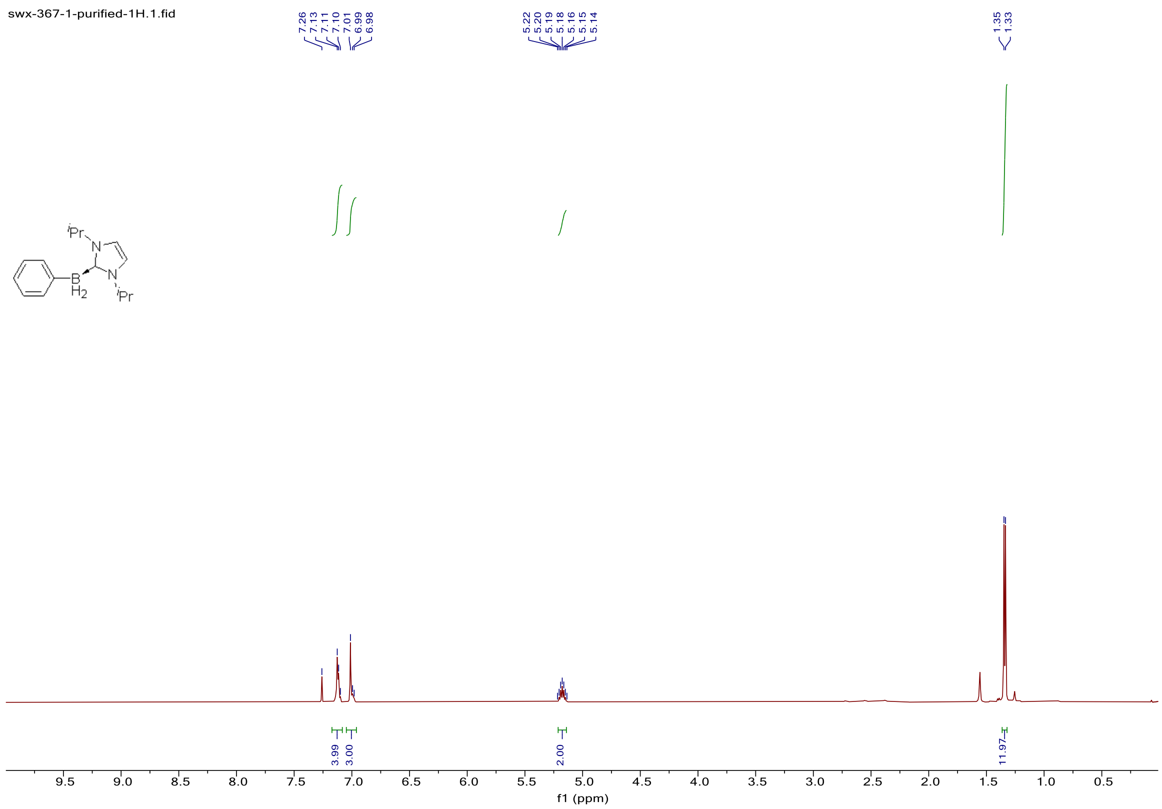

^{1}H NMR spectrum of **4f** in $CDCl_3$, 500 MHz.

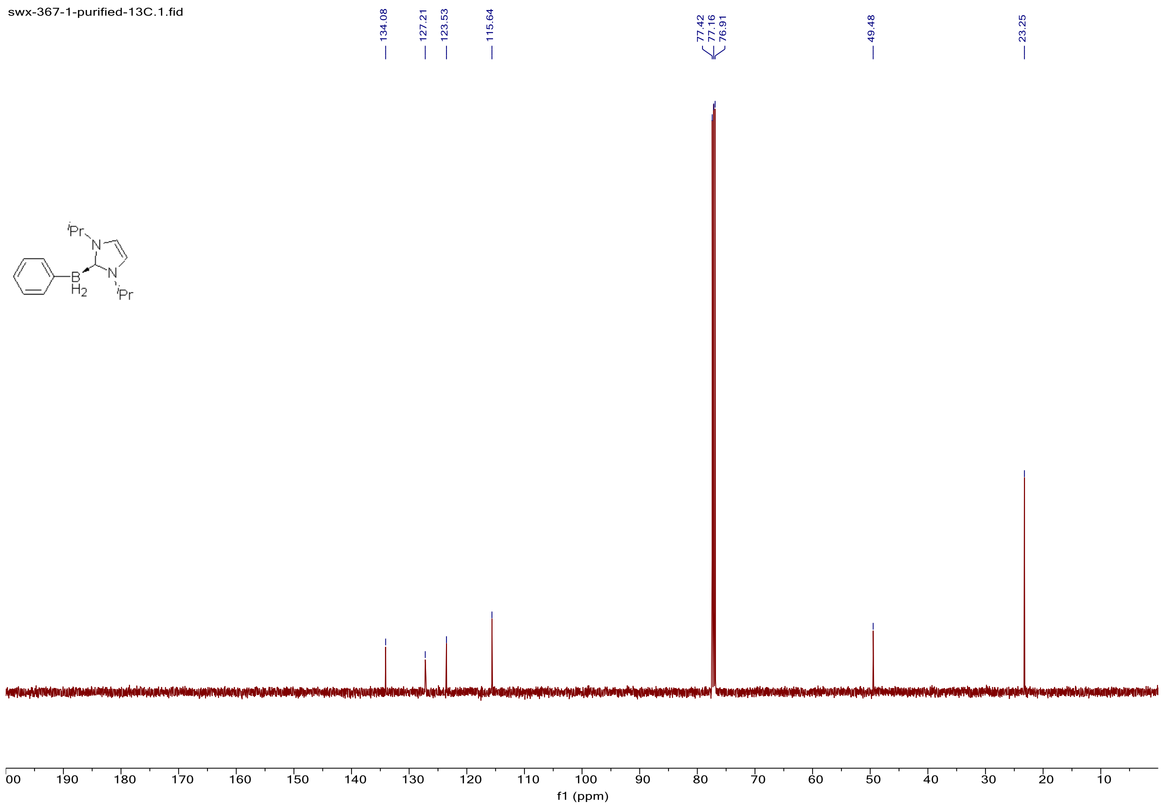

^{13}C NMR spectrum of **4f** in $CDCl_3$, 126 MHz.

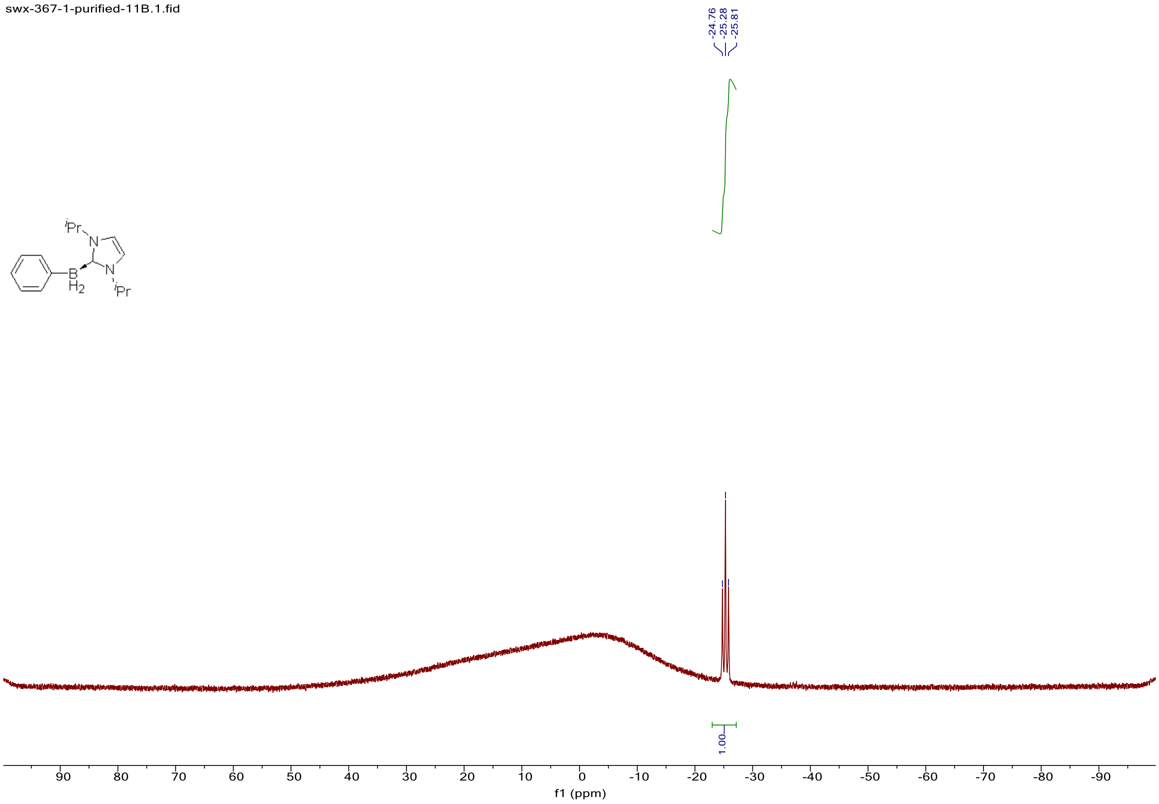

^{11}B NMR spectrum of **4f** in $CDCl_3$, 160 MHz.

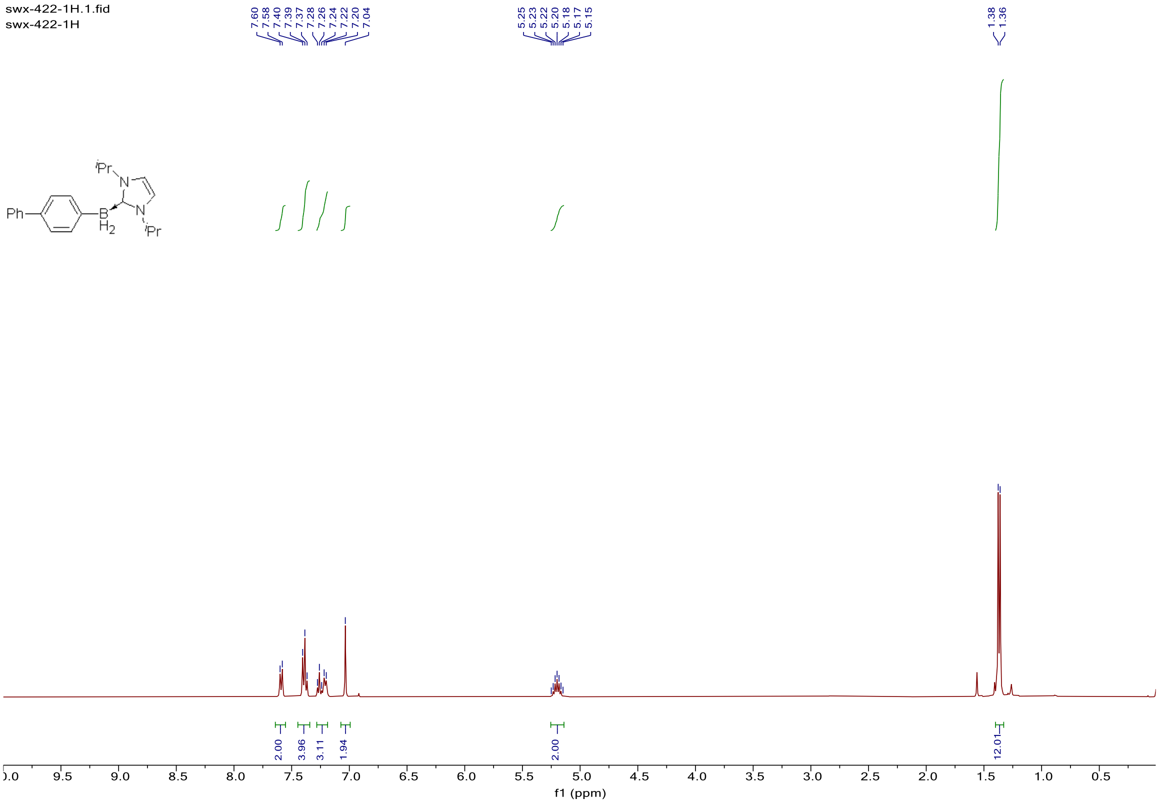

^{1}H NMR spectrum of **4g** in $CDCl_3$, 400 MHz.

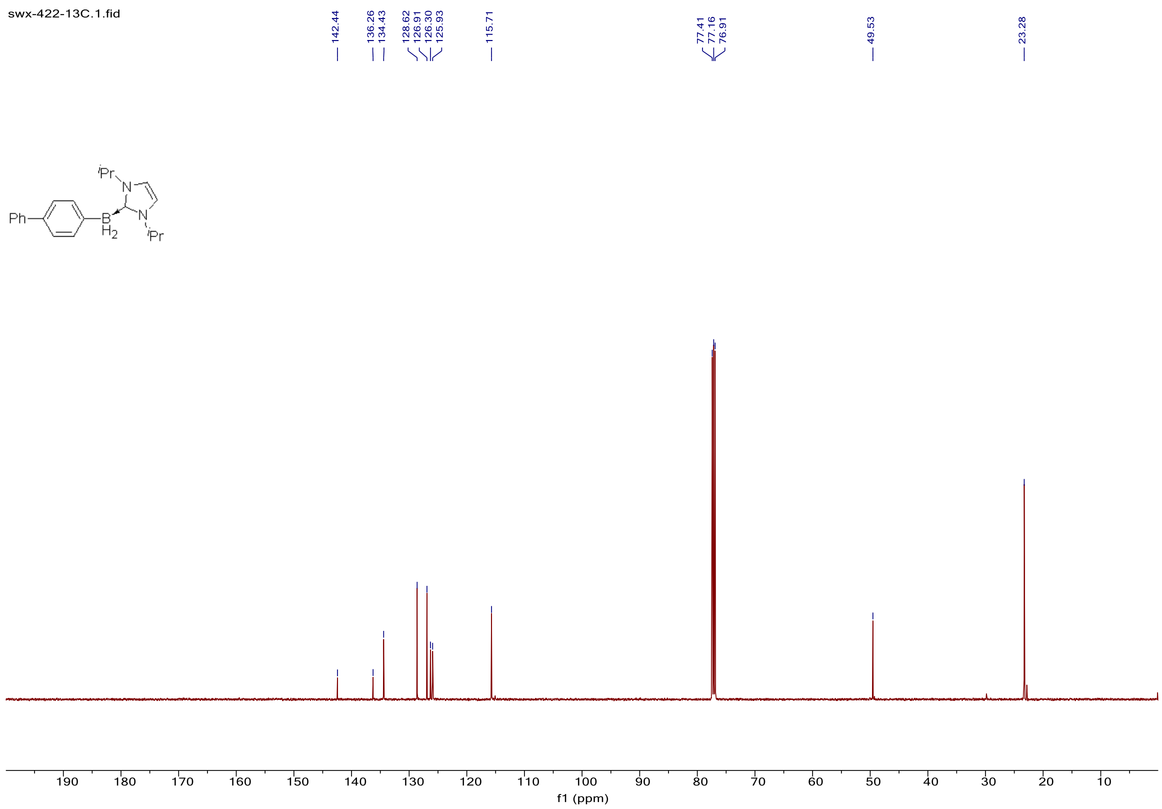

^{13}C NMR spectrum of **4g** in $CDCl_3$, 126 MHz.

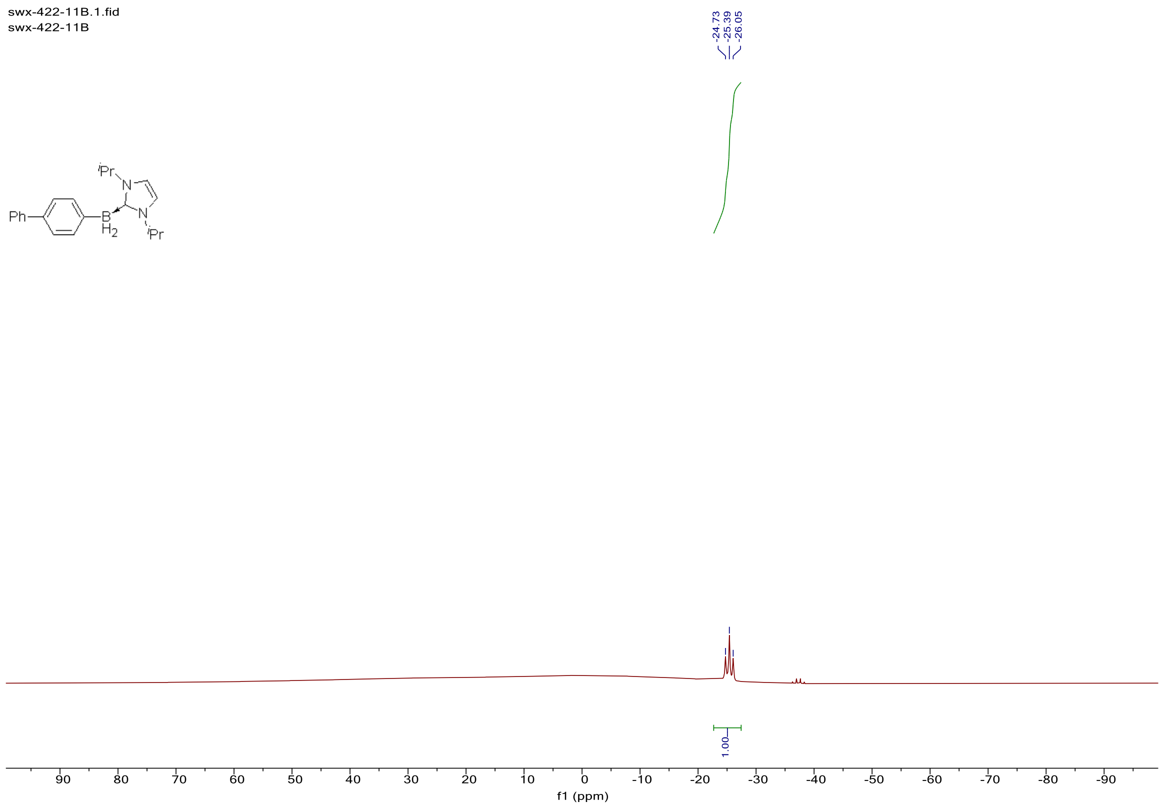

^{11}B NMR spectrum of **4g** in $CDCl_3$, 128 MHz.

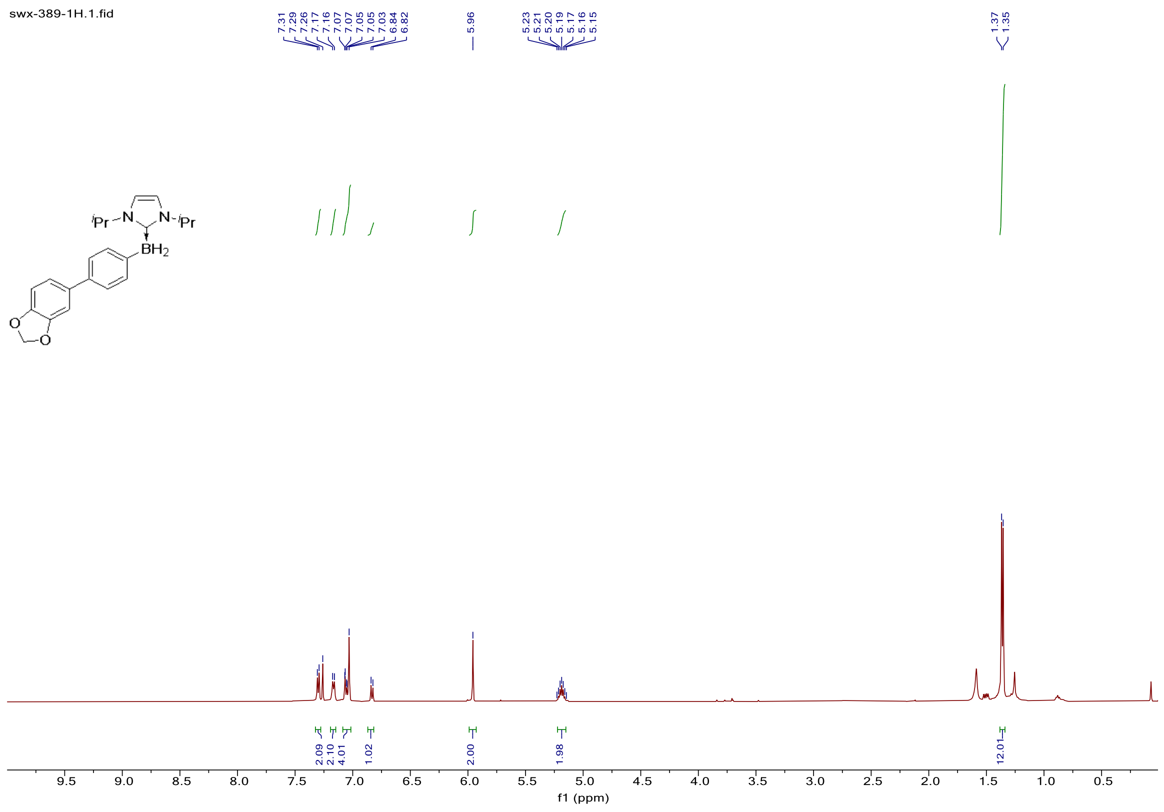

^{1}H NMR spectrum of **4h** in $CDCl_3$, 500 MHz.

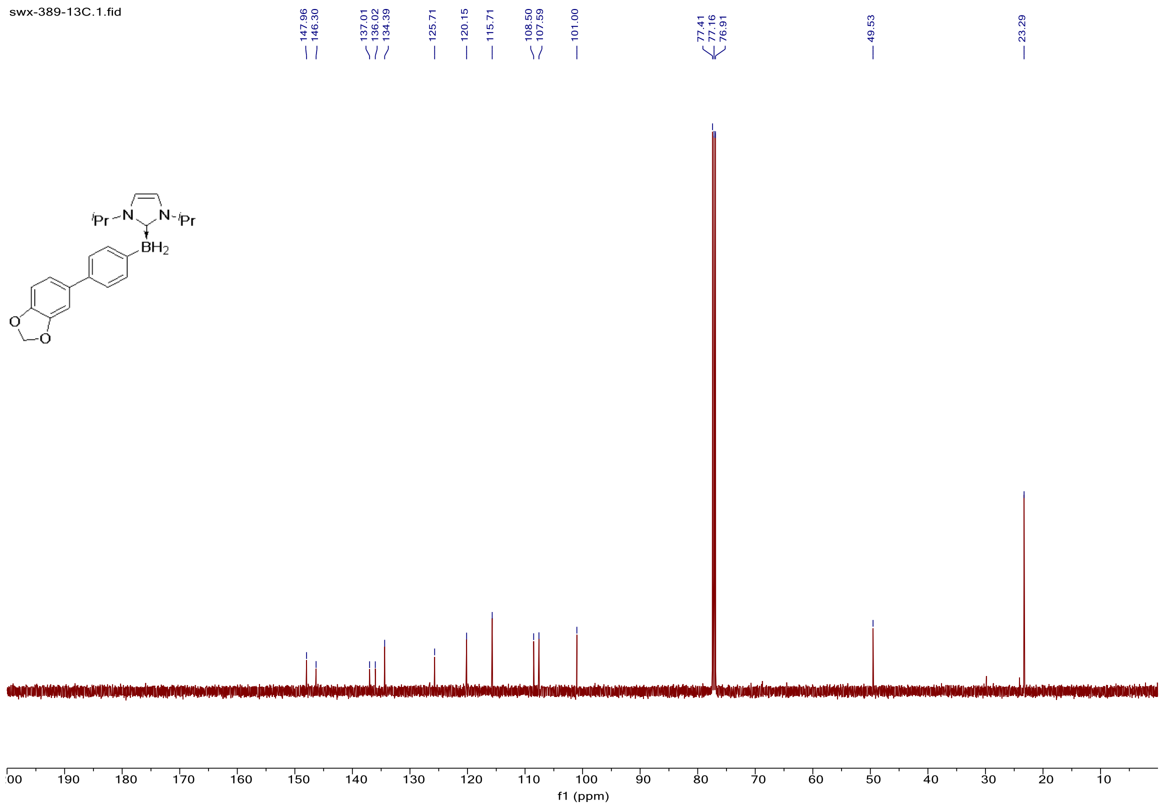

^{13}C NMR spectrum of **4h** in $CDCl_3$, 126 MHz.

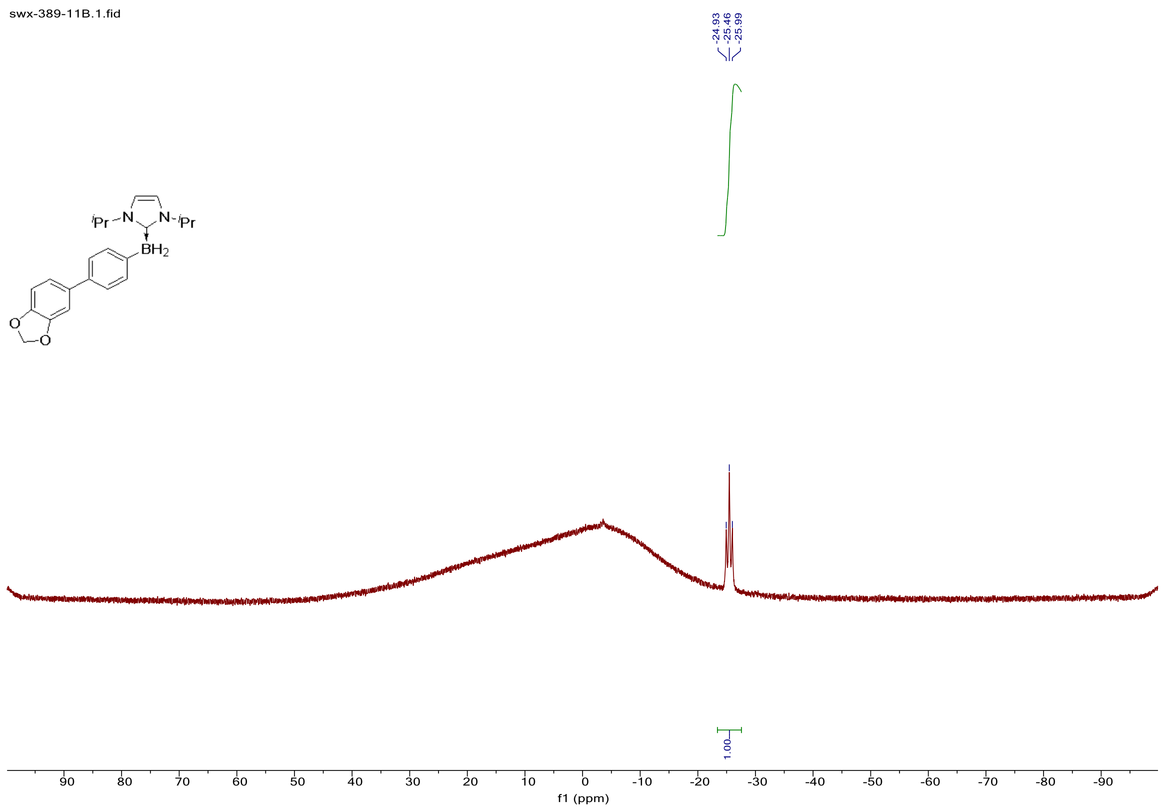

^{11}B NMR spectrum of **4h** in $CDCl_3$, 160 MHz.

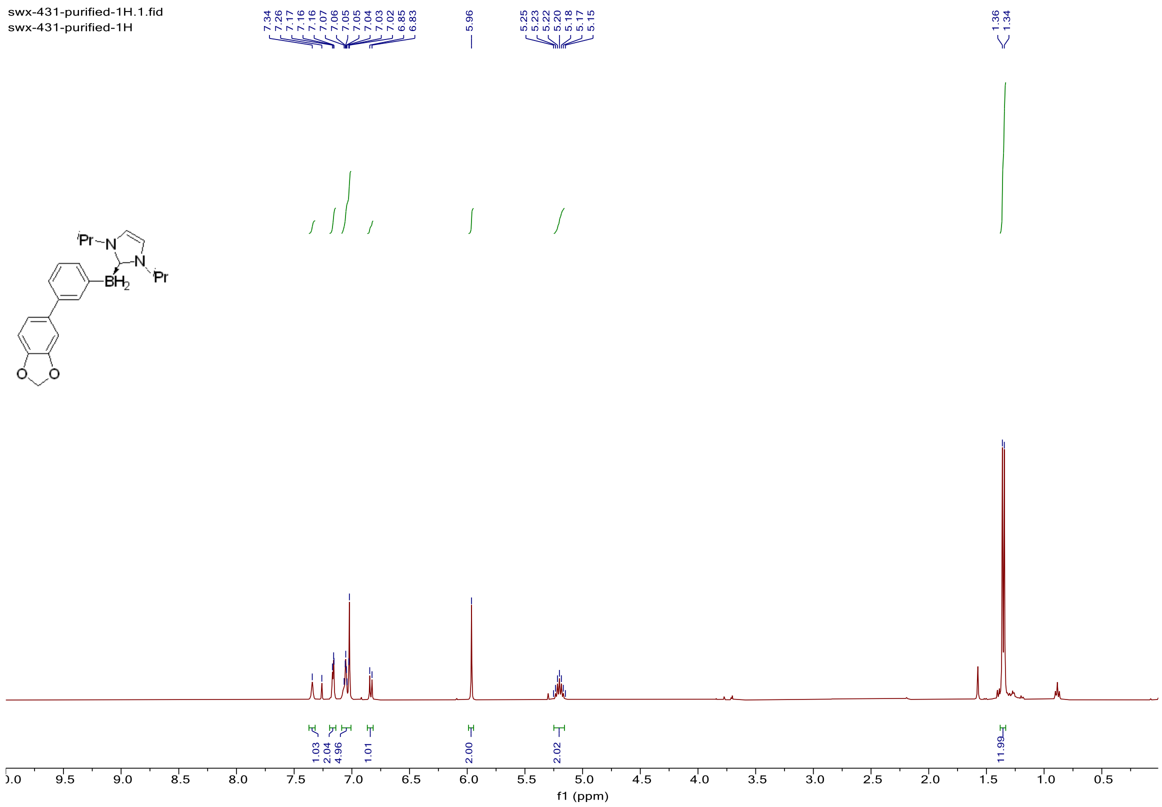

^{1}H NMR spectrum of **4i** in $CDCl_3$, 400 MHz.

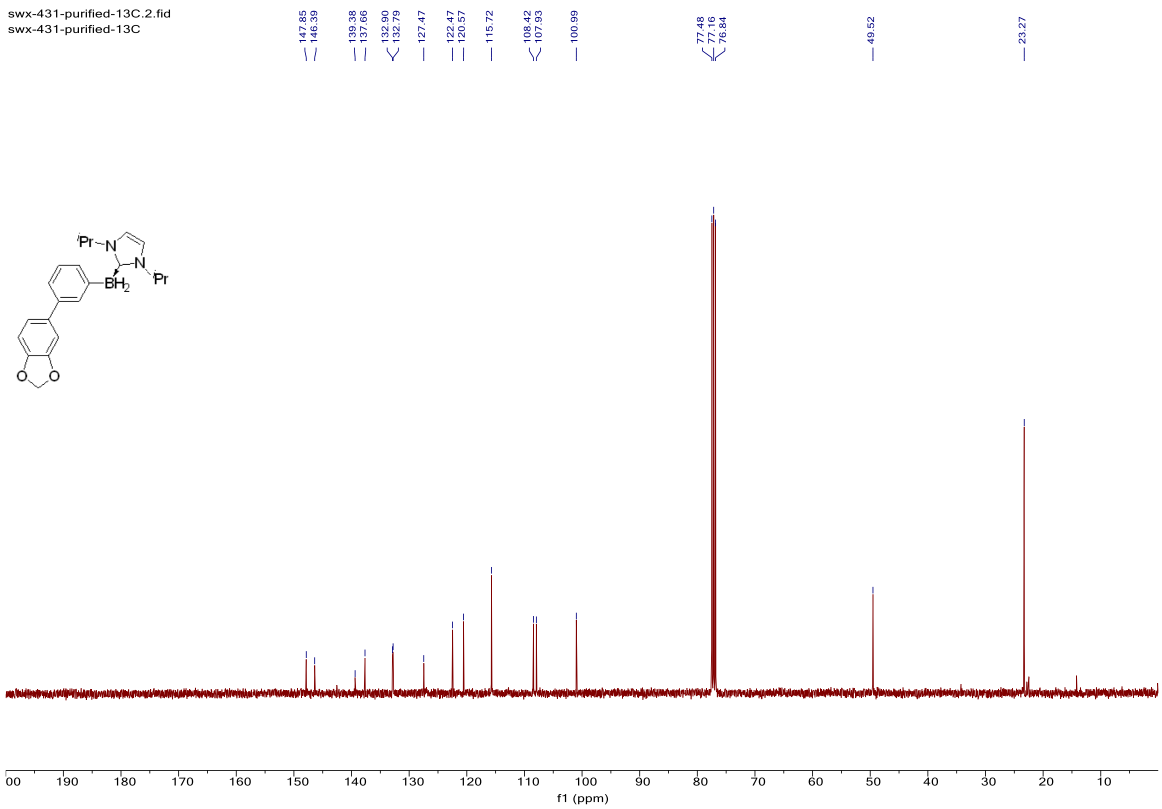

^{13}C NMR spectrum of **4i** in $CDCl_3$, 101 MHz.

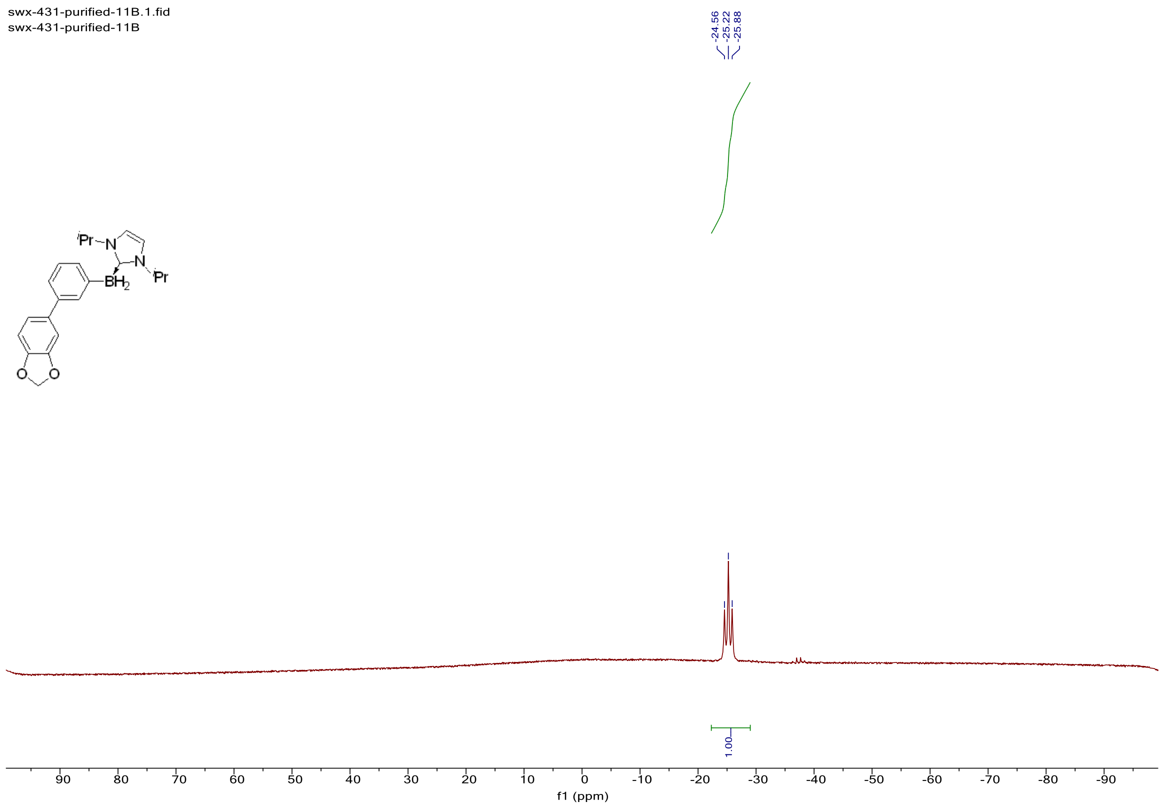

^{11}B NMR spectrum of **4i** in $CDCl_3$, 128 MHz.

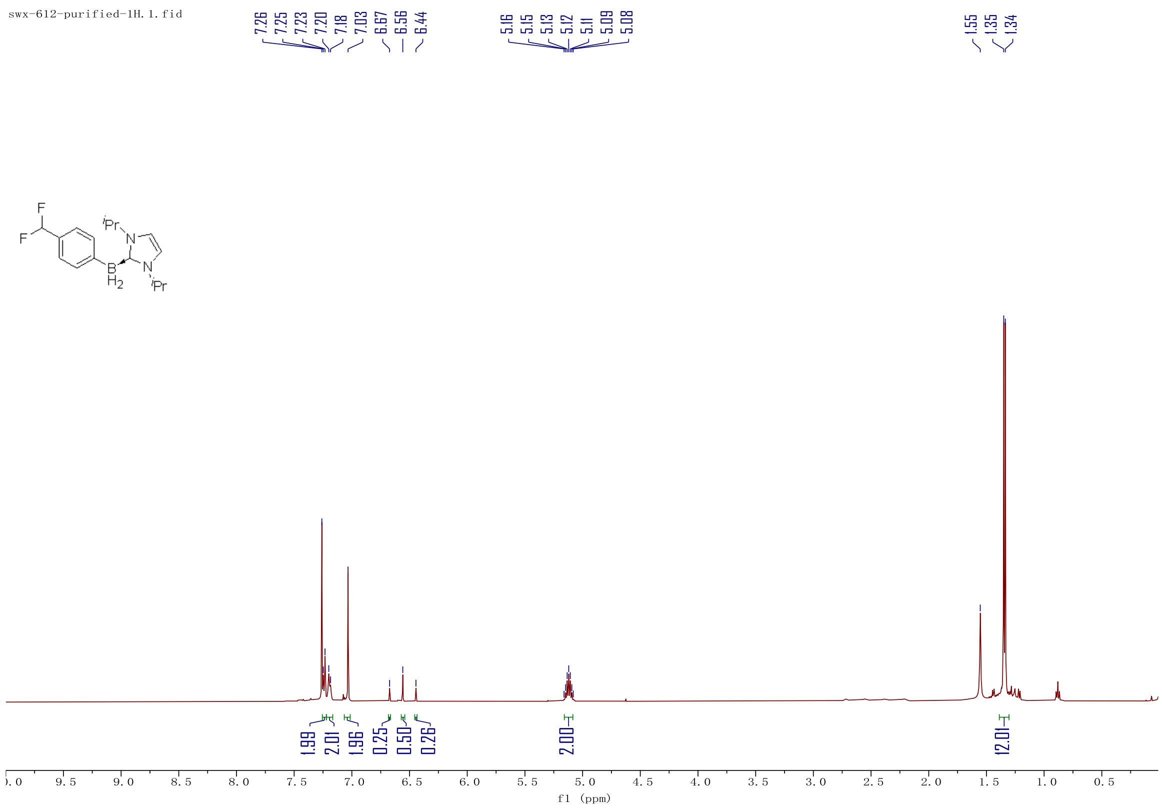

^{1}H NMR spectrum of **4j** in $CDCl_3$, 500 MHz.

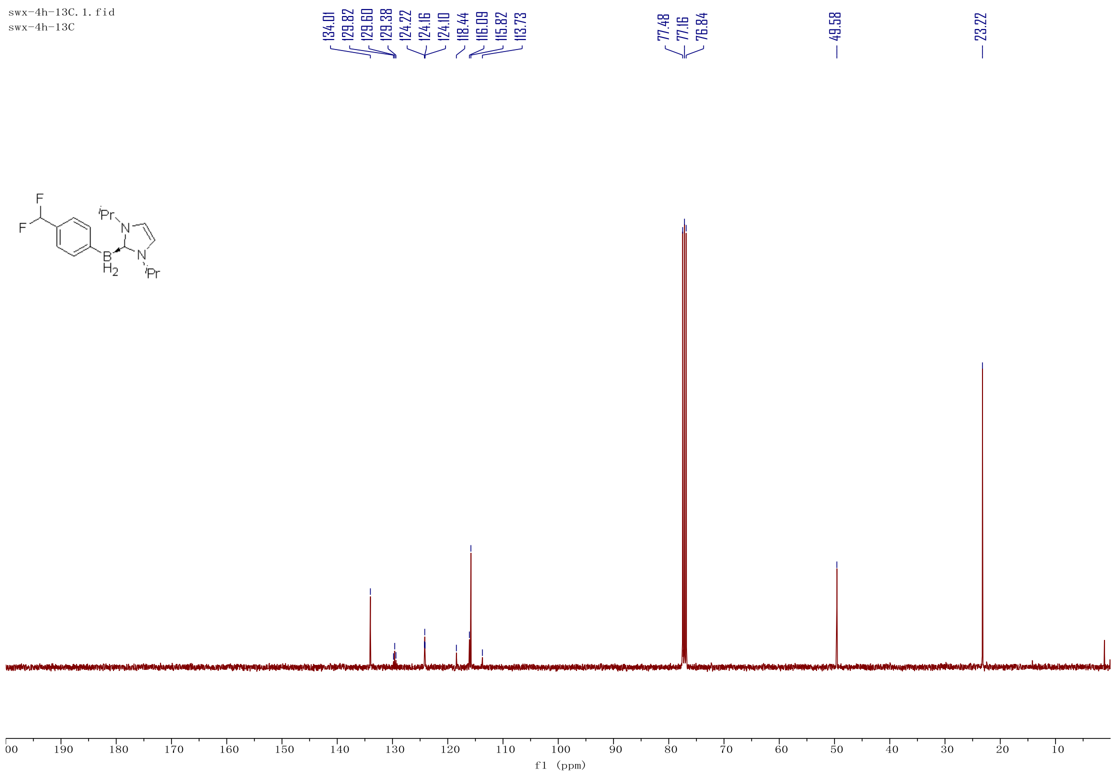

^{13}C NMR spectrum of **4j** in $CDCl_3$, 101 MHz.

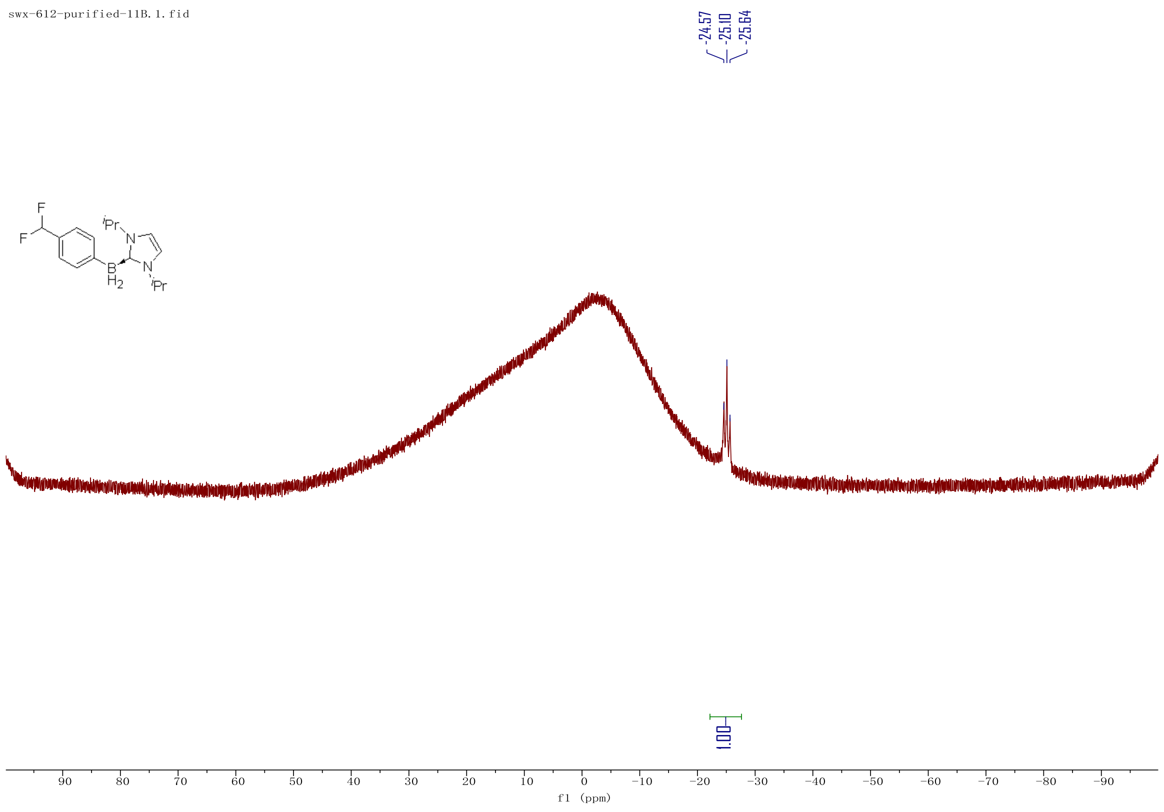

^{11}B NMR spectrum of **4j** in $CDCl_3$, 160 MHz.

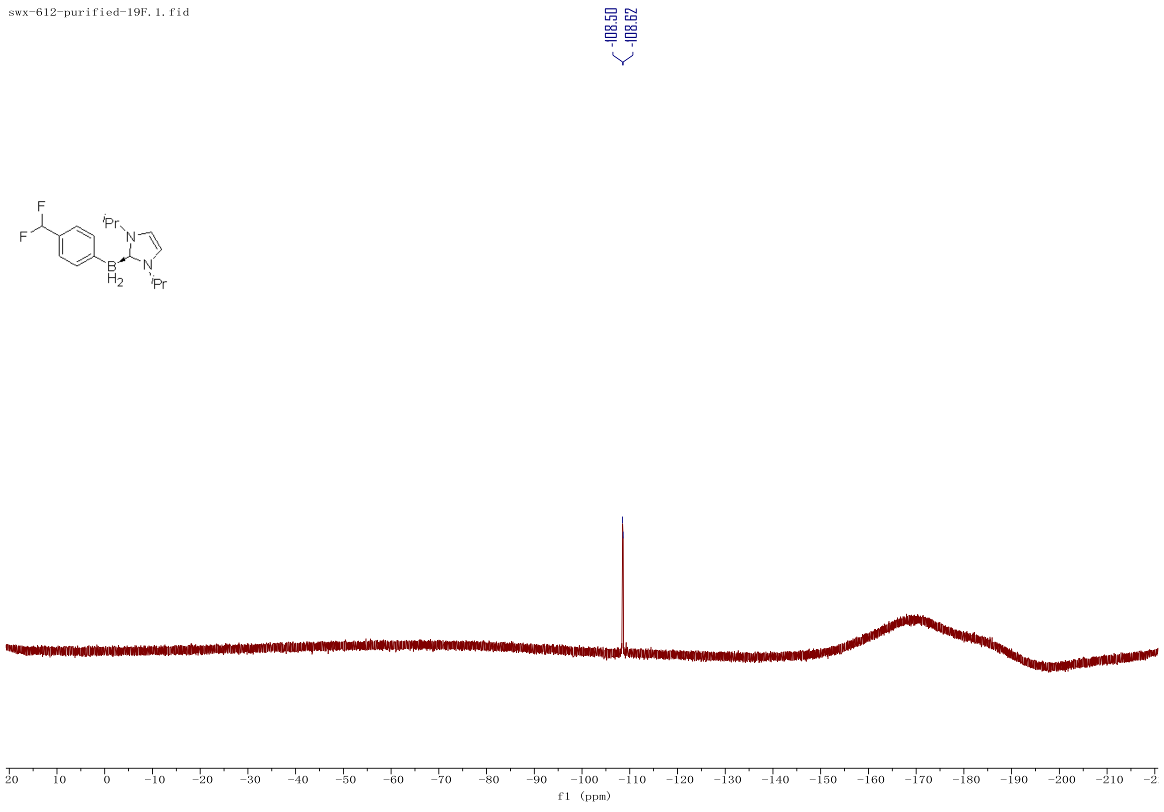

^{19}F NMR spectrum of **4j** in $CDCl_3$, 471 MHz.

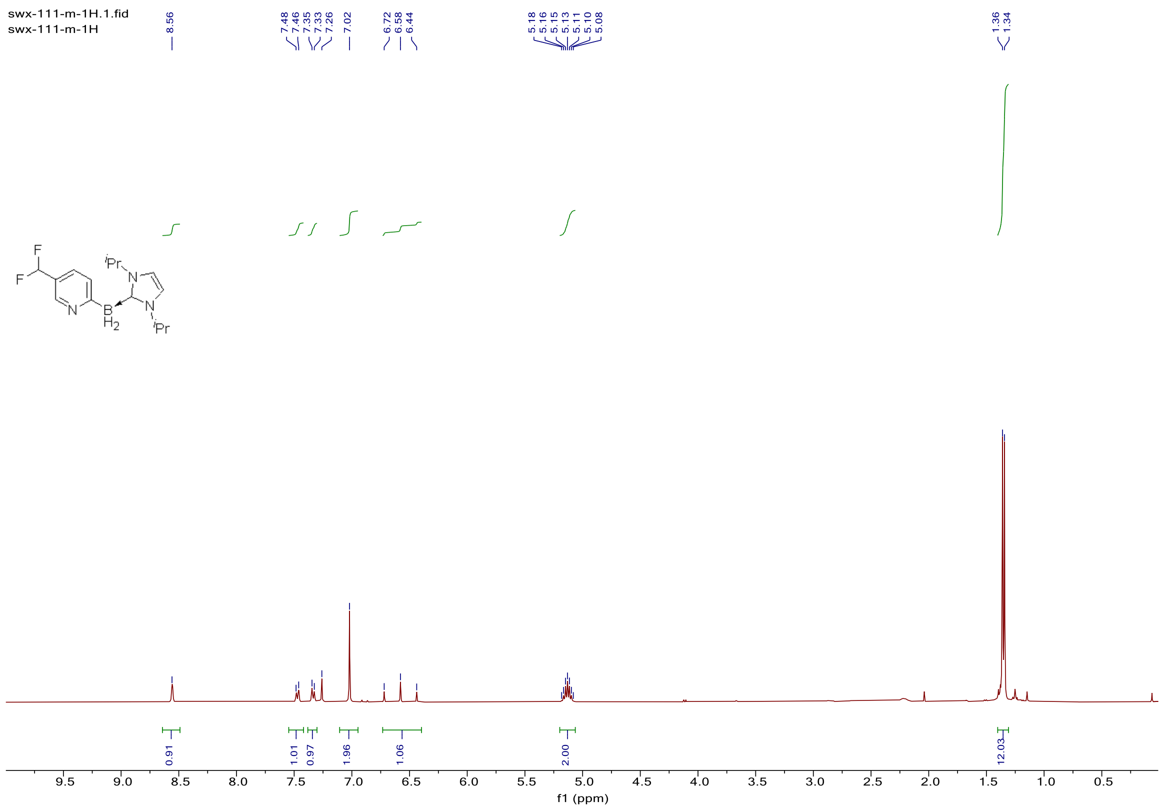

^{1}H NMR spectrum of **4k** in $CDCl_3$, 400 MHz.

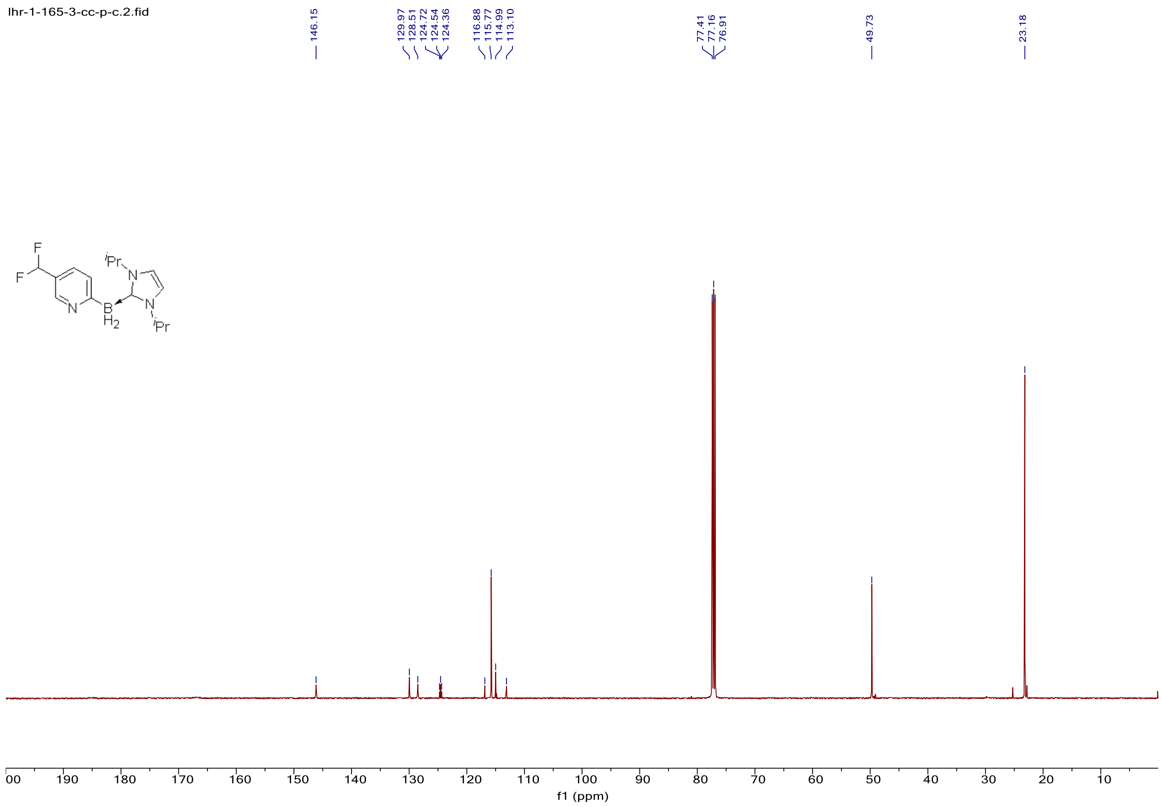

^{13}C NMR spectrum of **4k** in $CDCl_3$, 126 MHz.

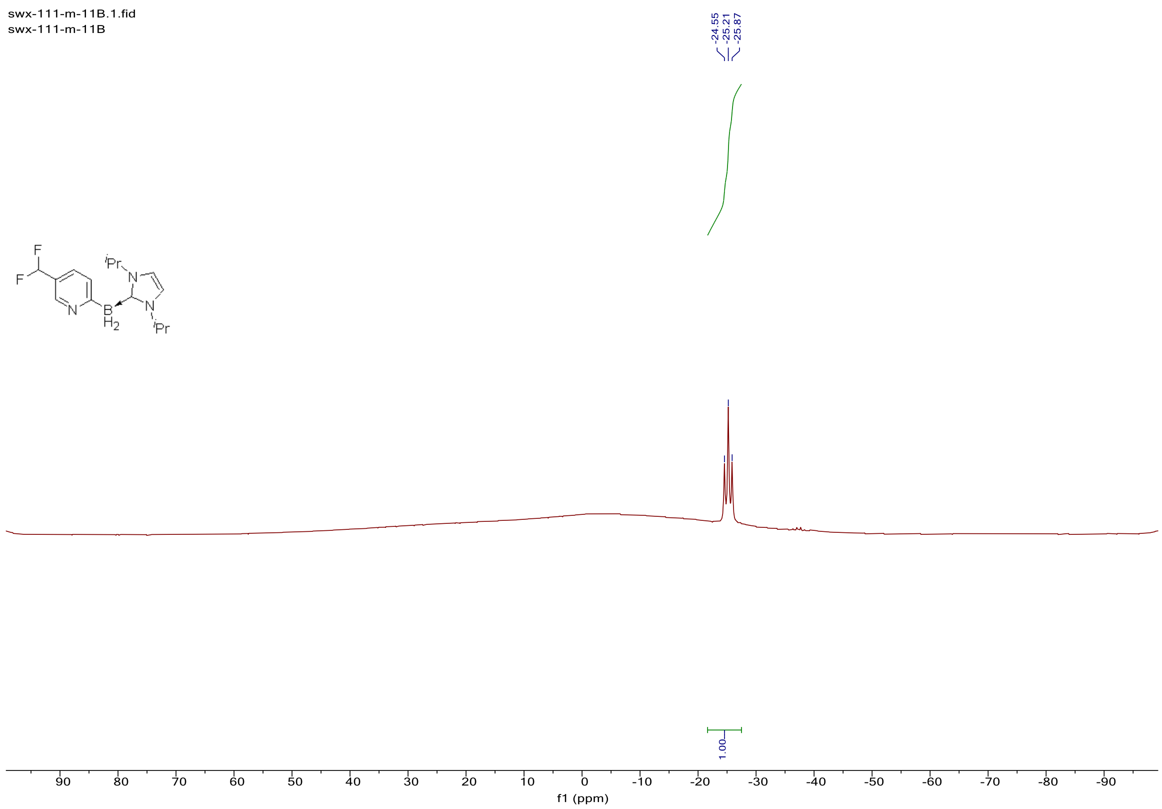

^{11}B NMR spectrum of **4k** in $CDCl_3$, 128 MHz.

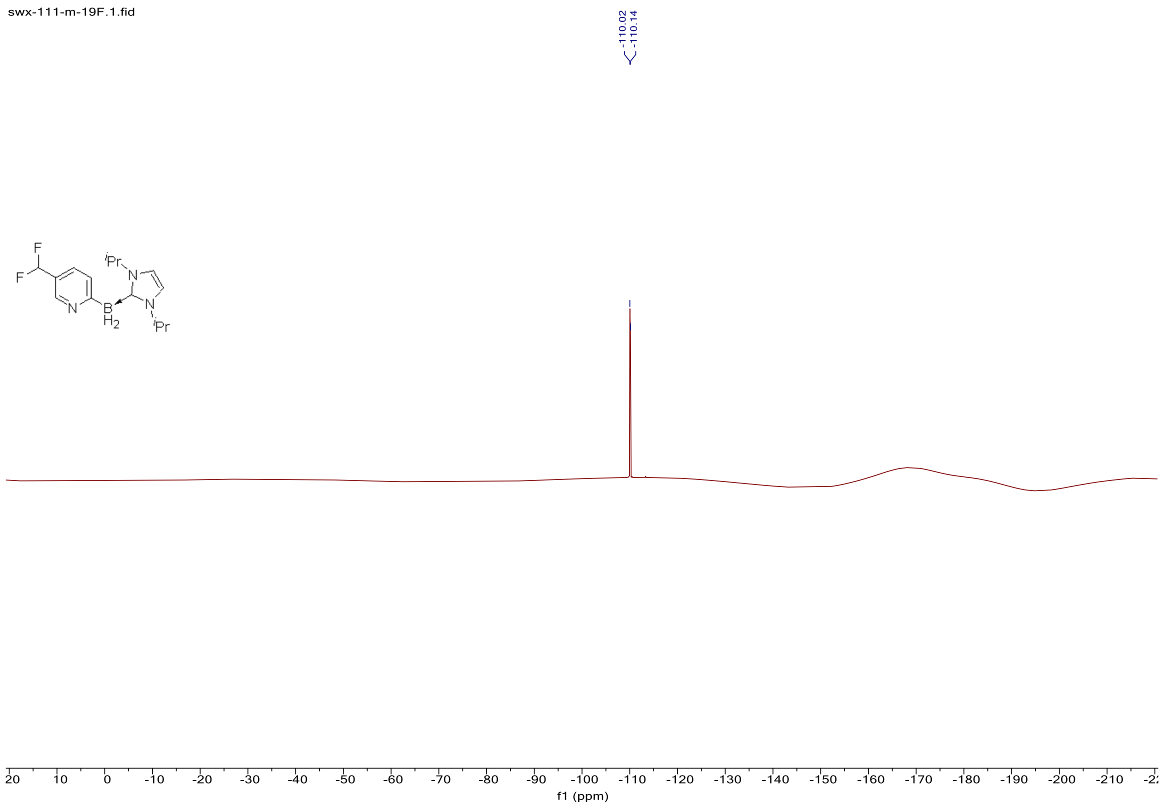

^{19}F NMR spectrum of **4k** in $CDCl_3$, 471 MHz.

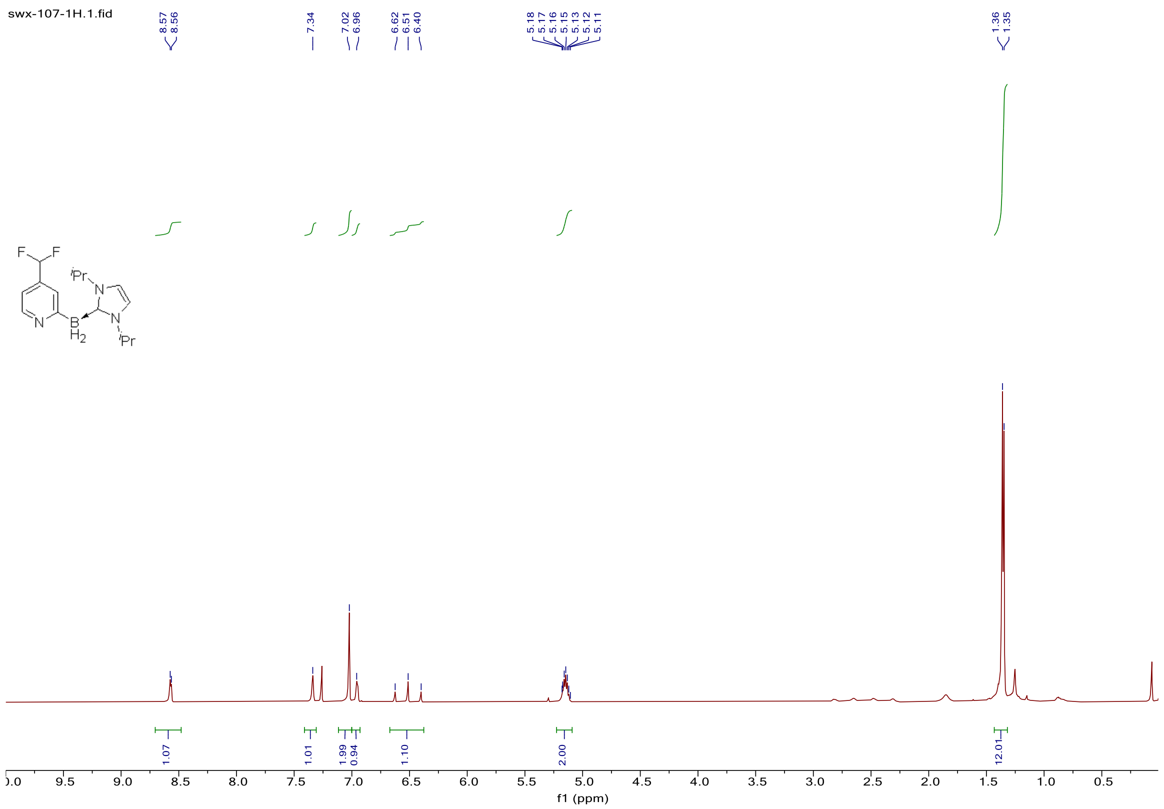

^{1}H NMR spectrum of **4l** in $CDCl_3$, 500 MHz.

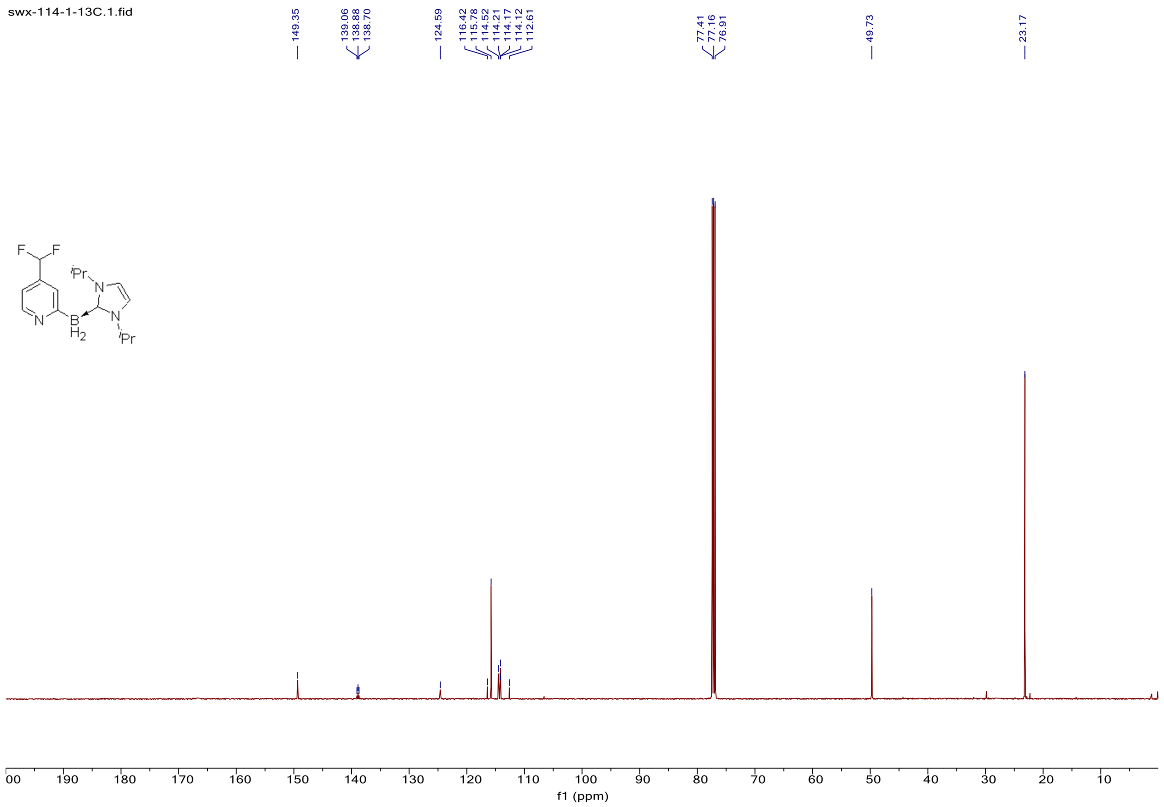

^{13}C NMR spectrum of **4l** in $CDCl_3$, 126 MHz.

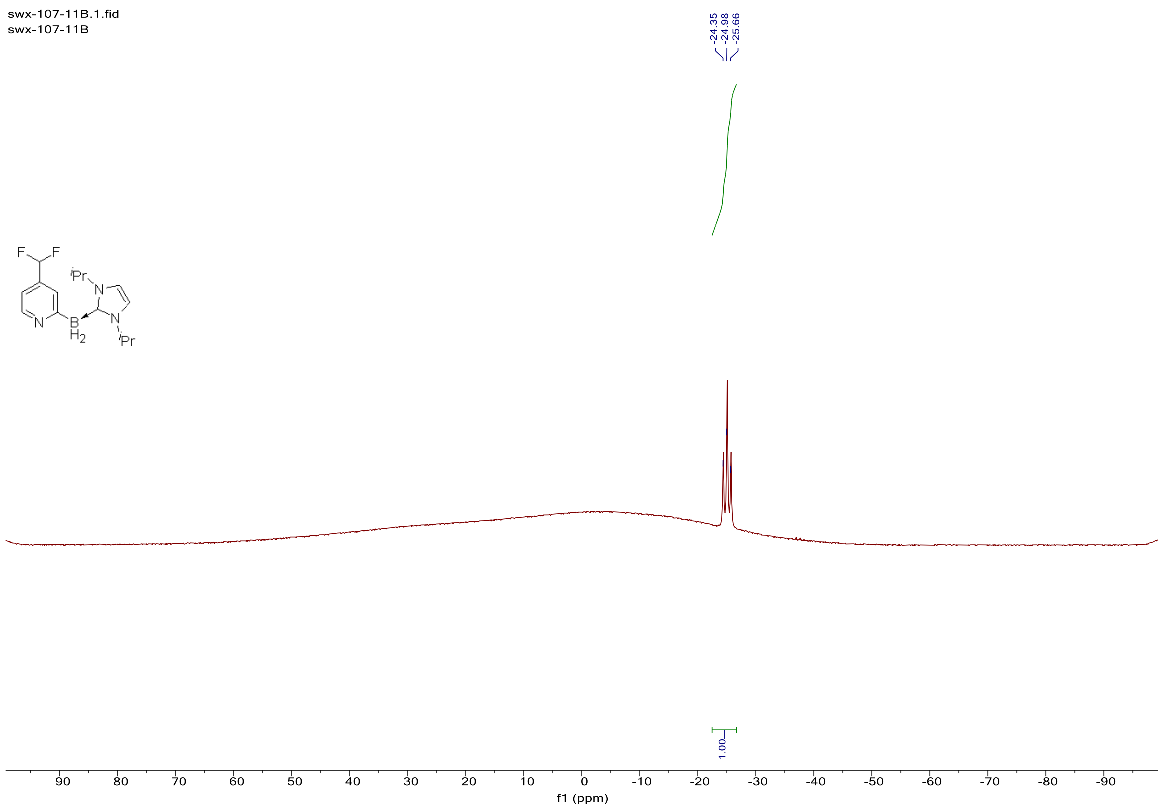

^{11}B NMR spectrum of **4l** in $CDCl_3$, 128 MHz.

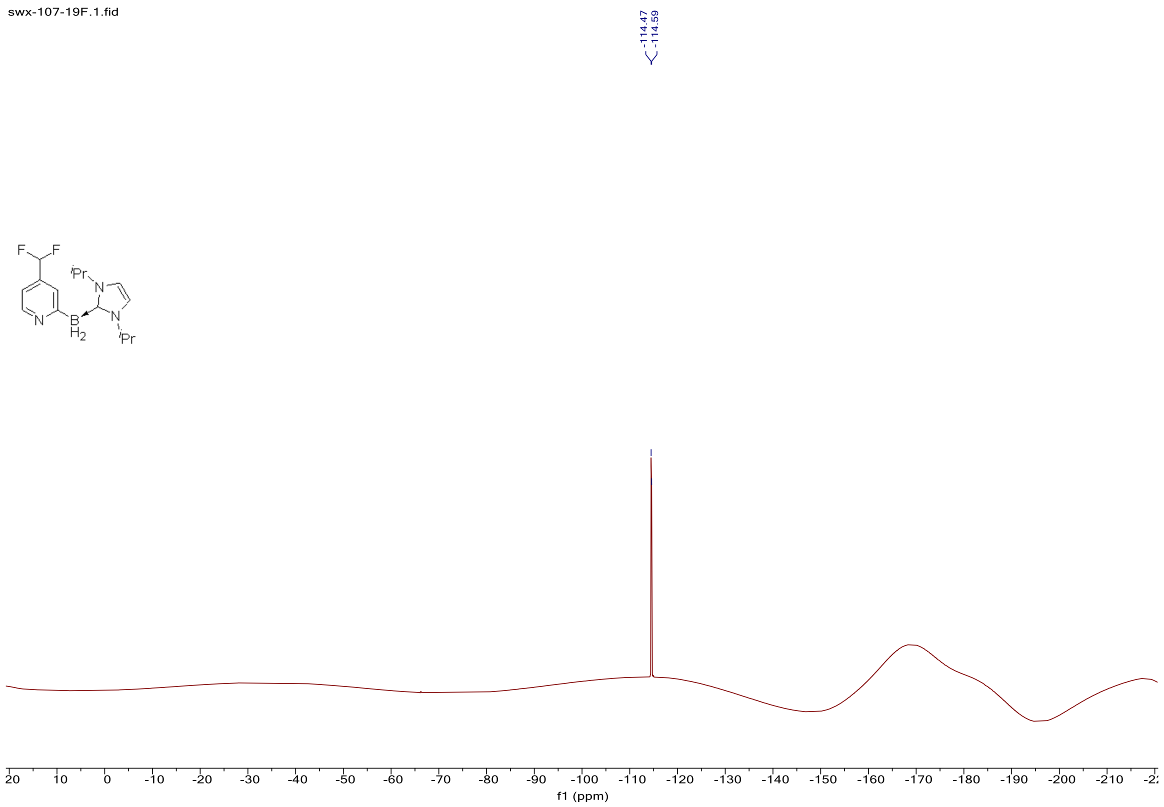

^{19}F NMR spectrum of **4l** in $CDCl_3$, 471 MHz.

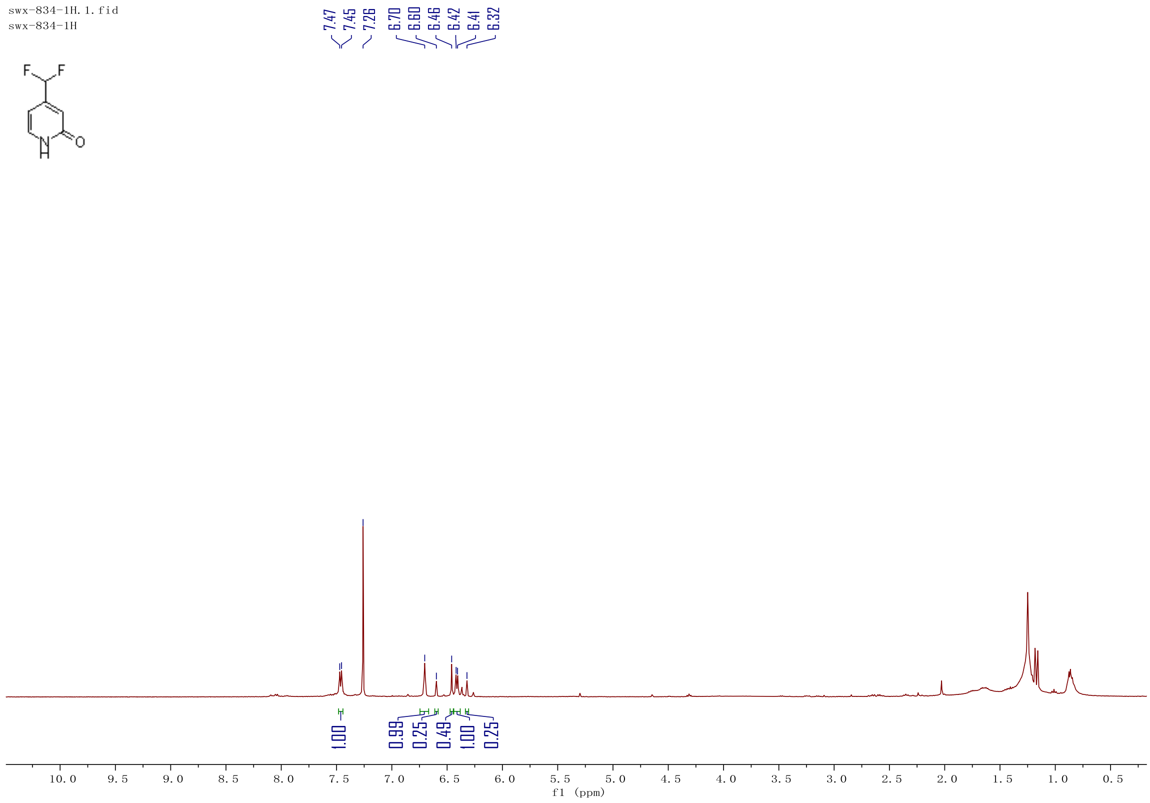

^{1}H NMR spectrum of **7** in $CDCl_3$, 400 MHz.

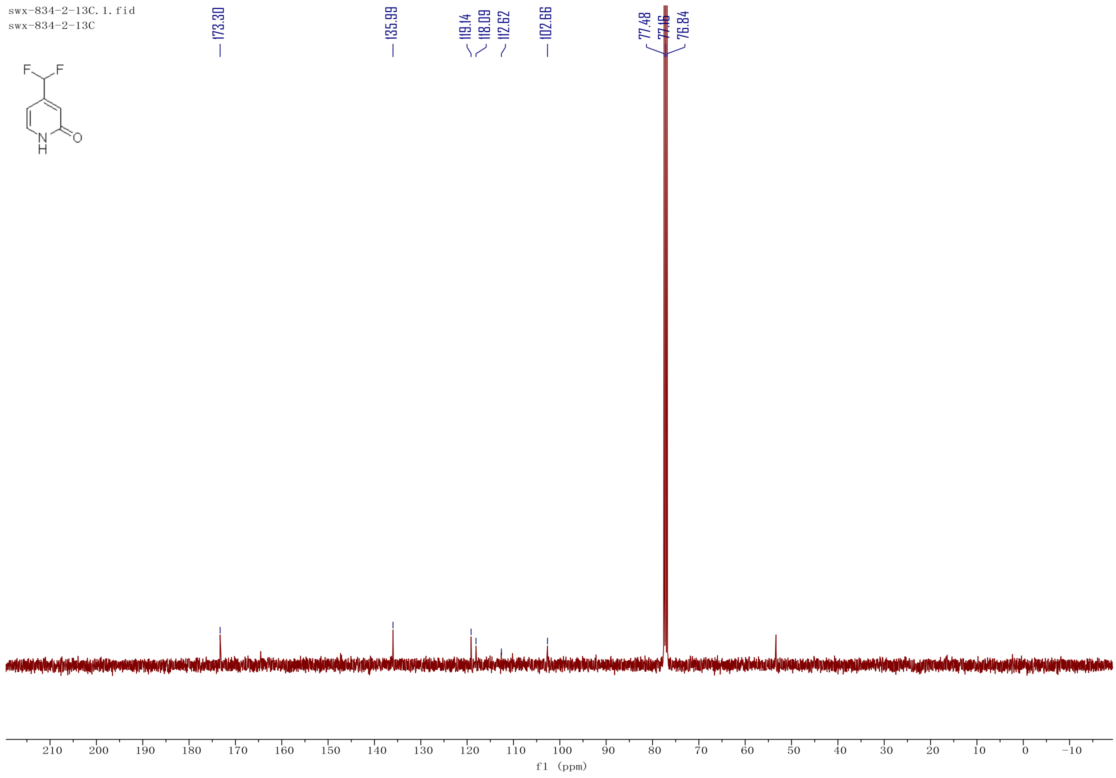

^{13}C NMR spectrum of **7** in $CDCl_3$, 101 MHz.

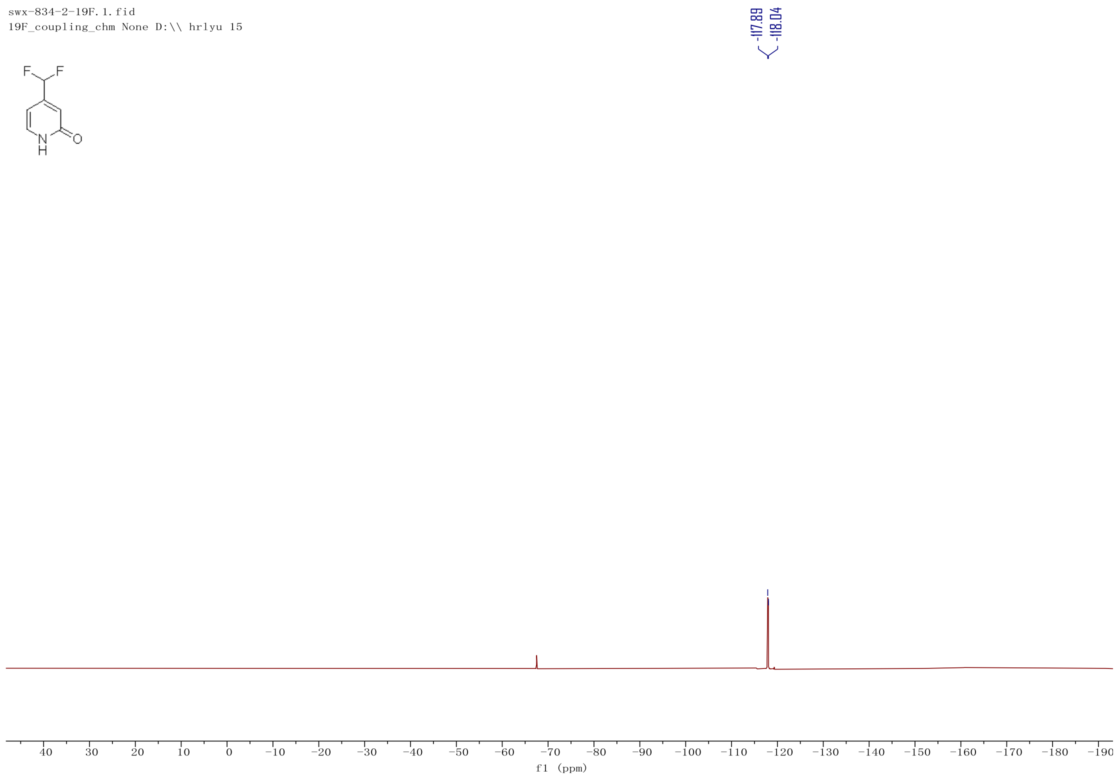

^{19}F NMR spectrum of **7** in $CDCl_3$, 377 MHz.

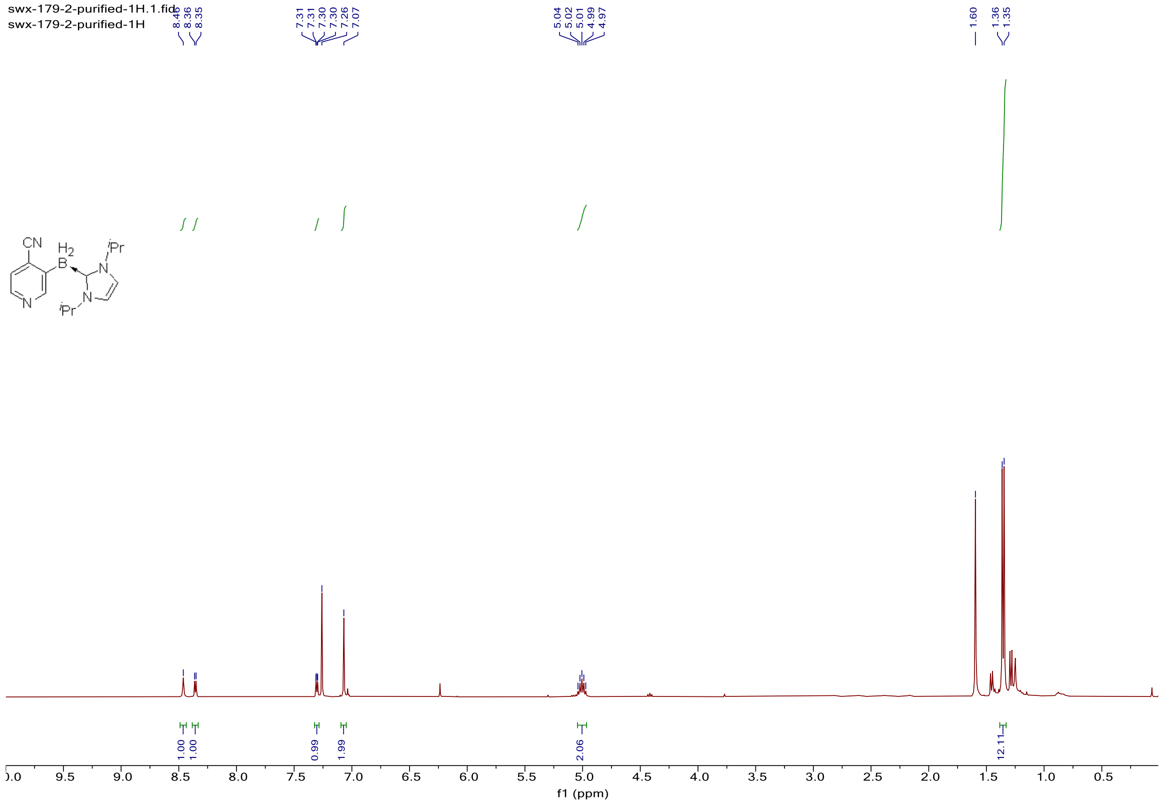

^{1}H NMR spectrum of **4m** in $CDCl_3$, 400 MHz.

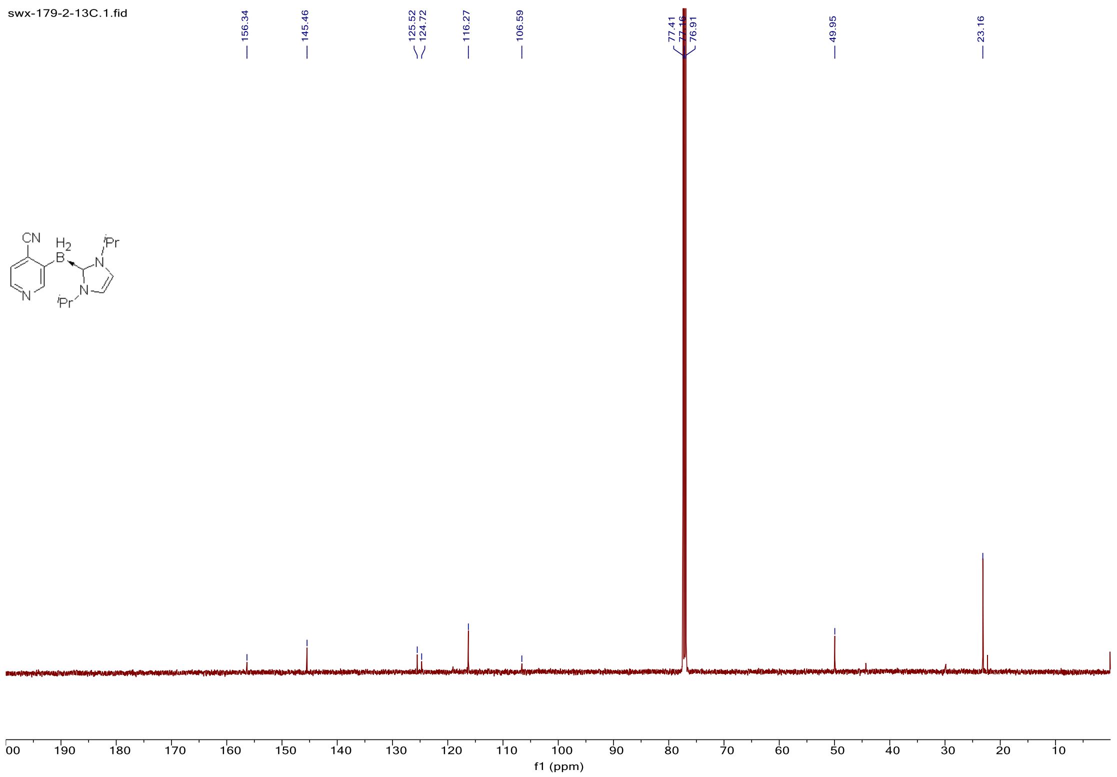

^{13}C NMR spectrum of **4m** in $CDCl_3$, 126 MHz.

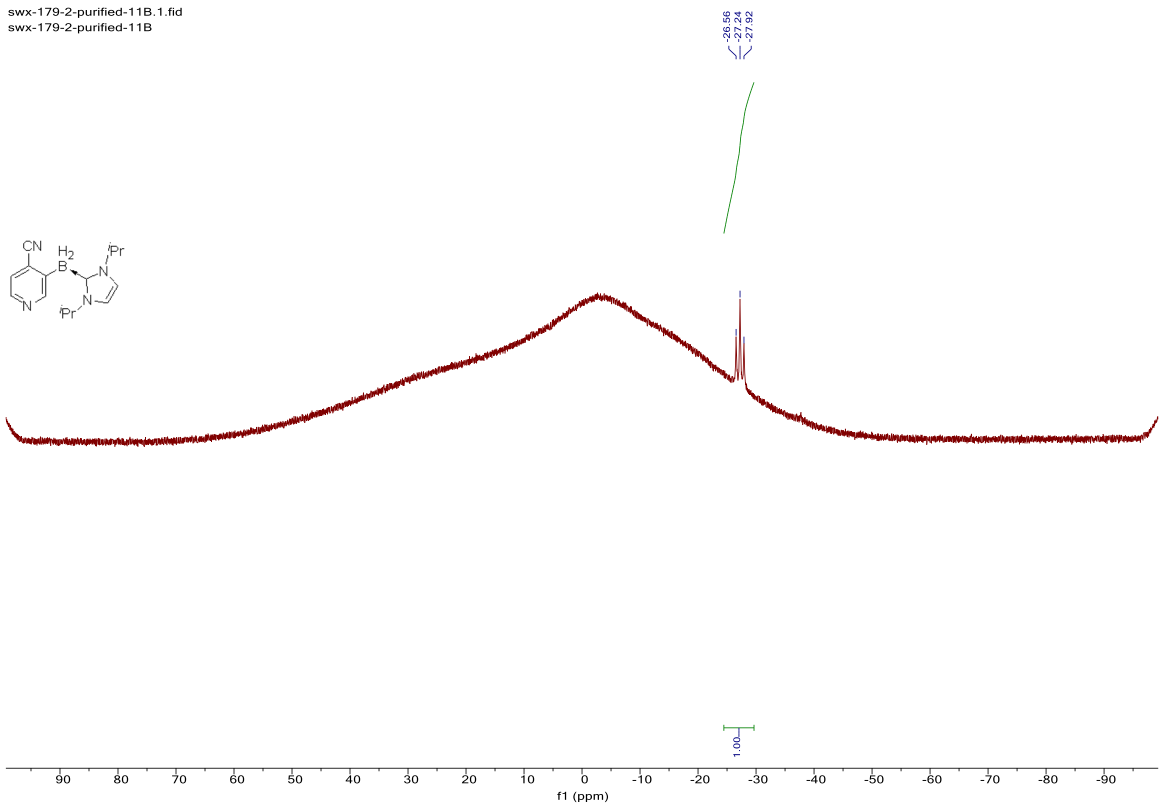

^{11}B NMR spectrum of **4m** in $CDCl_3$, 128 MHz.

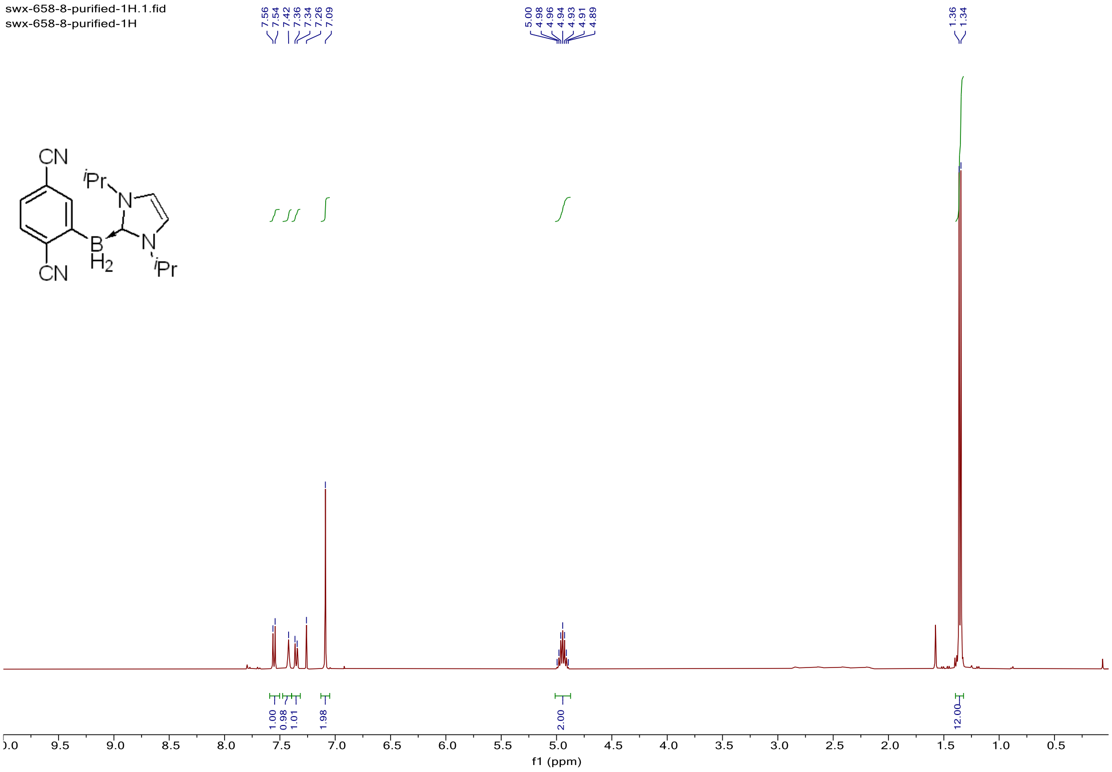

^{1}H NMR spectrum of **4n** in $CDCl_3$, 400 MHz.

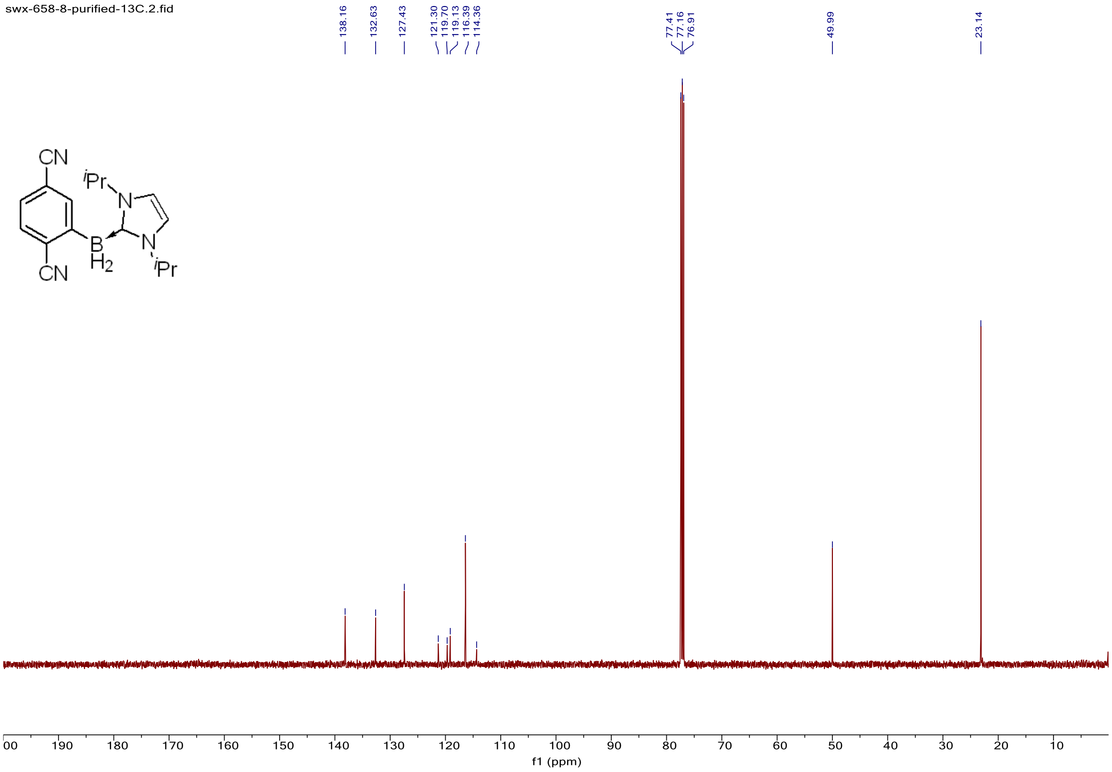

^{13}C NMR spectrum of **4n** in $CDCl_3$, 126 MHz.

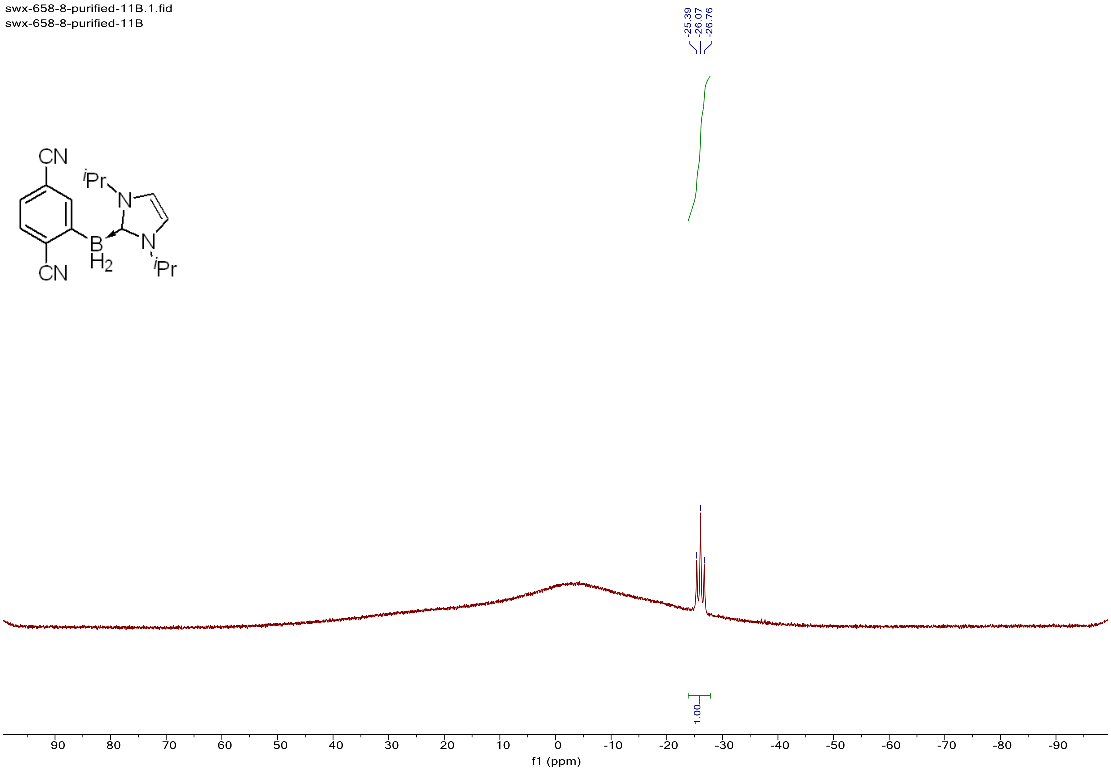

^{11}B NMR spectrum of **4n** in $CDCl_3$, 128 MHz.

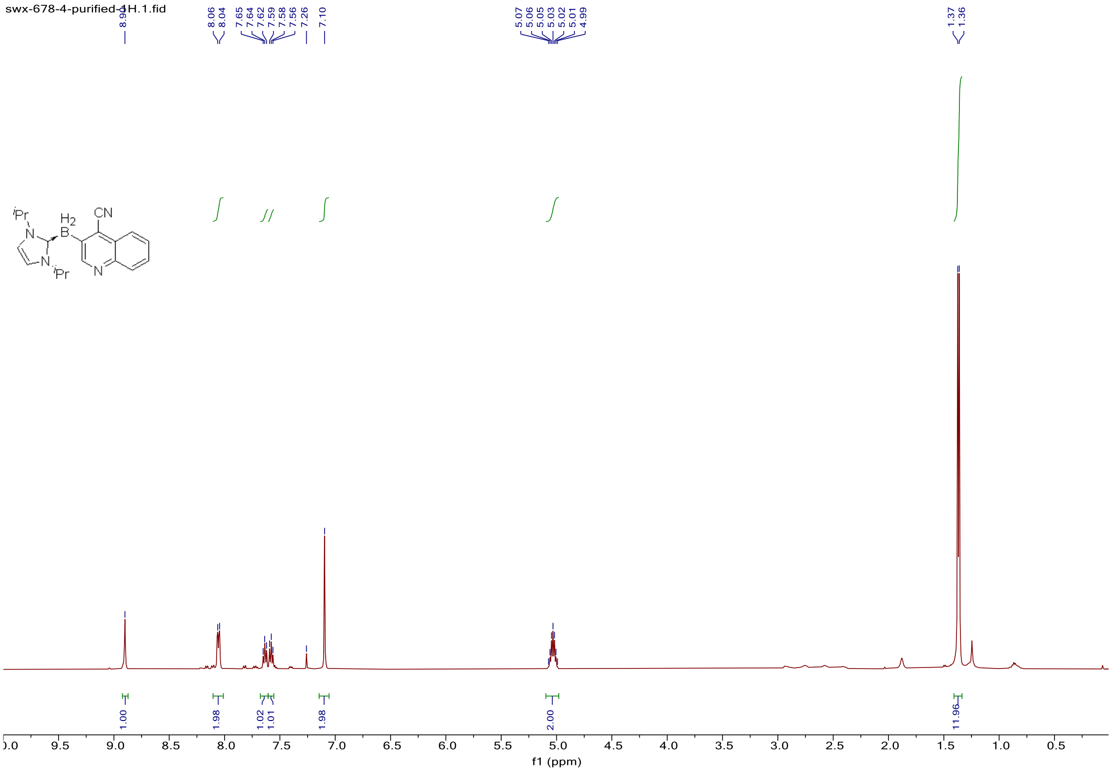

^{1}H NMR spectrum of **4o** in $CDCl_3$, 500 MHz.

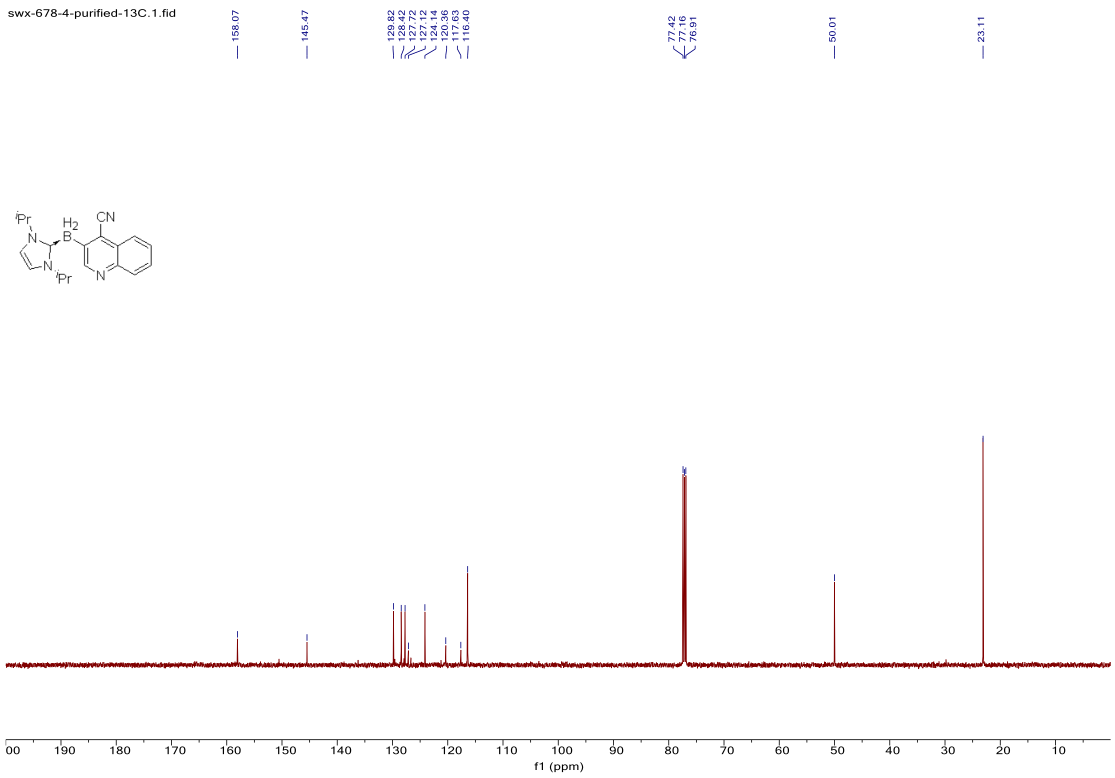

^{13}C NMR spectrum of **4o** in $CDCl_3$, 126 MHz.

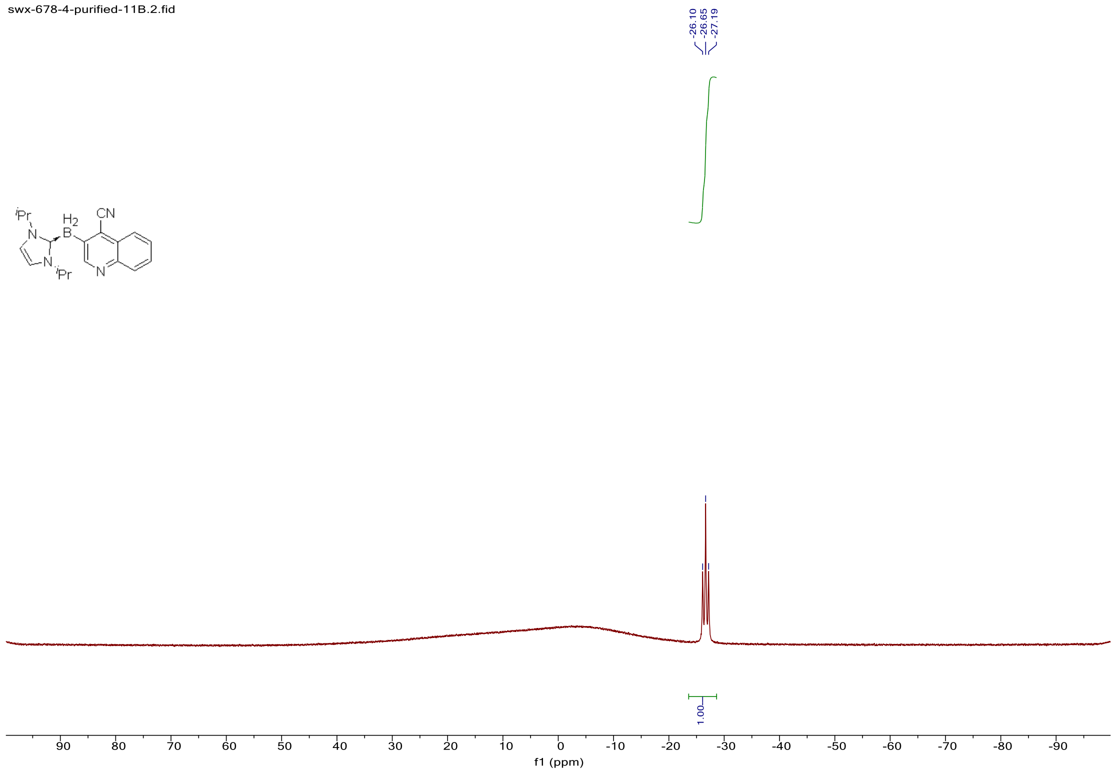

^{11}B NMR spectrum of **4o** in $CDCl_3$, 160 MHz.

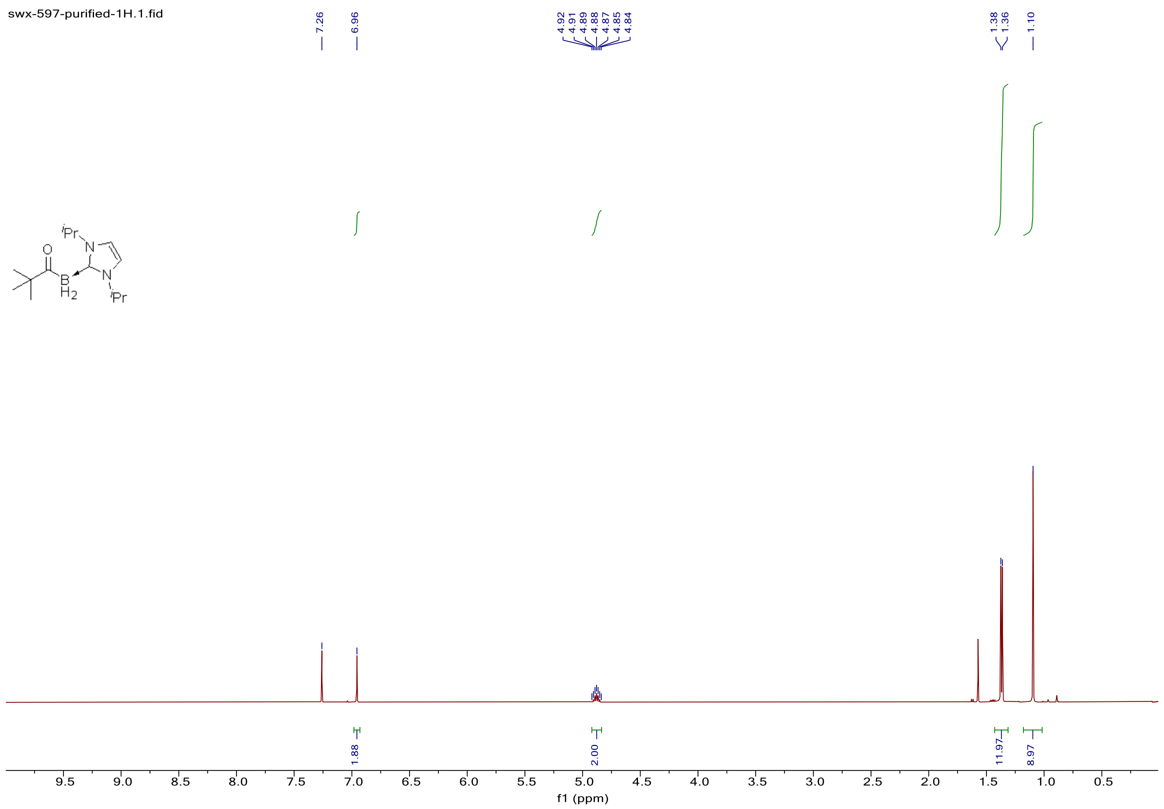

^{1}H NMR spectrum of **4p** in $CDCl_3$, 400 MHz.

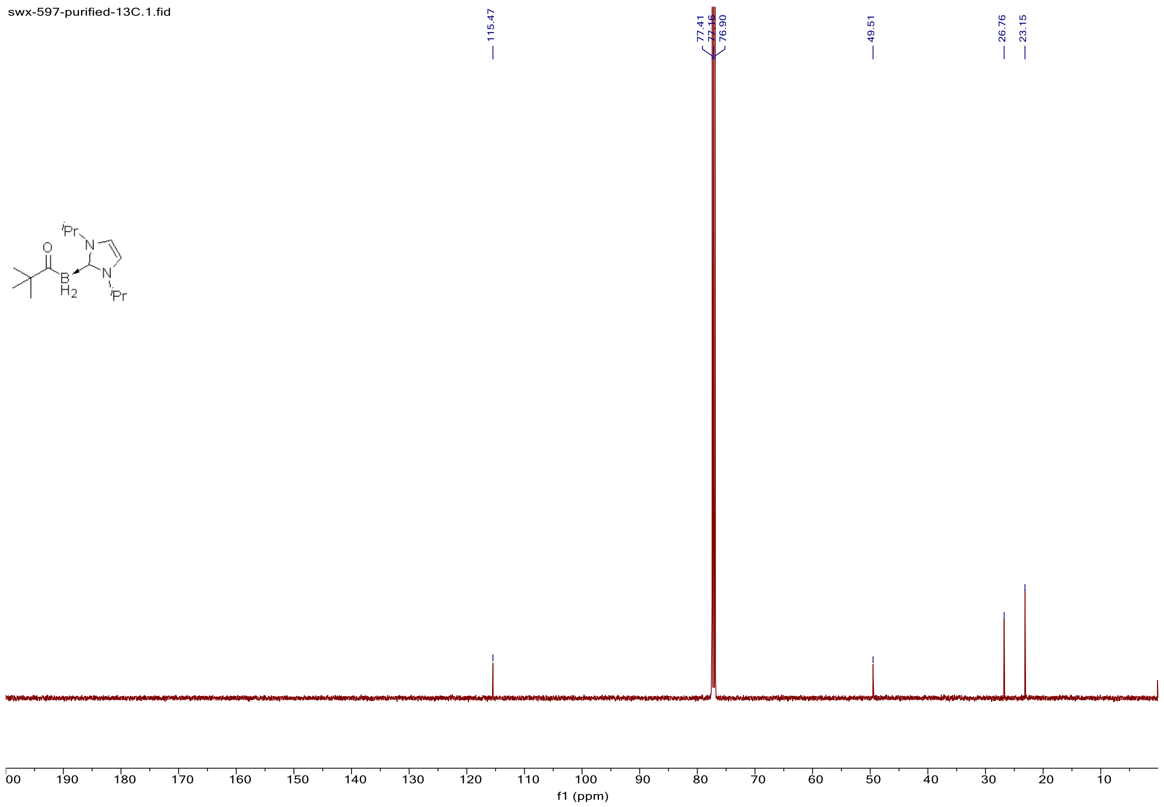

^{13}C NMR spectrum of **4p** in $CDCl_3$, 126 MHz.

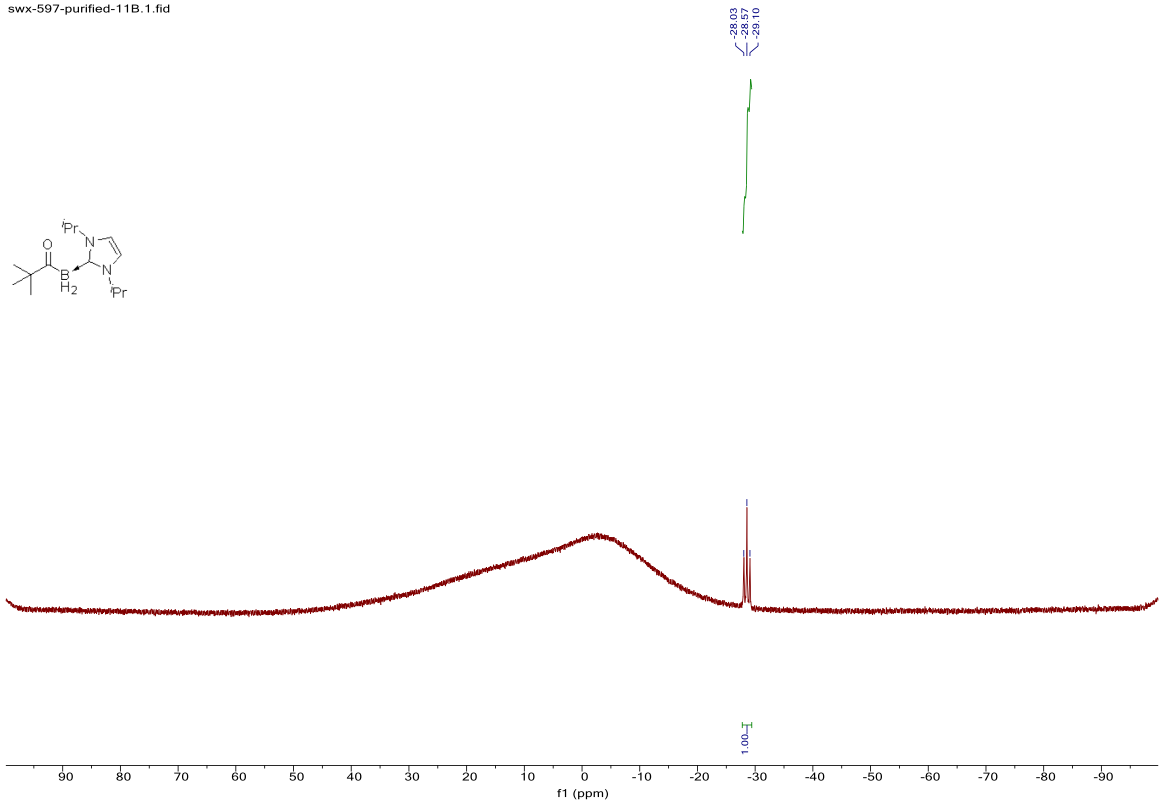

^{11}B NMR spectrum of **4p** in $CDCl_3$, 128 MHz.

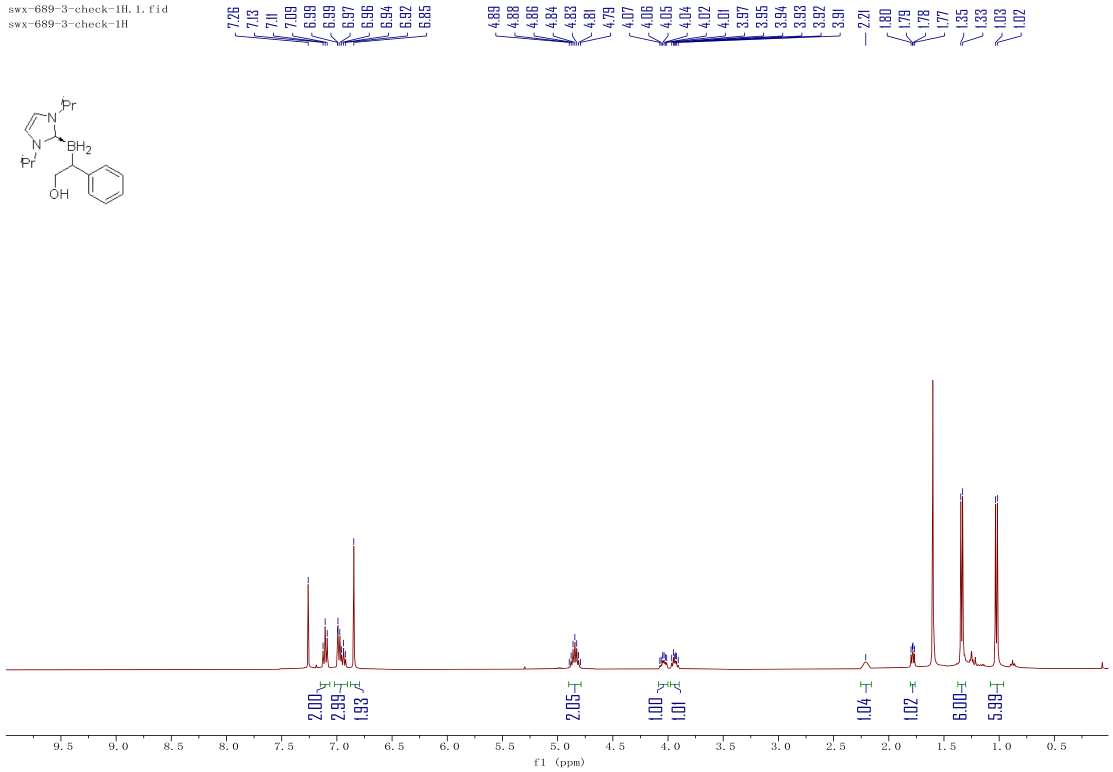

^{1}H NMR spectrum of **4q** in $CDCl_3$, 400 MHz.

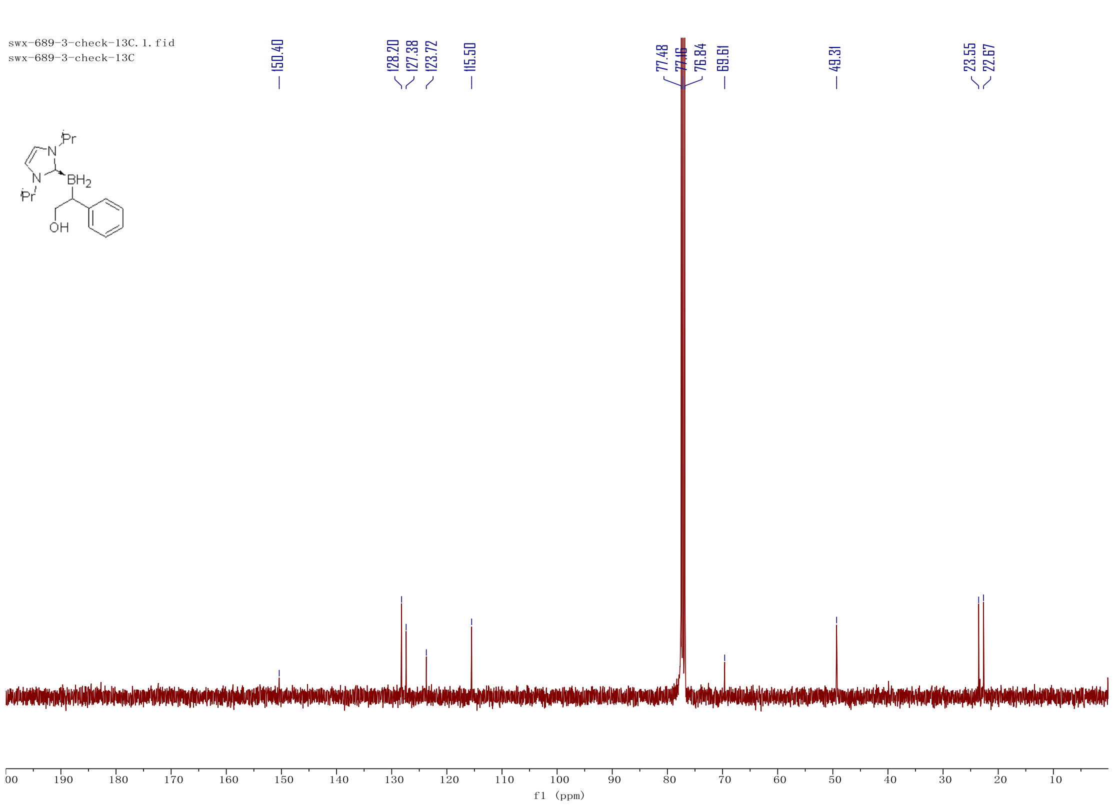

^{13}C NMR spectrum of **4q** in $CDCl_3$, 101 MHz.

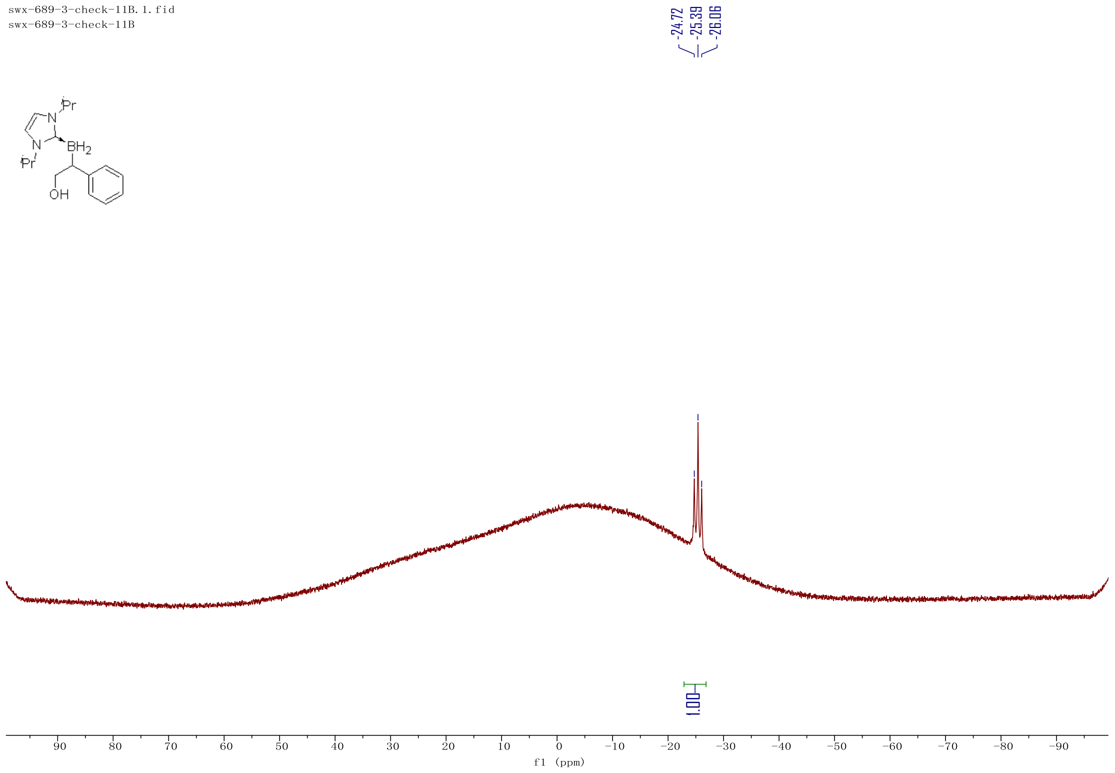

^{11}B NMR spectrum of **4q** in $CDCl_3$, 128 MHz.

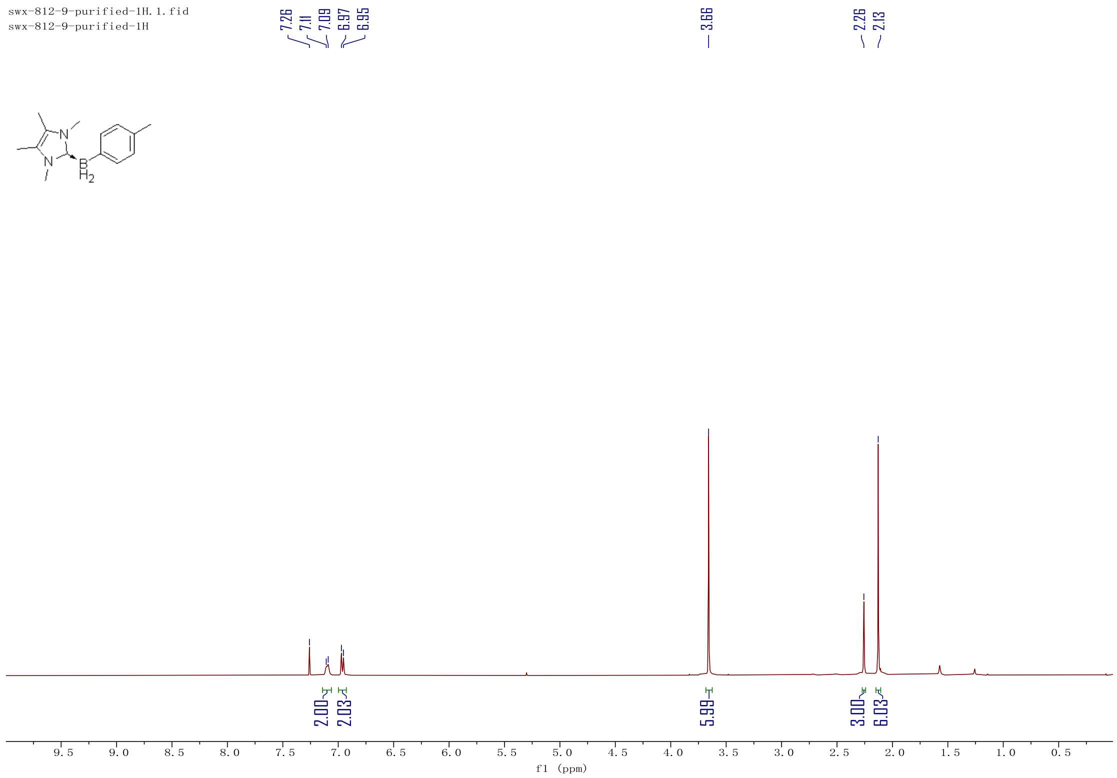

^{1}H NMR spectrum of **4r** in $CDCl_3$, 400 MHz.

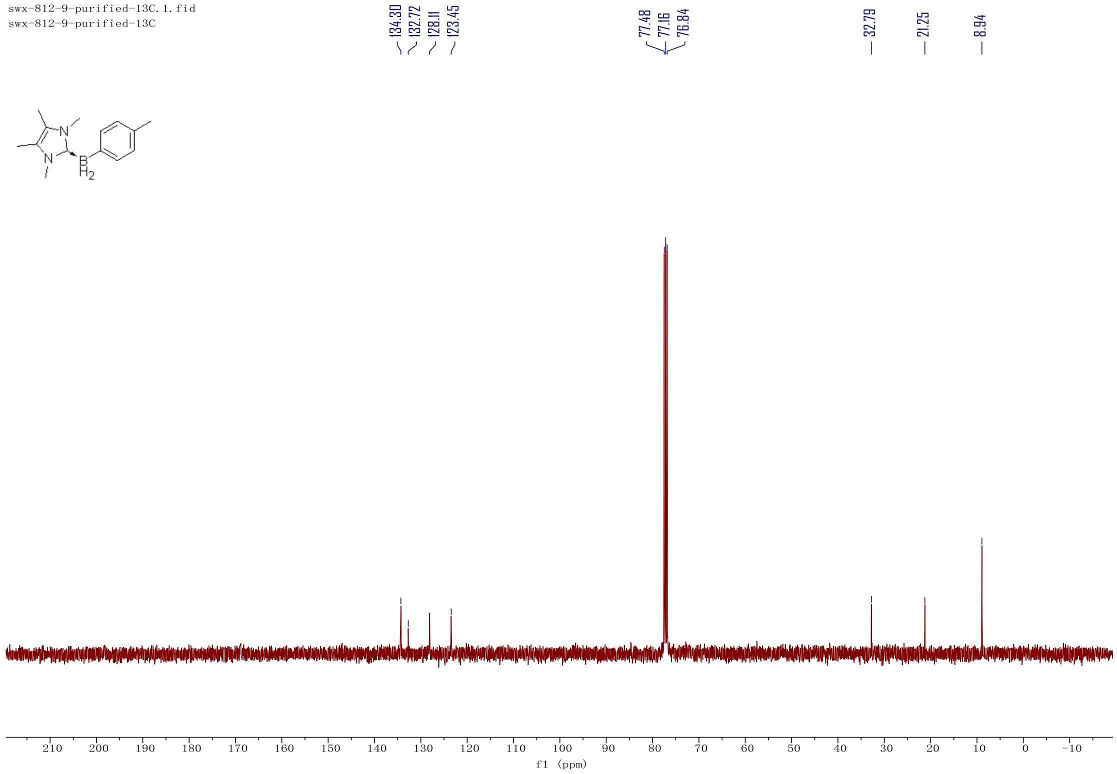

^{13}C NMR spectrum of **4r** in $CDCl_3$, 101 MHz.

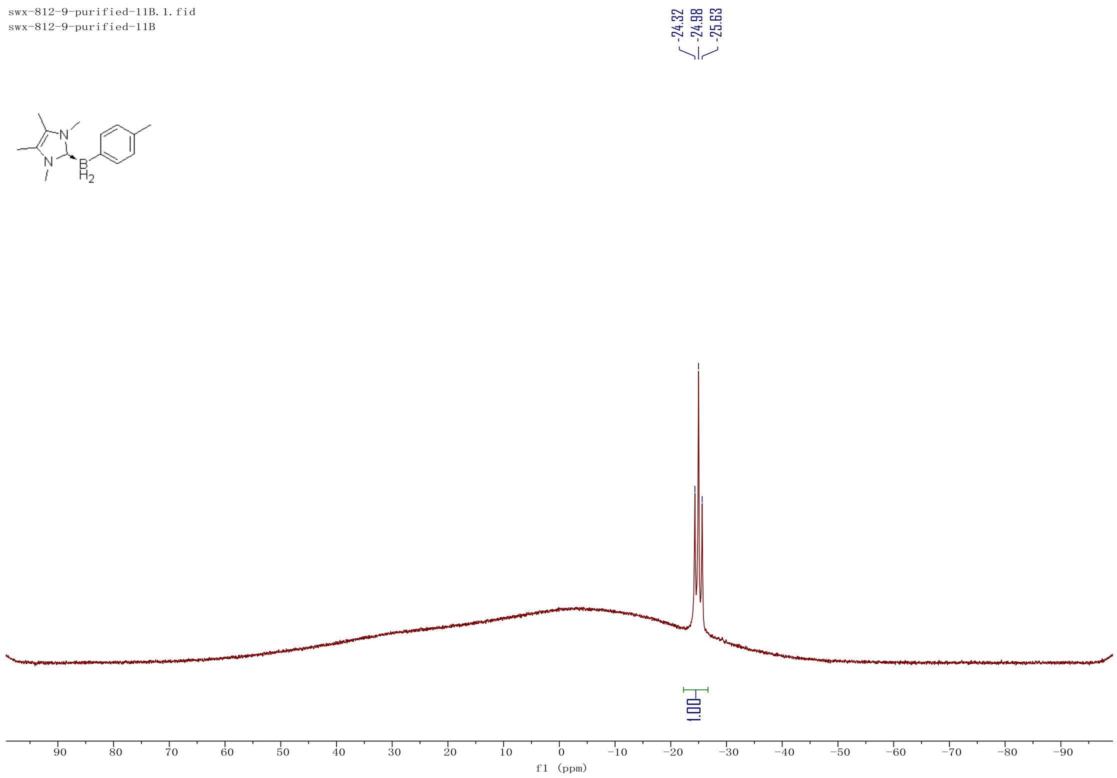

^{11}B NMR spectrum of **4r** in $CDCl_3$, 128 MHz.

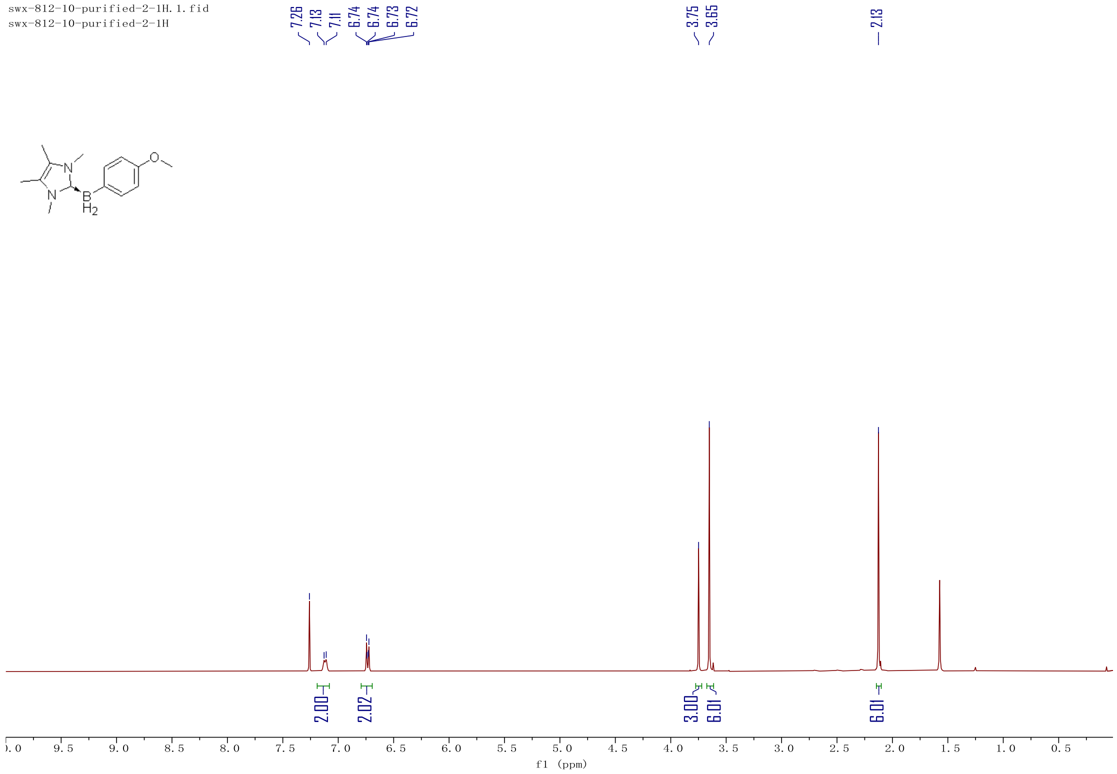

^{1}H NMR spectrum of **4s** in $CDCl_3$, 400 MHz.

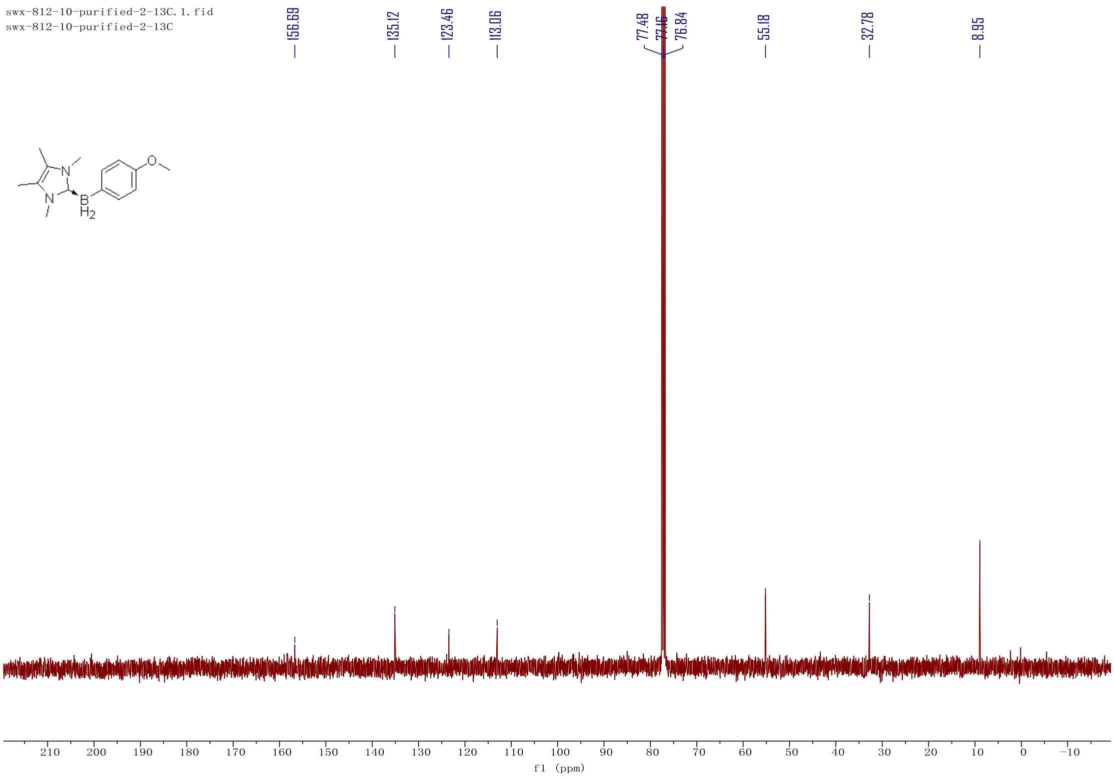

^{13}C NMR spectrum of **4s** in $CDCl_3$, 101 MHz.

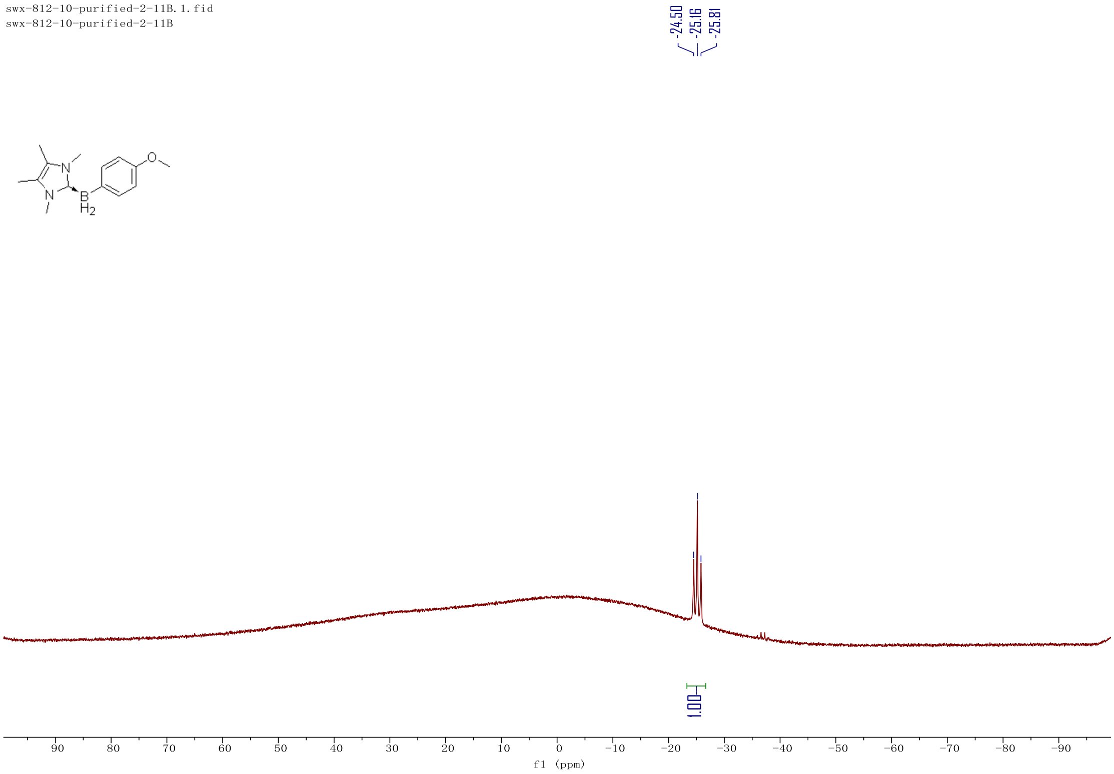

^{11}B NMR spectrum of **4s** in $CDCl_3$, 128 MHz.

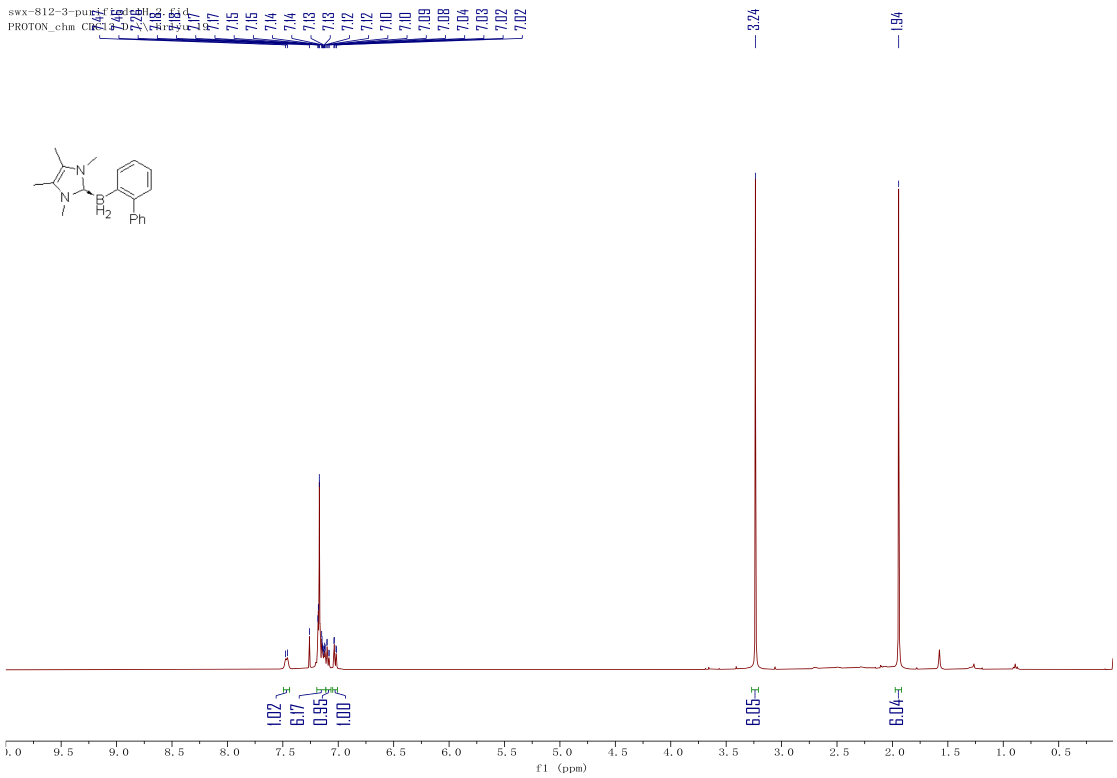

^{1}H NMR spectrum of **4t** in $CDCl_3$, 400 MHz.

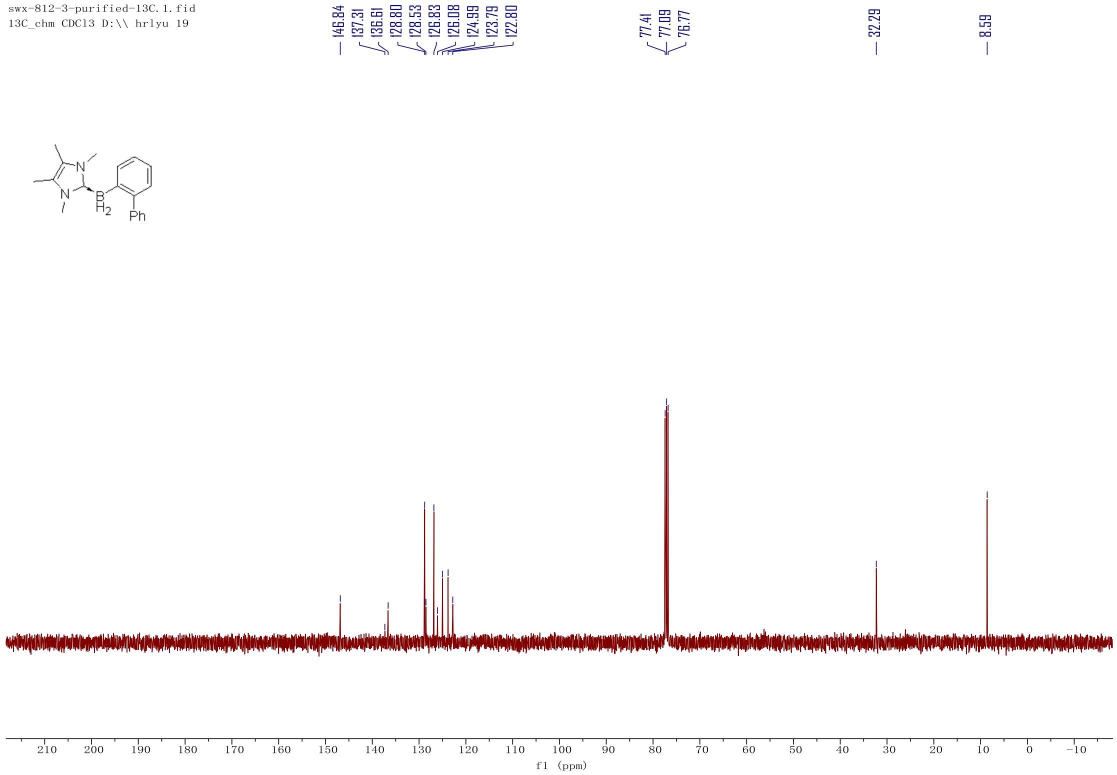

^{13}C NMR spectrum of **4t** in $CDCl_3$, 101 MHz.

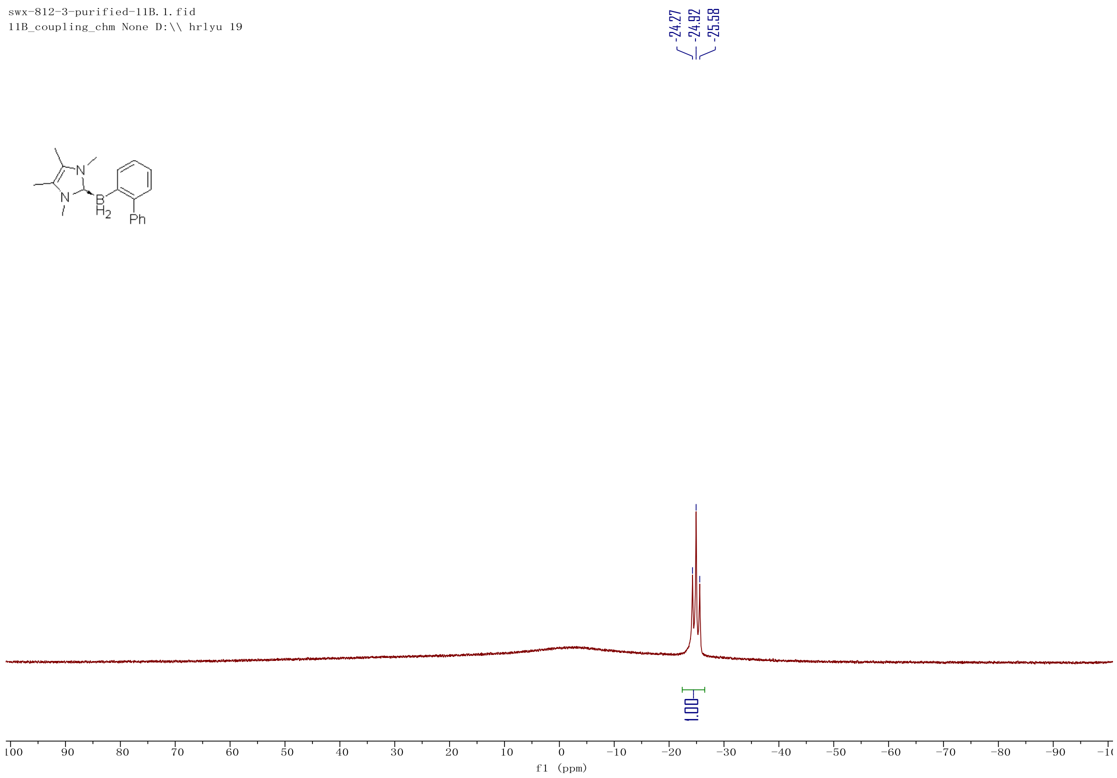

^{11}B NMR spectrum of **4t** in $CDCl_3$, 128 MHz.

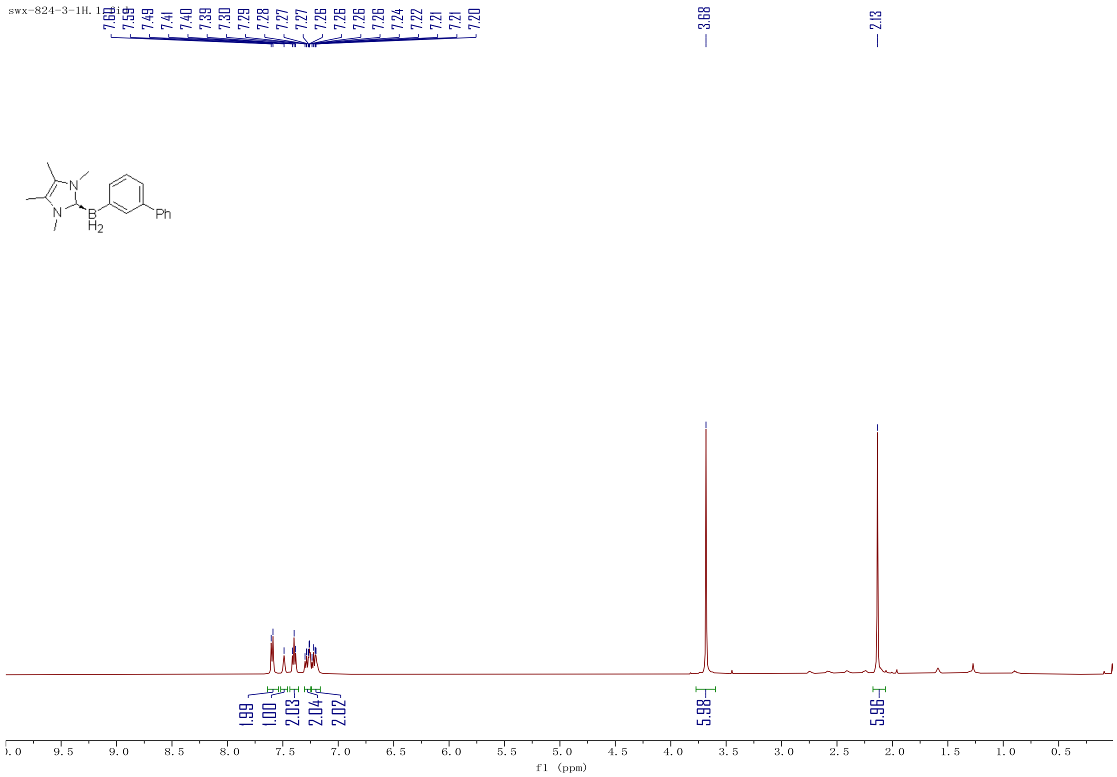

^{1}H NMR spectrum of **4u** in $CDCl_3$, 500 MHz.

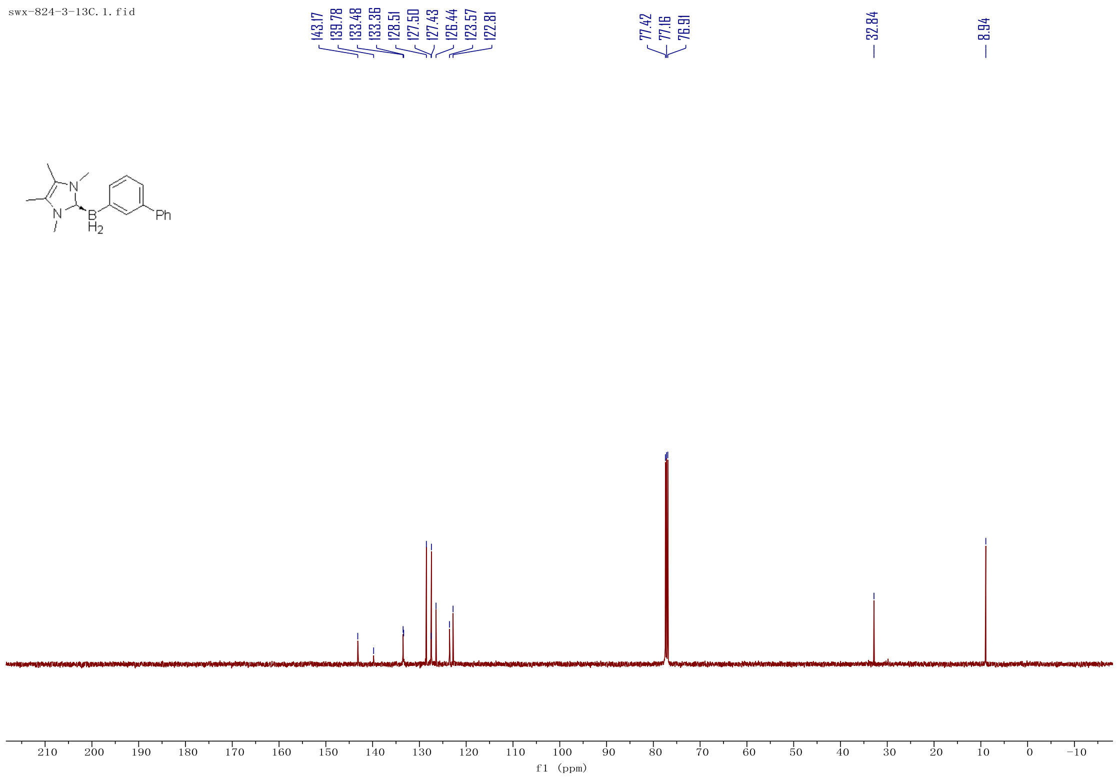

^{13}C NMR spectrum of **4u** in $CDCl_3$, 126 MHz.

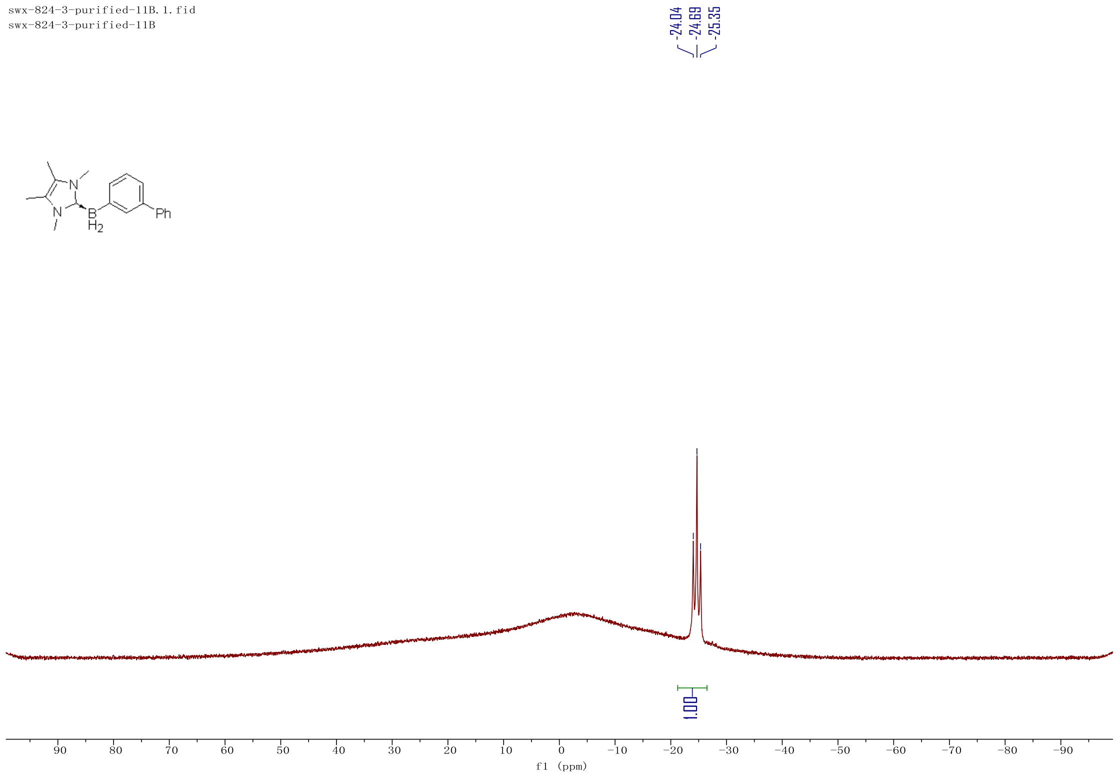

^{11}B NMR spectrum of **4u** in $CDCl_3$, 128 MHz.

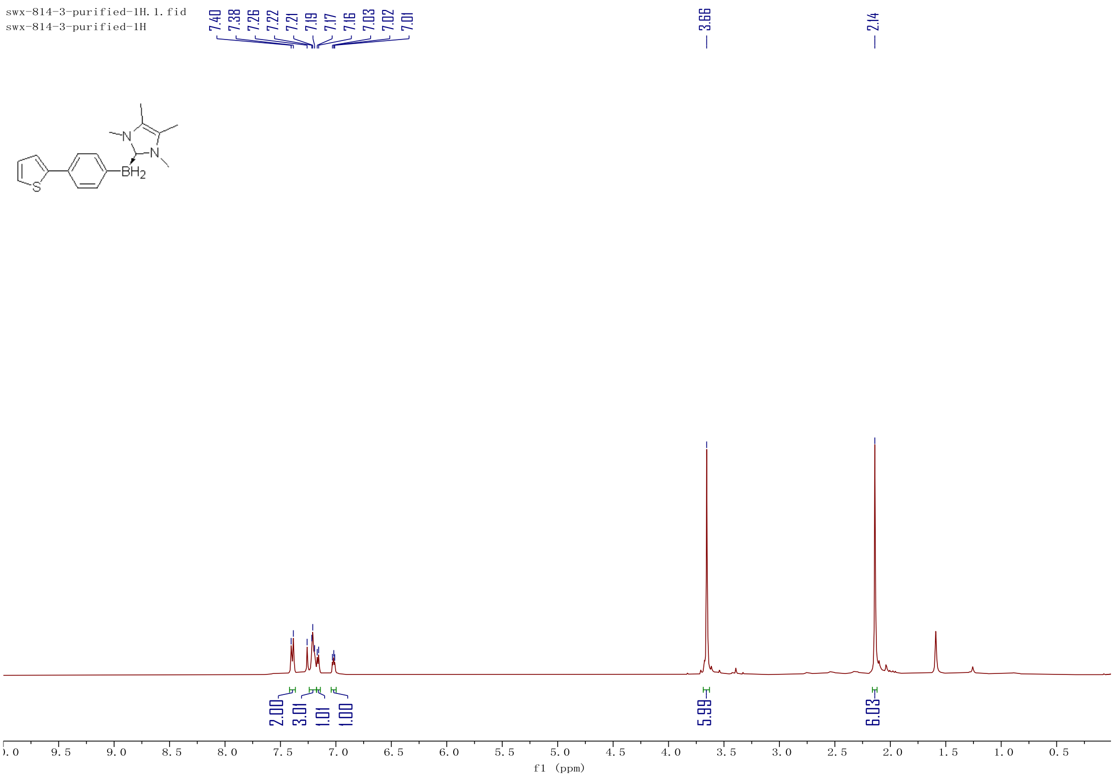

^{1}H NMR spectrum of **4v** in $CDCl_3$, 400 MHz.

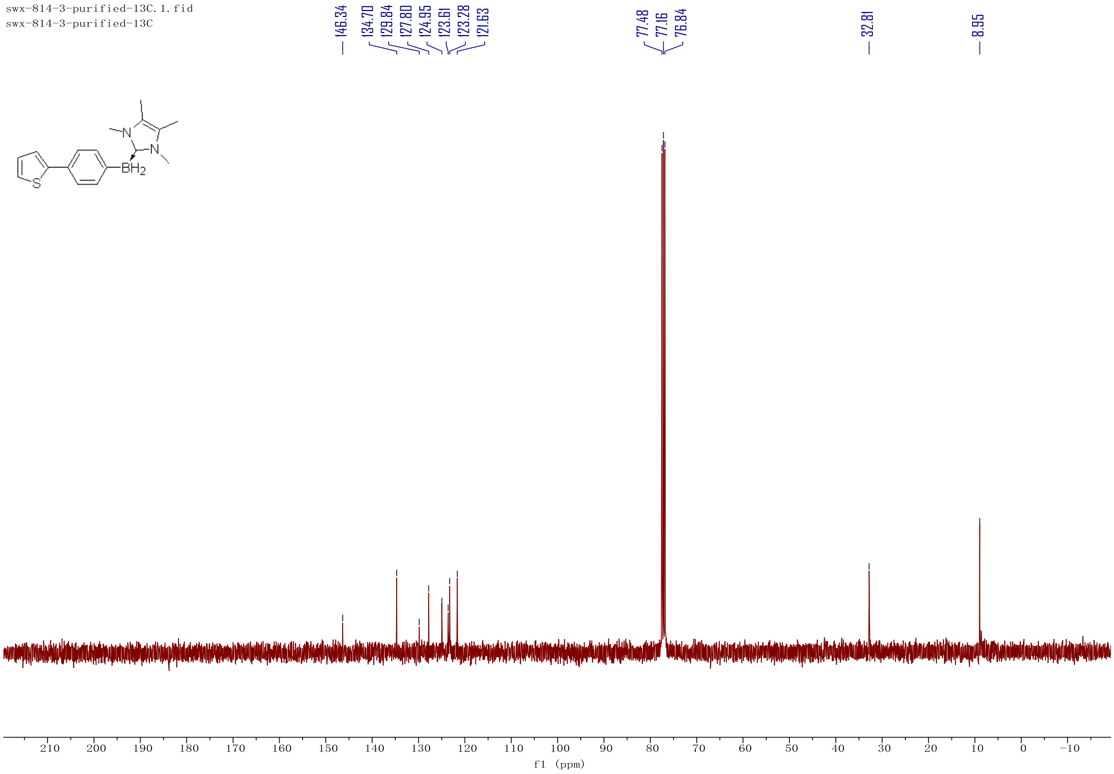

^{13}C NMR spectrum of **4v** in $CDCl_3$, 101 MHz.

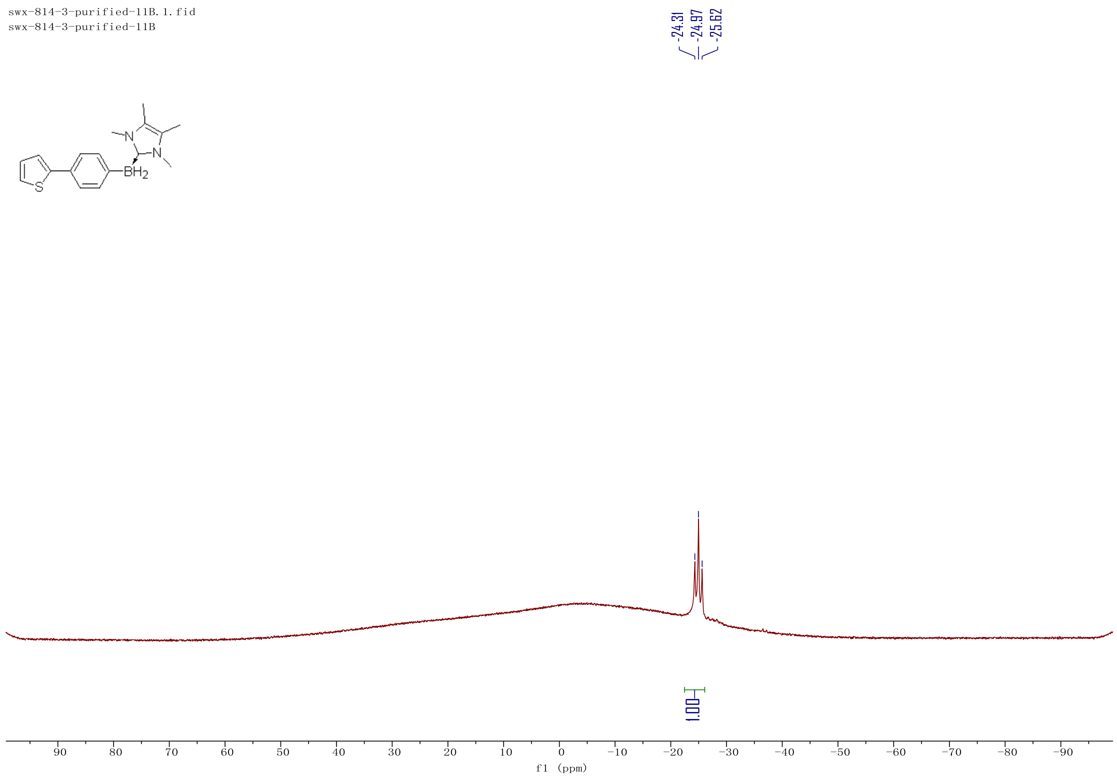

^{11}B NMR spectrum of **4v** in $CDCl_3$, 128 MHz.

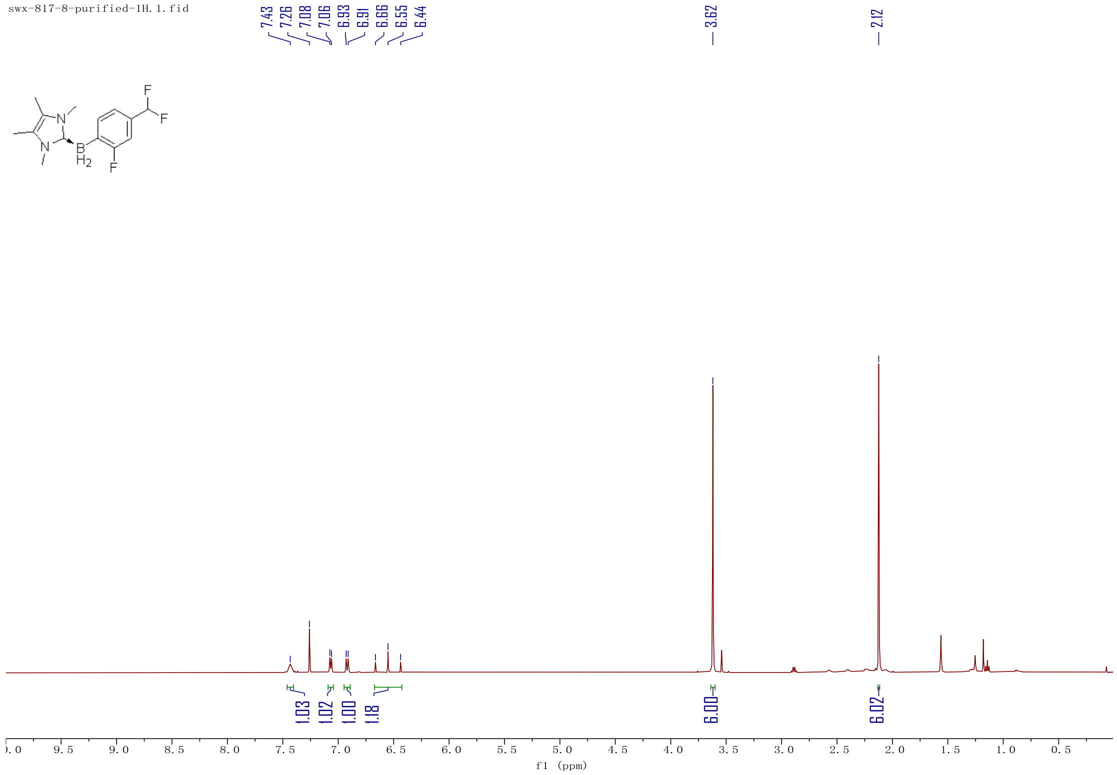

^{1}H NMR spectrum of **4w** in $CDCl_3$, 500 MHz.

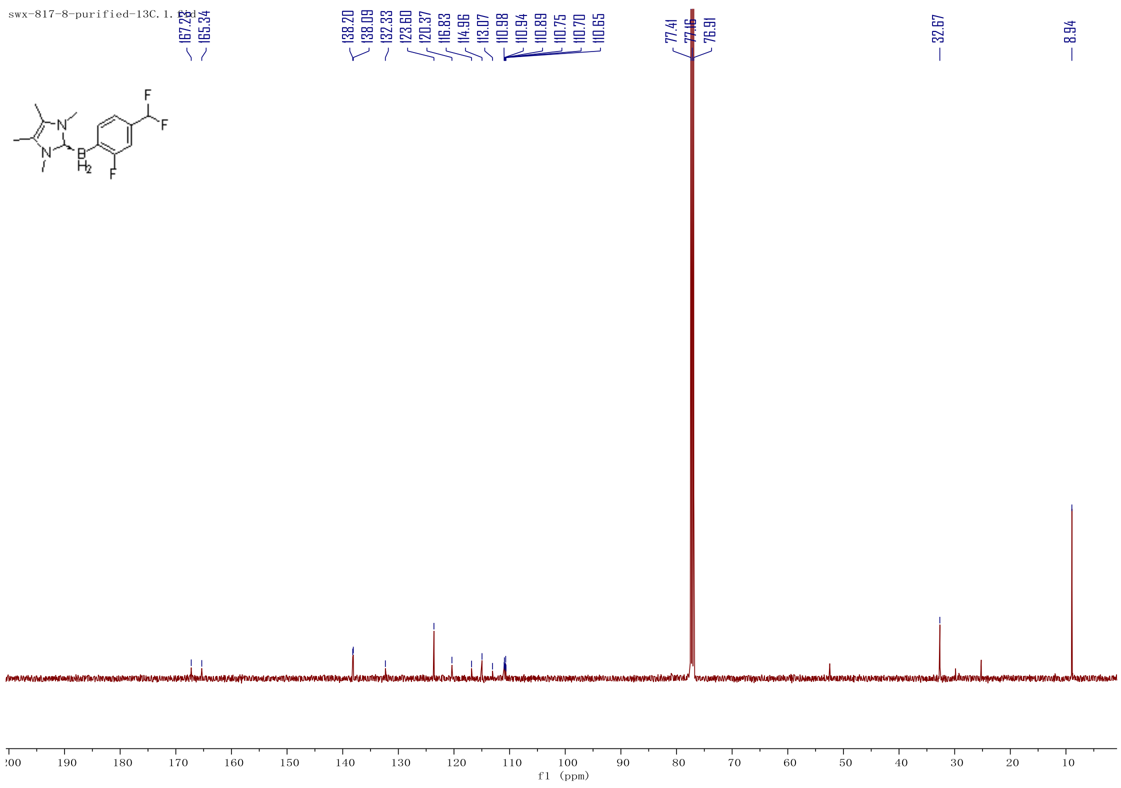

^{13}C NMR spectrum of **4w** in $CDCl_3$, 126 MHz.

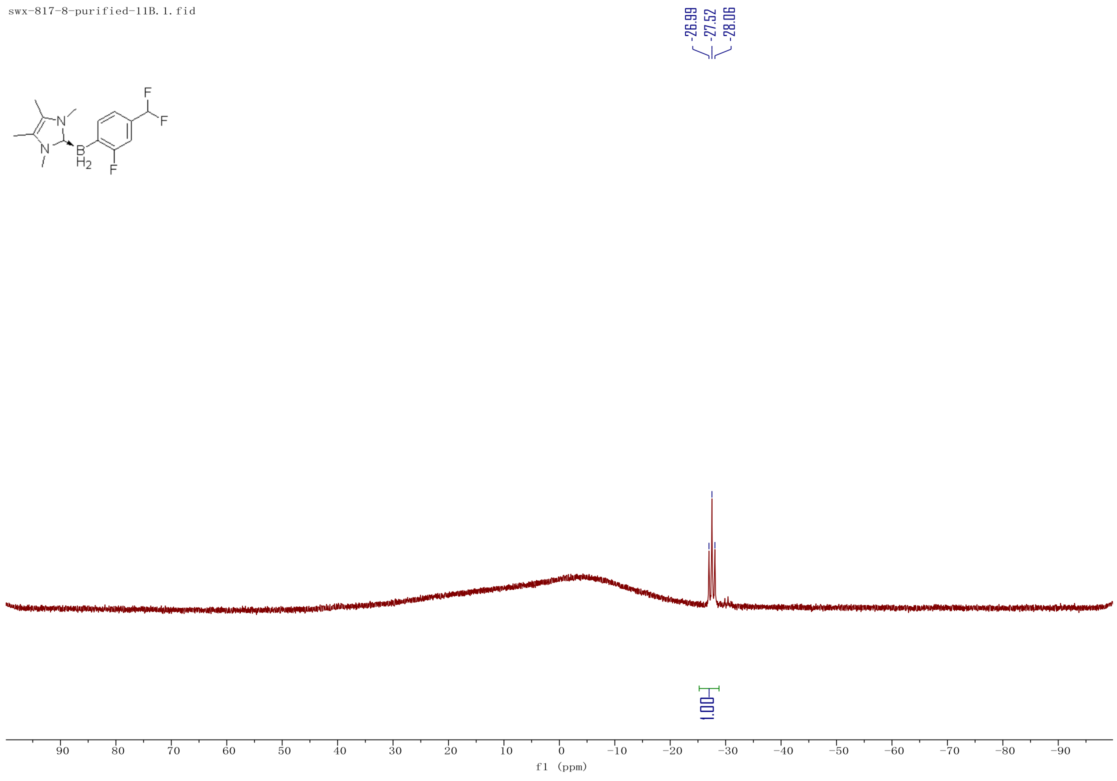

^{11}B NMR spectrum of **4w** in $CDCl_3$, 128 MHz.

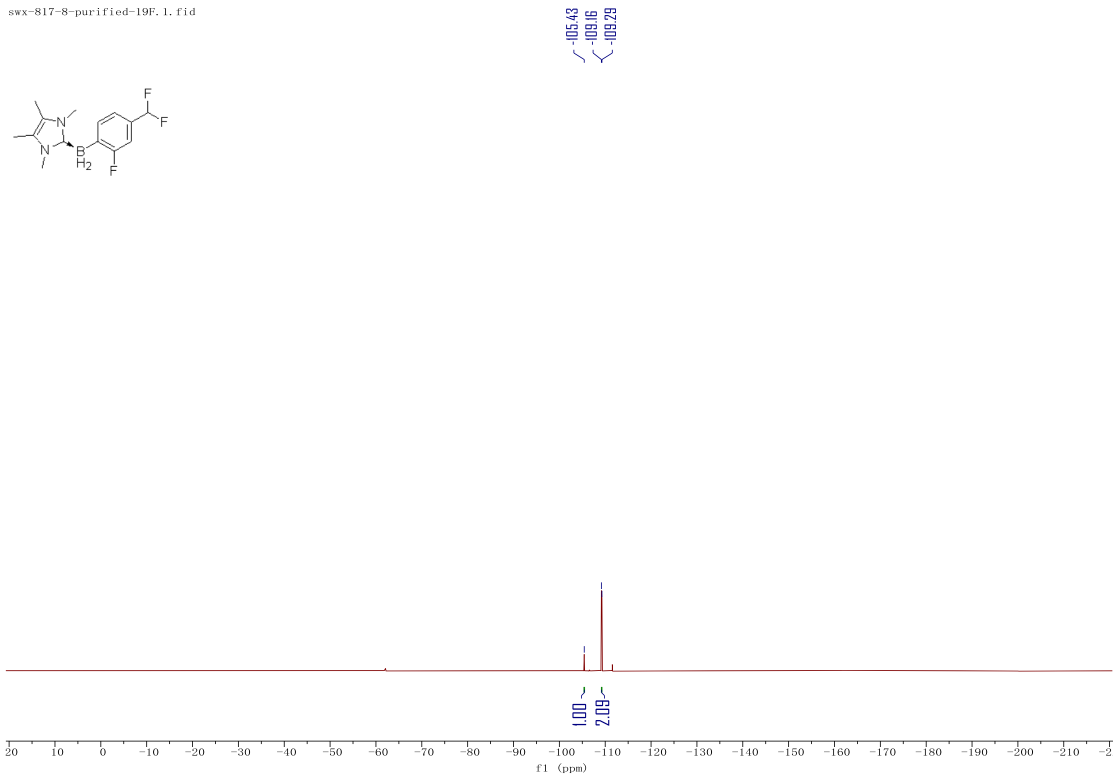

^{19}F NMR spectrum of **4w** in $CDCl_3$, 471 MHz.

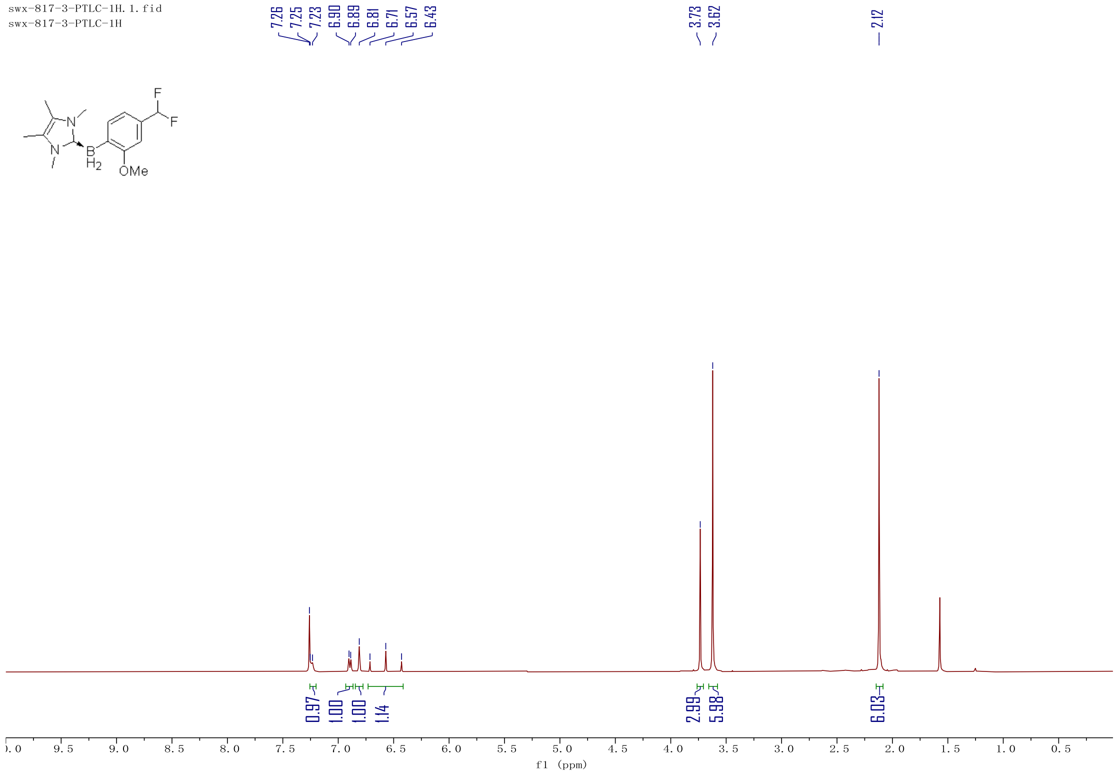

^{1}H NMR spectrum of **4x** in $CDCl_3$, 400 MHz.

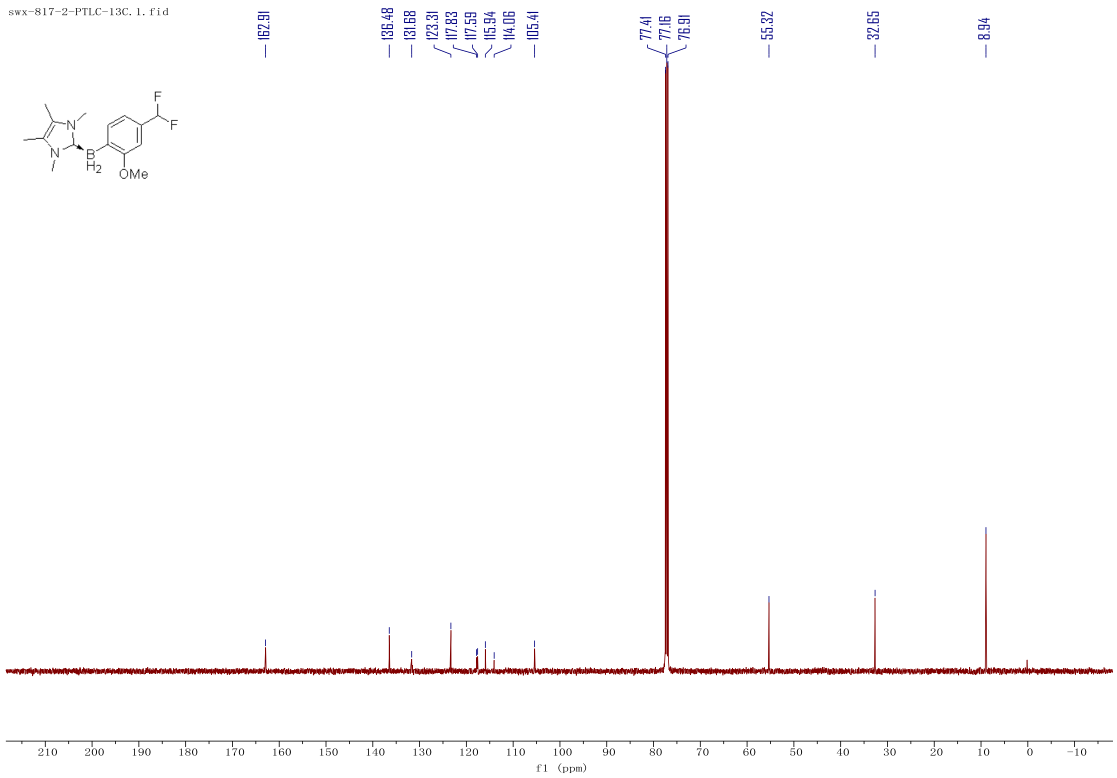

^{13}C NMR spectrum of **4x** in $CDCl_3$, 126 MHz.

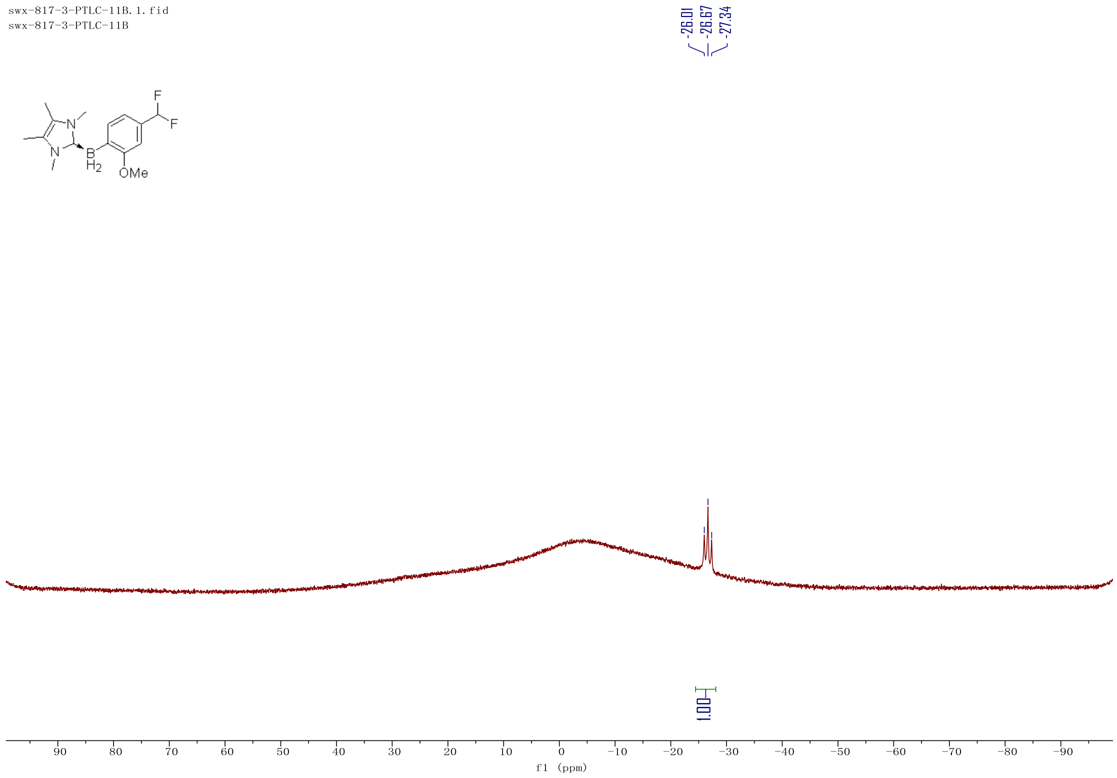

^{11}B NMR spectrum of **4x** in $CDCl_3$, 128 MHz.

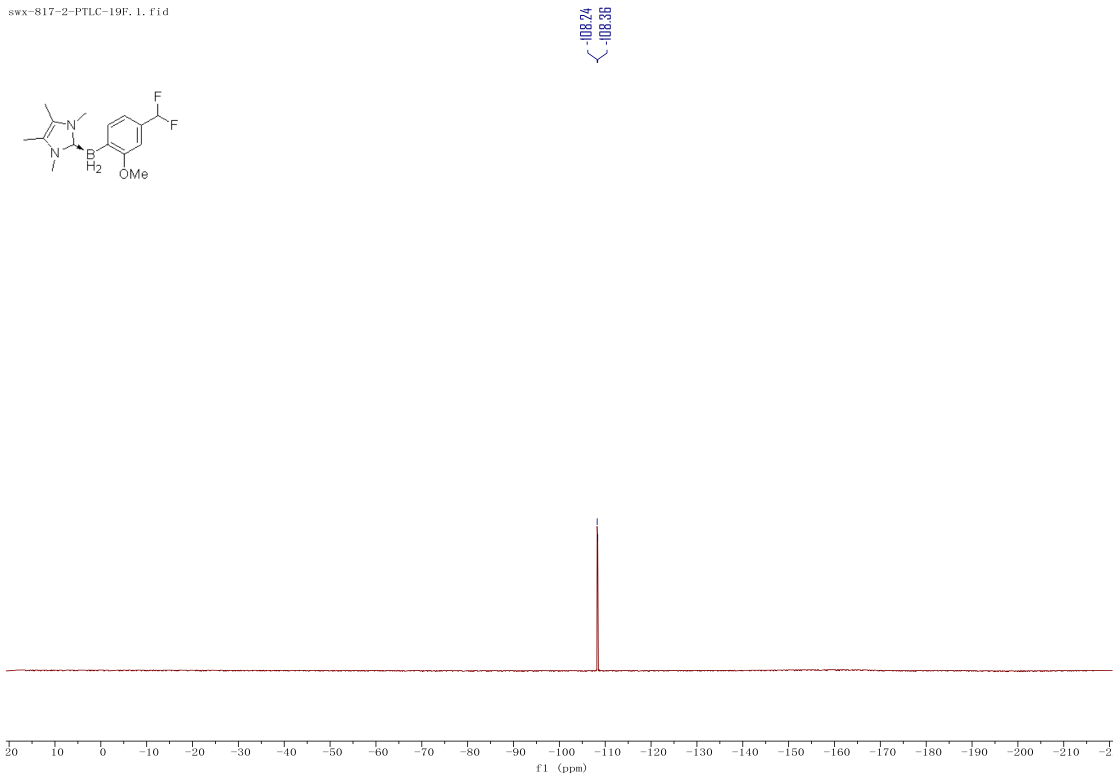

^{19}F NMR spectrum of **4x** in $CDCl_3$, 471 MHz.

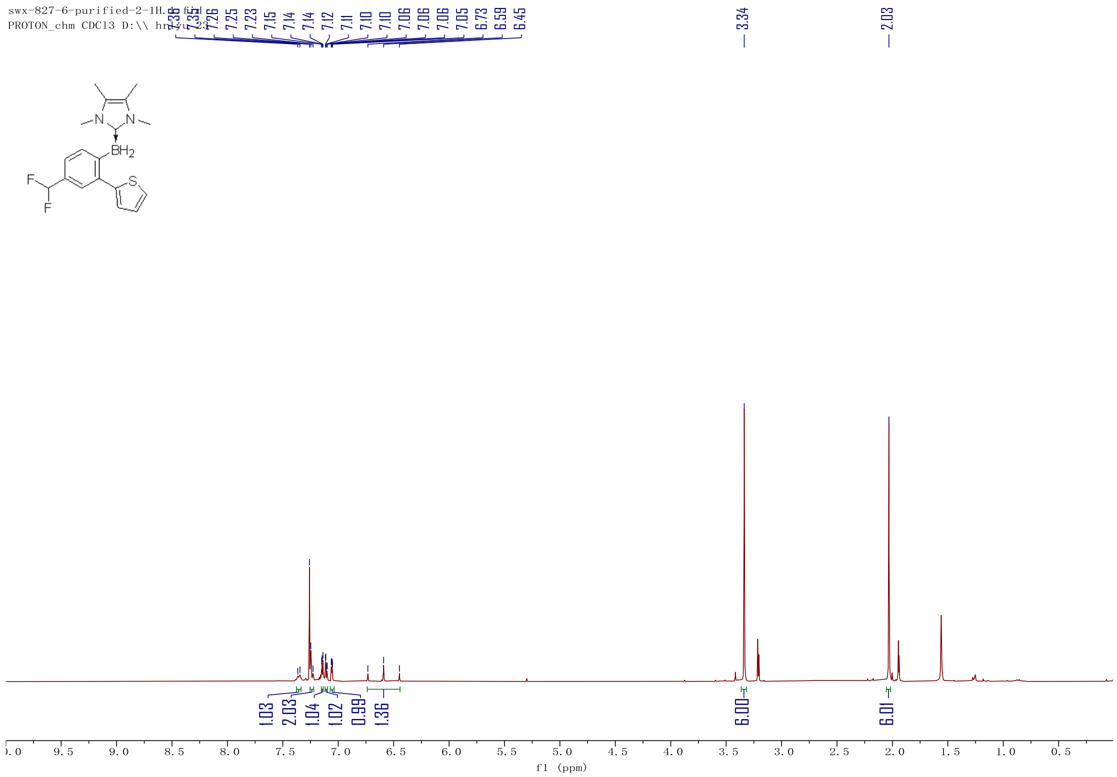

^{1}H NMR spectrum of **4y**in $CDCl_3$, 400 MHz.

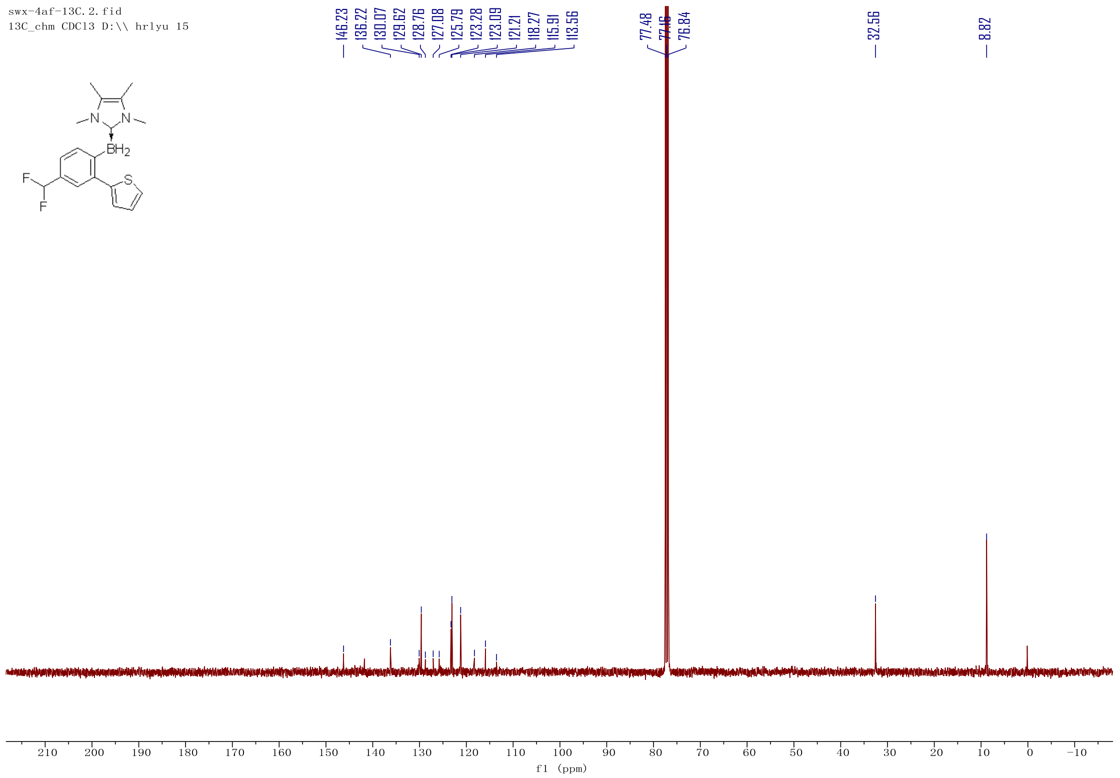

^{13}C NMR spectrum of **4y** in $CDCl_3$, 101 MHz.

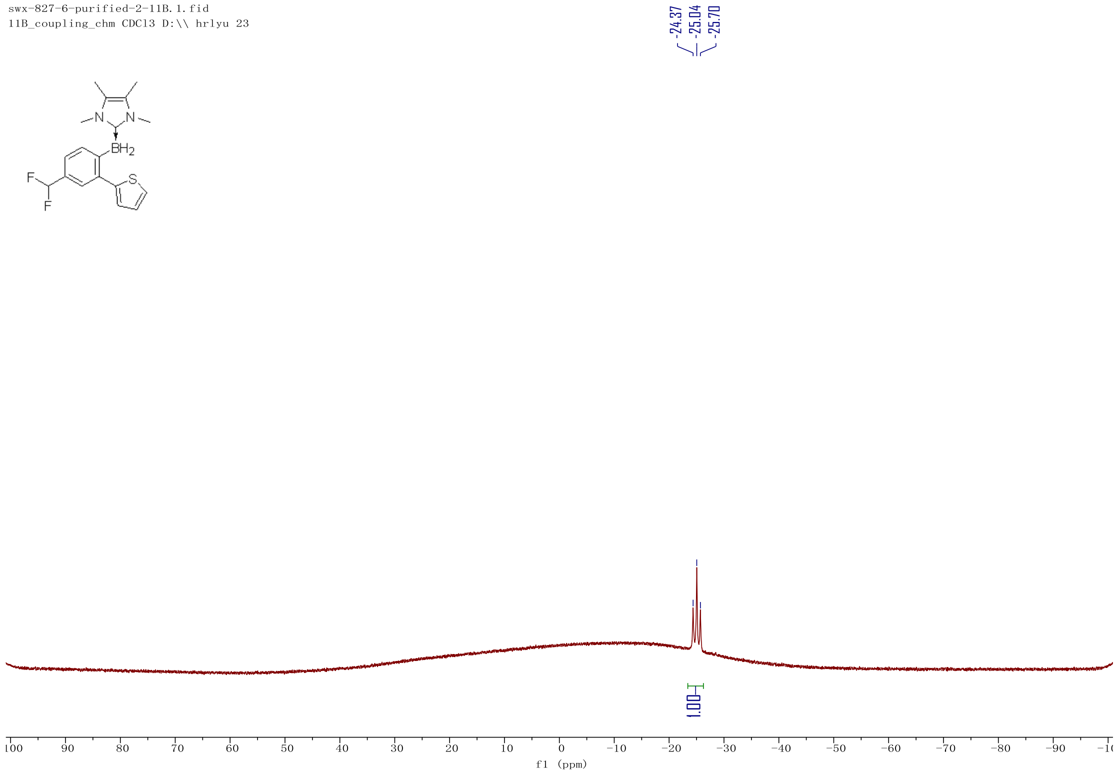

^{11}B NMR spectrum of **4y** in $CDCl_3$, 128 MHz.

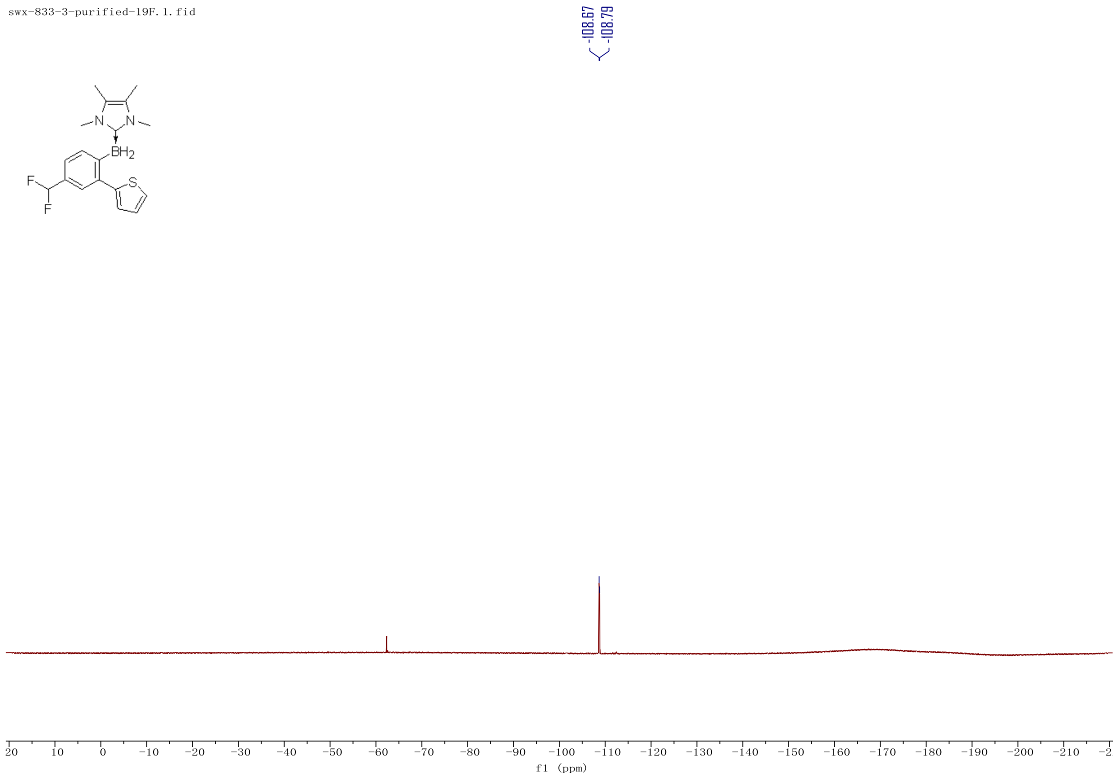

^{19}F NMR spectrum of **4y** in $CDCl_3$, 471 MHz.

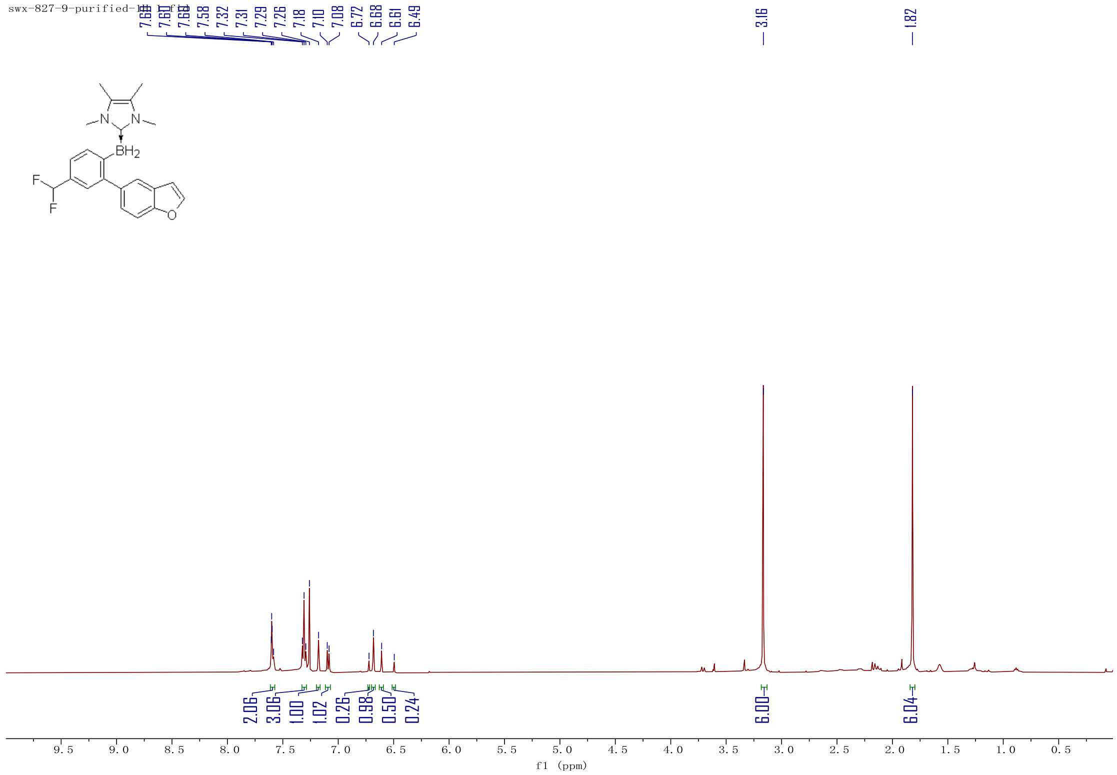

^{1}H NMR spectrum of **4z** in $CDCl_3$, 500 MHz.

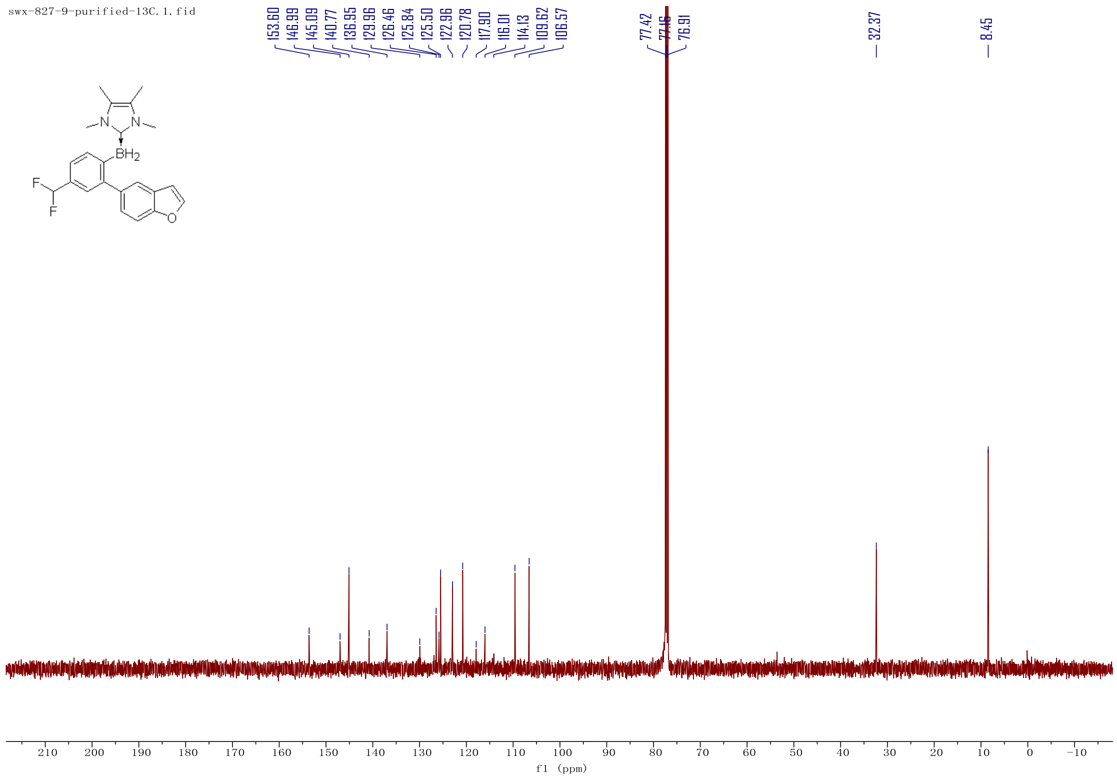

^{13}C NMR spectrum of **4z** in $CDCl_3$, 126 MHz.

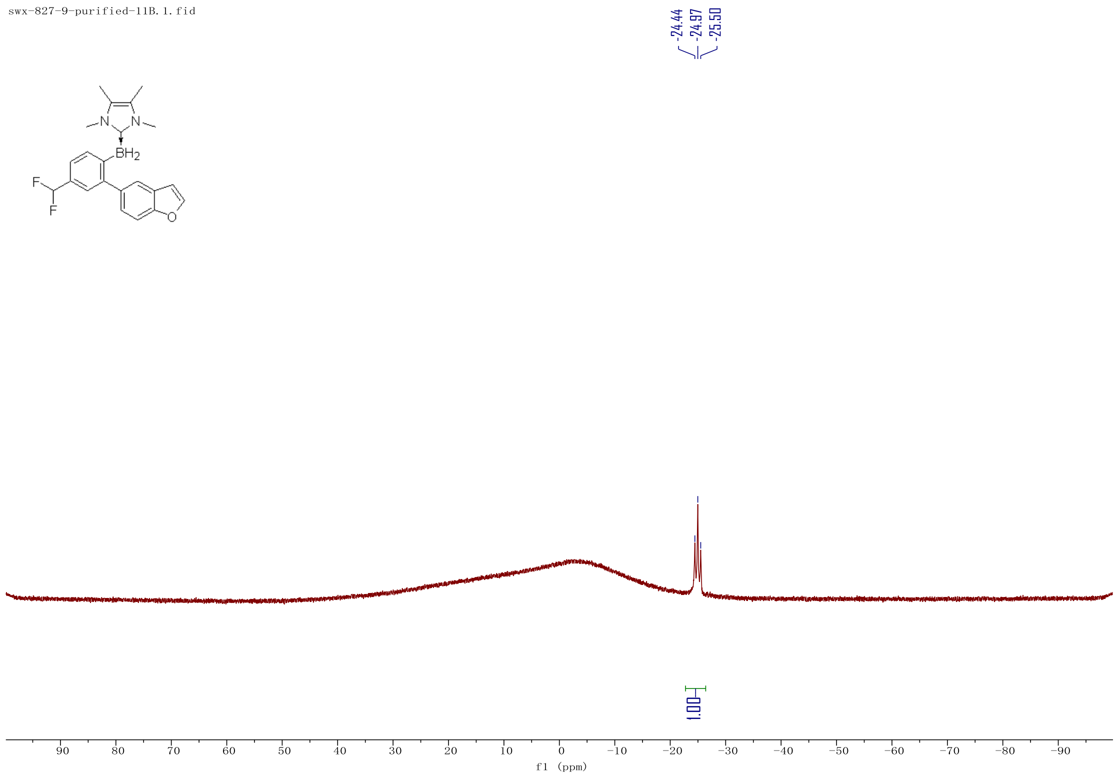

^{11}B NMR spectrum of **4z** in $CDCl_3$, 160 MHz.

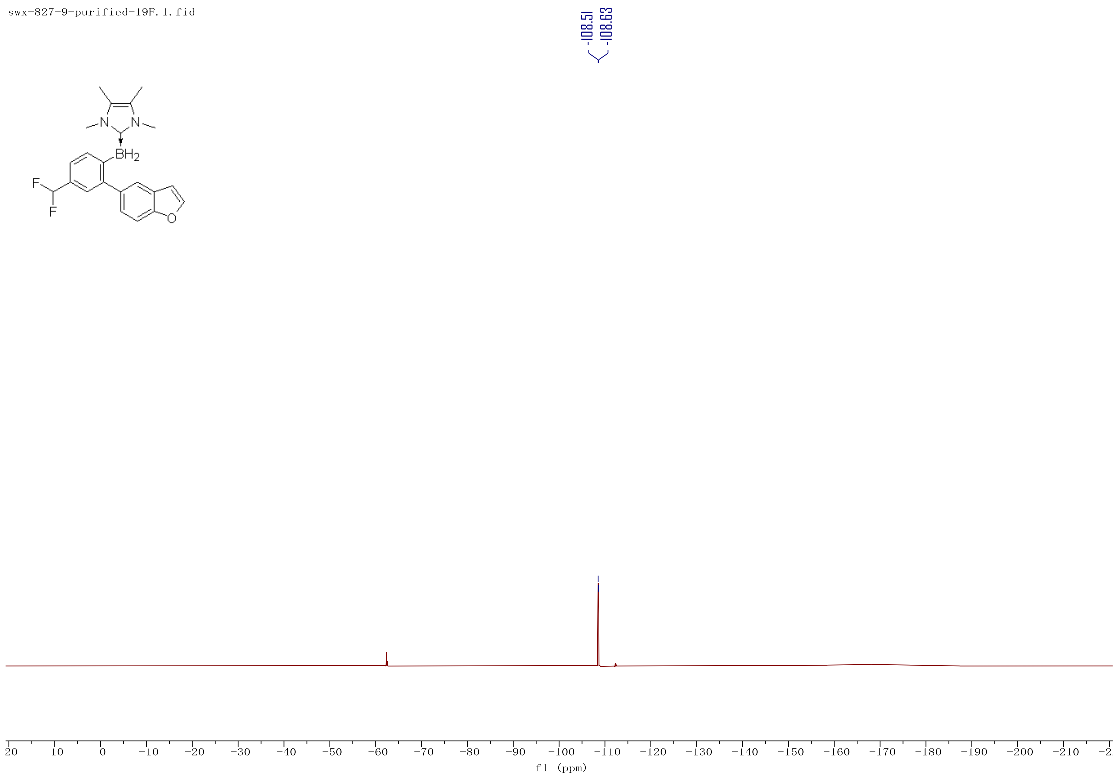

^{19}F NMR spectrum of **4z** in $CDCl_3$, 471 MHz.

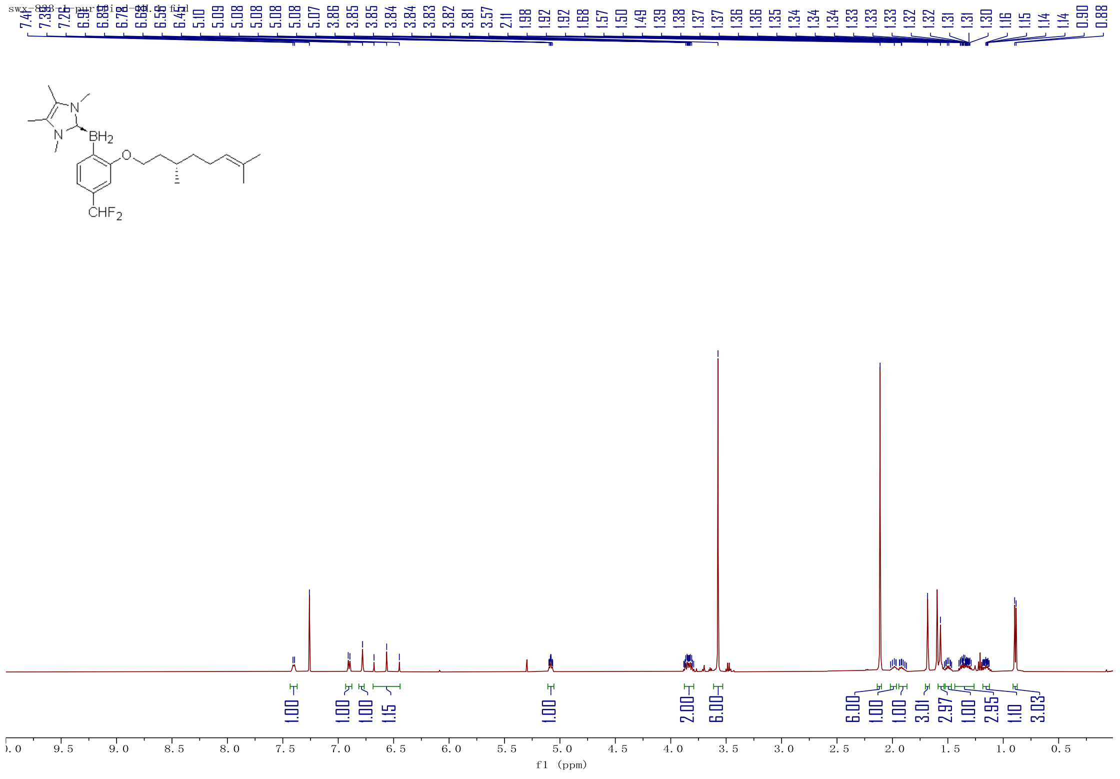

^{1}H NMR spectrum of **4aa** in $CDCl_3$, 500 MHz.

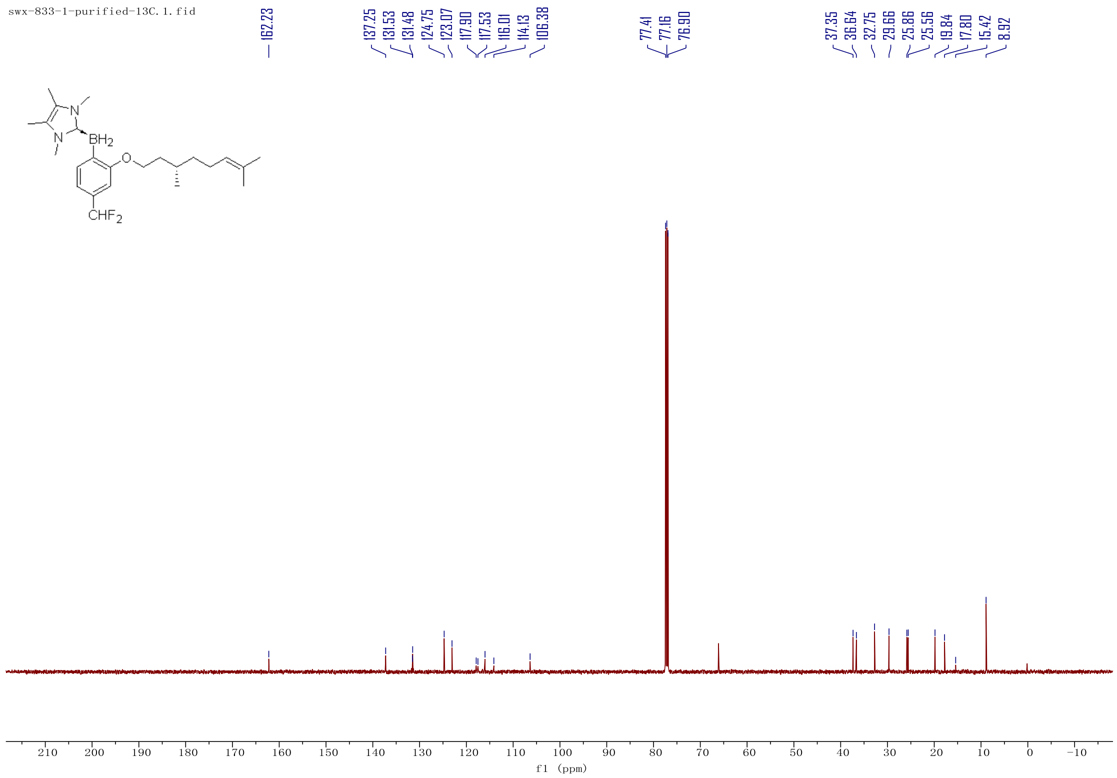

^{13}C NMR spectrum of **4aa** in $CDCl_3$, 126 MHz.

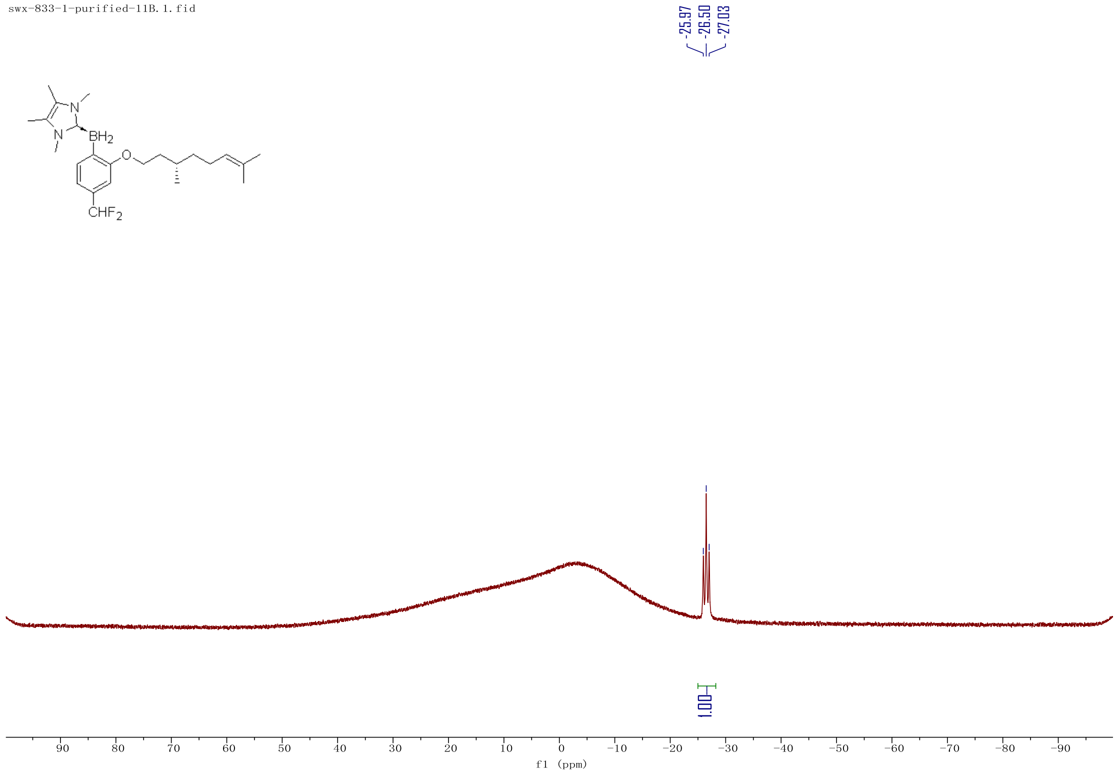

^{11}B NMR spectrum of **4aa** in $CDCl_3$, 160 MHz.

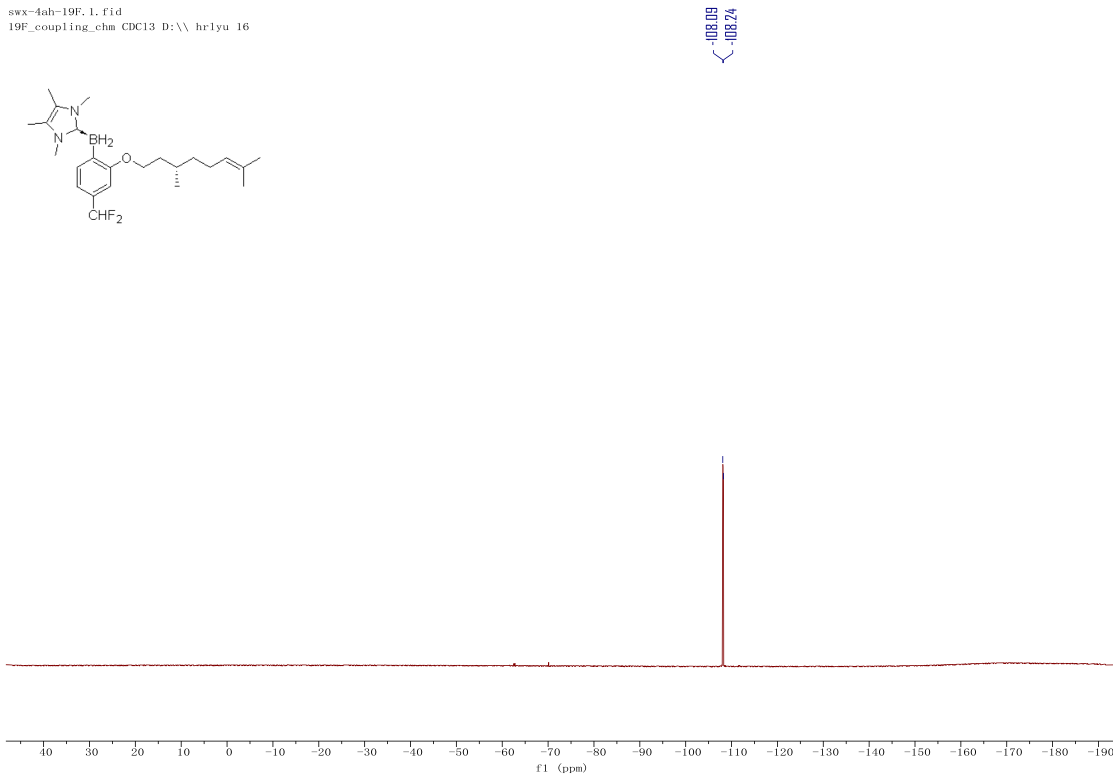

^{19}F NMR spectrum of **4aa** in $CDCl_3$, 377 MHz.

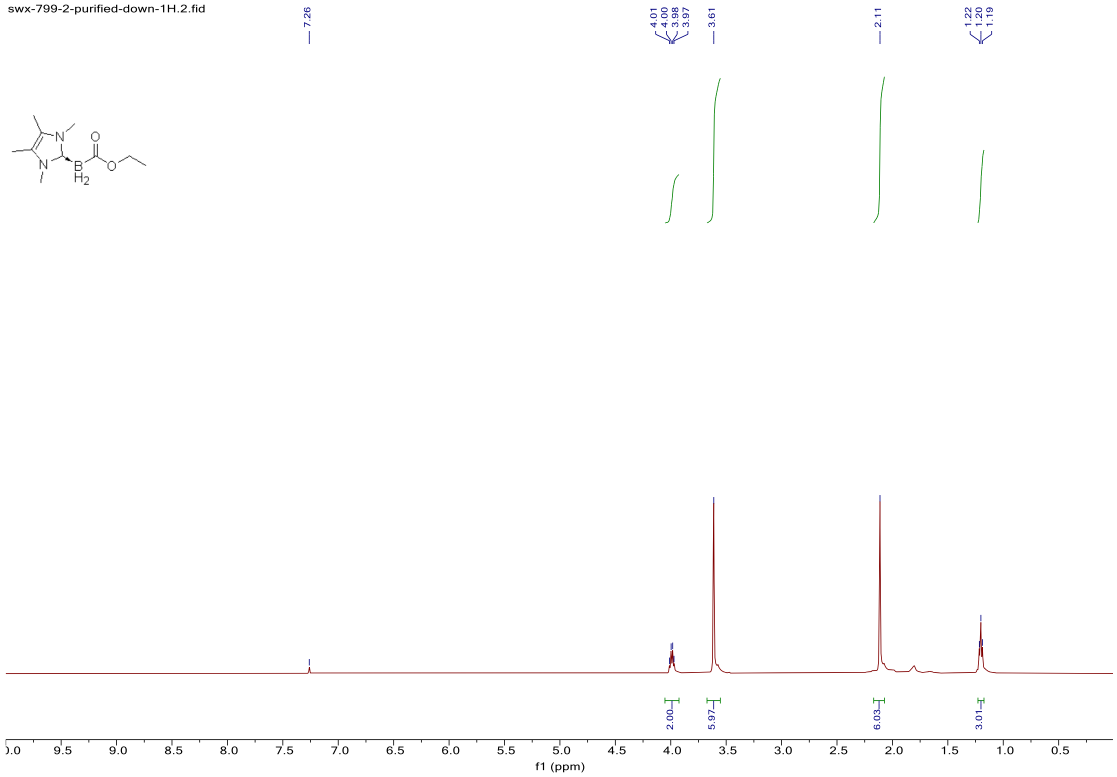

^{1}H NMR spectrum of **4ab** in $CDCl_3$, 500 MHz.

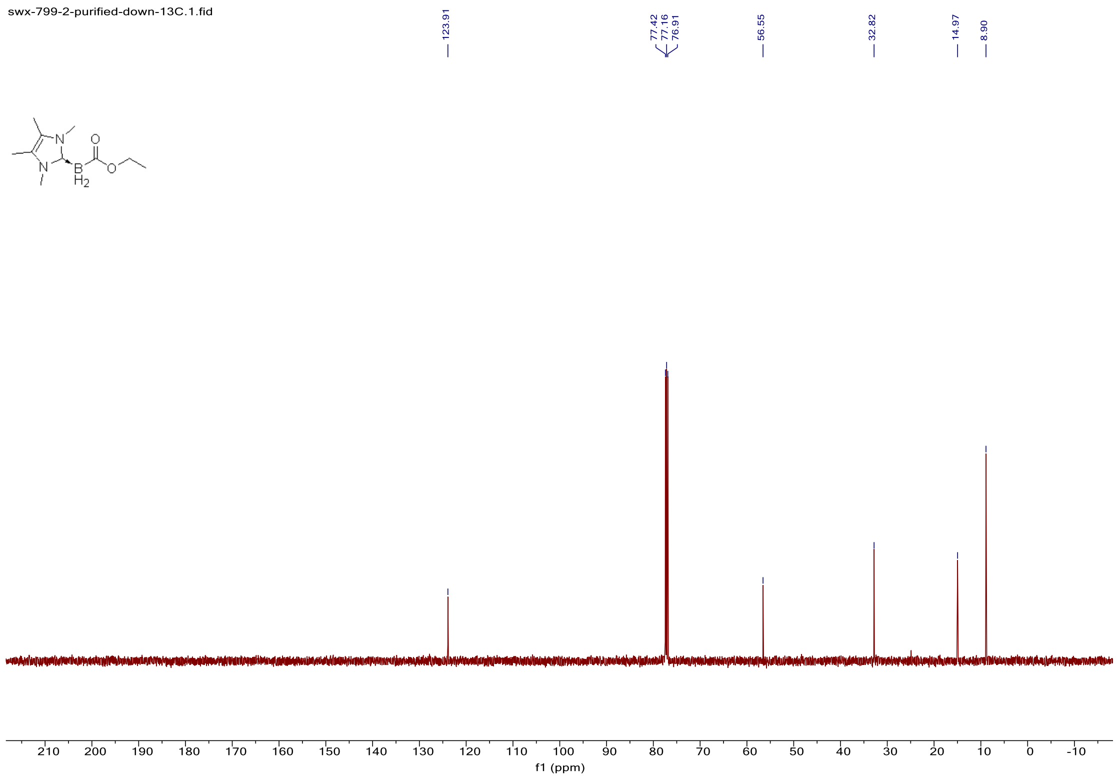

^{13}C NMR spectrum of **4ab** in $CDCl_3$, 126 MHz.

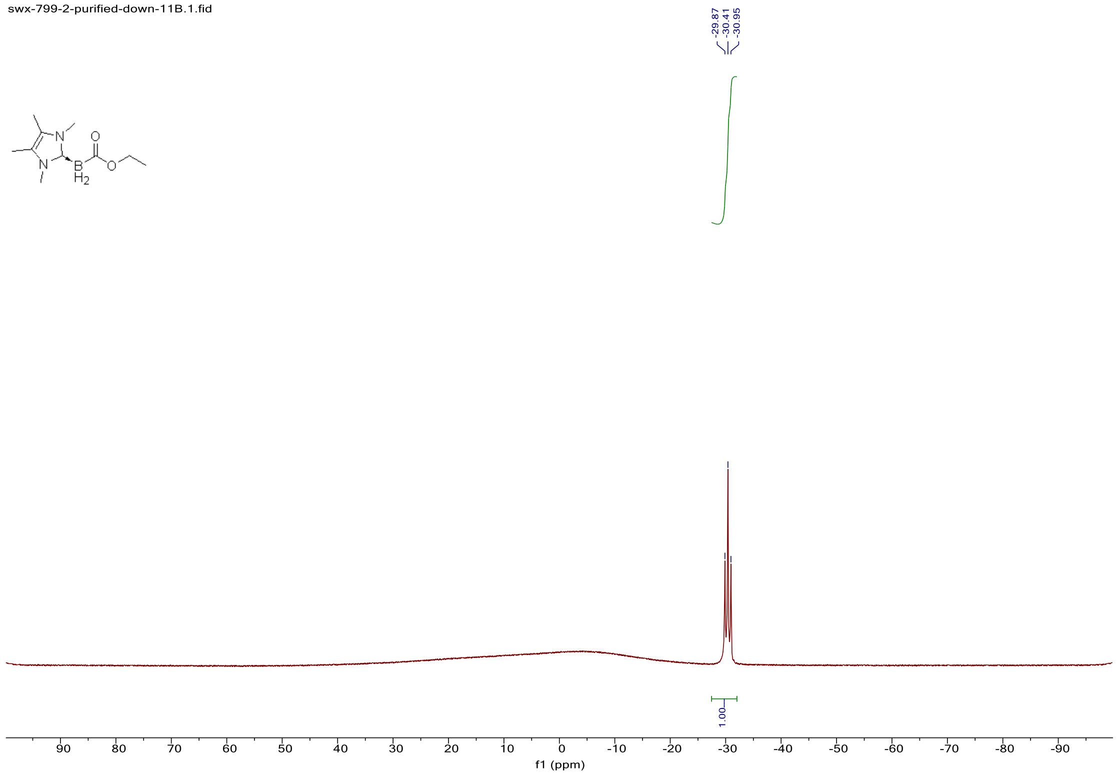

^{11}B NMR spectrum of **4ab** in $CDCl_3$, 160 MHz.

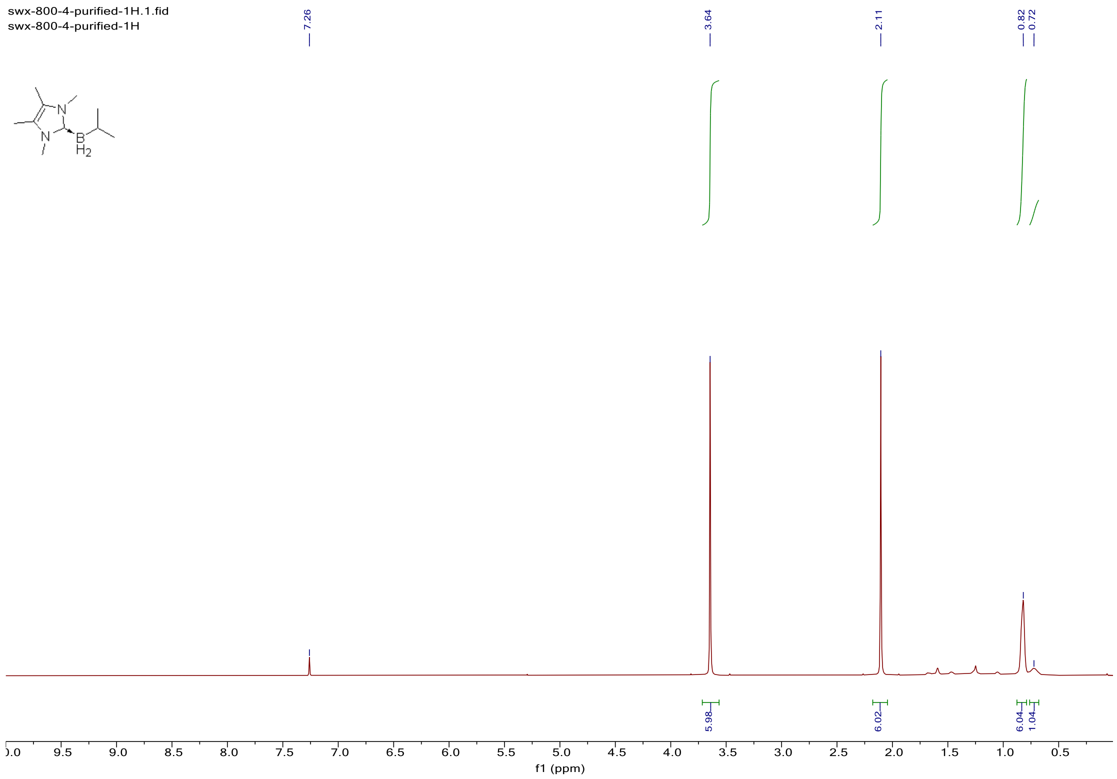

^{1}H NMR spectrum of **4ac** in $CDCl_3$, 400 MHz.

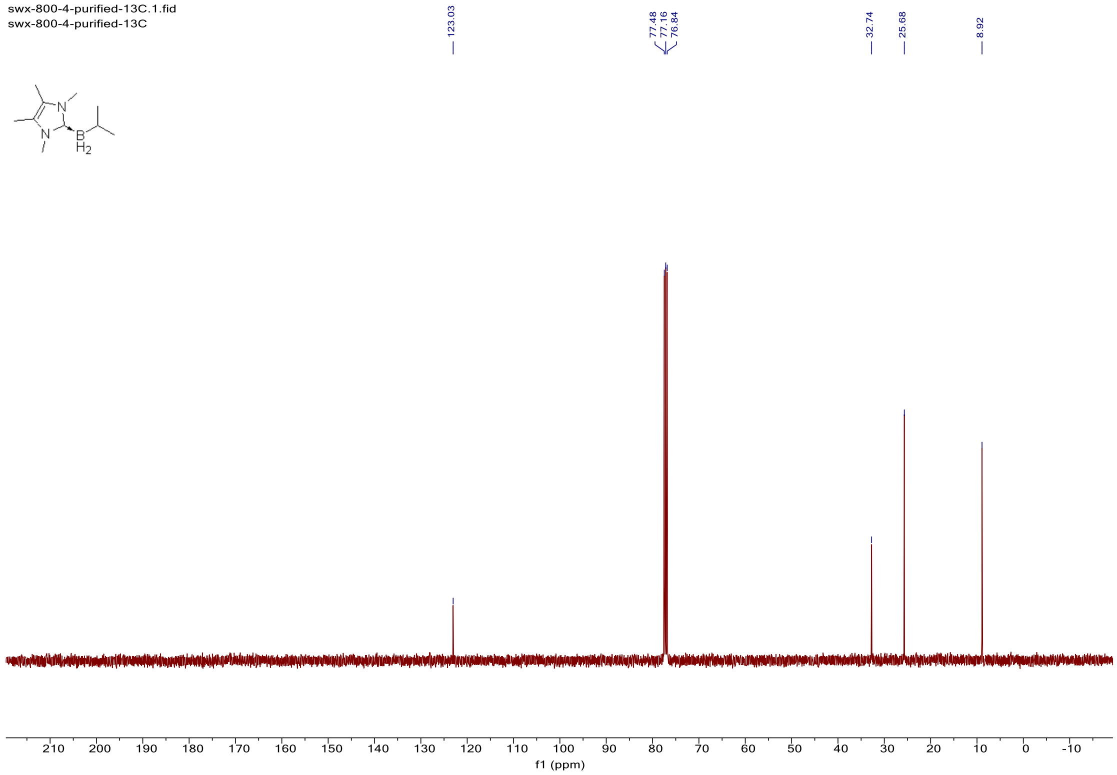

^{13}C NMR spectrum of **4ac** in $CDCl_3$, 101 MHz.

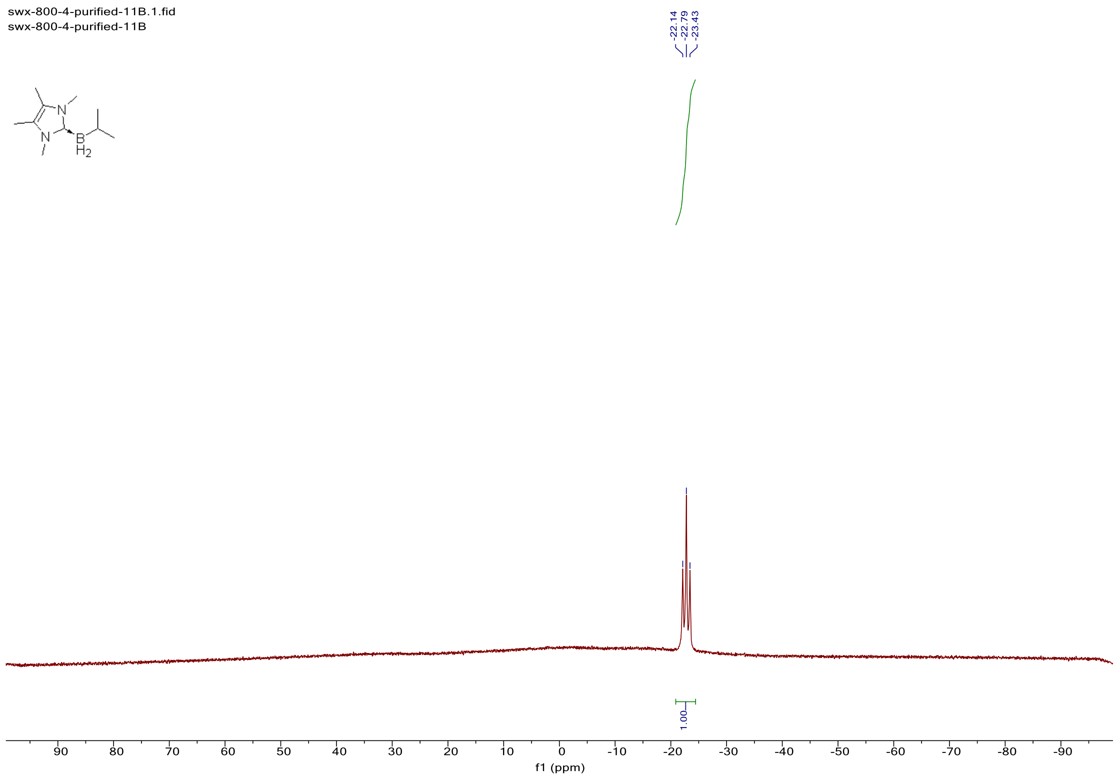

^{11}B NMR spectrum of **4ac** in $CDCl_3$, 128 MHz.

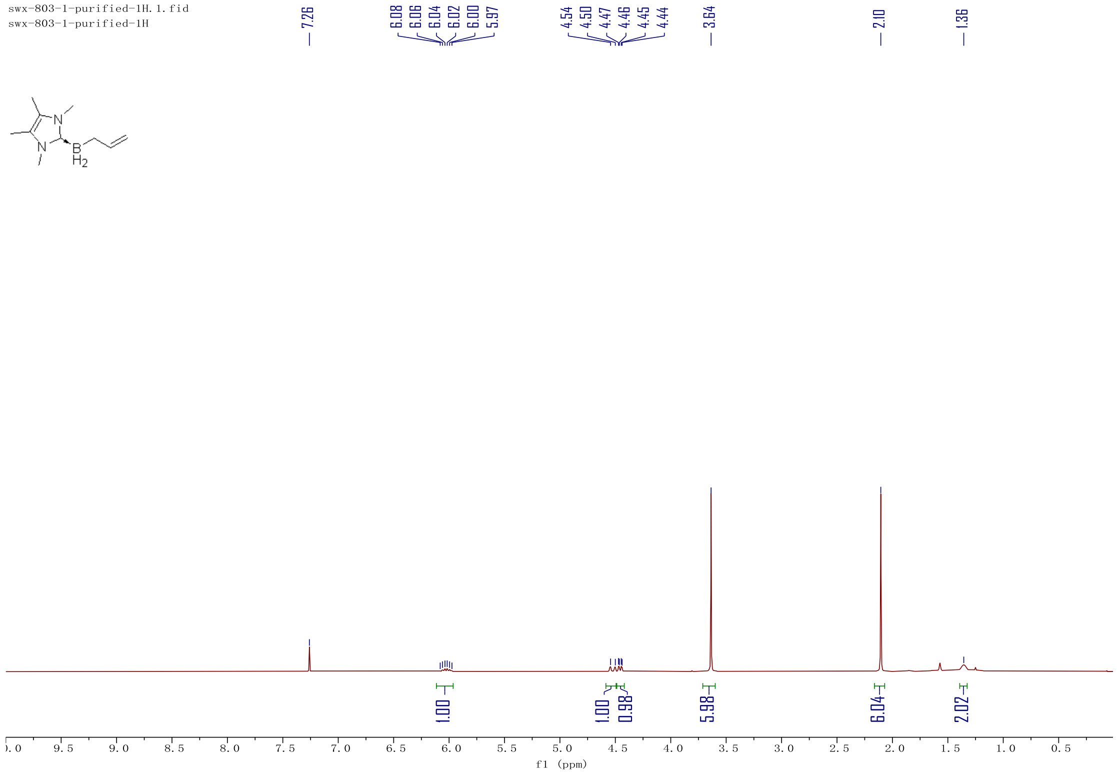

^{1}H NMR spectrum of **4ad** in $CDCl_3$, 400 MHz.

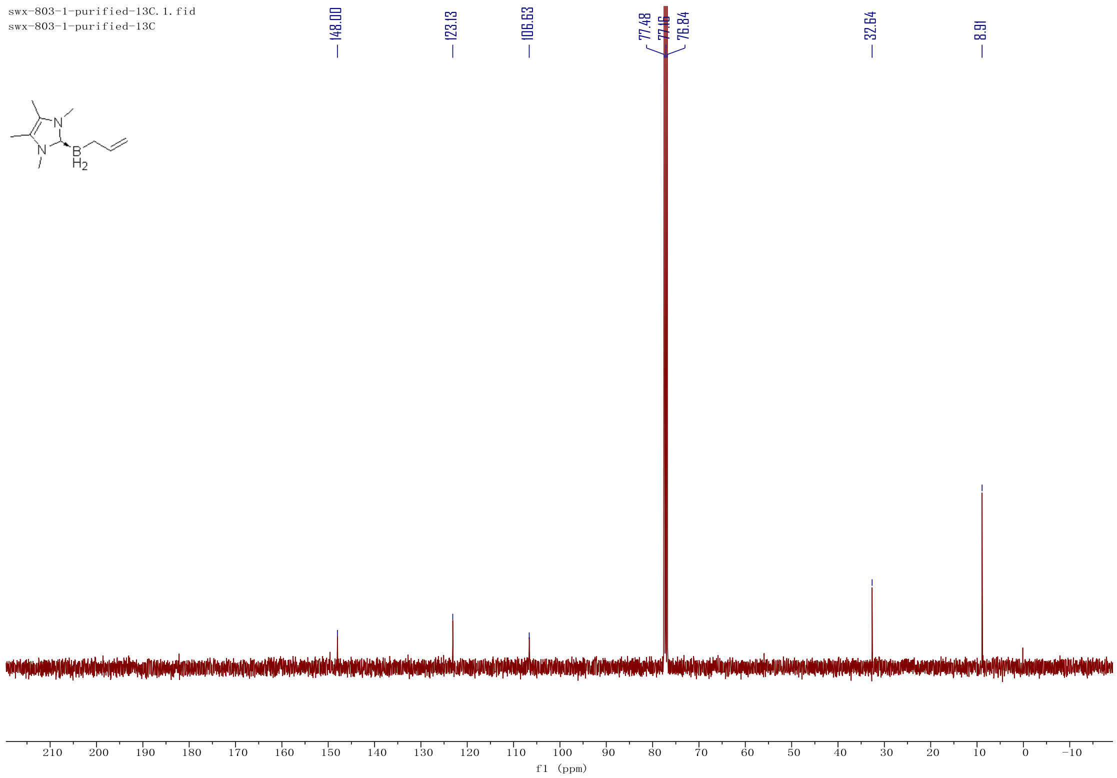

^{13}C NMR spectrum of **4ad** in $CDCl_3$, 101 MHz.

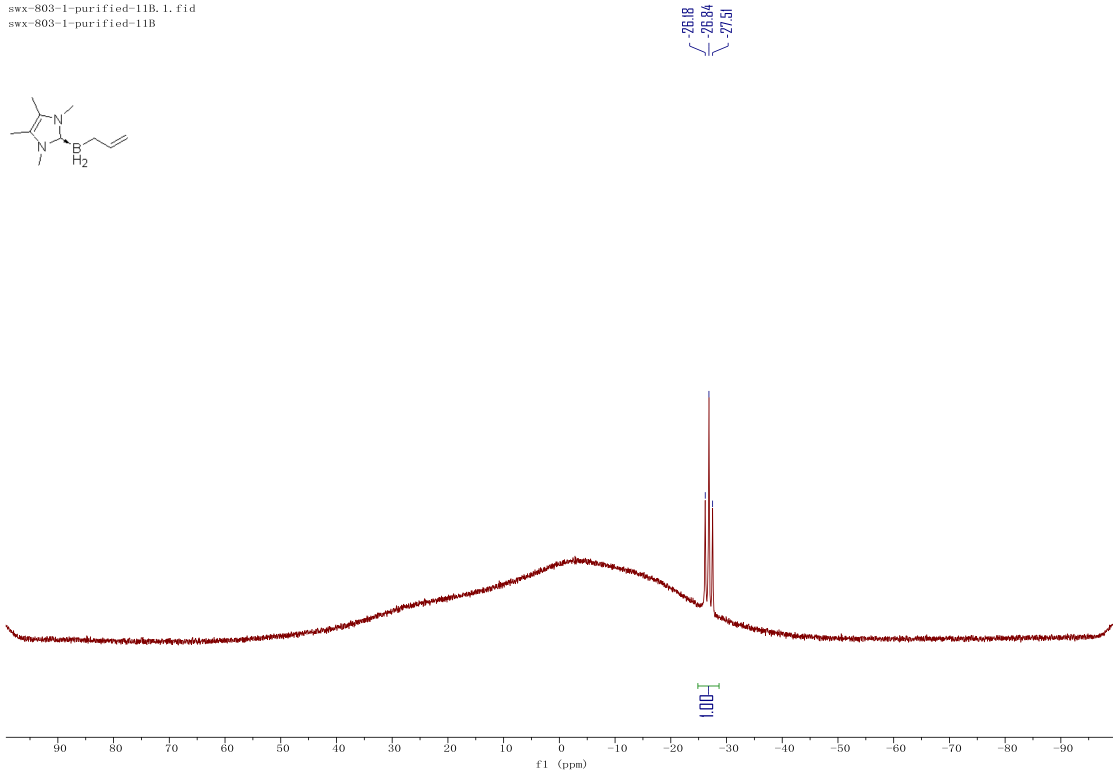

^{11}B NMR spectrum of **4ad** in $CDCl_3$, 128 MHz.

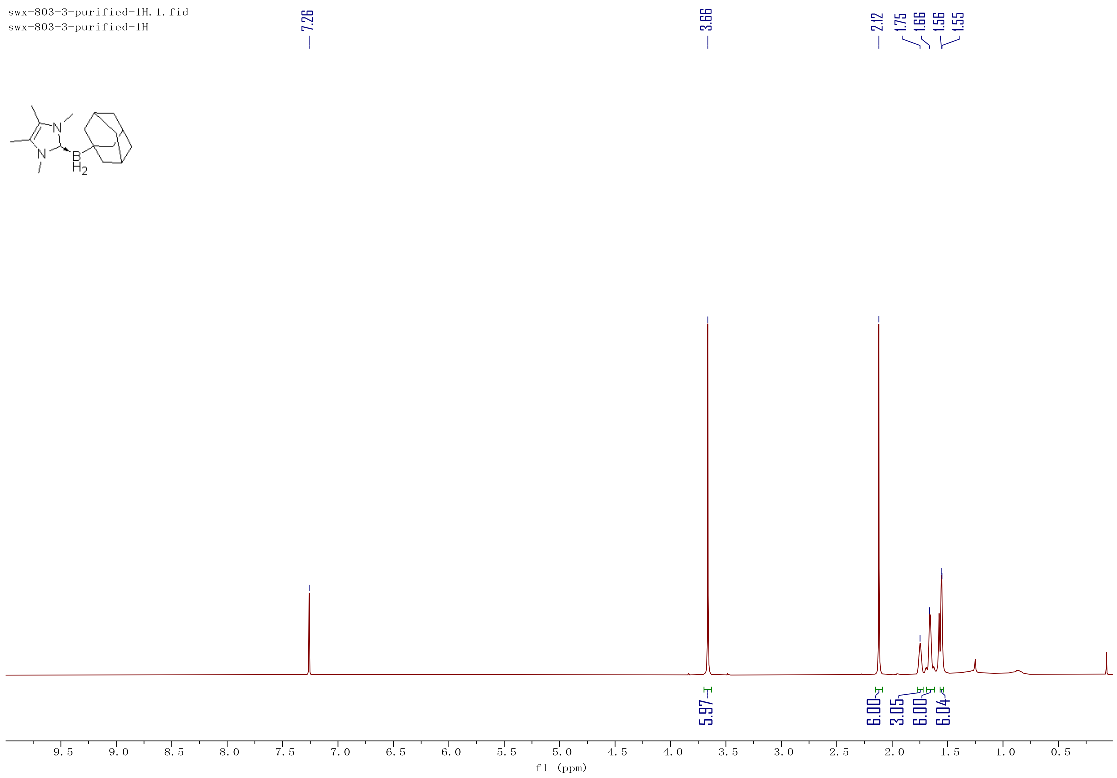

^{1}H NMR spectrum of **4ae** in $CDCl_3$, 400 MHz.

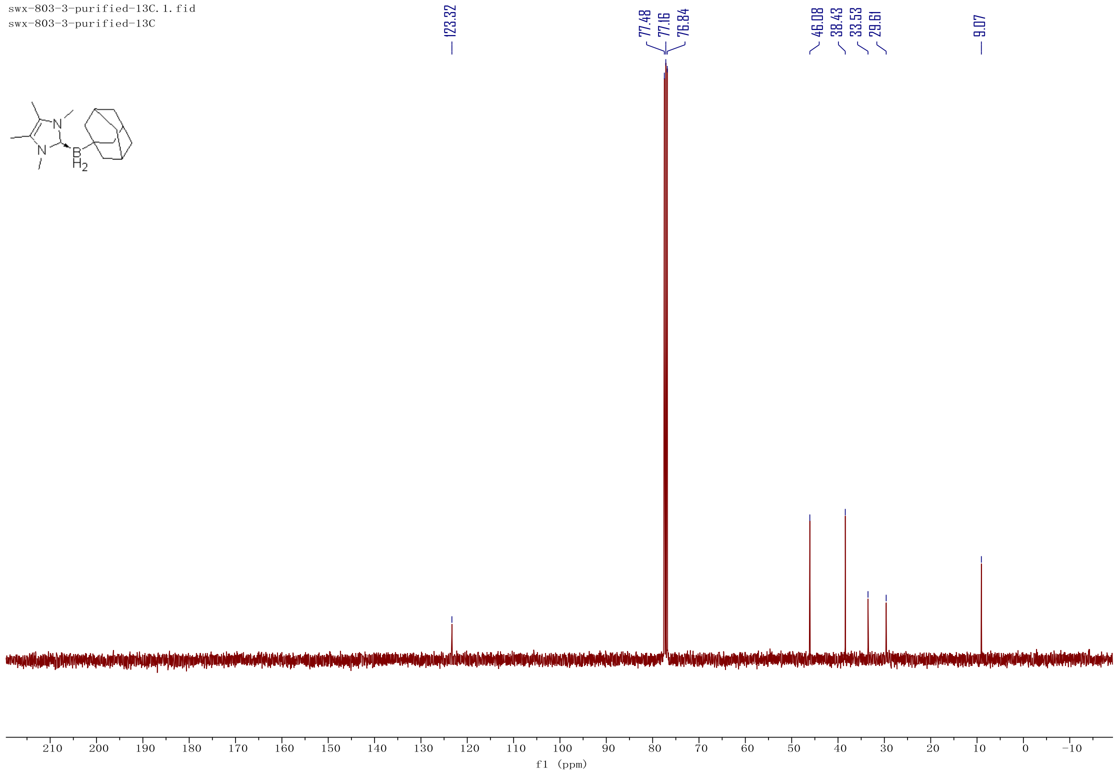

^{13}C NMR spectrum of **4ae** in $CDCl_3$, 101 MHz.

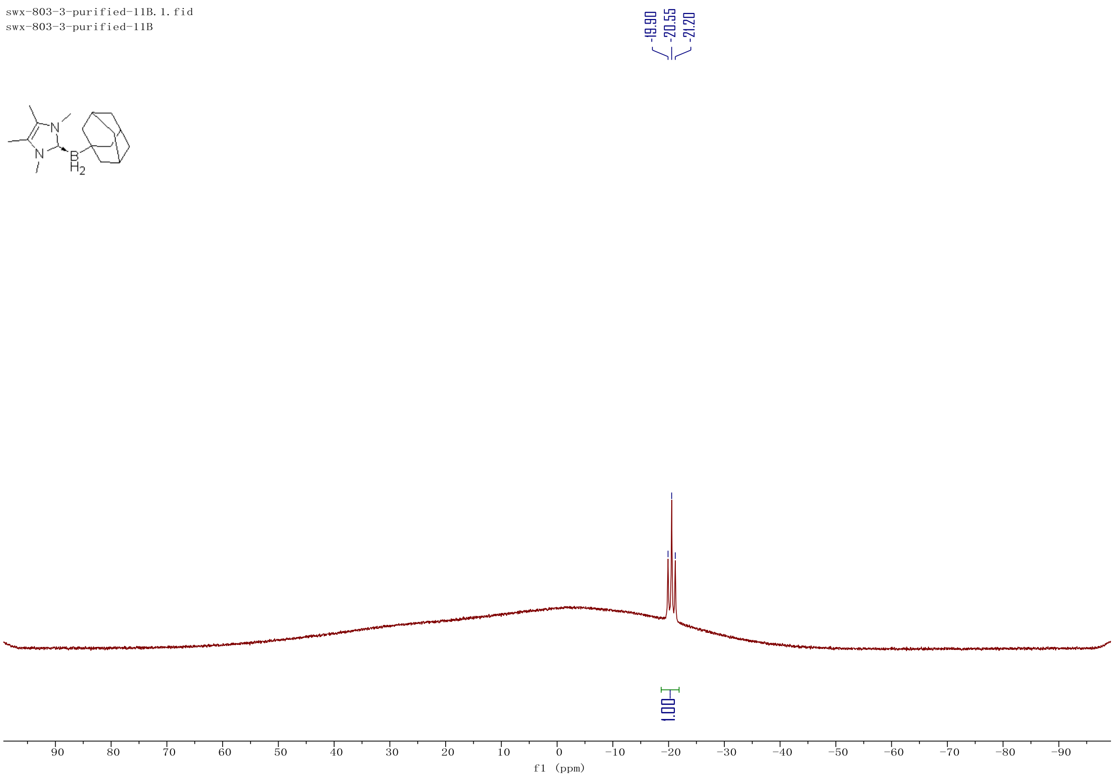

^{11}B NMR spectrum of **4ae** in $CDCl_3$, 128 MHz.

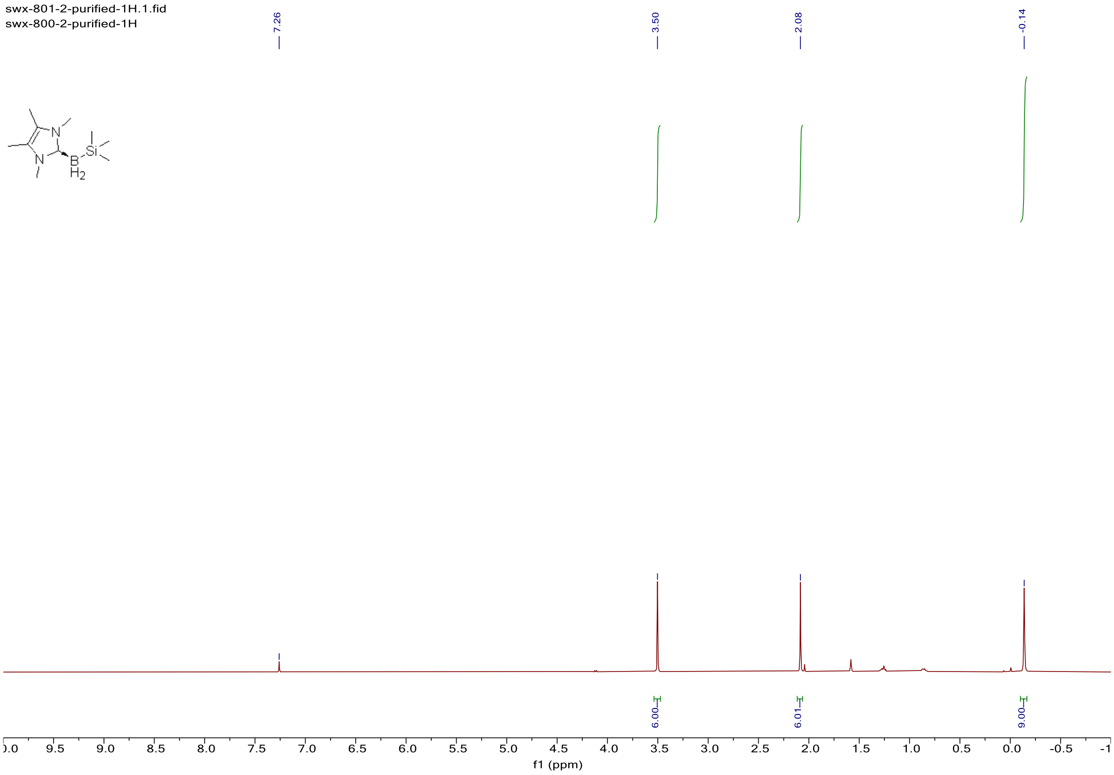

^{1}H NMR spectrum of **4af** in $CDCl_3$, 400 MHz.

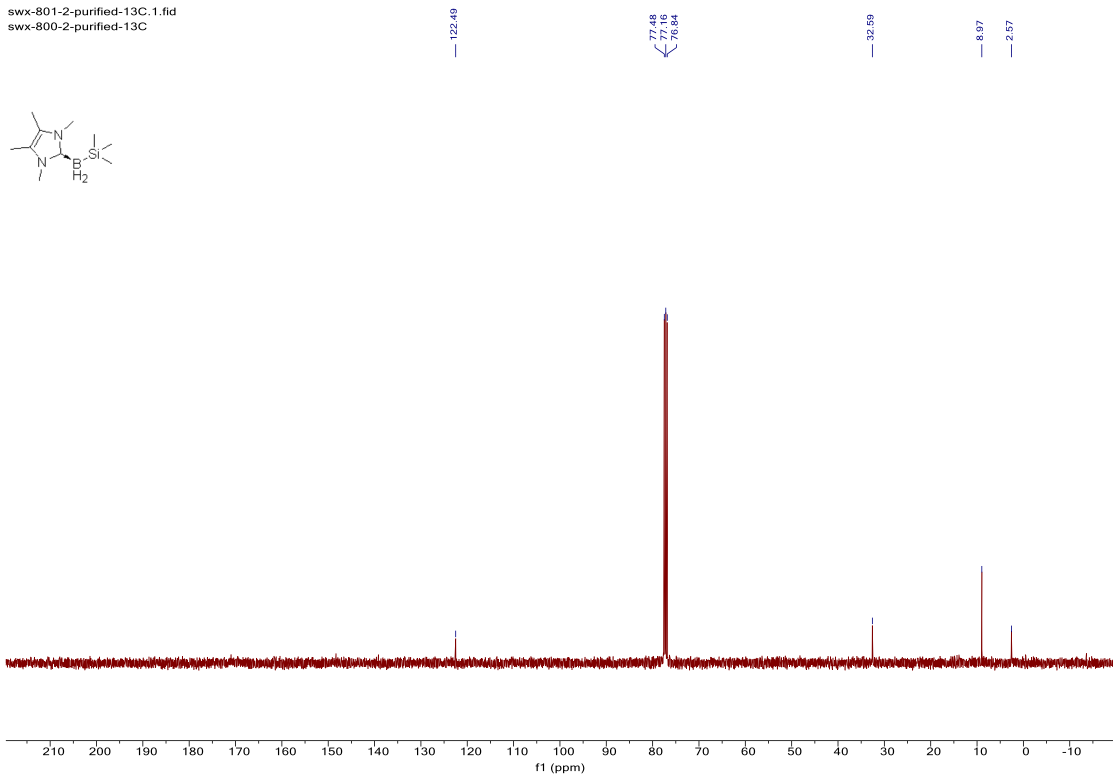

^{13}C NMR spectrum of **4af** in $CDCl_3$, 101 MHz.

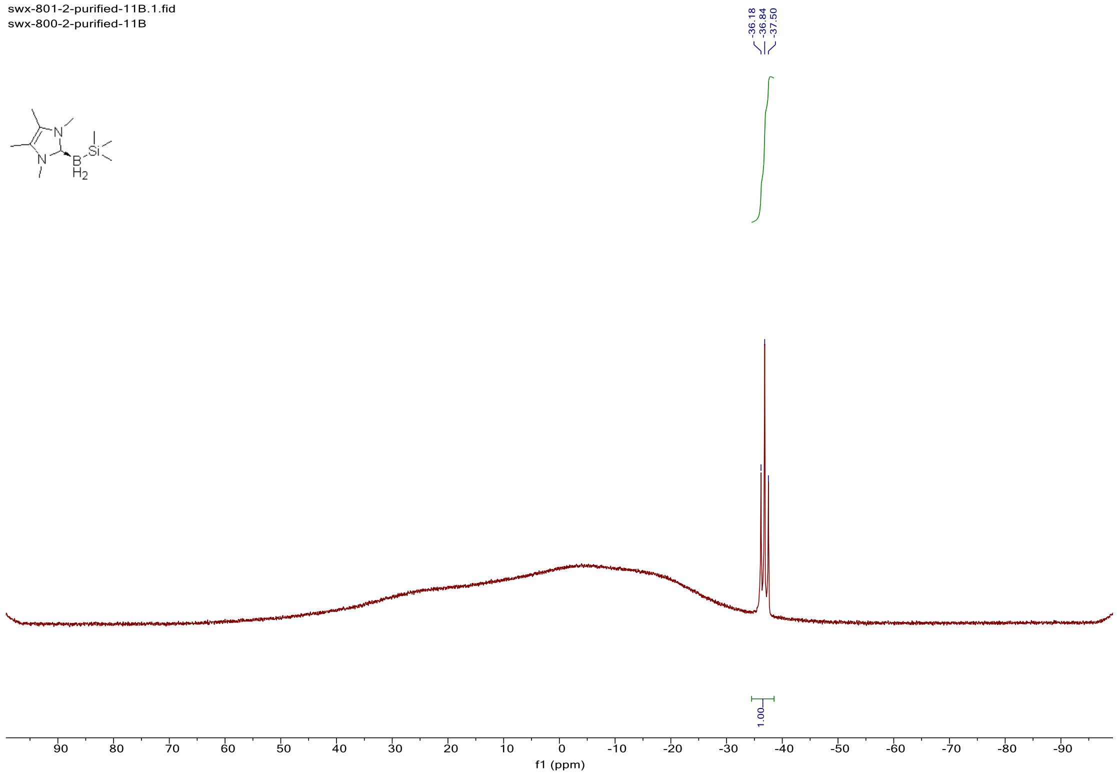

^{11}B NMR spectrum of **4af** in $CDCl_3$, 128 MHz.

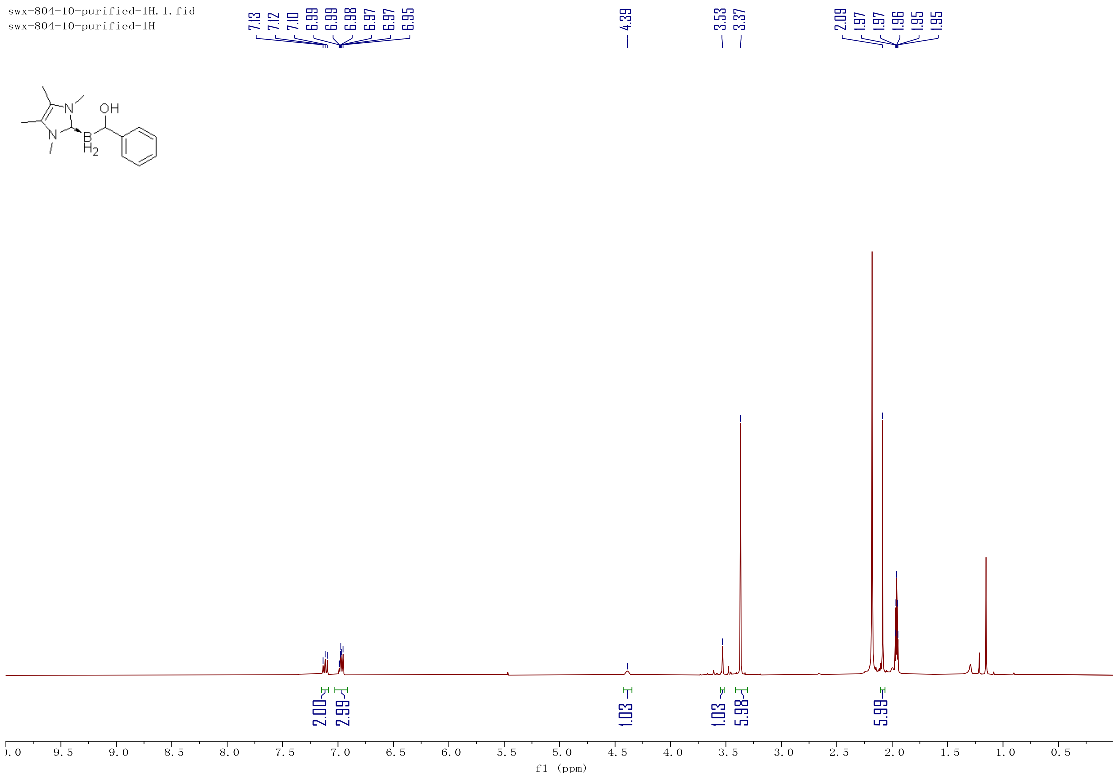

^{1}H NMR spectrum of **4ag** in CD_3CN, 400 MHz.

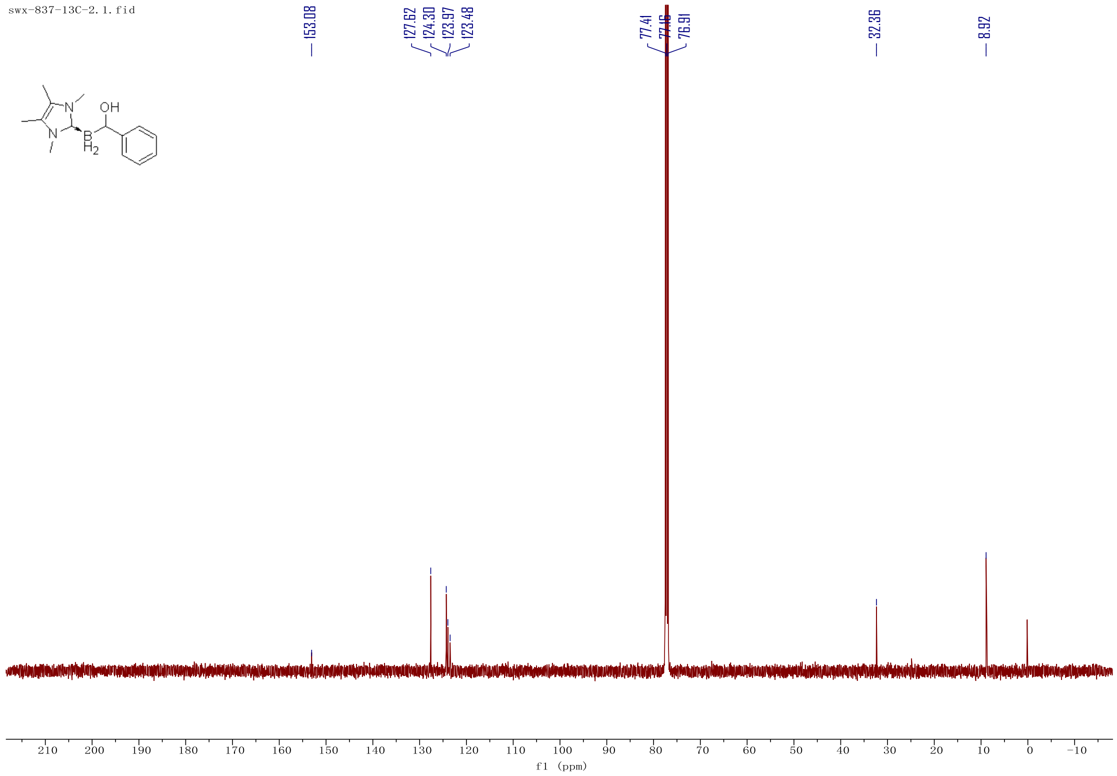

^{13}C NMR spectrum of **4ag** in $CDCl_3$, 126 MHz.

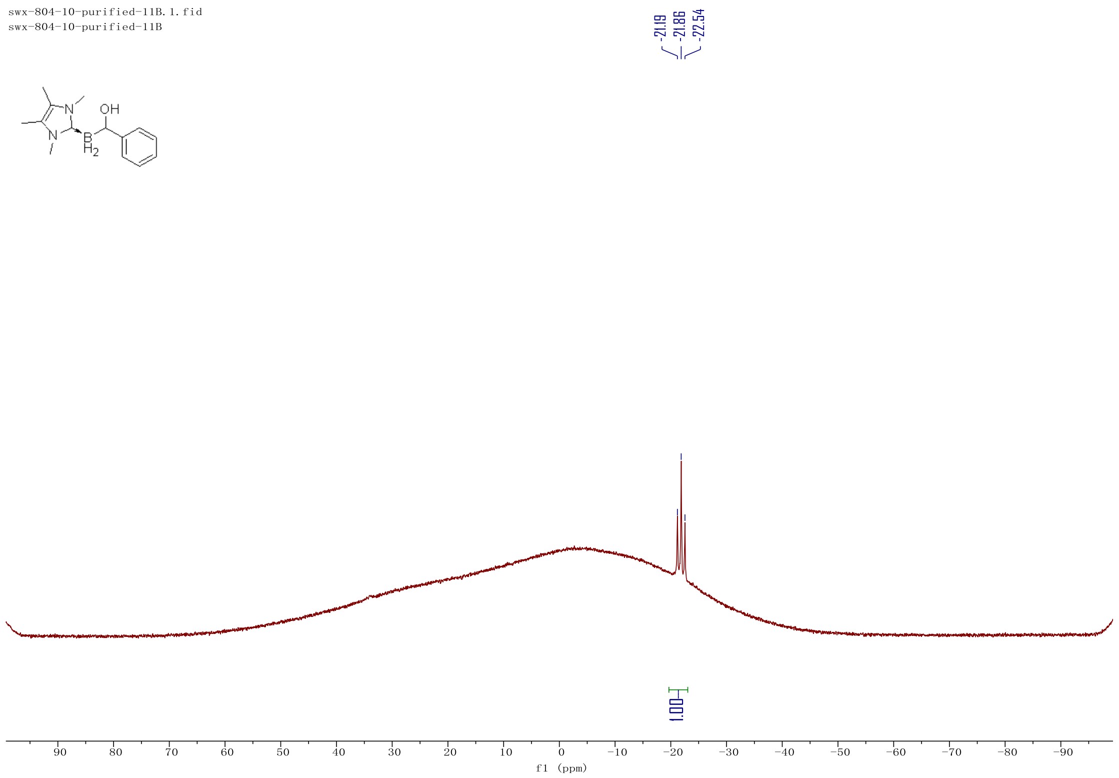

^{11}B NMR spectrum of **4ag** in CD_3CN, 128 MHz.

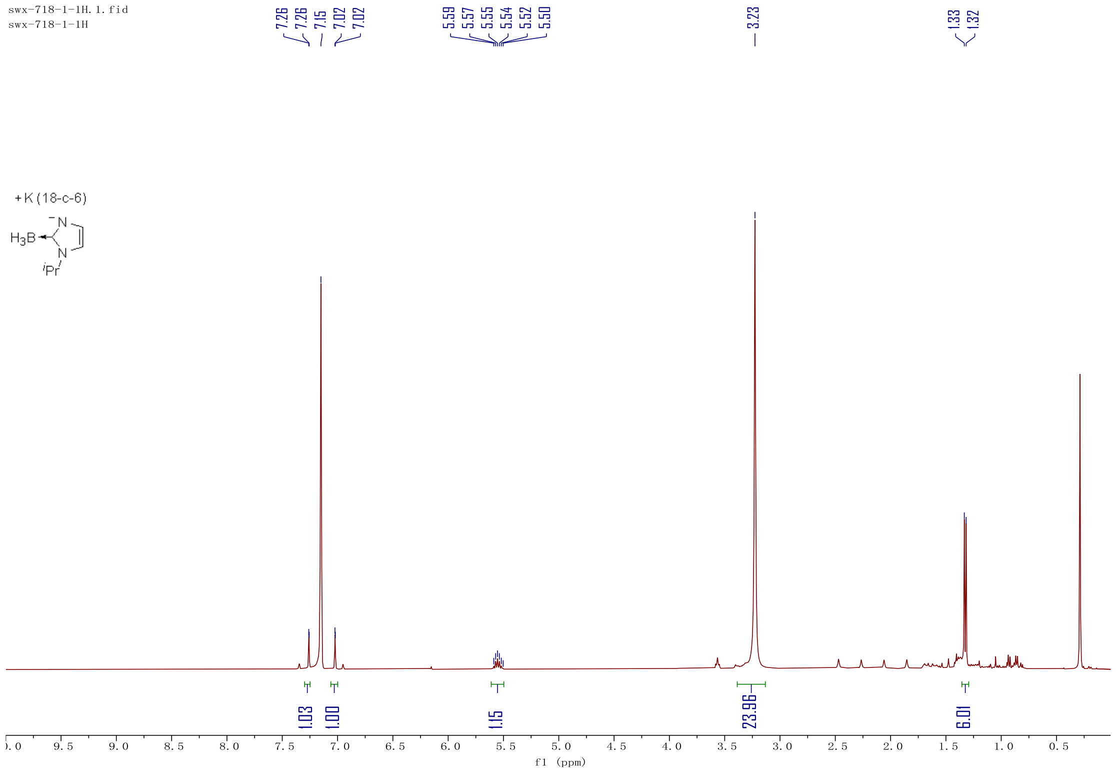

^{1}H NMR spectrum of **5a** in C_6D_6, 400 MHz.

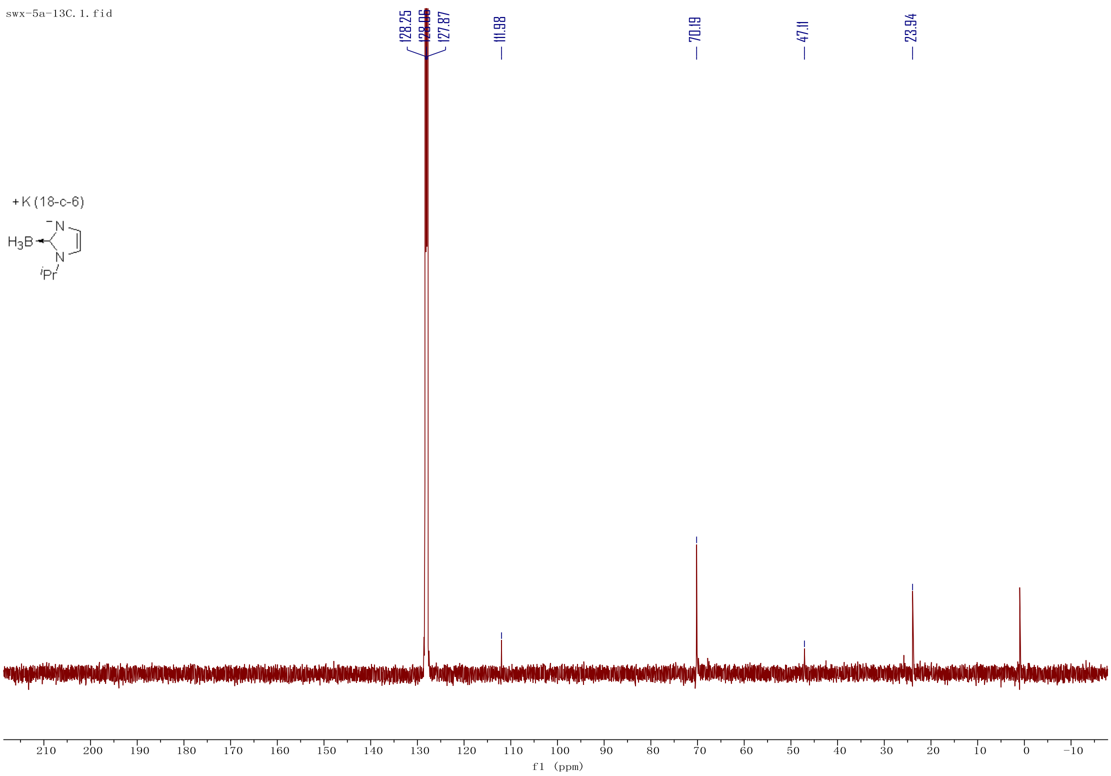

^{13}C NMR spectrum of **5a** in C_6D_6, 126 MHz.

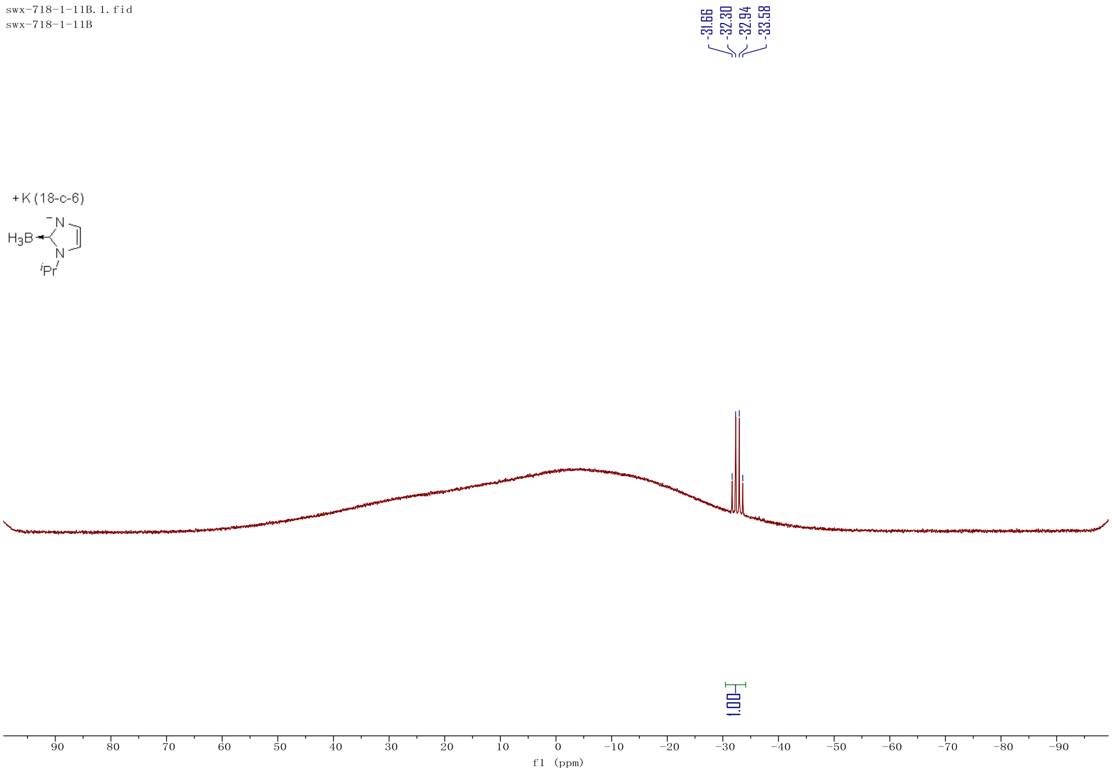

^{11}B NMR spectrum of **5a** in C_6D_6, 128 MHz.

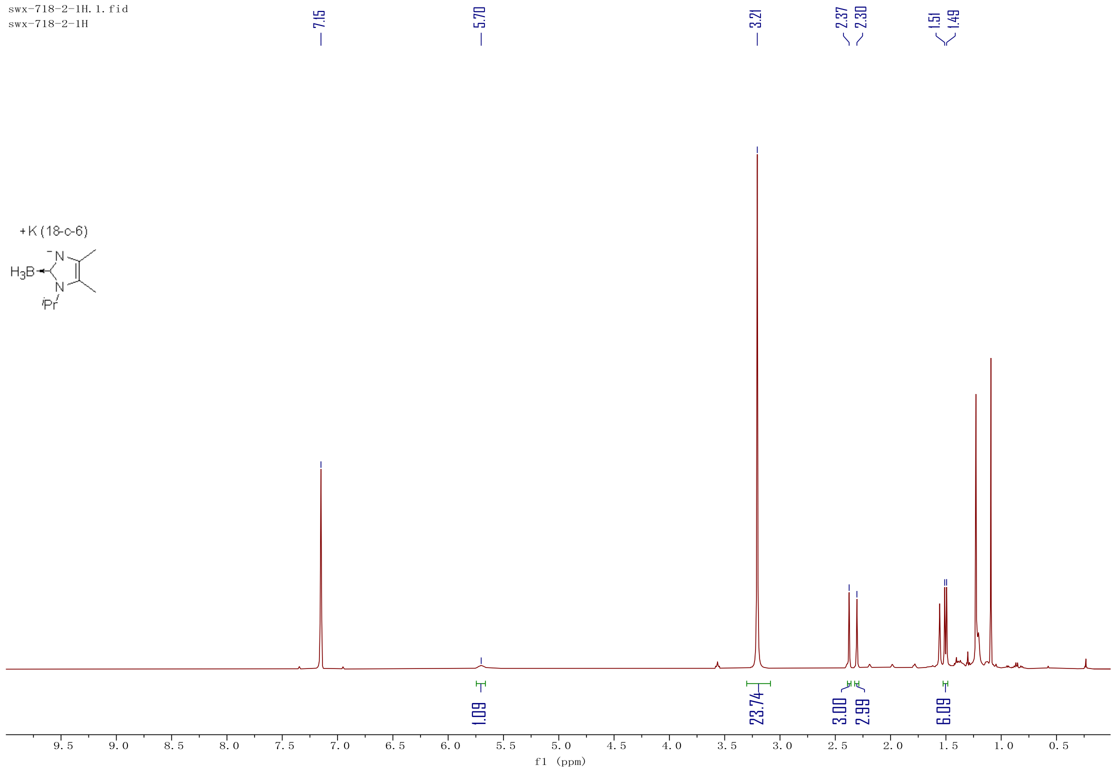

^{1}H NMR spectrum of **5b** in C_6D_6, 400 MHz.

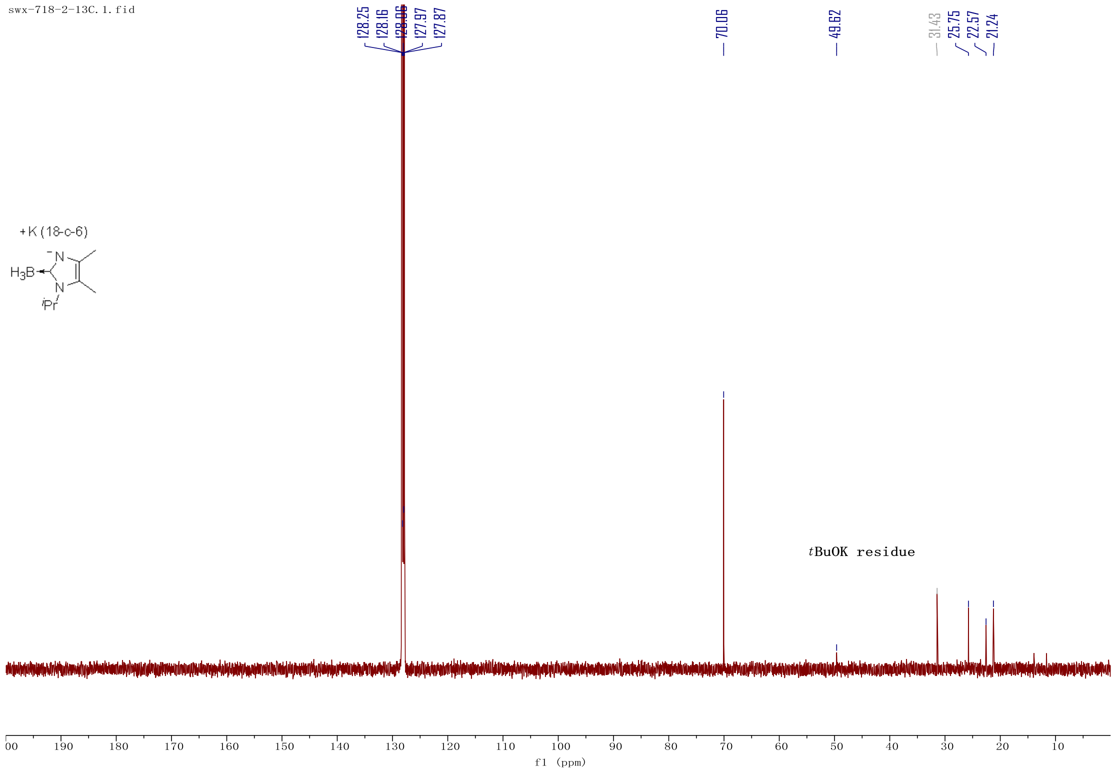

^{13}C NMR spectrum of **5b** in C_6D_6, 126 MHz.

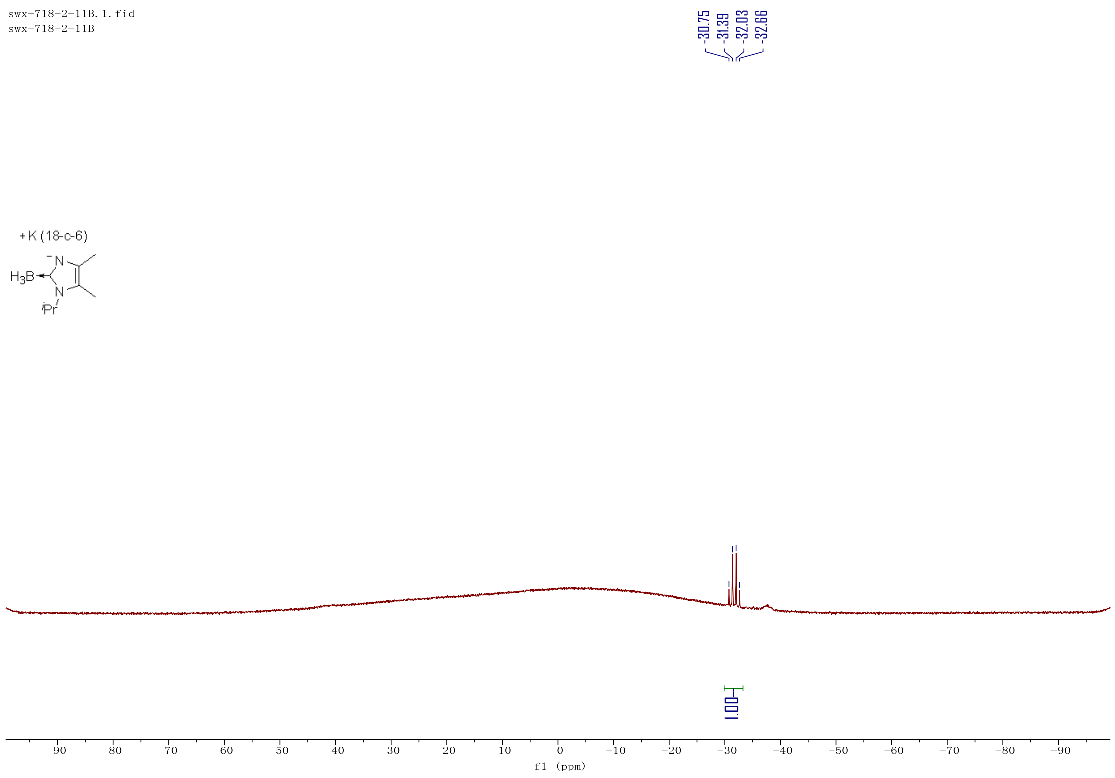

^{11}B NMR spectrum of **5b** in C_6D_6, 128 MHz.

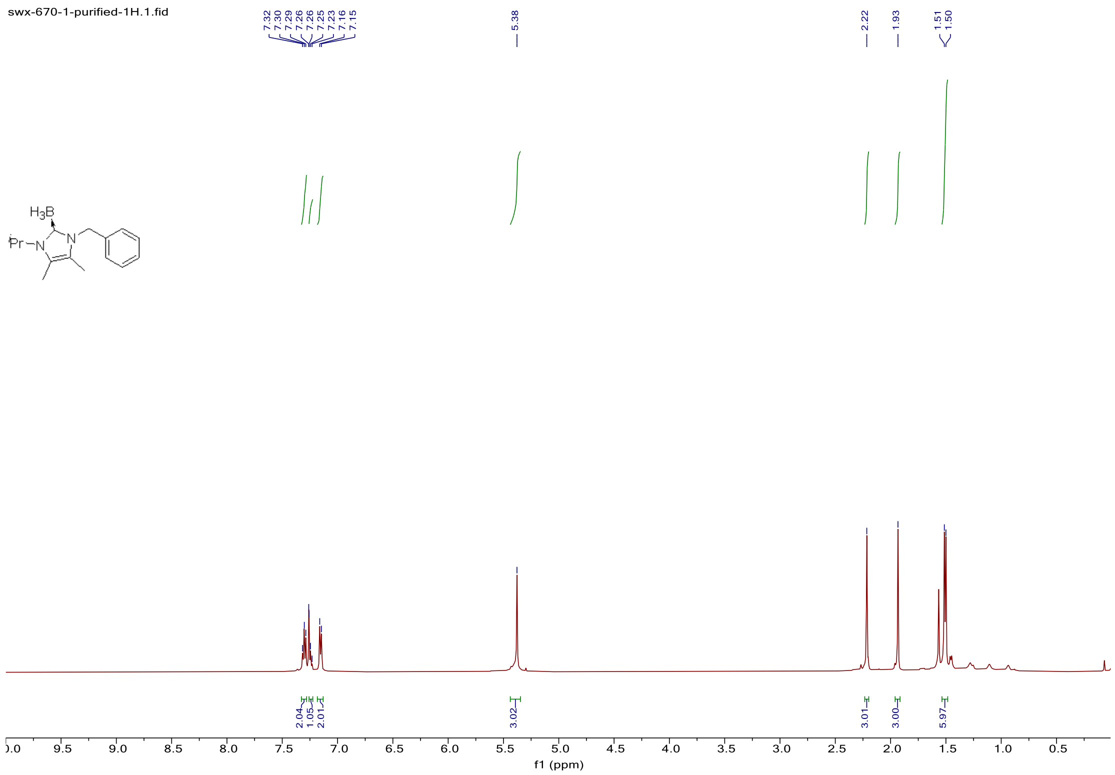

^{1}H NMR spectrum of **6** in $CDCl_3$, 500 MHz.

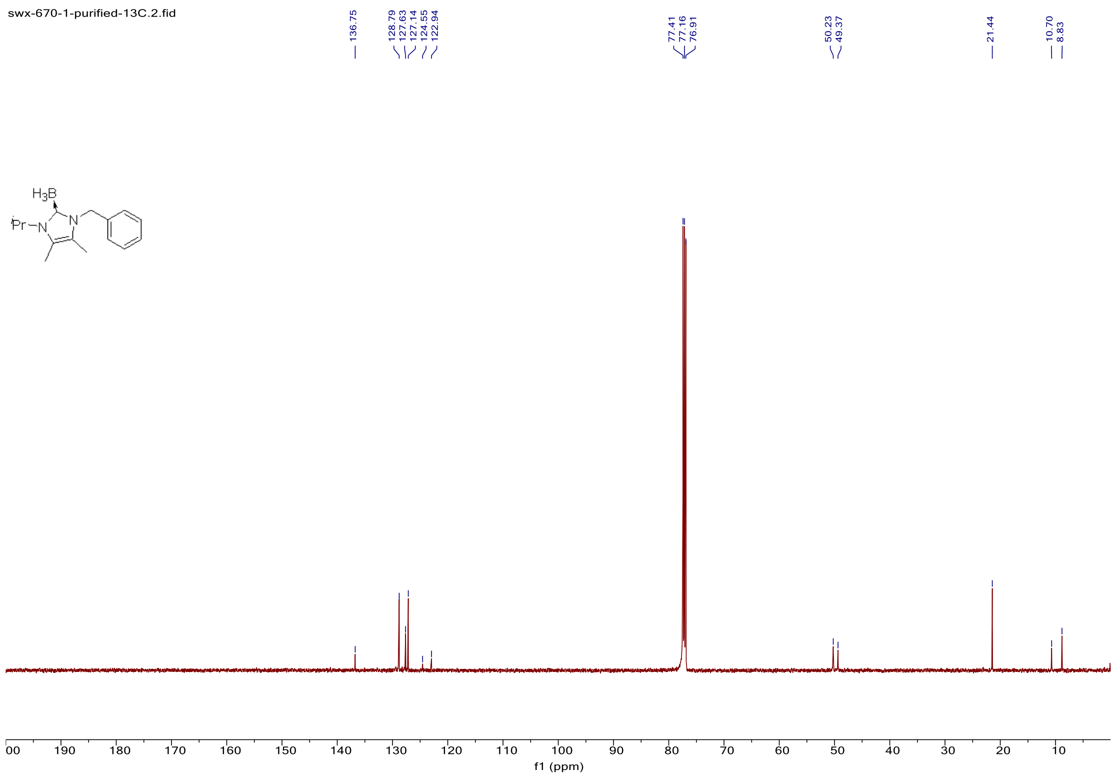

^{13}C NMR spectrum of **6** in $CDCl_3$, 126 MHz.

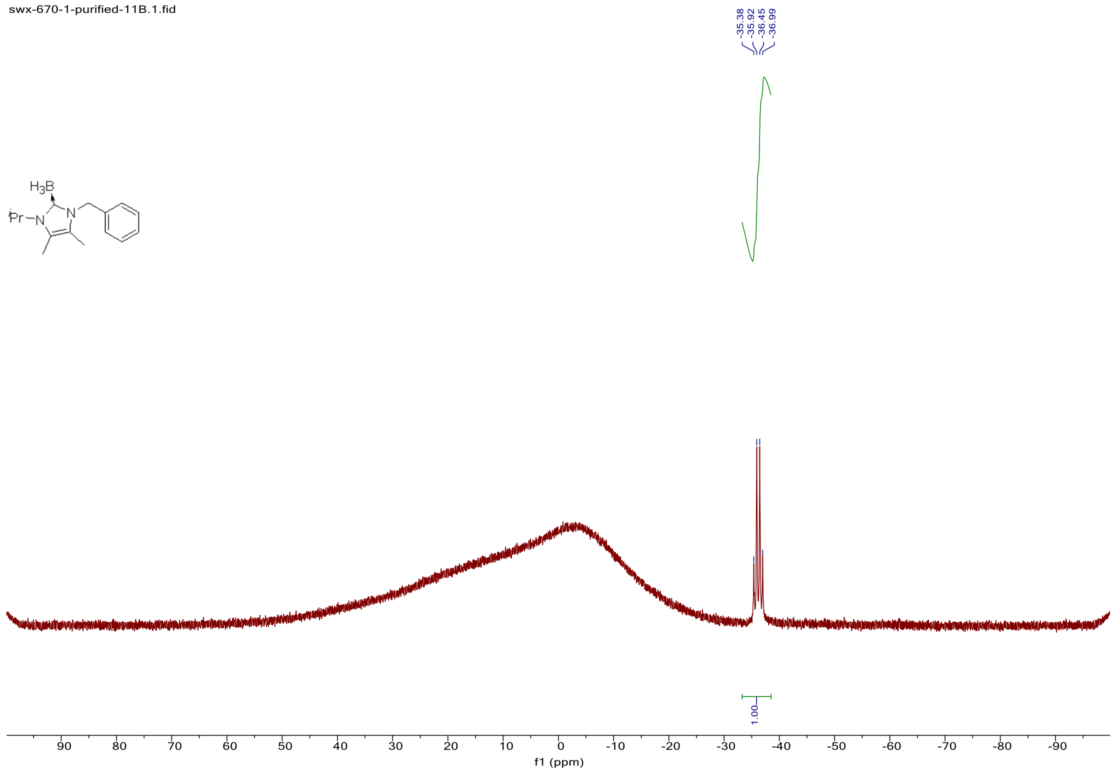

^{11}B NMR spectrum of **6** in $CDCl_3$, 160 MHz.

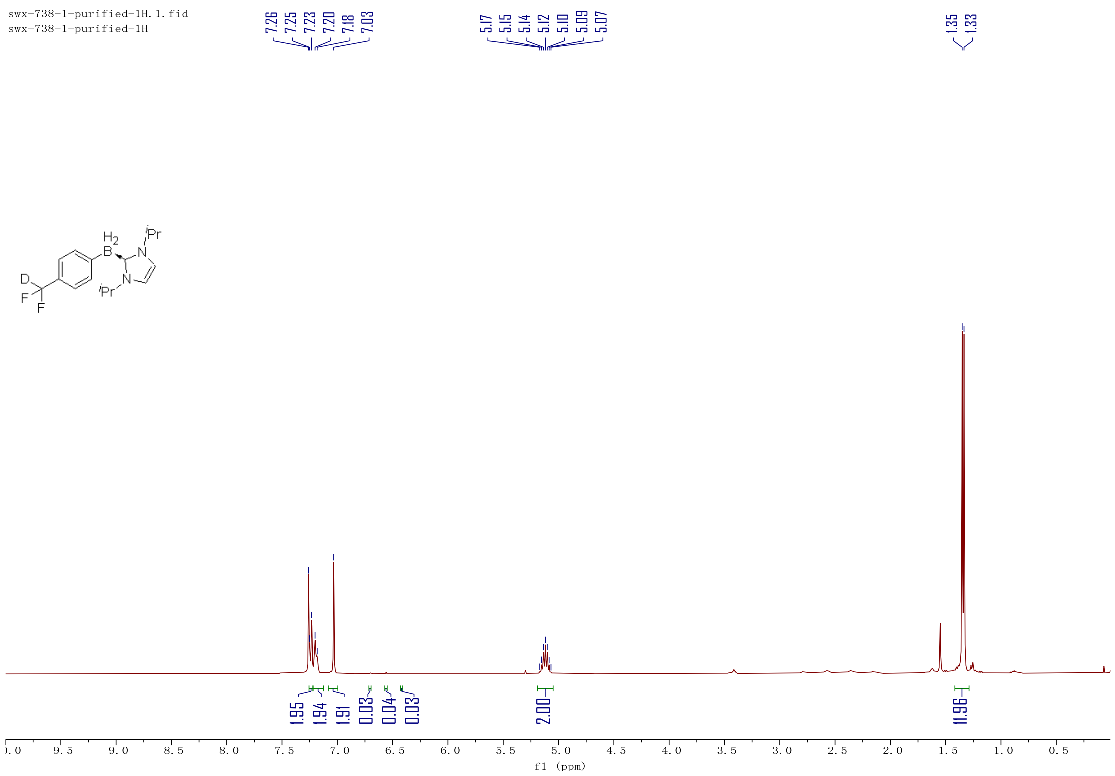

^{1}H NMR spectrum of **4j-d₁** in $CDCl_3$, 400 MHz.

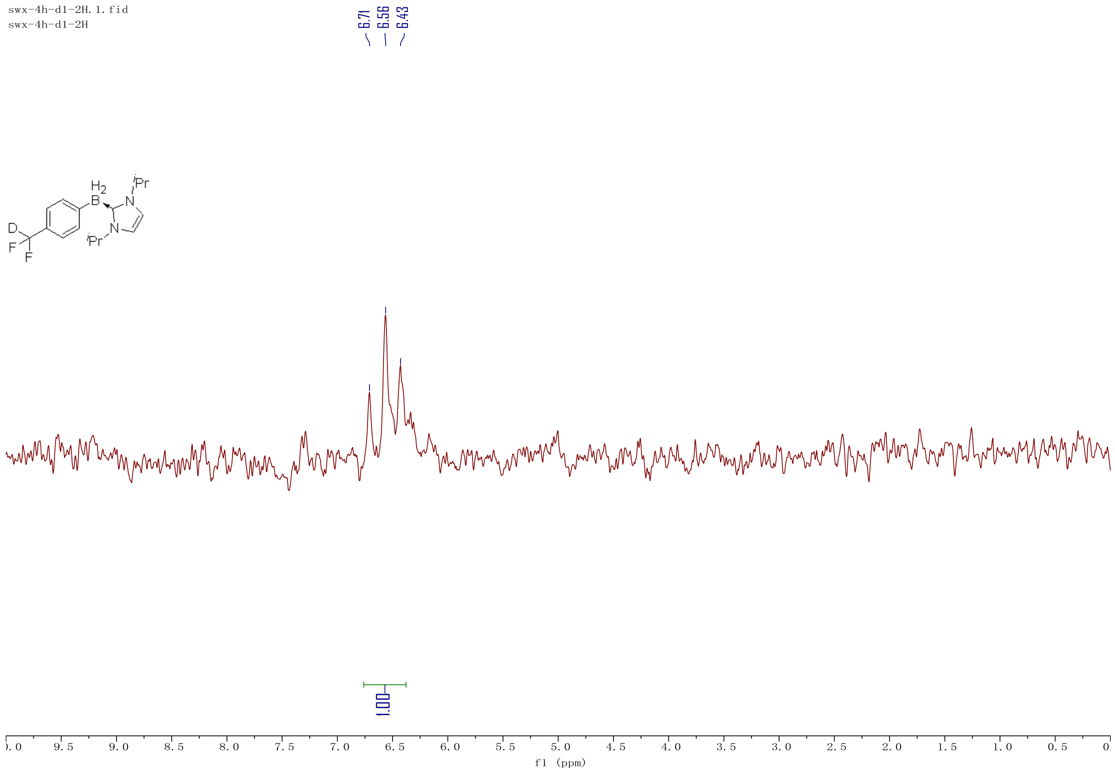

^{2}H NMR spectrum of **4j-d₁** in $CHCl_3$, 61 MHz.

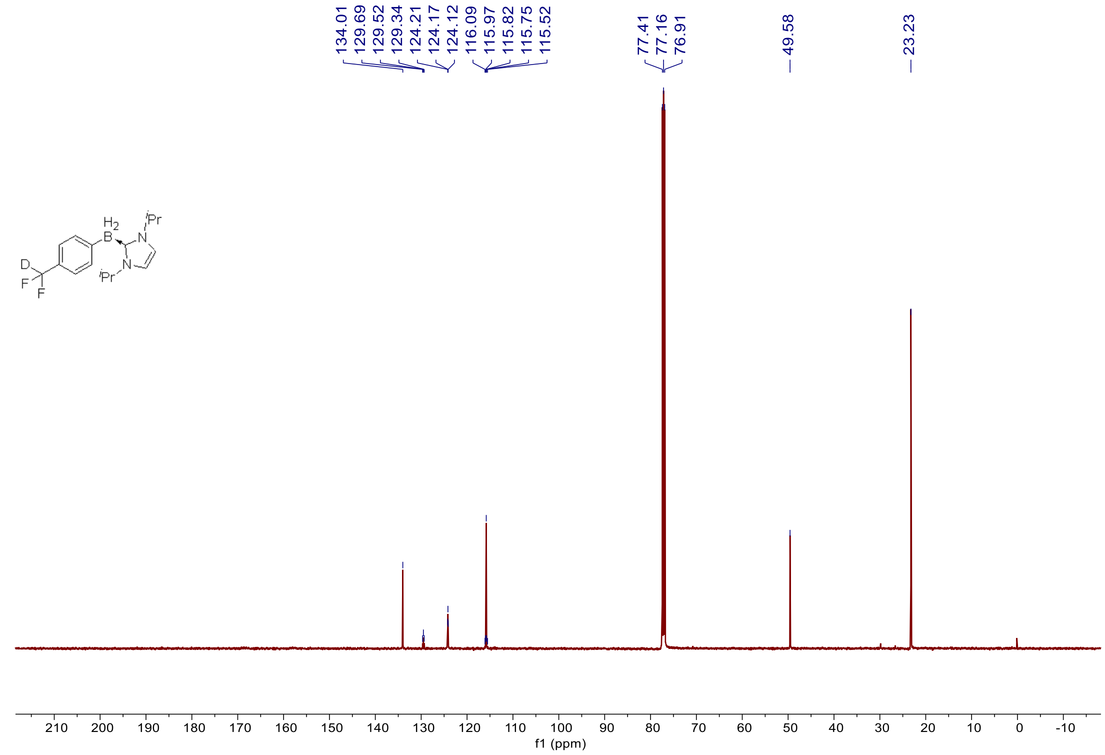

^{13}C NMR spectrum of **4j-d₁** in $CDCl_3$, 126 MHz.

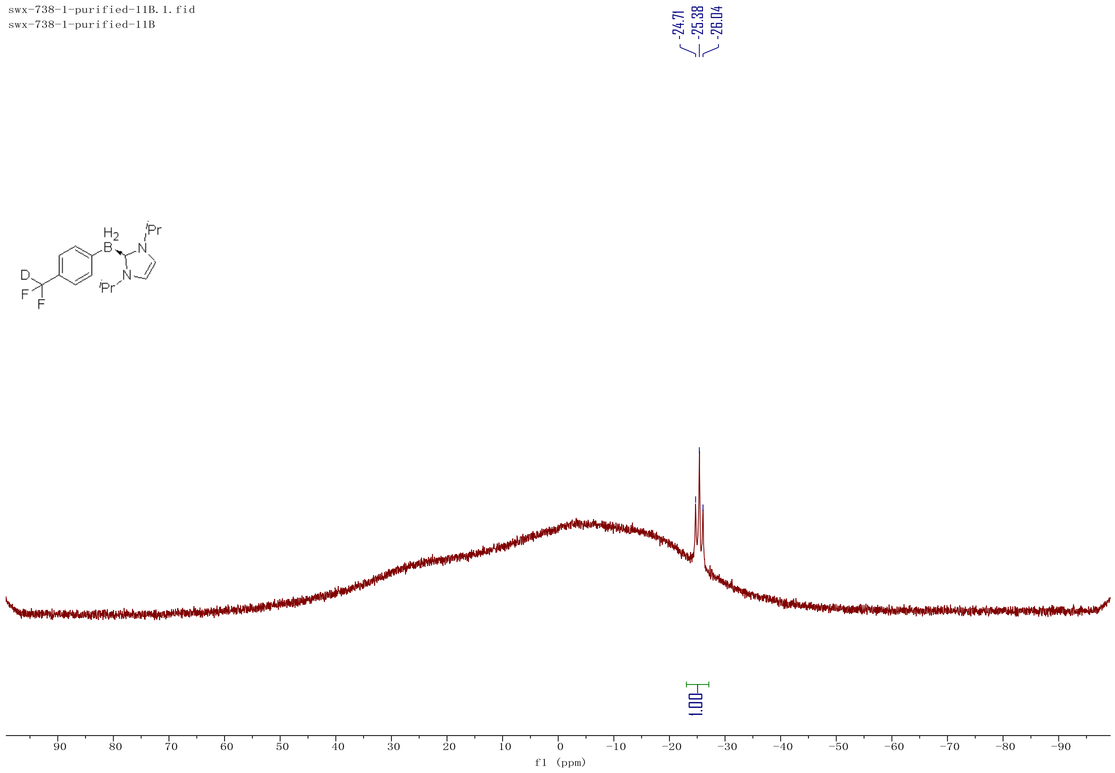

^{11}B NMR spectrum of **4j-d₁** in $CDCl_3$, 128 MHz.

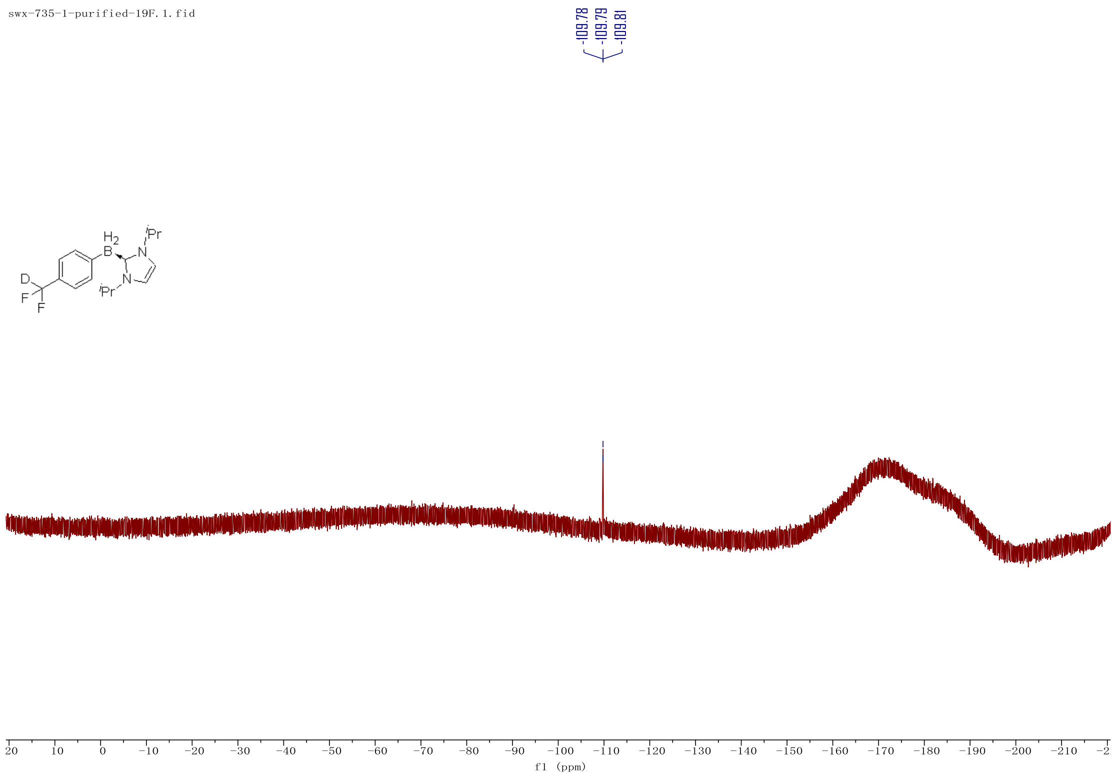

^{19}F NMR spectrum of **4j-d$_1$** in $CDCl_3$, 471 MHz.

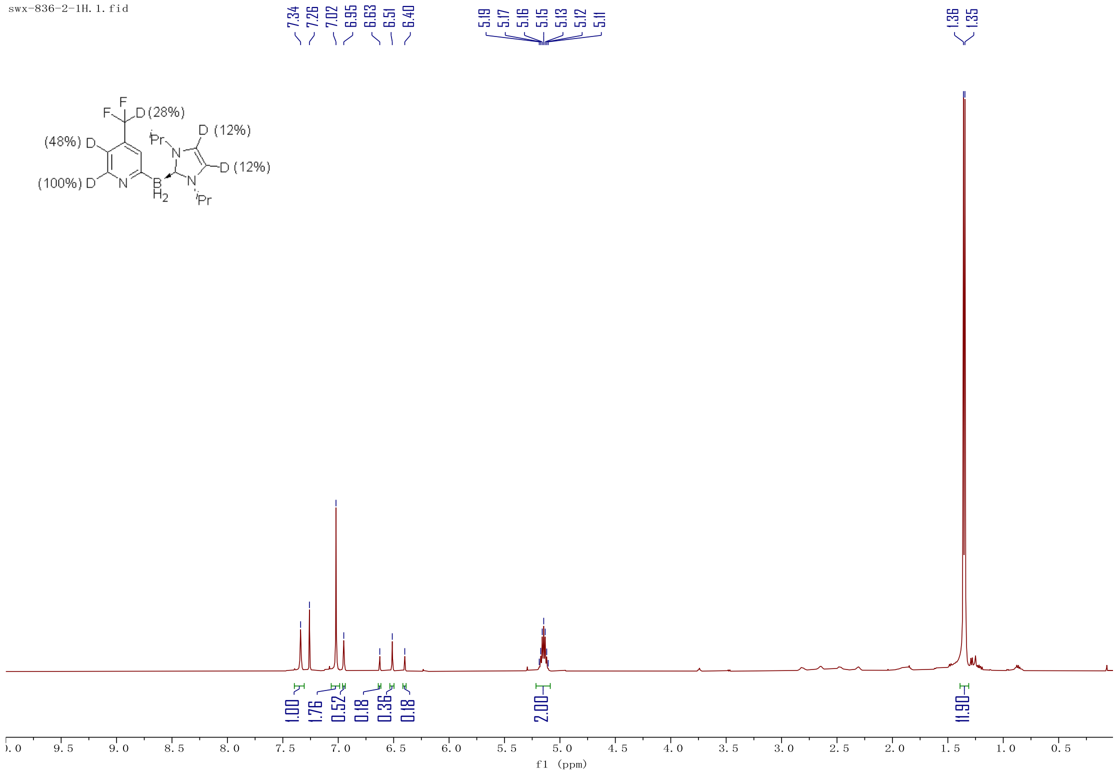

^{1}H NMR spectrum of **4l-d$_2$** in $CDCl_3$, 500 MHz.

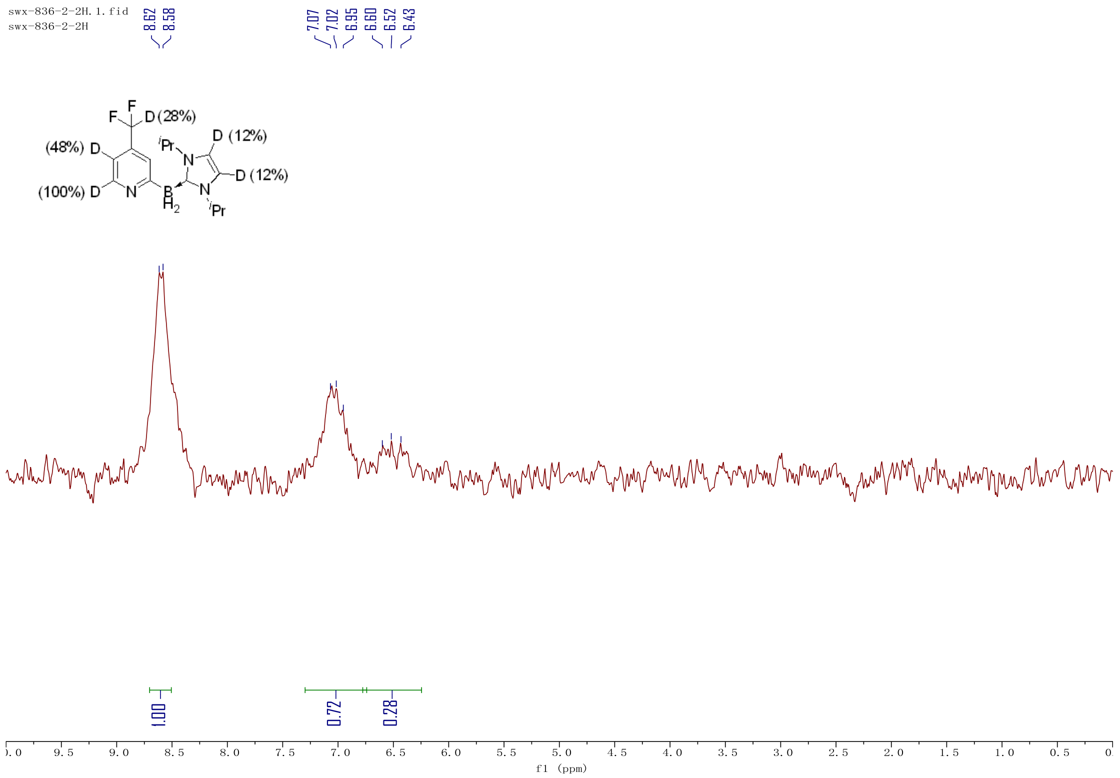

^{2}H NMR spectrum of **4l-d₂** in $CHCl_3$, 61 MHz.

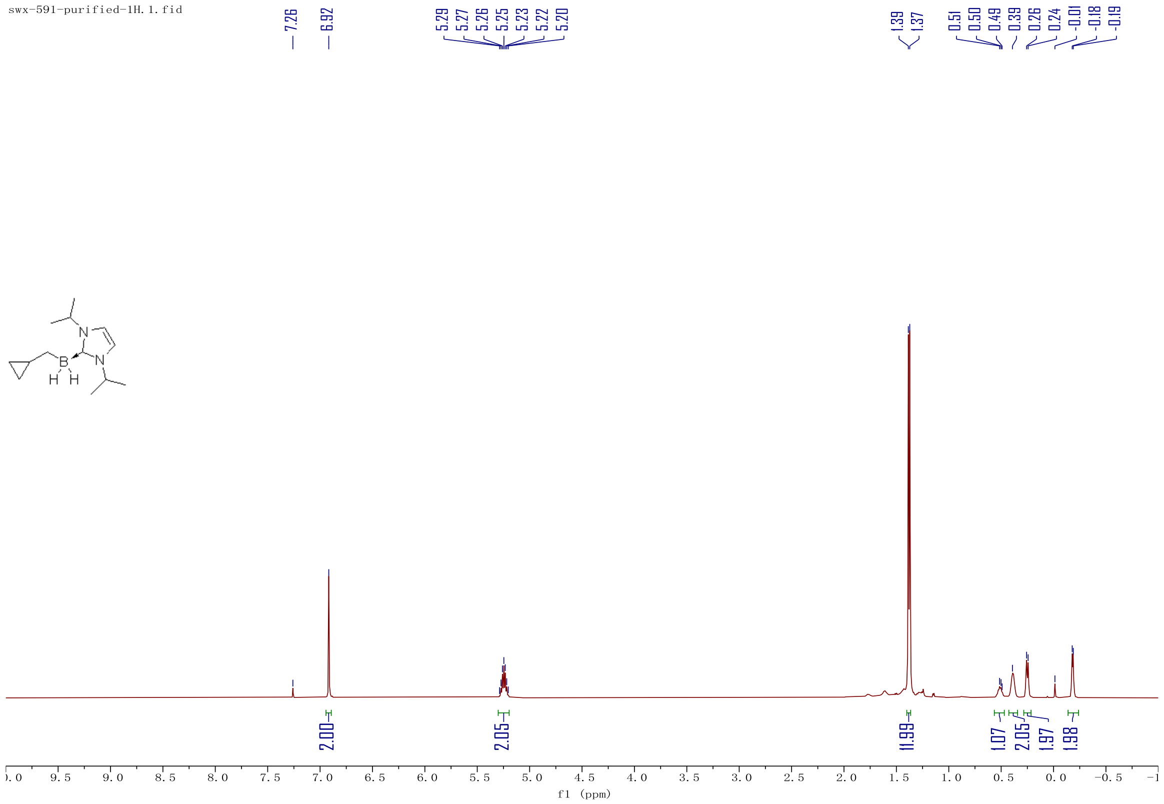

^{1}H NMR spectrum of **4ah** in $CDCl_3$, 500 MHz.

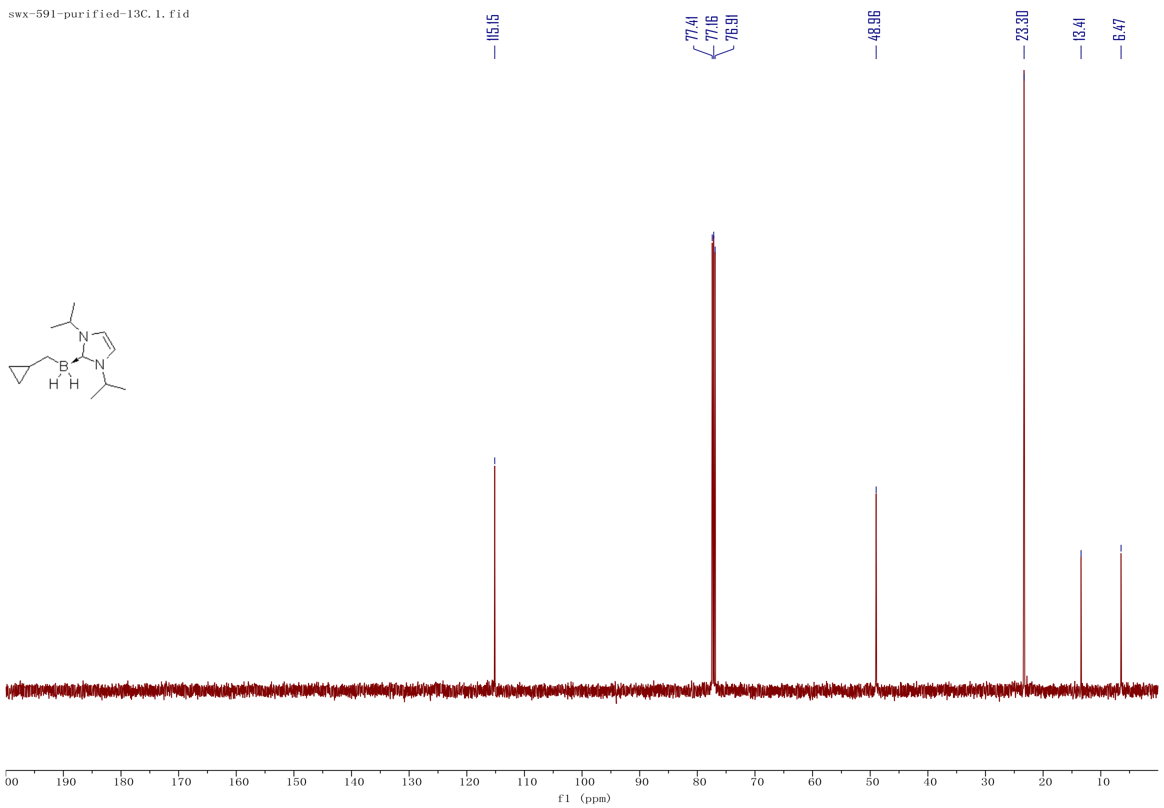

^{13}C NMR spectrum of **4ah** in $CDCl_3$, 126 MHz.

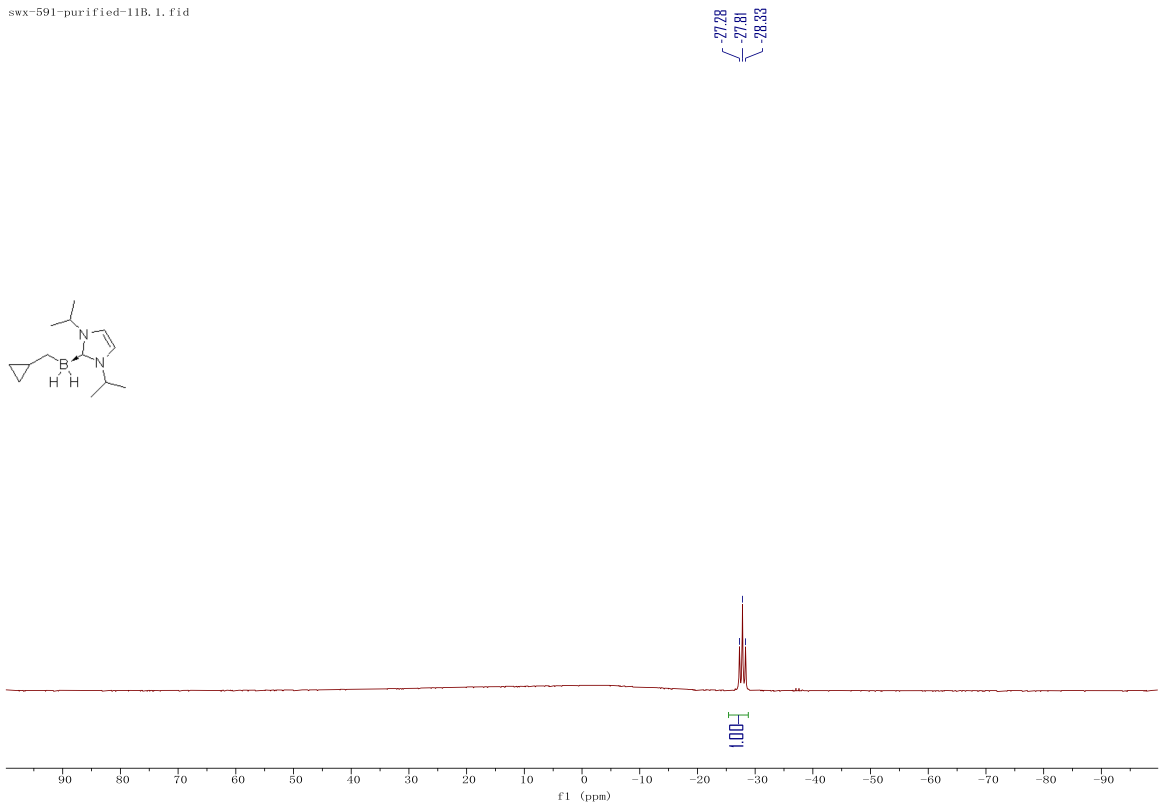

^{11}B NMR spectrum of **4ah** in $CDCl_3$, 160 MHz.

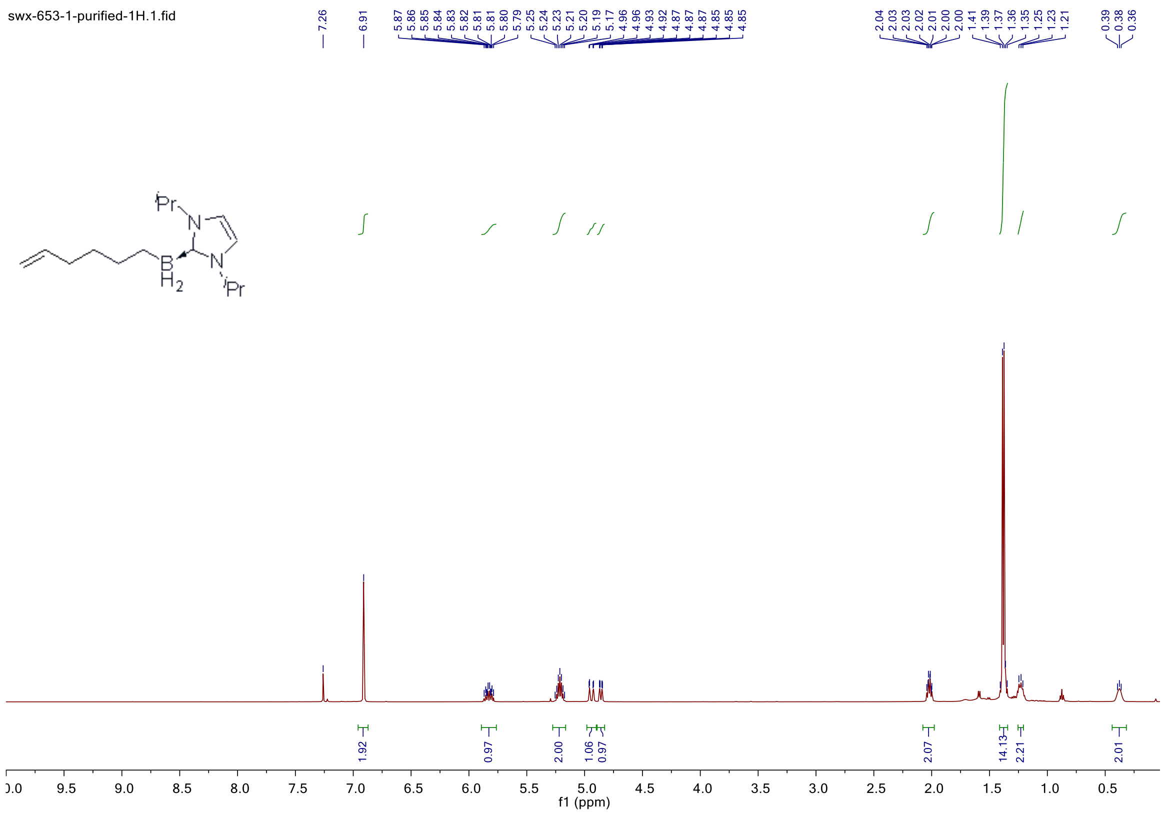

^{1}H NMR spectrum of **4ai** in $CDCl_3$, 500 MHz.

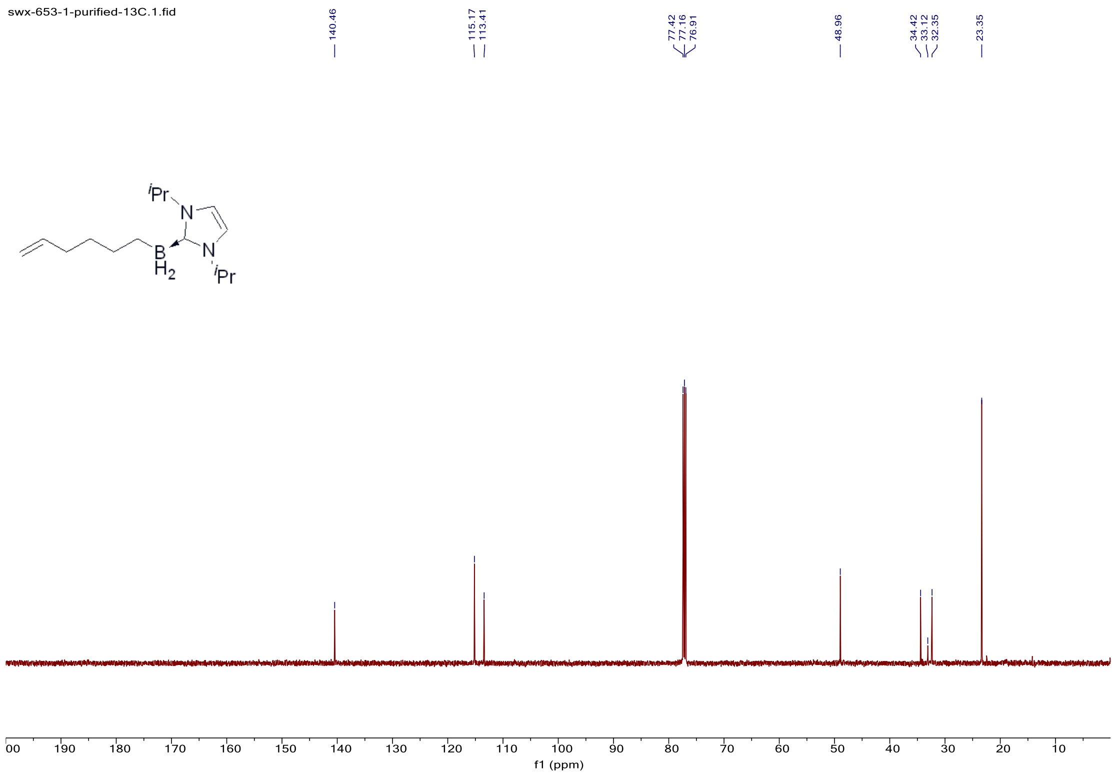

^{13}C NMR spectrum of **4ai** in $CDCl_3$, 126 MHz.

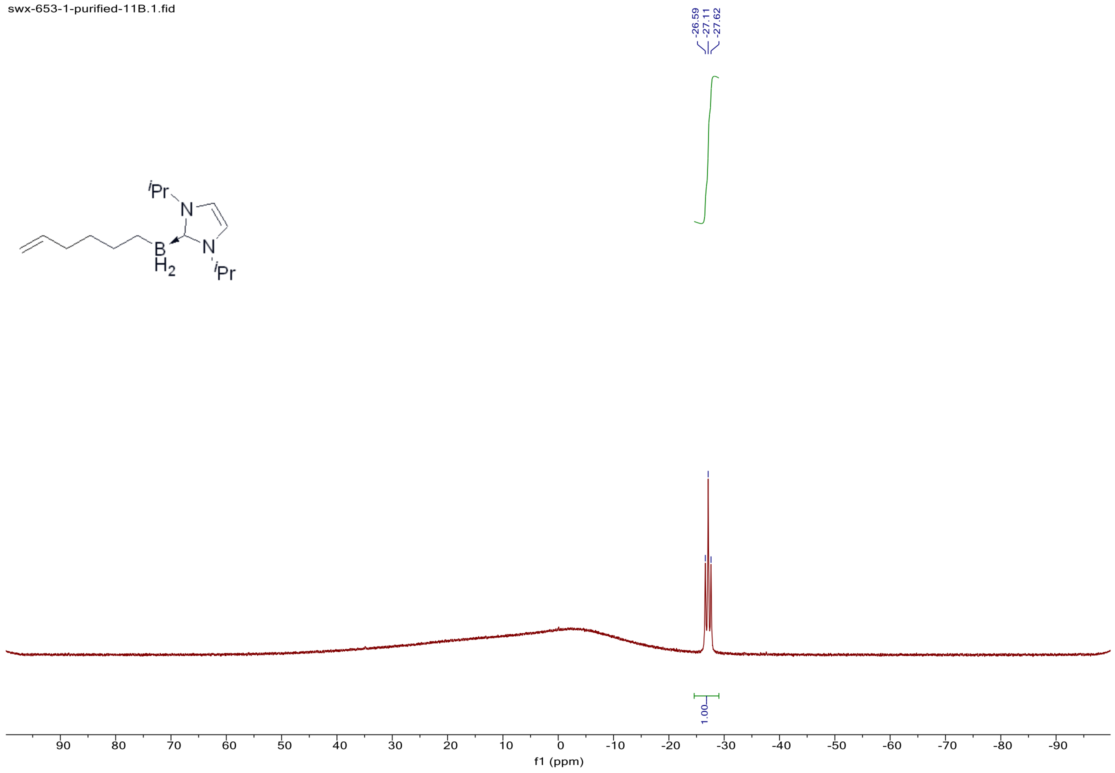

^{11}B NMR spectrum of **4ai** in $CDCl_3$, 160 MHz.

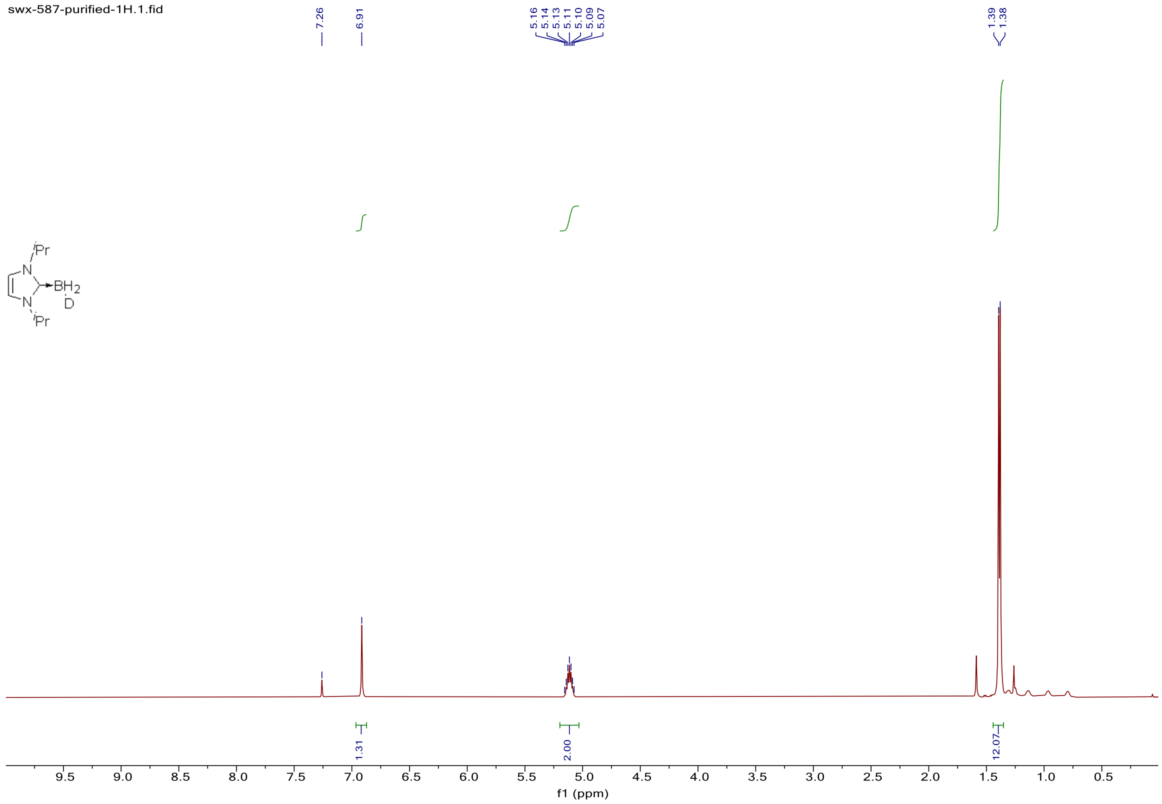

^{1}H NMR spectrum of **8** in $CDCl_3$, 500 MHz.

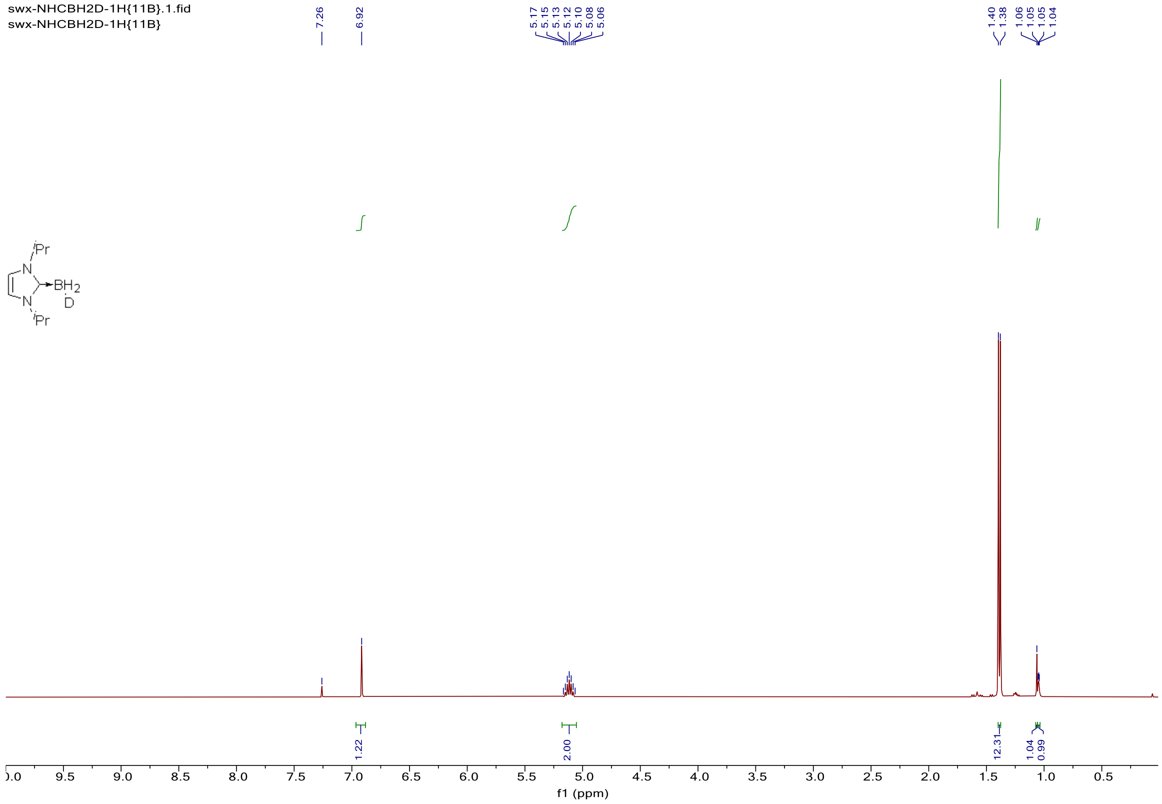

$^1H\{^{11}B\}$ NMR spectrum of **8** in $CDCl_3$, 400 MHz.

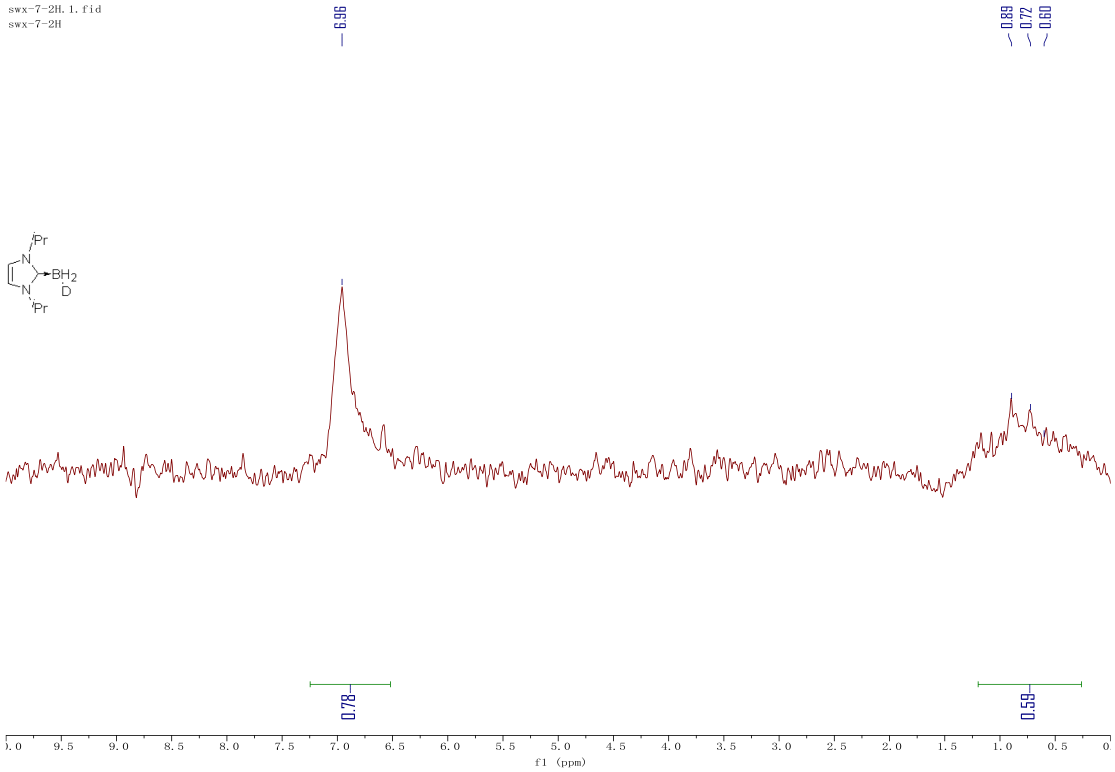

^2H NMR spectrum of **8** in $CHCl_3$, 61 MHz.

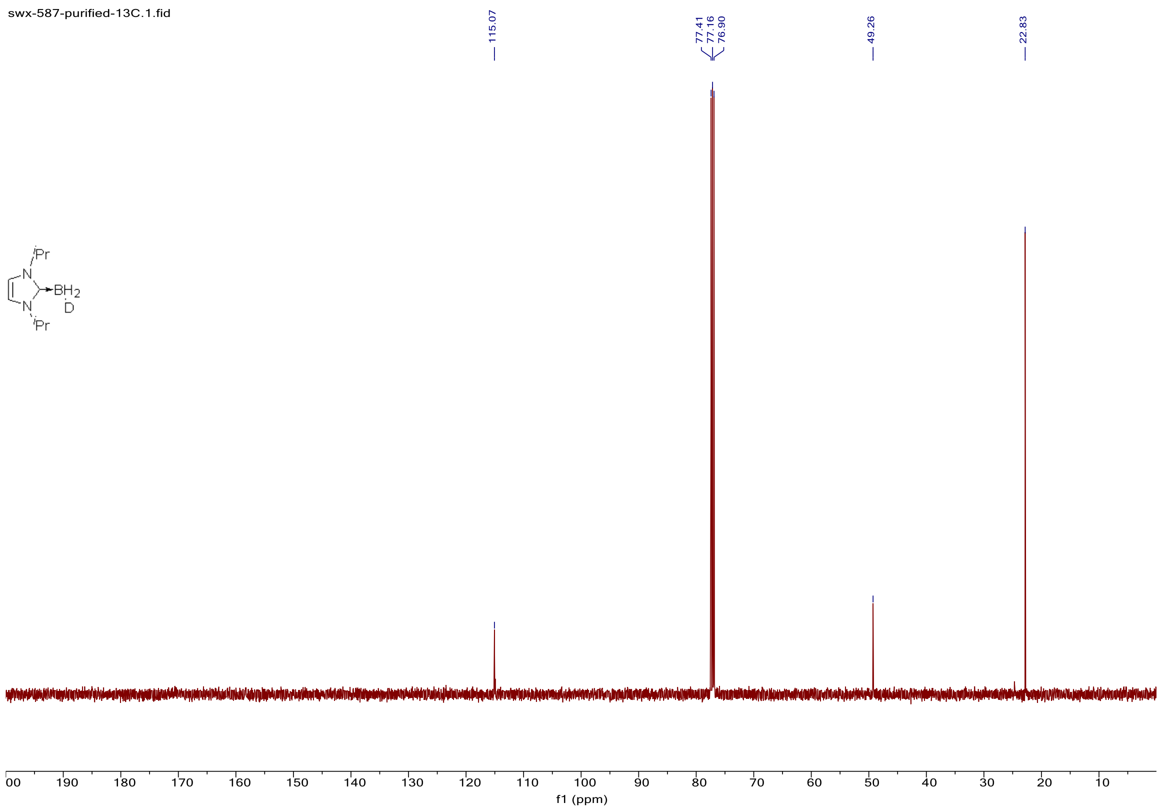

^{13}C NMR spectrum of **8** in $CDCl_3$, 126 MHz.

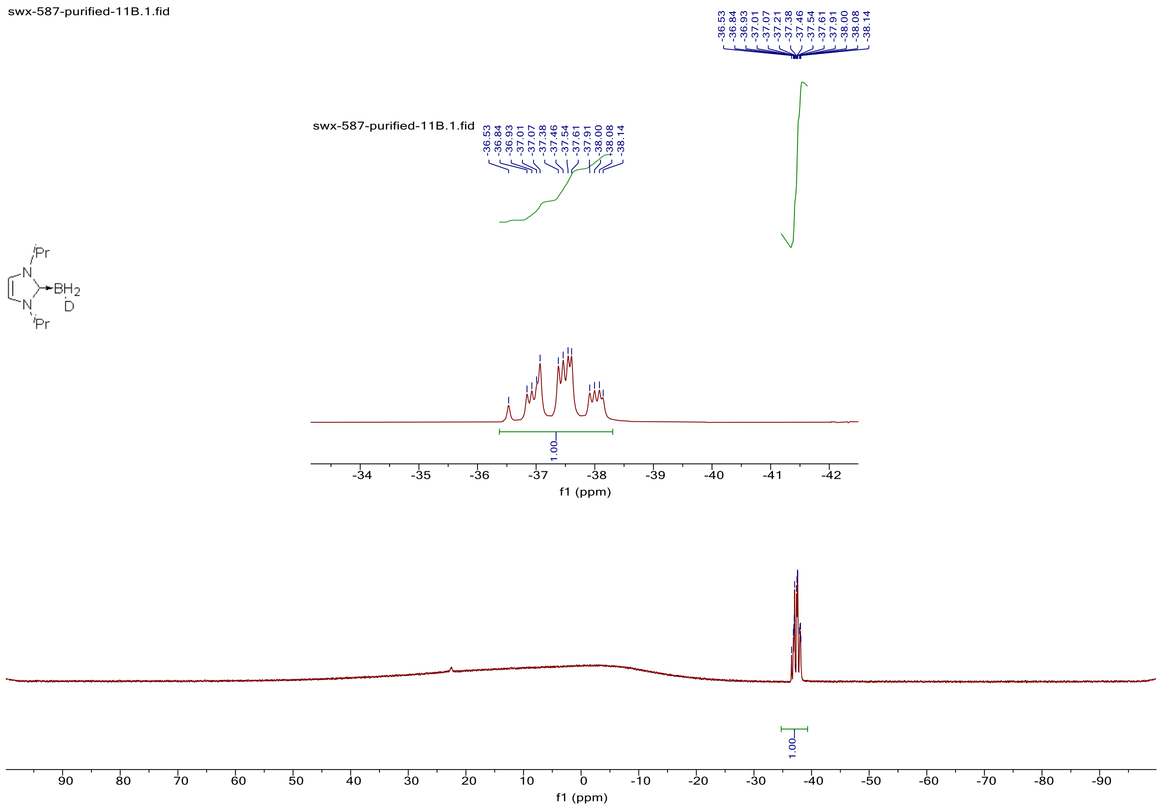

^{11}B NMR spectrum of **8** in $CDCl_3$, 160 MHz.

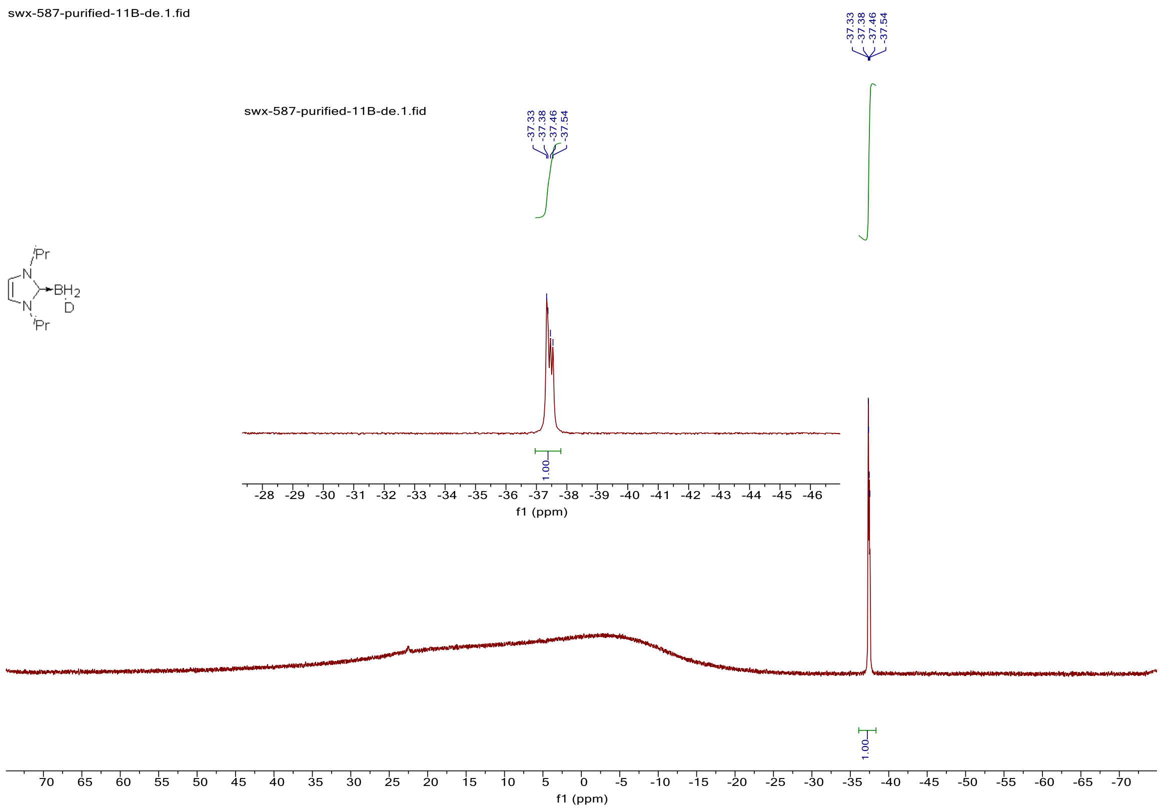

$^{11}B\{^{1}H\}$ NMR spectrum of **8** in $CDCl_3$, 160 MHz.

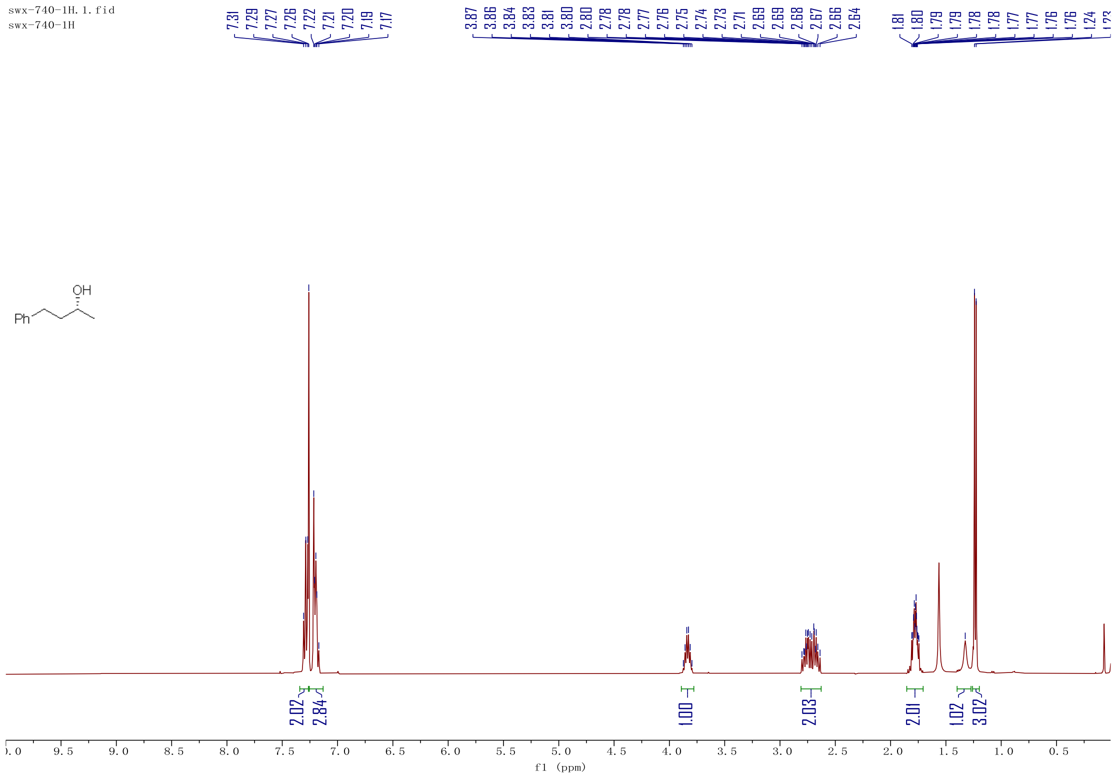

^{1}H NMR spectrum of (*R*)-**3d’** in $CDCl_3$, 400 MHz.

10. HPLC Analysis of Enantioenriched Substrates

HPLC-UV chromatogram of the racemic sample of **3d** (injection volume 10 μL):

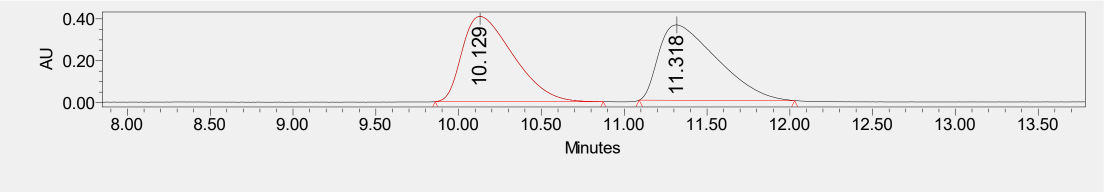

	Retention Time	Area	% Area
1	10.129	8706763	49.75
2	11.318	8795346	50.25

HPLC-UV chromatogram of the enantioenriched sample of (*S*)-**3d** (injection volume 10 μL):

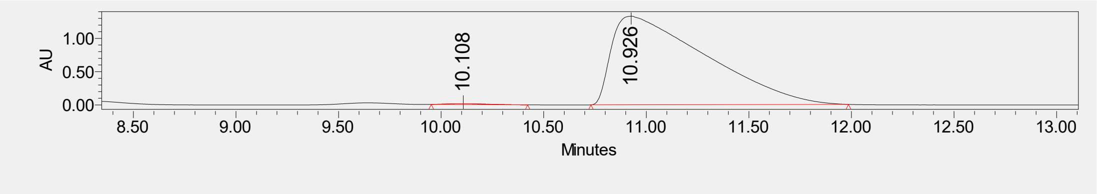

	Retention Time	Area	% Area
1	10.108	168786	0.39
2	10.926	42753360	99.61

Separation conditions: concentration 1 mg/mL n-hexane, Column Chiralcel OJ-3, eluent n-hexane, temperature 20 °C, flow 1.0 mL/min, detected wavelength 210 nm.

HPLC-UV chromatogram of the racemic sample of **4d** (injection volume 10 μL):

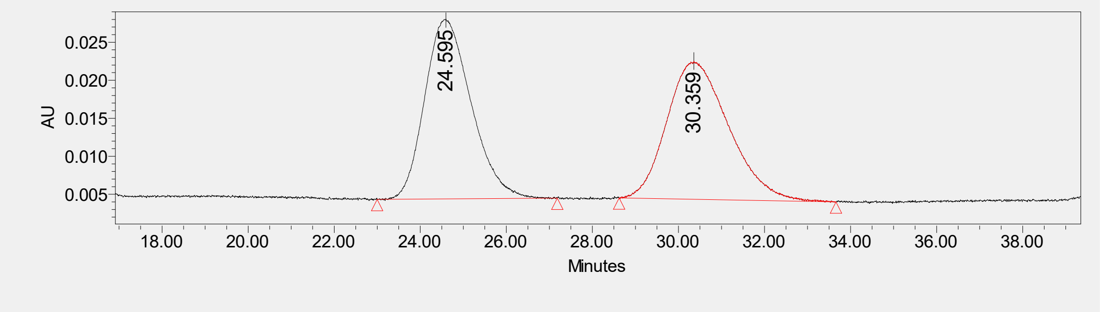

	Retention Time	Area	% Area
1	24.595	1771580	49.52
2	30.359	1806165	50.48

HPLC-UV chromatogram of the enantioenriched sample of (*R*)-**4d** (injection volume 10 μL):

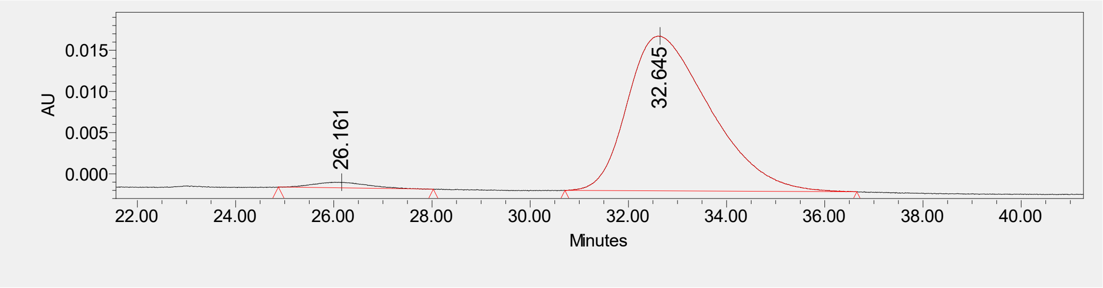

	Retention Time	Area	% Area
1	26.161	51805	2.31
2	32.645	2194729	97.69

Separation conditions: concentration 1 mg/mL n-hexane/isopropanol = 95/5, Column Chiralpak AD-H, eluent n-hexane/isopropanol = 95/5, temperature 20 °C, flow 1.0 mL/min, detected wavelength 234 nm.

HPLC-UV chromatogram of the racemic sample of **3d'** (injection volume 2 μL):

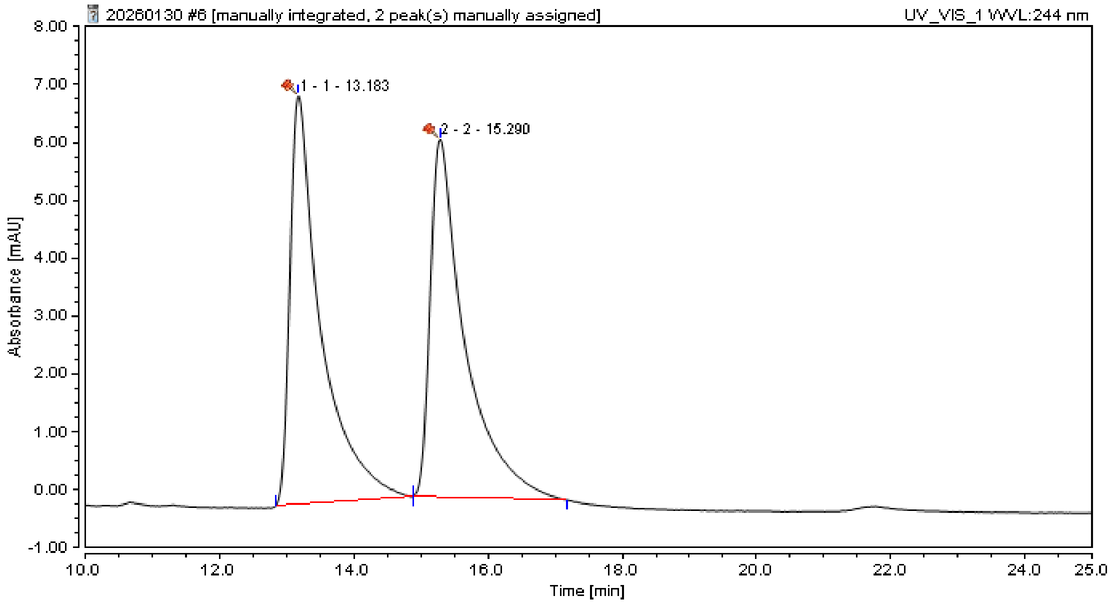

No.	Peak name	Retention Time min	Area mAU*min	Relative Area %
1	1	13.183	3.7310	50.41
2	2	15.290	3.6700	49.59

HPLC-UV chromatogram of the enantioenriched commercially available sample of (*R*)-**3d'** (injection volume 2 μL):

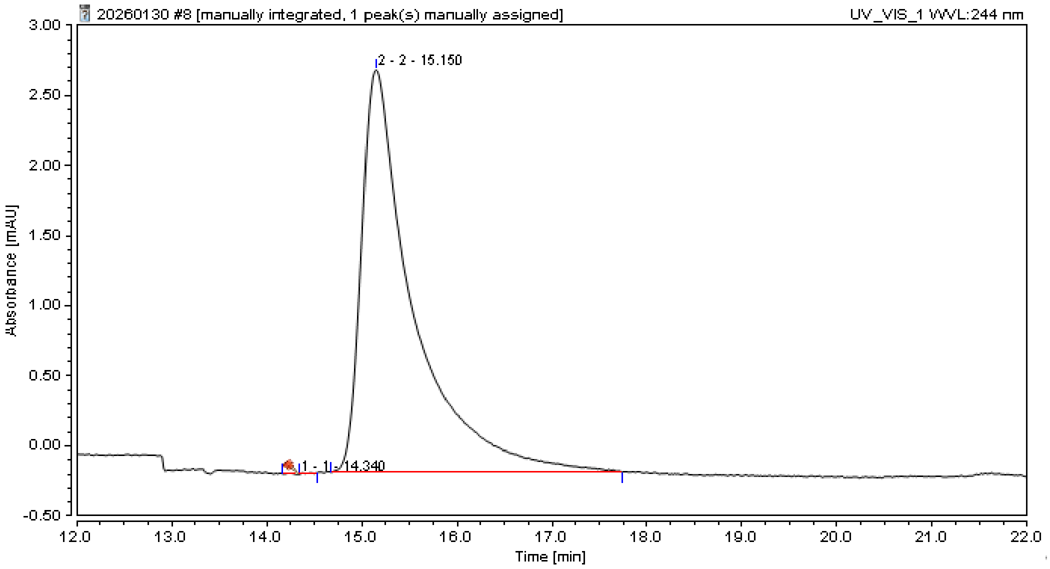

No.	Peak name	Retention Time<br>min	Area<br>mAU*min	Relative Area<br>%
1	1	14.340	0.0000	0.03
2	2	15.150	1.8020	99.97

HPLC-UV chromatogram of the oxidation product of (*R*)-**4d** ((*R*)-**3d'**) (injection volume 2 μL):

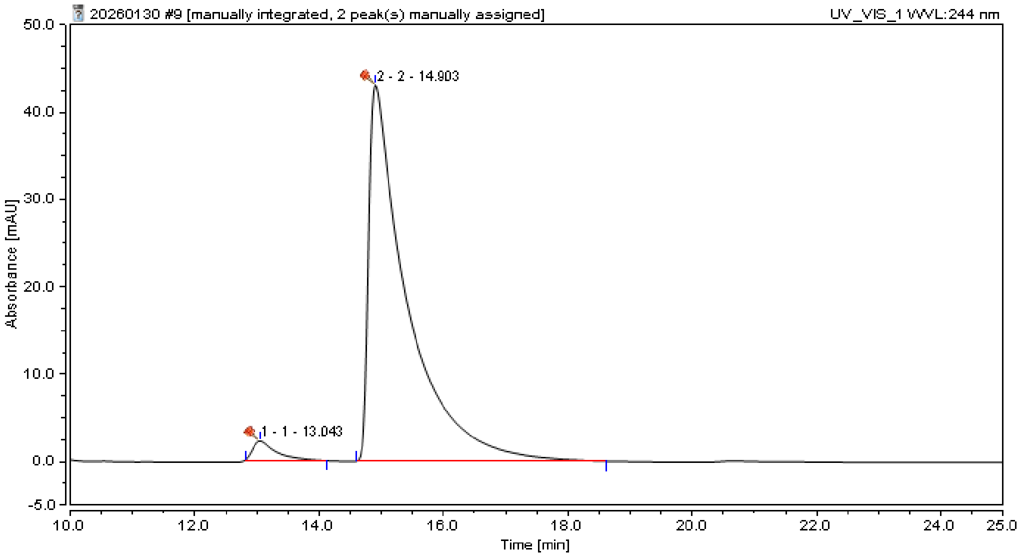

No.	Peak name	Retention Time<br>min	Area<br>mAU*min	Relative Area<br>%
1	A	13.043	0.9420	2.99
2	B	14.903	30.5650	97.01

Separation conditions: concentration 1 mg/mL n-hexane/isopropanol = 95/5, Column Chiralcel OX-H, eluent n-hexane/isopropanol = 98/2, temperature 20 °C, flow 1.0 mL/min, detected wavelength 244 nm.

11. References

[37] Y. Segawa, M. Yamashita, K. Nozaki, *Science* **2006**, *314*, 113.

[39] M. Arrowsmith, J. D. Mattock, S. Hagspiel, I. Krummenacher, A. Vargas, H. Braunschweig, *Angew. Chem., Int. Ed.* **2018**, *57*, 15272.

[51] D. A. Ruiz, G. Ung, M. Melaimi, G. Bertrand, *Angew. Chem., Int. Ed.* **2013**, *52*, 7590.

[54] J. Monot, A. Solovyev, H. Bonin-Dubarle, É. Derat, D. P. Curran, M. Robert, L. Fensterbank, M. Malacria, E. Lacôte, *Angew. Chem., Int. Ed.* **2010**, *49*, 9166.

[56] X. Lou, J. Lin, C. Y. Kwok, H. Lyu, *Angew. Chem., Int. Ed.* **2023**, *62*, e202312633.

[57] Z. Ye, C. Y. Kwok, S. L. Lam, L. Wu, H. Lyu, *J. Am. Chem. Soc.* **2025**, *147*, 14915.

[83] Y. Cai, M. Wang, Y. Yang, X. Xu, *Inorg. Chem.* **2025**, *64*, 14598.

[84] L. Candish, E. A. Standley, A. Gómez-Suárez, S. Mukherjee, F. Glorius, *Chem. Eur. J.* **2016**, *22*, 9971.

[85] T. V. Nguyen, A. Bekensir, *Org. Lett.* **2014**, *16*, 1720.

[86] S. Wang, D. Hu, W. Hua, J. Gu, Q. Zhang, X. Jia, K. Xi, *RSC Adv.* **2015**, *5*, 53935.

[87] J. Lv, X. Chen, X.-S. Xue, B. Zhao, Y. Liang, M. Wang, L. Jin, Y. Yuan, Y. Han, Y. Zhao, Y. Lu, J. Zhao, W.-Y. Sun, K. N. Houk, Z. Shi, *Nature* **2019**, *575*, 336.

[88] K. Cheng, H.-Z. Yu, B. Zhao, S. Hu, X.-M. Zhang, C. Qi, *RSC Adv.* **2014**, *4*, 57923.

[89] D. Cantillo, C. Mateos, J. A. Rincon, O. de Frutos, C. O. Kappe, *Chem. Eur. J.* **2015**, 21, 12894.

[90] D. C. Fabry, Y. A. Ho, R. Zapf, W. Tremel, M. Panthöfer, M. Rueping, T. H. Rehm, *Green Chem.* **2017**, *19*, 1911.

[91] T. Niwa, H. Ochiai, Y. Watanabe, T. Hosoya, *J. Am. Chem. Soc.* **2015**, *137*, 14313.

[92] Y. S. Sokeirik, H. Mori, M. Omote, K. Sato, A. Tarui, I. Kumadaki, A. Ando, *Org. Lett.* **2007**, *9*, 1927.

[93] M. J. Frisch, G. W. Trucks, H. B. Schlegel, G. E. Scuseria, M. A. Robb, J. R. Cheeseman, G. Scalmani, V. Barone, G. A. Petersson, H. Nakatsuji, X. Li, M. Caricato, A. V. Marenich, J. Bloino, B. G. Janesko, R. Gomperts, B. Mennucci, H. P. Hratchian, J. V. Ortiz, A. F. Izmaylov, J. L. Sonnenberg, Williams, F. Ding, F. Lipparini, F. Egidi, J. Goings, B. Peng, A. Petrone, T. Henderson, D. Ranasinghe, V. G. Zakrzewski, J. Gao, N. Rega, G. Zheng, W. Liang, M. Hada, M. Ehara, K. Toyota, R. Fukuda, J. Hasegawa, M. Ishida, T. Nakajima, Y. Honda, O. Kitao, H. Nakai, T. Vreven, K. Throssell, J. A. Montgomery Jr., J. E. Peralta, F. Ogliaro, M. J. Bearpark, J. J. Heyd, E. N. Brothers, K. N. Kudin, V. N. Staroverov, T. A. Keith, R. Kobayashi, J. Normand, K. Raghavachari, A. P. Rendell, J. C. Burant, S. S. Iyengar, J. Tomasi, M. Cossi, J. M. Millam, M. Klene, C. Adamo, R. Cammi, J. W. Ochterski, R. L. Martin, K. Morokuma, O. Farkas, J. B. Foresman, D. J. Fox, Wallingford, CT, **2016**.

[94] A. D. William Humphrey, Klaus Schulten, *J. Mol. Graph.* **1996**, *14*, 33.

[95] Y. Zhang, F. K. Sheong, Z. Lin, *J. Am. Chem. Soc.* **2024**, *146*, 34591.

[96] L. R. Domingo, E. Chamorro, P. Pérez, *J. Org. Chem.* **2008**, *73*, 4615.

[97] G. M. Sheldrick, *SADABS: Program for Expirical Absorption Correction of Area Detector Data*, University of Göttingen, Germany, **1996**.

[98] G. M. Sheldrick, *SHELXTL Version 5.1. Program for Solution and Refinement of Crystal Structures*, University of Göttingen, Germany, **1997**.
